# Proceeding Abstracts (Invitation Only)

**DOI:** 10.1097/MD.0000000000041090

**Published:** 2025-01-31

## MHBM24001: RESEARCH ON THE PATHWAYS TO ENHANCE MEDICAL STUDENTS’ MENTAL HEALTH LITERACY

Hongcheng Shou^a^, Jing Liu^b^, Xiang Chen^c^

^a^ *The Second Clinical Medical College of Zhejiang Chinese Medical University, Hangzhou, China,* ^b^ *College of Humanities and Management, Zhejiang Chinese Medical University, Hangzhou, China,* ^c^ *Zhejiang Chinese Medical University, Hangzhou, China.*

HS and JL contributed equally to this work.

**Background:** The significance of mental health literacy among medical students is apparent, as it is crucial for their physical and mental well-being, enhances learning efficacy, and establishes a robust foundation for future professional competence. Medical students, immersed in the vortex of rigorous academic and professional demands, frequently exhibit heightened vulnerability to mental health issues. These obstacles may stem from a substantial academic workload, the intricacies of career planning, the complexities of interpersonal interactions, and the disparity between self-expectations and actual outcomes. Consequently, enhancing the mental health literacy of medical students has emerged as a significant concern within medical education. The primary objective of this project is to thoroughly investigate and elucidate the optimal approach to enhance the mental health literacy of medical students, with the intention of developing a tailored suite of educational and support programs that address their specific requirements through empirical methodologies and methodical analysis. This initiative aims to enhance the mental health literacy of medical students, fostering greater psychological resilience, improved self-regulation, and a more optimistic outlook on life amidst the dual pressures of academia and career, while simultaneously reforming the foundational structure of the medical education system.

**Subjects and Methods:** This study employs a comprehensive interdisciplinary approach, utilizing literature analysis to systematically analyze and summarize the present mental health state of medical students.

**Results:** Currently, medical students are encountering numerous significant mental health challenges, including but not limited to excessive academic demands and career-related pressures, heightened anxiety and depression, chronic sleep disturbances, escalating job burnout, and a considerable deficiency in self-efficacy, among other intricate issues. Consequently, the mental health literacy of medical students must be enhanced extensively and effectively.

**Conclusions:** Enhancing the mental health literacy of medical students necessitates the establishment of a comprehensive and systematic mental health education framework to provide a robust basis for their mental health growth. Within this context, we must proactively devise and execute diverse targeted mental health education initiatives to enhance medical students’ comprehension of the importance of mental health and foster their psychological adaptability. The implementation of an effective mental health screening and early warning system is essential for offering prompt support and guidance to medical students facing psychological challenges. Furthermore, it is imperative to enhance the training and development of mental health education instructors, augment the training of mental health education teachers, and continually elevate their professional competence and pedagogical skills.

## MHBM24002: CAMPUS ENVIRONMENTAL FACTORS AND COLLEGE STUDENTS’ MENTAL HEALTH: A MULTIDIMENSIONAL ANALYSIS FROM A SPIRITUAL ECOLOGICAL PERSPECTIVE

Xia Lu ^a^

^a^ *College of Marxism, Shenzhen Polytechnic University, Shenzhen 518055, China.*

**Background:** With the general improvement of education level, the spiritual ecology of college student groups has been increasingly emphasized by academics. As a key factor influencing the psychological, academic, social and other aspects of college students’ development, the campus environment’s role in shaping spiritual ecology has become a hot topic of research. Current research has paid some attention to the improvement of the physical environment, but there is not enough research on the multidimensional impact of the campus environment, especially on how the social and cultural environments synergize on the spiritual ecology of college students.

**Subjects and Methods:** This paper was conducted by searching the literature in, Google Scholar, Web of Science, EI Compendex and SpringerLink, CNKI. Search terms included campus environment, ecology, building design, transportation system, mental health, spiritual ecology, spiritual support, community support, and cultural role. The impact of campus physical, social, and cultural dimensions on the spiritual ecology of universities is analyzed, and the implementation of effective strategies combining the joint efforts of schools, society, and families to enhance the well-being and comprehensive development of college students provides effective ways to jointly promote the healthy development of the spiritual ecology of college students.

**Results:** Research has found that the construction of ecological campus is significantly related to the development of college students’ mental health. The ecological optimization of the physical environment can effectively promote the emotional stability and learning efficiency of college students; the strengthening of the campus social environment can enhance students’ sense of belonging and stress management ability; and the rich and diversified campus cultural environment not only stimulates students’ sense of responsibility and innovation, but also positively influences their values and moral judgments. These findings provide empirical evidence for the comprehensive optimization of campus environment.

**Conclusions:** This study constructs a model of the interaction between the ecological campus environment and the spiritual ecosystem of college students, providing a new theoretical framework for the development of college students’ mental health and the harmonious symbiosis between the environment and people. It comprehensively examines the profound impact of campus physical, social, and cultural environments on college students’ spiritual ecosystems, reveals the importance of optimizing campus environments in alleviating students’ mental health stress, promoting interpersonal interactions, and enhancing academic performance and career planning, and develops a theoretical and practical ecological campus model. This model not only guides university management and policy making, but also promotes interdisciplinary research, injects new insights into the field of mental ecology theory and mental health, promotes theoretical innovation in the integrated study of environmental psychology and psychology, and enriches the development of mental ecology theory.

**Acknowledgments**

This work was supported by a project grant from Shenzhen Education Science “14th Five-Year Plan” 2023 Project: Countermeasures Research on the Influence of Campus Survival Space on Young College Students’ Spiritual Ecology (Grant No.yb23026).

## MHBM24003: STUDY ON THE RELATIONSHIP BETWEEN DIETARY BEHAVIOR AND MENTAL HEALTH OF HIGHER VOCATIONAL COLLEGE STUDENTS IN SANYA AND THE STRATEGIES TO IMPROVE THEIR PHYSICAL HEALTH

Zejun Li^a^, Yawen Sun^a^, ChunYang Liu^a^

^a^ *Sanya City College, Sanya City 572000, China.*

**Background:** Higher vocational students accounted for more than 60% of the total number of Chinese college students, vocational students as a national and social construction and development of reserve cadre, its scientific diet and a healthy diet behavior is directly related to their health, so as to affect the country’s future development and construction. This study was to explore China’s sanya regional higher vocational students’ eating behaviors and the relationship between mental health, thus formulated Scientific dietary behavior improvement strategies to promote the health of vocational students.

**Subjects and Methods:** This study selected sanya three higher vocational college, a total of 1000 students as the investigation object. By using the method of random whole sampling of higher vocational students’ eating behaviors and mental health relations self-designed questionnaire to investigate. Higher vocational students eating behavior questionnaire including basic status, diet quality and dietary psychological, eating behavior influencing factors (external factors and psychological factors) and physical health, a total of four parts. SPSS 25 was used to analyze the survey data, and the dietary behavior of higher vocational students was related to gender, major, economic ability, mental health, etc.

**Results:** The obvious characteristics of students’ dietary behavior in higher vocational colleges affected by mental health include emotional dietary behavior, external dietary behavior and restrictive dietary behavior. Under the influence of psychological factors, the dietary behavior of higher vocational students is extremely enhanced, the randomness of time and space is increased, and the dietary behavior is lack of planning. Under the influence of external environmental factors, dietary behavior is diversified and quality is emphasized. Restrictive factors such as losing weight have a great influence on the dietary behavior of higher vocational students, and the dietary balance is insufficient. The dietary behavior of higher vocational students will naturally be influenced by demographic characteristics such as gender and age.

**Conclusions:** Personal healthy eating knowledge literacy and mental health have internal intervention effects on eating behavior. Environmental factors such as economic level, cultural tradition and the influence of others play a guiding role in individual dietary behavior. Strategies to improve the healthy diet of higher vocational students: the school provides perfect canteens, provides scientific course guidance for nutrition majors, and establishes perfect system supervision. Students should carry out more publicity work and activities to deal with students’ emotional factors. Students actively learn the scientific knowledge of healthy eating and practice scientific and healthy eating behavior.

## MHBM24004: AN EMPIRICAL STUDY ON THE IMPACT OF THE DLRM MULTI-FACTOR QUANTITATIVE INVESTMENT MODEL ON INVESTOR ANXIETY

Yuanhai Yan^a^, Chengying He^b,c^, Xinwen Liu^c,d^, Ruijin Guo^c^

^a^ *College of Artificial Intelligence, Guangzhou Huashang College, Guangzhou, China,* ^b^ *Yangtze Delta Region Fintech Research Institute, Jiaxing University, Jiaxing, China,* ^c^ *School of Economics, Guangxi University, Nanning, China,* ^d^ *School of Economics and Management, Beibu Gulf University, Qinzhou, China*

**Background:** In the realm of investment, In the realm of investment, psychological factors play a significant role in decision-making processes. The multi-factor quantitative investment model is a sophisticated approach that considers various factors to inform investment decisions, which can significantly alleviate the anxiety associated with investment choices. The DLRM (Deep Learning Recommendation Model) multi-factor quantitative investment model, in particular, has been designed to address the complexities of investor anxiety by providing a more nuanced understanding of market dynamics. This model not only considers the financial metrics but also incorporates psychological factors, such as investor sentiment and market perception, which are crucial in mitigating investment anxiety.

**Subjects and Methods:** This paper conducts empirical research on the multi-factor quantitative investment model, utilizing the Deep Learning Recommendation Model (DLRM) algorithm. The research begins with industry selection using random forests, recognizing the correlation between stocks and industries to refine the selection scope. Subsequently, the DLRM is employed to predict the investment portfolio’s performance after stock selection, aiming to achieve the desired return on investment. The paper also introduces improvements to the DLRM, designed to handle large volumes of stock data with sparse features effectively. To further highlight the DLRM’s efficacy in stock forecasting, Long Short-Term Memory (LSTM) networks are incorporated into the empirical study. The DLRM model’s performance is evaluated against traditional investment models, with a focus on its ability to predict market trends and manage risk, which are key factors in reducing investment anxiety.

**Results:** Comparative and empirical analyses are conducted to evaluate the performance of the investment strategy constructed using the random forest and DLRM approach, which achieved an impressive investment accuracy rate of 93.98%. The application of this model can effectively reduce people’s psychological anxiety about stock selection. The study also reveals that the model’s ability to process large volumes of data and identify patterns that may not be apparent to human analysts contributes to a more robust and reliable investment decision-making process. This, in turn, leads to a decrease in the psychological stress associated with investment uncertainty and anxiety.

**Conclusions:** The DLRM multi-factor quantitative investment model offers a significant advantage in managing investment anxiety by providing a more accurate and psychologically informed approach to investment decisions. The model’s success in handling complex data sets and its consideration of investor sentiment demonstrate its potential to transform the way investors perceive and engage with the market. By leveraging advanced algorithms and psychological insights, the DLRM model not only improves investment outcomes but also contributes to the psychological well-being of investors. This study underscores the importance of integrating psychological factors into quantitative investment models to create a more holistic and anxiety-reducing investment experience.

**Acknowledgments**

This research was supported by Special project “Research on the Quality Detection Method of Agricultural Products based on Near-infrared spectroscopy combined with machine learning” in the key fields of ordinary universities in Guangdong Province 2023ZDZX4069

Project name: Research on the Development Strategy of Carbon Financial Market of Hainan Free Trade Port under Fintech Vision “ [2022 Hainan Philosophy and Social Science Planning Project (Base Project); Project No.: HNSK (JD) 22-20]

Guangzhou Huashang College tutorial system project: Research on financial big data technology under the Flink framework (2024HSDS07)

## MHBM24005: THE CULTIVATION STRATEGIES OF CHILDREN’S CRITICAL THINKING, INNOVATION ABILITY AND MENTAL HEALTH FROM THE PERSPECTIVE OF FAMILY EDUCATION

Pengli Zhong^a,b^, Youwen Deng^a^

^a^ *Zhejiang Normal University, Jinhua, China,* ^b^ *Gongqing Institute of Science and Technology, Gongqingcheng, China.*

**Background:** In today’s ever-changing era full of challenges and opportunities, critical thinking enables children to analyze problems rationally. Innovation ability can help children explore the future. And mental health is the cornerstone for children’s healthy growth. However, although these qualities are extremely important for children’s growth, the crucial role of family education in this regard is often overlooked.

**Subjects and Methods:** This study deeply explores the significant meaning of family education in cultivating children’s critical thinking, innovation ability and mental health. Through literature review, it absorbs the wisdom and experience of predecessors. By using case analysis, it shows different results brought by different family education methods with practical examples. And through in-depth interviews with many parents and children, it truly understands their feelings and needs. Thus, it elaborates in detail the profound influence of factors such as family environment, parents’ educational behaviors and concepts on children’s growth.

**Results:** Research shows that the influence of the family in the early stage is like a cornerstone, laying the foundation for children’s future development. Positive parenting methods can greatly promote the development of children’s thinking and abilities. However, excessive protection and doing everything for children will seriously limit their ability to think independently and explore. In addition, emotional support can give children strong confidence and a sense of security, which is of great benefit to children’s mental health. But unfortunately, some families are obviously deficient in this aspect. At the same time, a long-term and continuous cultivation environment is crucial. However, in reality, there are many problems in family education. For example, psychological needs of children are often ignored, which leads to psychological problems such as anxiety and inferiority in children.

**Conclusions:** Family education plays an irreplaceable and important role in shaping children’s critical thinking, innovation ability and mental health. Parents should actively adopt scientific and effective educational strategies, strive to create a good family atmosphere, always pay attention to children’s psychological needs, and provide appropriate guidance and support for children, thus laying a solid foundation for children’s future development.

## MHBM24006: EMOTIONAL INTELLIGENCE AND IDENTITY CRISIS: A STUDY ON THE STRATEGIES FOR PSYCHOLOGICAL HEALTH AND PERSONAL DEVELOPMENT IN COLLEGE STUDENTS

Weiwei Huang^a^, Xianzhong Lin^b^

^a^ *School of Innovation and Creation Design, Shenzhen Polytechnic University, Shenzhen 518055, Guangdong, China,* ^b^ *College of Marxism, Shenzhen Polytechnic University, Shenzhen 518055, Guangdong, China.*

**Background:** In the ever-evolving tapestry of contemporary society, college students are confronted with a unique set of identity challenges. The interplay of globalization, information technology, and the digital network society has thrust them into a crucible of multiculturalism, shifting values, and an unpredictable job market. This period of life, according to Erik Erikson’s developmental stages, is marked by the critical “identity versus role confusion” phase, where the quest for a stable self-identity is paramount. It is a time when the psychological needs for belonging, self-esteem, and autonomy are particularly acute, and the potential for anxiety and identity diffusion looms large.

**Subjects and Methods:** This study delves into the buffering role of emotional management against the identity crisis that pervades the college student population. It is predicated on the hypothesis that proficiency in emotional management is a cornerstone for the development of a resilient self-identity, which is essential for holistic personal growth. Drawing from emotional intelligence theory, the research constructs a framework that encompasses the fundamental elements of self-awareness, self-regulation, empathy, and social skills. These dimensions are scrutinized for their capacity to mitigate the stressors of identity formation and to bolster psychological resilience and adaptability.

**Results:** The study’s results illuminate the essential role of emotional management in navigating the labyrinth of an identity crisis. Emotional cognition, understanding, and regulation are revealed as interwoven processes that empower students to navigate their affective landscapes and maintain equilibrium during the tumultuous journey of self-discovery. Emotional intelligence, in tandem with adept emotional regulation strategies, is found to significantly bolster students’ adaptive capacities within the dynamic terrain of identity construction. This constellation of competencies not only foretells greater resilience to identity-related challenges but also heralds a marked improvement in students’ overall psychological well-being and social functionality. The empirical findings from recent scholarly endeavors underscore the profound benefits of emotional management on the adaptive mechanisms of college students as they engage with the complex dynamics of identity development, including the mitigation of anxiety and the cultivation of a healthy sense of self.

**Conclusions:** This paper systematically elucidates the mediating influence of emotional management on the identity crisis among college students and affirms its profound effectiveness in fostering self-identity stability and personal development. The research underscores the pivotal role of emotional intelligence as a core competency, encompassing self-awareness, self-regulation, empathy, and social skills, which positively influence students’ adaptability amidst the cultural crosscurrents and evolving values. The confluence of emotional management with values education offers a comprehensive strategy for university students to navigate the challenges of identity exploration with greater efficacy, thereby enhancing their psychological health and fostering a robust sense of self in a rapidly changing world.

## MHBM24007: STRESS COPING THERAPY OF TEACHERS’ PSYCHOLOGICAL ADAPTABILITY AND THEIR SUBLIMINAL EMOTION MANAGEMENT

Wenna Wang^a^, Juan Yu^b^, Rong Xi^c^

^a^ *Beijing Aopeng Distance Education Center Co., Ltd, Wuhan, Hubei 430074, China,* ^b^ *Hubei University of Education, Wuhan, Hubei 430205, China,* ^c^ *Hubei Normal University, Huangshi, Hubei 435002, China.*

**Background:** “Psychological adaptability, viewed through the humanistic therapy, positions teachers’ healthy values as their subliminal emotion management. It acknowledges the profound interconnections among personality disorder, psychological intervention and psychological resilience, thereby fostering a holistic development of teachers as complete beings through psychological counseling that spiritual needs. The realization of the emotion management of psychological adaptability requires the establishment of a position of life development in which the body and mind are united, sensibility and rationality are blended, and individuality and sociability go hand in hand, the creation of holistic, open and structured psychological adjustment situations, and the promotion of the process of experiencing psychological support with the dynamism of life.

**Subjects and Methods:** Essentially, this approach constitutes a stress coping therapy—encompassing both psychological adaptability and emotion management.In the study, stratified random sampling was performed to select a sample based on the three types of school scales, less/fewer than 1000 teachers, between 1000 and 2000, and more than 3000 teachers. They were obtained and were the teachers selected in schools.

**Results:** The intrinsic significance of teachers’ psychological adaptability lies in psychological health care and psychological counseling; it emphasizes the importance of emotion management while bridging self-awareness with self-control to transform the mental fatigue into psychological security. To actualize the subconsciousness essence of emotion management, it is imperative to adopt a perspective that integrates body and mind, emotion and reason, individuality and sociality. It is required to adhere to the humanistic therapy, deeply understand the identity value of the psychoanalysis, implement the concept of emotion management, strengthen the guidance of correct values, emphasize the cultivation of psychological intervention and psychological adjustment, and implement the fundamental task of cultivating moral integrity into specific psychological rehabilitation. Strengthen the stress coping in humanistic therapy, enhance the unity of knowledge and practice, combine learning and thinking, give full play to the healthy values of emotion management, guide teachers to participate in group psychotherapy.

**Conclusions:** To strengthen the psychological defense mechanism of teachers’ personality as a whole through emotion management with psychological tolerance. The humanistic therapy of psychological adaptability is to look at the need for emotional regulation as a whole, highlight the emotion management, integrate the psychological needs and the psychological dependence, and let the psychological resilience become a developmental process of psychological rehabilitation. The realization of the psychological tolerance of teachers’ personality requires the establishment of a psychological defense mechanism in which the body and mind are united, sensibility and rationality are blended, and individuality and sociability go hand in hand, and the promotion of the process of experiencing psychological security with the positive thinking of emotion management. Strengthen the stress coping through emotion management, enhance the unity of identity and self-control, combine motivation and personality, give full play to the healthy values of positive thinking, guide teachers to participate in psychological rehabilitation activities, experience the process of discovering problems, solving problems, constructing psychological defense mechanism, and applying psychological security, strengthen the connection between environmental adaptability and interpersonal relationship sensitivity, resilience to frustration, and self-efficacy, and pay attention to the creation of real situations to cultivate teachers’ psychological tolerance, sense of social responsibility and subconsciousnessstress coping.

## MHBM24008: MITIGATING NEGATIVE LEARNING EMOTIONS IN BLENDED TEACHING MODE: THE MEDIATING EFFECT OF ACADEMIC SELF-EFFICACY ON LEARNING MOTIVATION AND LEARNING ENGAGEMENT

Ying Xue^a^, Si Chen^a^

^a^ *Xi ‘an International University, Xi’an710077, Shaanxi, China*.

**Background:** In recent years, negative academic emotions have been spreading among Chinese college students. Attitudes such as “Buddha-like,” “lying flat,” and “slacking off,” accompanied by a lack of learning beliefs and motivation, have led to insufficient learning engagement and academic performance. This study aims to explore the mediating effect of academic self-efficacy on learning motivation and learning engagement in a blended teaching mode.

**Subjects and Methods:** The study used a questionnaire survey method, involving 544 college students from a university in Shaanxi, China. These students were participants in a blended teaching model, which combines traditional face-to-face learning with online educational technologies. The survey was designed to gather comprehensive data on students’ learning motivations, engagement levels, academic self-efficacy, and their psychological states. The detailed questionnaire aimed to understand the intricate dynamics between these variables and how they influence students’ academic and psychological outcomes.

**Results:** The results showed that the learning motivation, learning engagement, and academic self-efficacy of college students were at high-intermediate level. Art students scored significantly higher than humanities and science students in external learning motivation, overall learning motivation, and the focus dimension of learning engagement. There were significant differences in academic self-efficacy among students of different majors. In terms of gender, male students scored significantly higher than female students in learning engagement and academic self-efficacy, but there were no significant gender differences in overall learning motivation and both internal and external learning motivation. Learning motivation, learning engagement, and academic self-efficacy were significantly positively correlated. Learning motivation not only directly predicted learning engagement but also indirectly influenced learning engagement through the mediating effect of academic self-efficacy. In other words, the stronger the learning motivation, the higher the academic self-efficacy, which in turn increased learning engagement. These findings underscore the importance of fostering strong learning motivation and self-efficacy to enhance students’ engagement in their academic activities.

**Conclusions:** The conclusions indicate that in blended teaching environment, the strength of college students’ learning motivation is directly linked to their levels of learning engagement and academic self-efficacy. By strengthening these factors, educational institutions can effectively mitigate negative academic emotions and reduce the impact of negative emotions and behaviors on learning engagement. This not only improves academic performance but also contributes to the overall mental health and well-being of students, fostering a more positive and productive academic environment. The study emphasizes the need for educational policies and practices that support and enhance students’ psychological needs, as well as their academic goals, to create a holistic approach to education that values both the mental health and academic success of students.

**Acknowledgements** Scientific Research Program Funded by Education Department of Shaanxi Provincial Government. “Research on the Pathways for Enhancing the Digital Application Skills of International Chinese Teachers in Shaanxi” (Program No.23JK0200)

## MHBM24009: CROSS-CULTURAL IDENTITY AND MENTAL HEALTH: ESSENTIALISM AND CONSTRUCTIVISM IN 20TH-CENTURY AMERICAN ETHNIC WOMEN’S LITERATURE

Yang Liu ^a^

^a^ *School of Foreign Languages, Tianjin University, Tianjin 300350, China*

**Background:** This paper explores two primary approaches to studying cross-cultural identity in 20th-century American ethnic women’s literature: essentialism and constructivism. Cross-cultural identity serves as a crucial bridge for individuals to understand and integrate diverse cultural backgrounds and is essential for fostering harmonious coexistence among different cultural groups. However, current domestic research on cross-cultural identity in American ethnic women’s literature lacks depth in identity theory and interdisciplinary integration, often neglecting psychological aspects such as emotional regulation, mental health needs, and overall well-being.

**Subjects and Methods:** The study focuses on the cross-cultural identity construction in the narratives of ethnic women writers, examining their responses to multiple oppressions and their creation of a “third space” for cultural identity through literary means. It employs narrative discourse analysis, postcolonial and feminist theories, and historical comparisons to explore how these writers address identity conflicts and resolutions. The research highlights the psychological dimensions of identity construction, emphasizing how these narratives cater to the emotional needs and mental health of women navigating complex cultural landscapes.

**Results:** The findings reveal that American ethnic women writers often depict the psychological struggles associated with cross-cultural identity, including anxiety, emotional distress, and the search for well-being. Essentialism views cultural identity as rooted in historical and ancestral heritage, providing a stable core for individuals. In contrast, constructivism emphasizes the fluidity and dynamic nature of identity shaped by migration experiences and social interactions. Both approaches underscore the importance of addressing psychological needs and emotional regulation in the process of identity formation.

**Conclusions:** Understanding the essentialist and constructivist perspectives on cross-cultural identity in ethnic women’s literature is crucial for advancing research on identity theory and enhancing interdisciplinary studies. Integrating psychological aspects such as emotional well-being, stress management, and the fulfillment of mental health needs provides a more comprehensive understanding of identity construction. This research offers valuable insights for improving cross-cultural identity studies and supports the development of narratives that promote mental health and emotional resilience among individuals in multicultural contexts.

## MHBM24010: MENTAL HEALTH BASED ON THE THEORY OF PERSONALITY STRUCTURE: TAKE THE CHARACTER DEVELOPMENT IN ‘THE PIG, THE SNAKE, AND THE PIGEON’ AS AN EXAMPLE

Yihui Ma ^a^

^a^ *Sichuan University of Culture and Arts, Mianyang, Sichuan, 621000, China.*

**Background:** Adolescent mental health is a topic of concern to society. The film ‘The Pig, the Snake, and the Pigeon’ uses metaphorical art to show the complexity of human nature and multiple reflections on society. The film uses characterisation and plot development to show the protagonist’s personality changes and psychological struggles at different times, emphasising the importance of a balanced personality structure for mental health. Using this film as an example, exploring the impact of an unbalanced personality structure on adolescent mental health and preventive measures has important social value.

**Subjects and Methods:** This paper explores the relationship between personality development and mental health through a textual analysis of the actions, dialogues and psychological changes of the protagonist Chen Guilin in the movie ‘The Pig, the Snake, and the Pigeon’. A textual analysis method is used to interpret in detail the protagonist’s id, ego and superego in the movie, to further understand the psychological mechanisms behind individual behaviour, and to analyse how these psychological factors drive the plot development and character changes. Symbolic methods are also used to interpret the mental health messages conveyed in the movie.

**Results:** The research results show that Chen Guilin’s personality development, from the transformation of the id to the superego, reveals the important role of personality development on mental health, especially in dealing with violent tendencies and the process of psychological self-redemption, which shows obvious improvement in mental health. In the film, Chen Guilin completes the psychological transformation of self-redemption and self-identification, from being wanted by the gang and the police to punishing the wicked and praising the good for social justice, achieving a moral reshaping from individualism to social justice, revealing the impact of social environment, family background, personal psychology and other factors on the construction of adolescents’ personality and mental health.

**Conclusions:** The film ‘The Pig, the Snake, and the Pigeon’ reveals the importance of personality structure on the mental health of adolescents by focusing on the psychological transformation of Chen Guilin’s id, ego and superego. By realistically recreating the psychological state of juvenile crime, the film guides the audience to pay attention to the phenomenon of personality structure imbalance hindering the mental health development of adolescents, and to reflect on social reality issues such as adolescents’ anti-social behaviour. The film calls for attention to be paid to family harmony, social invisible education and the construction of adolescents’ personal psychological defence mechanisms, so as to help adolescents correctly perceive and grasp the psychological challenges in their growth. It is of great practical significance and social value.

## MHBM24011: DEVELOPMENT AND VALIDATION OF A SCALE FOR MEASURING PSYCHOLOGICAL FEAR IN NOVICE SWIMMERS IN HIGHER EDUCATION INSTITUTIONS

Dongyu Li^a^, Wenmin Li^b^, Kun Liang^a^

^a^ *School of Physical Education, Guangzhou Sport University, Guangzhou, China,* ^b^ *Graduate School, Guangzhou Sport University, Guangzhou, China.*

**Background:** Within the context of higher education, novice swimmers often grapple with psychological fear, a factor that profoundly impedes the advancement and efficacy of swimming instruction. Despite the critical need for tools to measure this fear, a tailored assessment instrument specifically for novice swimmers in college settings has been notably absent. This study addresses this void by developing a psychological fear scale that is calibrated to the unique challenges of college swimming courses, incorporating psychological fear theories, the distinctive features of college swimming, and the intrinsic aspects of novice swimmers’ hydrophobia.

**Subjects and Methods:** The scale’s reliability and validity were meticulously evaluated through data collected from 1330 students across 12 colleges in Guangdong Province, China. A comprehensive scale was crafted following item analysis and exploratory factor analysis, which was further subjected to confirmatory factor analysis, reliability testing, and validity assessments.

**Results:** The refined psychological fear scale is structured around four dimensions: subjective manifestation, somatic symptoms, avoidance thinking, and fear thinking, comprising a total of 23 items. The scale exhibits an impressive internal consistency coefficient of 0.851, with each dimension demonstrating coefficients ranging from 0.781 to 0.973. Moreover, the test-retest reliability coefficient is an encouraging 0.797.

**Conclusions:** The psychometric properties of the psychological fear scale, including its fit, reliability, and validity, align with the stringent standards of academic measurement, offering a robust tool for assessing the psychological fear experienced by novice swimmers in college settings. This scale not only fills a critical gap in the assessment of psychological fear in swimming education but also provides educators with a valuable instrument to enhance teaching strategies and support the psychological well-being of novice swimmers. The development of this scale represents a significant step forward in the field of swimming instruction, contributing to a more nuanced understanding of the psychological barriers faced by novice swimmers and offering a framework for the development of targeted interventions.

**Acknowledgements**

This work was supported by The Guangdong Province Higher Education Society’s “Fourteenth Five-Year” Plan for Higher Education Research [Grant No.22GQN47]; Research on the Construction of an Evaluation Index System for the Special Abilities of Students Majoring in Physical Education in Universities, funded by the School-administered Scientific Research Project of Guangzhou Sport University (Project Number: XGYB202404); Research on the Path to Enhancing the Teaching Quality of Swimming Courses in Universities under the Background of Digital Empowerment, funded by the Teaching Quality and Teaching Reform Engineering Project of Guangzhou Sport University.

## MHBM24012: ENHANCING STUDENTS’ MENTAL HEALTH AND PROFESSIONAL IDENTITY IN ASEAN LANGUAGE STUDIES

Juntong Liu^a^, Qiuxue Luo^b^, Lin Li^b^

^a^ *Guangxi Minzu University, Guangxi, China,* ^b^ *Baise University, Guangxi Province, China.*

**Objective:** This study explores the impact of cooperation between China and ASEAN countries on the mental health and professional identity of students studying ASEAN languages. It aims to identify challenges faced by these students, particularly in the post-pandemic era, and to propose strategies for enhancing their educational experience and mental well-being. By integrating psychological health considerations, this research underscores the significance of mental well-being as a cornerstone of professional development and academic achievement.

**Subjects and Methods:** The research involved in-depth focus group interviews with 18 students majoring in Thai and Vietnamese languages at Baise University in Guangxi Province, China. Participants shared their experiences regarding personal, professional, and pandemic-related factors that influence their mental health and professional identity. The study analyzed these qualitative insights to construct a model illustrating the influence mechanism of students’ ASEAN language professional identity, emphasizing mental health as a critical component.

**Results:** Three primary factors were identified as affecting students’ professional identity: personal circumstances, the professional situation within their field, and the effects of the pandemic. The findings revealed that personal factors, such as mental health and resilience, significantly impacted students’ professional identity. Additionally, the professional context, including opportunities for collaboration and career prospects, influenced students’ engagement levels. The pandemic emerged as a moderating factor, exacerbating mental health challenges and impacting students’ perceptions of their professional identity in language studies. The mental health of students plays a critical role in their ability to cope with the demands of their field and the disruptions caused by the pandemic.

**Conclusions:** To foster the development of ASEAN language majors in higher education, it is essential for the Chinese government, universities, and students to collaborate effectively. The government should prioritize a strategic approach to international cooperation in higher education, addressing mental health as a vital aspect of student development. Universities must establish supportive environments that promote mental well-being and professional collaboration. For students, adopting a growth mindset and engaging in active learning are crucial for enhancing their core competencies and strengthening their professional identity in ASEAN languages. By addressing these factors, stakeholders can improve the educational experience and mental health of students, ultimately benefiting the broader internationalization of higher education in China.

## MHBM24013: MEDICAL DETECTION AND MENTAL HEALTH INTERVENTION OF TAJIK AND KIRGIZ FEMALE MIDDLE SCHOOL STUDENTS UNDER THE INFLUENCE OF DIFFERENT LIVING ENVIRONMENTS

Zhiwei Li^a^, Wenhuan Huang^b^, Qinqin Yang^c^, Yang Liu^b^, Wenting Hao^b^

^a^ *University of Sanya Instituer of physical, Sanya, 572022, China;* ^b^ *Hainan College of Economics and Business, Haikou, 571127, China;* ^c^ *Xinjiang Police Academy, Urumqi, 830011, China*

**Background:** Tashkurgan Tajik Autonomous County is located in the southwest of Xinjiang Uygur Autonomous Region, more than 4,000 meters above sea level, and is the only county in China that borders the three countries. It has an arid and semi-arid plateau climate. Long and cold winter, drought and little rain, sufficient light energy, insufficient heat energy, large temperature difference between day and night, in the long-term high-altitude anoxic living environment, it is easy to cause Tajik teenagers to form impatient and anxious personality psychological characteristics, and small body shape. Therefore, the study concluded that the Tajik nationality has formed its own unique personality and psychological characteristics, national style and physical health characteristics in the transformation and adaptation to the harsh natural environment.

**Subjects and Methods:** The research group selected Tashkurgan Tajik Autonomous County of Xinjiang Province, which has the largest Tajik population, as the research object of students’ mental health and physical health monitoring. A total of 600 male and female middle school students aged from 13 to 17 were randomly selected as research objects to investigate, analyze and study 12 medical indicators related to body shape. At the same time with the whole country, especially the Kirgiz students personality psychological characteristics and physical health related medical indicators statistics and inference, the conclusion is drawn.

**Results:** Genetic factors, natural living environment conditions, local nutrition and health conditions, sports mental health education facilities are insufficient. The mental health and physical health characteristics of Tajik and Kirgiz students are lower than the national average, and Tajik girls have a lower body size index and smaller appearance, but strong personality and psychological sensitivity, high maximal oxygen uptake and strong oxygen absorption capacity.

**Conclusion:** through analysis, research and expert consultation, we should carry out mental health education and medical sports intervention for Xinjiang Tajik students from the following aspects. 1. Education authorities at all levels should attach great importance to improving the living and health environment of Tajik students and the scientific matching of school canteens with the nutritional structure of the family. 2. In the full implementation of the educational guidelines, priority should be given to physical and mental health and high priority should be given to medical care, physical education and hygiene in schools in Tajikistan, ensuring one hour of physical exercise per day. 3. Strengthen Health Consciousness through medical sports, develop the habit of lifelong physical exercise, and cultivate medical ability. 4. In the course of physical and health education, comprehensive physical and health education and exercise training, especially strength training, should be strengthened for female students in Tajikistan in order to improve their physical and mental health in an all-round way.

**Acknowledgements**

1. Supported by Leisure Sports Tourism Research and Innovation Team of Hainan Economic and Trade Vocational and Technical College (HNJMT2023-104)

2. 2024 Hainan Provincial Philosophy and Social Sciences Planning Project: Research on the Development Strategy of Cultural and Sports Tourism in the Core Area of the South China Sea from the Perspective of Strengthening the Chinese National Community, No. HNSK (YB) 24-70

## MHBM24014: RESEARCH ON IMPROVING MENTAL HEALTH OF THE ELDERS WHO LOST THE SINGLE-CHILD BY PHYSICAL EXERCISE INTERVENTION

Yuwei Liu^a^, Yanfang Huang^b^, Zekai Xu^a^, Meng Hu^a^, Mingzhe Li^a^, Yane Xiong^a^, Xiaoliu Liu^a^

^a^ *Wuhan University of Science and Technology, Wuhan 430065, China,* ^b^ *Geriatric Hospital Affiliated to Wuhan University of Science and Technology, Wuhan 430065, China.*

YL and YH contributed equally to this study.

**Background:** To investigate the effects of physical exercise intervention on the mental health and sleep quality of elderly people who have lost their only child. Elderly people who have lost their children are commonly referred to as “Aged Shidu Person”.

**Subjects and Methods:** In October 2023, 100 aged Shidu person were intervened with physical exercise, including Tai Chi, brisk walking, and square dancing, for a duration of 2 months. The physical exercise intervention is divided into 2 months, In the first month, the main purpose is to develop sports interest, body coordination ability and exercise habits. In the second month, help aged Shidu Person with social communication and interpersonal communication during exercise. Practice for 50 minutes every morning or evening, all three are aerobic exercises and are led by exercise enthusiasts. The Health Promoting Lifestyle Scale (HPLP), Pittsburgh Sleep Quality Index (PSQI), Self Rating Anxiety Scale (SAS), Self Rating Depression Scale (SDS), and Symptom Checklist 90 (SCL-90) were used to compare the lifestyle, sleep quality, and mental health status of aged Shidu Person before and after physical exercise intervention.

**Result:** The score after physical exercise intervention in HPLP scale and PSQI of the aged shidu person is higher than that before (*p*＜0.05). The scores of SAS, SDS and anxiety, depression, paranoid ideation, obsessive-compulsive symptom, terror, interpersonal sensitivity and the total score in SCL-90 scale after the physical exercise intervention are lower than those before physical exercise intervention, and the difference is significant in statistical sense (*p*＜0.05) either.

**Conclusion:** Engaging in physical exercise can effectively reduce anxiety and depression among Aged Shidu Person, improve their mental health, enhance sleep quality, and elevate their overall quality of life. It enables them to face reality with a more positive lifestyle, thereby alleviating the social challenges associated with their elderly care.

**Acknowledgements**

This work was supported by Research Project on the Construction and Teaching of Online Courses for National Medical and Pharmaceutical Graduate Students (YXC2022-02-02); Wuhan University of Science and Technology Teaching Project (2023Z017); Wuhan University of Science and Technology Teaching Project (2023X047); Provincial College Students Innovation and Entrepreneurship Training Program Project of Wuhan University of Science and Technology (S202410488173).

## MHBM24015: EXPLORING THE IMPACT OF CONSUMER PSYCHOLOGICAL HEALTH ON CROSS-BORDER E-COMMERCE EXPORT PERFORMANCE: A MULTIDIMENSIONAL ANALYSIS

Hongbin Tong ^a^

^a^ *School of Business Intelligence, Zhejiang Institute of Economics and Trade, Hangzhou 310018, China.*

**Objective:** This study examines the relationship between consumer psychological health and the export performance of cross-border e-commerce enterprises. Focusing on three critical dimensions—security, shopping experience, and logistics services—it investigates how consumer perceptions in these areas influence their psychological well-being, including trust, satisfaction, and loyalty, and how these psychological factors subsequently drive export performance. By integrating psychological health into the analysis, the study provides actionable strategies for enterprises to enhance both consumer well-being and business outcomes, promoting sustainable growth in international markets.

**Methods:** The research adopts a mixed-method approach. Initially, a comprehensive literature review identified key factors influencing consumer behavior in cross-border e-commerce, with a focus on psychological health. An index system was developed, incorporating three primary dimensions and eleven secondary indicators. A questionnaire survey was conducted among professionals in cross-border e-commerce enterprises, resulting in 186 valid responses. Using SPSS software, reliability and validity tests, correlation analysis, and multiple linear regression were performed to assess the relationships between the identified factors, consumer psychological health, and export performance. The study further examines how improvements in consumer perceptions contribute to psychological health and drive enterprise success.

**Results:** The findings reveal that security, shopping experience, and logistics services significantly and positively influence export performance. Among these, shopping experience has the strongest impact (β = 0.332, p < 0.001), followed by security (β = 0.324, p < 0.001) and logistics services (β = 0.255, p < 0.001). The analysis also highlights that improved consumer perceptions in these areas enhance psychological health by fostering trust, reducing anxiety, and improving satisfaction. This, in turn, strengthens consumer loyalty and drives higher export performance. The study underscores the dual benefit of integrating psychological health considerations into cross-border e-commerce strategies, benefiting both consumers and businesses.

**Conclusions:** Cross-border e-commerce enterprises should adopt a consumer-centered approach that prioritizes psychological health by enhancing security measures, streamlining shopping processes, and optimizing logistics services. Policymakers are encouraged to support these efforts by establishing consumer-friendly regulations and fostering international cooperation to address industry challenges. This research contributes to the growing body of literature on the intersection of psychological health and e-commerce, offering practical insights for enterprises, researchers, and policymakers to improve consumer well-being and strengthen global competitiveness.

**Acknowledgments**

This research was supported by the Fundamental Research Funds for the Provincial Universities, Zhejiang Institute of Economics and Trade (Grant Number: 21SBYB05).

## MHBM24016: RESEARCH ON THE INFLUENCE OF SCHOOL ORANIZATIOINAL JUSTICE AND MORAL DISENGAGEMENT ON TEACHERS’ MISBEHAVIOR IN PRIMARY AND SECONDARY SCHOOLS

Xiangqian Wei ^a^

^a^ *School of Psychology, Qufu Normal University, Qufu, Shandong 273165, China.*

**Background:** For a long time, causations of organization unethical behaviors have become an important problem that puzzled managers and scholars in organization management. In this study, teachers’ misbehavior is understood as a kind of deviant behavior of teachers in the context of school organization. The core issue of this study is whether teachers’ individual psychological variables and school organizational situation variables will affect teachers’ misbehavior. The purpose of this paper is to examine the influence mechanism of school organizational justice and teacher moral disengagement on teachers’ misbehavior.

**Subjects and Methods:** The paper employs a quantitative study to investigate 676 primary and secondary school teachers by the scale of moral disengagement, perceived organizational justice and teachers’ misbehavior. Using multiple regression, this study analyzed respectively the influence of moral disengagement and perceived organizational justice on teachers’ misbehavior, and the moderating effect of perceived organizational justice on the influence of moral disengagement on teachers’ misbehavior.

**Results:** The results of multiple regression analysis show that moral disengagement positively predicts teachers’ misbehavior, and perceived organizational justice negatively predicts teachers’ misbehavior. The results of moderating effect analysis show perceived organizational justice negatively moderates the relationship between moral disengagement and teachers’ misbehavior.

**Conclusions:** The results of this study show that moral disengagement and perceived organizational justice are important influencing variables of teachers’ misbehavior. Moral disengagement is the internal motivating factor of teachers’ misbehavior, and perceived organizational justice is the external inhibiting factor of teachers’ misbehavior. The fair environment of school organization will effectively restrain the influence of teachers’ moral disengagement on their misbehavior. The limitation of this study is that it adopts a cross-sectional research method, and its results cannot determine the causal relationship between the variables. Therefore, it is necessary to use experimental method or longitudinal study method to further study the causal relationship of these variables in the future. In conclusion, the study enriches the research about teachers’ misbehavior, and discusses the implications of these findings for future research and for school management practice.

## MHBM24017: PSYCHOLOGICAL ANALYSIS OF THE PEASANT CLASS IN THE CONSTRUCTION OF THE JUDICIAL POWER SYSTEM IN THE SOVIET UNION REGION

Wei Hu ^a^

^a^ *Anyang Normal University, Anyang 455000, Henan, China.*

**Background:** The construction of the judicial power system in the Soviet Union Region is of great significance, providing a blueprint and reference for the judicial power system in the base areas and in New China. Furthermore, it constitutes an important element in the revolution of the democratic regime of workers and peasants. The Soviet Union Region is situated in a socio-economically disadvantaged rural context, where the prevailing small peasant ideology constrains the development of the regime. It is therefore vital to comprehend the psychological expectations of the peasantry and address their needs.

**Subjects and Methods:** The composition of the social classes in the modern countryside is analysed, and psychological, sociological and legal methods are employed to clarify the perception of each class on the social revolution. Furthermore, the psychological expectations and tolerance of the peasant class are analysed.

**Results:** The analysis of the rural class structure and ecological situation, coupled with an exploration of the psychological expectations of each class, is of great significance. In reflecting on the adaptive strategy of constructing the judicial power system of the Soviet Union Region regime, we conclude that the experience was guided by three key factors: revolutionary ideals, the satisfaction of the psychological expectations of the peasants, and the guidance of the latter in the reconstruction of the judicial power system.

**Conclusions:** The judicial power system has a role to play in the present era. In the Central Soviet Union Region, the establishment of peasants’ associations constituted a significant turning point in the reconstruction of the judicial power system and the establishment of its operating rules. An understanding of the psychological makeup of each social entity, which had disparate considerations and responses based on their respective interests, proved instrumental in grasping the intricacies and challenges inherent to the construction of the judicial power system in the Soviet Union Region.

**Acknowledgments**

This paper has been selected for funding by the 2019 Henan Provincial Philosophy and Social Science Planning Program: “Justice and Revolution: a Study of the Tensions and Limits of Justice in the Central Soviet Union Region” (2019BFX001).

## MHBM24018: UNLOCKING BRAND LOYALTY THROUGH HOTEL LIVE STREAMING: ADDRESSING CONSUMER PSYCHOLOGICAL NEEDS

Jin Wang^a,b^, Alaa Abukhalifeh^c^, Jiaying Liu^a^

^a^ *School of Tourism and Social Management, Nanjing Xiaozhuang University, Nanjing, China,* ^b^ *Department of Hospitality and Tourism, Woosong University, Daejeon, SouthKorea,* ^c^ *International Hotel Management (IHM), Kyungdong University-Global Campu, Gosung, Gangwondo, South Korea.*

**Background:** In the fast-paced hospitality sector, recognizing and responding to consumer psychological needs is crucial for boosting customer satisfaction and cultivating brand loyalty. The emergence of live streaming as a marketing strategy opens new avenues for hotels to engage consumers on a deeper psychological level. This interactive, real-time platform addresses both the emotional and cognitive needs of consumers by offering personalized and engaging experiences. As hotels embrace live streaming, particularly in the post-pandemic context, the significance of these psychological elements in reinforcing brand loyalty has become increasingly important.

**Subjects and Methods:** This research explores the effects of live streaming marketing strategies used by hotels on customer brand loyalty. It emphasizes the principles of positive psychology and the psychological needs of consumers. We applied a quantitative approach to assess key variables such as perceived interactivity, emotional engagement with live streaming hosts, and content quality during live sessions. The research gathered 259 valid responses from consumers who participated in hotel purchases through live streaming. We used SPSS software to perform regression analysis, revealing the complex relationships between these psychological factors and their impact on consumer satisfaction and brand loyalty. This analysis seeks to provide insights into how effective live streaming marketing fosters enduring loyalty among hotel customers, based on their emotional and psychological experiences during the buying process.

**Results:** The empirical investigation reveals that the perceived interactivity inherent in live content, along with the efficacy of host performance and the caliber of hotel products and services, plays a significant role in amplifying customer satisfaction. This heightened satisfaction, it should be noted, acts as a vital mediator that reinforces brand loyalty among consumers. By strategically aligning with consumer psychological needs-particularly through enhanced engagement and interactive elements-hotel establishments can not only elevate consumer satisfaction metrics but also fortify their brand loyalty. The findings underscore that live streaming serves as an effective mechanism for addressing these psychological needs. Thus, it plays a pivotal role in strengthening brand allegiance in the competitive hospitality landscape. This study provides compelling evidence that prioritizing these factors substantially contributes to the overall success of hotel operations.

**Conclusions:** This research presents an extensive psychological model for analyzing live streaming marketing within the hotel sector. By focusing on the psychological needs of consumers, hotels can utilize live streaming to foster deeper emotional bonds, thereby improving long-term brand loyalty. The results yield significant insights and actionable strategies for hotel marketers who seek to refine their live streaming approaches, effectively addressing the psychological and emotional needs of consumers to maintain loyalty and gain a competitive edge.

## MHBM24019: PSYCHOLOGICAL DIMENSIONS IN BIM-BASED DYNAMIC MANAGEMENT OF ENGINEERING PROJECTS: ENHANCING COLLABORATION AND EFFICIENCY

Weiguo Fang ^a^

^a^ *Fuzhou University of International Studies and Trade, Fuzhou 350202, China.*

**Objective:** The application of Building Information Modeling (BIM) technology is transformative for engineering projects, not only in optimizing workflows but also in influencing the psychological dynamics of collaboration among project stakeholders. This study investigates the interplay between BIM-based dynamic management and the psychological factors affecting team collaboration, decision-making, and stress reduction. It aims to identify how BIM tools can enhance project outcomes by fostering a positive psychological environment.

**Methods:** The study employs a case-based approach, analyzing 101 construction projects to evaluate the effectiveness of BIM dynamic management. Psychological surveys were conducted among 50 experts, including government officials, designers, contractors, and supervisors, to assess their perception of collaboration, cognitive workload, and stress levels. A fuzzy mathematical model was developed to quantify the importance of psychological and technical factors in project success.

**Results:** Findings indicate that BIM-based dynamic management significantly improves stakeholder collaboration and reduces cognitive stress by providing a clear visualization of project goals and progress. Key factors, including environmental and policy alignment, demonstrated the highest impact on reducing psychological barriers and fostering cooperative attitudes. The results also reveal that poor model connectivity and lagging schedules negatively influence team morale and efficiency. Addressing these issues through early engagement, accurate positioning, and process control enhances both psychological and operational outcomes.

**Conclusion:** BIM-based dynamic management positively influences the psychological dynamics of engineering teams, improving collaboration and reducing stress. Integrating psychological insights into BIM processes can further optimize project outcomes. Future studies should explore the long-term psychological effects of BIM implementation across diverse project types and cultural settings to refine these practices further.

## MHBM24020: RESEARCH ON THE INTEGRATION OF POSITIVE PSYCHOLOGY IN COLLEGE ENGLISH TEACHING LANGUAGE AND EDUCATION

Zeng Ju^a^, Jihong Li^a^

^a^ *Department of General Education, Chongqing Chemical Industry Vocational College, Chongqing, China.*

**Background:** This paper aims to propose methods of how to cultivate students’ mental health in the process of college English teaching from the perspective of positive psychology, to improve students’ learning ability and promote students’ mental health development.

**Subjects and Methods:** The concept of positive psychology is proposed and deepened, which not only points out the development direction for the reform of university English teaching but also improves the language of university English teaching out of higher requirements. Under this kind of situation, college English teachers should promote the organic integration of teaching language and education, introduce positive psychology concepts into course teaching, and explore a more scientific and reasonable teaching mode for college English courses. In the implementation process, teachers aim to promote students’ mental health, bring positive experiences to students, and stimulate students’ positive emotions. Pay attention to students’ positive traits in the teaching process and give full play to students’ character advantages. At the same time, create a positive learning environment to help cultivate positive emotions and traditional virtues.

**Results:** By analyzing the practical strategies of integrating English teaching language and education in colleges and universities under the background of positive psychology. It effectively stimulates students’ positive emotional elements and successfully achieves the goal of integrating English education language and moral education in colleges and universities. Master the language needs and language level of the main body of students, so that students can face English knowledge learning with a positive attitude, make up for their own shortcomings, and move forward bravely in the face of difficulties in study and life. Cultivate students’ healthy psychology and achieve the purpose of mental health education. It also effectively stimulates the desire for knowledge and enthusiasm of college students, helps students successfully complete learning tasks, and enhances students’ sense of gain and joy in learning activities in a comfortable learning atmosphere and environment.

**Conclusions:** In the context of positive psychology, the integration of English teaching language and education in colleges and universities are extremely important, and there are many complementarities between the two parts. In this situation, colleges can carry out English teaching activities in the above ways, which can significantly improve the teaching efficiency and quality of English courses in colleges and Give full play to the unique educational function of English language teaching in colleges and universities, It will bring students a good learning experience, help students understand the importance of mental health, develop the core literacy of English subjects, develop English language ability, develop positive and healthy learning psychology, and make them become high-quality talents with both ability and political integrity as well as multi-functional talents as soon as possible.

**Acknowledgments**

This work was supported by the Research and Practice on Cultivating the Workplace Foreign-related Competence of Higher Vocational Students under the POA (Production-oriented Approach) Framework and TSCA (Teacher-student Cooperative Assessment) Theory (From Chongqing Higher Vocational and Technical Education Research Association. China).

## MHBM24021: CHINA’S HIGHER VOCATIONAL EDUCATION FUNDING INVESTMENT AND ECONOMIC GROWTH INTERACTIVE RELATIONSHIP RESEARCH: BASED ON LR-VAR MODEL AND PSYCHOLOGICAL NEEDS ANALYSIS

Yong Yang^a^, Guoxiong Chen^b^, Khong Y. W. Eva^c^, Wenhan Zhu^d^, Baojiang Zhu^a^

^a^ *Yancheng Polytechnic College, Yancheng, Jiangsu 224005, China,* ^b^ *Hezhou University, Hezhou, Guangxi 542899, China,* ^c^ *City University of Macau, Macau 999078, China,* ^d^ *Guangzhou Institute of Science and Technology, Guangzhou, Guangdong 510540, China.*

**Background:** With the transformation and upgrading of China’s economic structure and industry, not only is the demand for high-skilled talents surging, but people’s psychological needs for career development are also changing, including the pursuit of job security, the desire to achieve personal values, and the need for continuous knowledge and skill updates. To meet these needs, the Chinese government and education departments have been continuously increasing their investment in higher vocational education. Statistical data shows that from 2011 to 2022, the number of students, total investment, and per capita investment in higher vocational education in China have all shown an upward trend, fully demonstrating the government and society’s emphasis and support for higher vocational education.

**Subjects and Methods:** The research aims to explore the interactive relationship between China’s investment in higher vocational education and economic growth. By adopting the LR-VAR (Linear Regression Vector Autoregression) model for empirical analysis, the study reveals how changes in investment affect economic growth, and further examines the deeper connections between psychological needs satisfaction, education investment, and economic growth. The research methods use per capita GDP as an indicator of economic growth, and per capita total investment in higher vocational education (PCC) as a variable reflecting educational investment. To eliminate heteroscedasticity, the variables are converted to natural logarithms, recorded as LNGDP and LNPCC respectively, and Pearson correlation coefficients, linear regression models, impulse response functions, and variance decomposition are used to deeply analyze the dynamic relationships and mutual influences between the two.

**Results:** The research results show that LNPCC and LNGDP have a significant positive correlation, and according to linear regression analysis, LNPCC has a significant positive effect on LNGDP, with a very high explanatory power (standardized coefficient of 0.971). The variance decomposition results show that over time, the explanatory power of LNPCC on the fluctuation of LNGDP gradually increases, and the fluctuation of LNGDP also has an increasingly large part that can be explained by the changes in LNPCC. The satisfaction of psychological needs, especially the sense of job security and the realization of personal values through higher vocational education, significantly improves the efficiency and innovation capability of the labor market, further strengthening the positive impact of education investment on economic growth.

**Conclusions:** Investment in higher vocational education has a significant promoting effect on China’s economic growth, and this effect becomes more and more pronounced over time. Investment in higher vocational education can significantly promote economic growth and also meet the public’s psychological needs such as a sense of educational achievement and skill improvement, thereby affecting consumers’ psychological expectations and behavioral decisions. Economic growth, in turn, can increase investment in higher vocational education and help meet the public’s psychological needs for educational fairness and employment prospects, thereby driving the sustained prosperity of the consumer market.

## MHBM24022: RESEARCH ON COLOR ANALYSIS OF LION DANCE BASED ON K-MEANS CLUSTERING AND CULTURAL AND CREATIVE DESIGN GUIDED BY PSYCHOLOGICAL NEEDS

Shan Sun^a^, Chufeng Huang^b^

^a^ *Guangdong Literature and Art Vocational College, Guangzhou, Guangdong 511400, China,* ^b^ *Guangdong University of Education, Guangzhou, Guangdong 510800, China.*

**Objective:** The purpose of this study is to quantitatively analyze the color characteristics of the waking lion graphic using the k-means clustering algorithm and explore its innovative application to satisfy psychological needs in cultural creative design. The study aims to make up for the lack of precise quantitative research on specific cultural elements in the fields of color psychology and demand psychology, to provide a new method for the modern transformation of traditional cultural elements, and to explore how to satisfy people’s psychological needs through color design.

**Methods:** 100 high resolutions still images were collected as samples. The images were normalized to a resolution of 2048x1080 pixels and 7×7 mean filtering for noise reduction. Color feature extraction is performed using k-means clustering algorithm with k=10, and the selection of the number of clusters k is based on the elbow rule and visual assessment. The color network model was constructed to visualize the color relationships and proportions. Through the fuzzy comprehensive evaluation method, 30 design professionals and psychologists were invited to assess the effectiveness of color extraction and application, as well as its potential impact on satisfying the psychological need levels such as security and belonging in Maslow’s hierarchy of needs theory.

**Results:** 1) The color of the waking lion image is dominated by white, orange, and yellow, which is consistent with the positive psychological associations of these colors and satisfies basic psychological needs. 2) The color network model demonstrates inter-color relationships, such as complementary pairing of yellow and dark brown, which contributes to psychological well-being. 3) Four modern design color schemes are generated: traditional gold, deep and solemn, fresh blue tones, and black and gold contrasts, which can satisfy high-level psychological needs. 4) The fuzzy Comprehensive evaluation shows that the k-means based method outperforms the traditional method in terms of cultural accuracy, visual appeal and psychological need satisfaction.

**Conclusion:** This study proposes a quantitative analysis framework for traditional cultural color schemes, integrating computational, cultural and psychological approaches. This data-driven approach contributes to the innovative inheritance of traditional culture and the development of cultural and creative industries, while showing great potential in meeting psychological needs at different levels and opening up new directions for the application of color psychology in cultural and creative design.

## MHBM24023: CONSTRUCTION OF AN INDICATOR SYSTEM FOR ASSESSING NETIZENS’ PSYCHOLOGICAL RISKS IN RESPONSE TO OUTBURST OF PUBLIC EVENTUALITY

Chen Wei^a^, Jin Zhao^b^

^a^ *School of Intelligence Policing, China People’s Police University, Hebei, Langfang, 065000, China,* ^b^ *Department of Economic Management, North China Institute of Aerospace Engineering, Hebei, Langfang, 065000, China.*

**Background:** This paper systematically organizes and deeply analyzes the evolving process of netizens’ psychological risks during the early, middle, and late stages of emergency public events. Following the principles of objectivity, scientific rigor, and dynamism, it employs the Analytic Hierarchy Process (AHP) to construct an indicator system for assessing the psychological risks of netizens in emergency public events. By calculating the weights of specific indicators, a more objective classification of psychological risk assessments is quantitatively derived.

**Subjects and Methods:** The study employed the Analytic Hierarchy Process (AHP) to construct an assessment index system for evaluating the psychological risks of netizens during sudden public incidents. This quantitative analysis method, proposed by mathematician T.L. Saaty, systematically organized influencing factors into a hierarchical network model. The system consisted of primary, secondary, and tertiary levels of indicators related to emotional abnormalities, cognitive abnormalities, and behavioral abnormalities experienced by netizens during such events. The construction of the indicator system followed principles of objectivity, scientific rigor, and dynamism to ensure its effectiveness and relevance.

**Results:** The assessment index system revealed that behaviors such as disruption of social order, destruction of public property, loss of control, stress reactions, participation in protests and demonstrations, and personal insults and attacks were main indicators of high psychological risk among netizens during sudden public incidents. Moderate psychological risk was indicated by factors such as lack of trust in the government, decision-making confusion, conflict tendencies, physical discomfort, and confusion about facts. Low-level psychological risk was indicated by pessimistic expectations, organizing gatherings, spreading rumors online, social avoidance, and excessive attention to events.

**Conclusions:** The findings provide an effective tool for quantifying netizens’ psychological risks during sudden public incidents. By accurately identifying and assessing the level of psychological risk, preventive and control strategies can be formulated to maintain public order stability and long-term tranquility. Future research should focus on optimizing and updating the assessment index system, incorporating advanced technologies such as artificial intelligence and big data analysis to enhance accuracy and timeliness.

**Acknowledgements**

This work was supported by the research innovation program for young teachersat the People’s Public Security University of China: “Risk Assessment and Preventionof Public Emergencies under the New Security Pattern” (Project No. ZQN202506).

## MHBM24024: A WAY TO MEET PEOPLE’S PSYCHOLOGICAL NEEDS FROM THE PERSPECTIVE OF SUSTAINABLE DEVELOPMENT IN THE DIGITAL AGE TO ENHANCE URBAN ECOLOGICAL CARRYING CAPACITY IN HENAN PROVINCE

Lijuan Li^a,b^, Shanyong Li^c^

^a^*School of Surveying & Land Information Engineering, Henan Polytechnic University, Jiaozuo 454000, Henan, China,* ^b^*College of Urban and Environmental Sciences, Peking University, Beijing 100871, China,* ^c^*School of Architecture & Arts, Henan Polytechnic University, Jiaozuo 454000, Henan, China*

**Objective:** The purpose of this study is to meet the public’s psychological demand for sustainable development and the public’s expectation for a stable living environment under the premise of improving the urban ecological carrying capacity in Henan Province in the digital era.

**Subjects and Methods:** In this paper, the ecological carrying capacity of Henan Province and people’s psychological needs for the stability and health of the environment are studied. The research method is based on the research of urban ecological carrying capacity and adopts multi-index comprehensive evaluation method.

**Results:** Through the research, the following results are obtained: (1) The city with the highest comprehensive score of ecological carrying capacity in Henan Province is Zhengzhou. Such achievements are attributed to the importance and investment of the Zhengzhou Municipal Government in ecological and environmental protection over the years, including improving forest coverage rate, promoting soil and water conservation projects, and strengthening pollution control. We should put the well-being of the people first and meet their psychological needs for a better life. (2) The evaluation results of ecological carrying capacity factor in Henan Province show that Zhengzhou has the most outstanding performance in the factor aspect, which may be related to the implementation of urban planning and ecological environmental protection policies. Nanyang scored the highest in Factor 2, which may reflect the city’s good performance in resource utilization and environmental management. Xinyang scored the highest in factor 3, indicating that the ecosystem stability of the region is strong, and the protection of natural resources is relatively in place. Zhoukou got the highest score in factor four, which may mean that the land use efficiency and ecological recovery ability of the region are relatively good. On the whole, cities in Henan Province have their own advantages and characteristics in different ecological carrying capacity factors.(3) The index system of urban ecological carrying capacity and the index with the highest correlation coefficient of carbon emission are the whole society’s electricity consumption, and the corresponding correlation coefficient is 0.96, indicating that the whole society’s electricity consumption has the strongest positive correlation with carbon emission.

**Conclusions:** This result shows that we need to pay more attention to and control urban energy consumption to reduce the negative impact on the environment. At the same time, it also reminds us to pay more attention to energy conservation and emission reduction in urban planning and construction, promote the implementation of the concept of green and low-carbon development, meet the people’s psychological expectations for good, and reduce their anxiety about environmental issues.

**Acknowledgements**

The authors acknowledge editor. This work was supported by the Project of Henan provincial social science federation(Grant: SKL-2023-2549), Henan Provincial University Humanities and Social Sciences Project (2024-ZDJH-771), Henan Provincial Key Research and Development Project (241111520700), Henan Polytechnic University Surveying and Mapping Science and Technology “Double first-class” discipline creation project (GCCYJ202430) The authors thank anonymous reviewers and the editor for their valuable comments.

## MHBM24025: EXPLORING THE EMPOWERMENT OF AGRICULTURAL CULTURAL HERITAGE FOR SUSTAINABLE DEVELOPMENT IN RURAL AGRICULTURAL TOURISM INDUSTRY AND ENHANCEMENT OF RESIDENTS’ HAPPINESS SENSE AND HAPPINESS INDEX: A CASE STUDY OF THE DISTINCTIVE TEA CULTURE SYSTEM IN THE HANGZHOU REGION

Zhiwei Jiang^a^, Min Yang^b^, Jing Wang^c^

^a^ *Zhejiang A&F University, Hangzhou, 311300, China,* ^b^ *Zhejiang Technical University of Mechanical & Electrical Engineering, Hangzhou, 310053, China,* ^c^ *Zhejiang Uniview Technology Co., Ltd, Hangzhou, 310051, China.*

**Background:** National rural revitalization strategy’s in-depth implementation aims to inject new momentum for sustainable development into rural areas. A critical aspect of this involves leveraging rural agricultural cultural heritage resources, deeply integrating them with the rural agricultural tourism industry, and enhance the residents’ happiness sense, creating sustainable agricultural cultural heritage projects that empower rural areas with autonomous growth capabilities. Study proposes new ideas, models, and methods to construct sustainable, developable, and replicable agricultural cultural heritage brands to enhance the happiness index in the rural agricultural tourism industry.

**Subjects and Methods:** Involves reviewing the advantages and constraints of agricultural cultural heritage brands and analyzing successful practices in protecting and developing the unique tea culture system in the Hangzhou region. Furthermore, the study examines the rich biodiversity and the contribution of the unique land use systems and agricultural landscapes—formed through long-term co-evolution and dynamic adaptation between rural areas and their environments—to the sustainable social, economic, and cultural development of the local region. From the perspective of the relationship between agricultural cultural heritage and rural industries, there is a close interdependence between the two. Agricultural cultural heritage is an endogenous driving force for the development of rural industries and enhance the residents’ happiness sense. On the one hand, it can promote the development of the primary industry, and on the other hand, it can also promote the revitalization of the agricultural product processing industry, leisure agriculture, and rural tourism industry. It can play a great role in promoting the organic integration of agriculture related industries, culture, and tourism. The excavation and protection of agricultural cultural heritage has become an important lever for promoting rural ecological civilization construction, beautiful rural construction, green agricultural development, multifunctional agricultural development, and rural revitalization. The economic, ecological, and social benefits of protecting and developing agricultural cultural heritage are gradually becoming prominent.

**Results:** Highlight the enhancement of industrial added value and happiness index through content reshaping and path innovation, accelerating high-quality development, and setting new benchmarks for the agricultural tourism integration industry. Agricultural cultural heritage has played an important role in protecting agricultural biodiversity and rural ecological environment, revitalizing and inheriting rural culture, and promoting the development of multifunctional agriculture. Fully utilizing agricultural cultural heritage can not only enhance the intrinsic value of agricultural products and increase farmers’ income, but also have many benefits for the sustainable development and residents’ happiness sense and happiness index of the cities and regions where they are located, with significant economic and social benefits.

**Conclusion:** Emphasizes that empowering the rural agricultural tourism industry through agricultural cultural heritage promotes sustainable development, comprehensive rural revitalization to enhance the residents’ happiness sense and common prosperity.

**Acknowledgments**

Development of Xiaoshan Tower Localization Characteristic Agriculture, Culture and Tourism Integration Project (2023 Hangzhou Science and Technology Development Plan Project) 20231122T08; The Comprehensive Cultural and Creative Product Design of the Wild Child Research Base (2024 technical service project) HW20240057.

## MHBM24026: SIMULATION OF FOREST ECOLOGICAL RESTORATION BENEFITS BASED ON FARMERS’ ENVIRONMENTAL BEHAVIOURAL PSYCHOLOGY

Na Meng^a^, Dong Meng^b^, Ying Zhang^a^, Tianke Liu^b^

^a^ *School of Economics and Management, Beijing Forestry University, 100083 Beijing, China,* ^b^ *Chinese Academy of Natural Resources Economics, 101149 Beijing, China*

**Background:** Ecological degradation is becoming an increasingly serious issue, and it is crucial to consider the comprehensive benefits of forest ecological restoration holistically. As both victims of environmental damage and key participants in restoration efforts, farmers’ willingness to engage in ecological activities significantly impacts the effectiveness of government policies. Understanding the psychological factors that influence farmers’ environmental behaviors can help improve participation in restoration programs, leading to better ecological outcomes and enhanced mental well-being for local communities. When farmers feel empowered and connected to environmental restoration efforts, their sense of mental health and social resilience is positively influenced.

**Subjects and Methods:** This study evaluates how socio-economic factors, population activities, and the natural environment impact forest restoration. The PLUS model was used to simulate forest restoration scenarios for 2030, incorporating the psychological factors that influence farmers’ decisions. The InVEST model combined with the Comprehensive Ecosystem Service Index (CESI) measured key ecosystem services such as carbon sequestration, water conservation, soil protection, and biodiversity. These services were then analyzed to assess the potential mental health benefits derived from improved environmental quality, as well as the overall community well-being under various development scenarios.

**Results:** The findings indicate that under forest restoration scenarios, there could be a 35% increase in the overall benefits in the study area. High-value regions are concentrated in the southeast and a small area in the central part of the country. Both the natural development and urbanization scenarios show similar spatial distribution and benefits. However, strict ecological protection measures significantly enhance multiple ecosystem services, leading to a higher CES score. The study suggests that mental well-being can be boosted through greater involvement in forest restoration, especially if farmers see direct benefits such as improved livelihoods, job creation, and environmental health. However, restoration efforts in arid and semi-arid regions could cause conflicts between water conservation, soil protection, and habitat quality. Such conflicts might increase stress and mental strain for farmers, highlighting the need for balanced and psychologically sensitive approaches to land use.

**Conclusions:** A combined approach, considering multiple scenarios, is more likely to yield comprehensive ecological and psychological benefits for farmers. Governments should incorporate traditional methods alongside digital platforms to promote multi-channel education on environmental governance, employability, and community demonstrations. This will help foster a stronger willingness among farmers to engage in pro-environmental behaviors, ultimately benefiting both the environment and the mental health of local populations.

**Acknowledgements**

This work was supported by a project grant from National Natural Science Foundation of China (Grant No. 72173011), National Key Research and Development Project (Grant No. 2022YFE0115200), Study on Natural Resources Support Policies for Coordinated Regional Development (Grant No. 102121201020000009010), System for the Preparation and Implementation of the 14th Five-Year Plan for Natural Resources (Grant No. 102121201020000009001).

## MHBM24027: A STUDY OF THE MEDIATING EFFECT OF HOME COMPOST BIN DESIGN FACTORS ON CONSUMER PREFERENCE AND PURCHASE INTENTION FROM A MENTAL HEALTH PERSPECTIVE

Cong Chen^a^, Mou Gong^a^

^a^ *Department of Design Innovation, Graduate School of Sejong University, Seoul05006, South Korea.*

CC and MG contributed equally to this study.

**Objective:** The objective of this study is to investigate the influence of design factors of home compost bins on psychological factors, such as preference and purchase intention, from a mental health perspective.

**Subjects and Methods:** The study’s Subjects are on home compost bins with food waste processing functions used indoors. This study used a quantitative research methodology to explore the relationship between the design factors of home compost bins and the consumer psychological factors of preference and purchase intention within a mental health perspective. A quantitative research method was employed, utilizing a Likert scale survey to gather consumer data. Design factors were treated as independent variables, purchase intention as the dependent variable, and preference as the mediating variable in the mediation effect analysis.

**Results:** Design factors have a positive impact on consumer psychological factors and mental health, such as preference and purchase intention. There is a positive correlation between design factors and preferences (β = 0.869, t = 2.207, P < 0.001), as well as between design factors and purchase intention (β = 0.782, t = 3.831, P < 0.001). Furthermore, preferences positively correlate with purchase intention (β = 0.378, t = 9.950, P < 0.001). The indirect effect of design factors on purchase intention is mediated by preferences (effect value = 0.103), indicating a significant mediating effect of consumer preferences.

**Conclusion:** Design factors positively influence purchase intention, with the importance of each factor ranked as follows: strength > material > form > functionality > color. So strength should be a design priority in the design of a home compost bin. When preference positively influenced purchase intention, design factors affected purchase intention through preference. Therefore, increasing consumer purchase intention requires addressing issues related to color, material, functionality, strength, and form design factors. Higher levels of these design factors lead to stronger purchase intentions. Furthermore, the results indicated that preference mediates the relationship between design factors and purchase intention. preference enhances consumer purchase intention for home compost bins, with higher preference leading to stronger purchase intention. Consequently, from a consumer psychology and mental health perspective, when designing home compost bins, designers must fully consider consumer preferences psychological regarding the design, enhance the design level to meet consumer preferences, and thereby increase consumer purchase intention for home compost bins.

## MHBM24028: INNOVATIVE RESEARCH ON THE PSYCHOLOGICAL HEALTH OF PRIVATE LOCAL APPLIED UNDERGRADUATE COLLEGE STUDENTS IN LABORATORY SAFETY EDUCATION UNDER THE BACKGROUND OF COLLEGE ENTRANCE EXAMINATION REFORM

Yuan Teng ^a^

^a^ *Department of Experimental Equipment, Anhui Sanlian University, Hefei Anhui 276000, China.*

**Background:** With the implementation of the new college entrance examination policy, the integration of industry and education, as well as the intersection of disciplines, in private local applied undergraduate universities, have further deepened, becoming a new normal for future professional construction. As a result, the frequency of laboratory use will sharply increase, especially in laboratories with professional training functions, open laboratories, and other places where the frequency of use will be higher, and the safety risks and management difficulties of laboratories will continue to increase. According to statistics, over 88% of laboratory safety accidents are caused by human factors, with weak safety awareness and lack of safety knowledge being the main reasons. In recent years, laboratory safety education has received increasing attention. How to incorporate students’ mental health into the education model to ensure that students learn and research in a safe and healthy environment has become a topic worth exploring.

**Subjects and Methods:** Carrying out laboratory safety education is an important guarantee for enhancing the safety awareness and mental health of teachers, students, and staff, improving their sense of safety responsibility and prevention level, and preventing and curbing accidents. It is a fundamental project for the safety management of university laboratories. The innovation and practice of laboratory safety education models are particularly important as they concern not only the physical safety of teachers and students but also their mental health. The school has introduced new methods in laboratory safety education, mainly including improving the institutional system, hiring full-time and part-time safety officers, conducting safety education and training, constructing online courses, and creating safety manuals. The aim is to achieve certain results in safeguarding laboratory safety work while also addressing the mental health aspects of safety education. It has achieved certain results in safeguarding laboratory safety work. This article also proposes an innovative model of laboratory safety education that is suitable for the development needs of private local applied undergraduate universities.

**Results:** This study innovatively incorporated student mental health into laboratory safety education and achieved significant results. Creating a laboratory safety culture is crucial for maintaining the mental health of students and laboratory safety. By establishing psychological safety standards and strengthening communication between teachers and students, a safe, healthy, and harmonious laboratory environment can be created, which helps students maintain a good psychological state and improve their awareness of experimental safety.

**Conclusions:** It is very meaningful to incorporate student mental health into laboratory safety education for innovative research. In the future, we should continue to explore the deep integration of mental health education and laboratory safety education, and improve the pertinence and effectiveness of laboratory safety education. At the same time, strengthen interdisciplinary cooperation and communication, and jointly promote the development of student mental health and laboratory safety education.

**Acknowledgements**

This work was supported by a project grant from Exploration and Practice of Laboratory Safety Education in Applied Undergraduate Universities under the Background of “Three Comprehensive Education” - Taking Anhui Sanlian College as an Example Project Task Book (Grant No. 22zlgc087).

## MHBM24029: MENTAL HEALTH CULTURE CONSTRUCTION UNDER THE FENGQIAO EXPERIENCE IN THE NEW ERA AND GOVERNMENT-LED MODEL: AN INFLUENCE ANALYSIS BASED ON LDA MODEL

Zhang Liping^a^, Kang Lanping^b^

^a^ *Zhang Liping, Faculty of Language and Literature, Anhui Sanlian University, Hefei City, Anhui Province, China;* ^b^ *Kang Lanping, School of Humanity and Law, Hefei University of Technology, Hefei City, Anhui Province, China.*

**Background:** The Fengqiao Experience, which originated from Fengqiao District in Zhuji City, Zhejiang Province, is a renowned model of grassroots social governance in China. This experience, initially a practice of resolving conflicts in situ by relying on local cadres and the masses, has evolved to encompass a government-led approach to mental health culture construction in the new era. The integration of mental health culture into social governance is crucial for addressing the psychological well-being of residents, especially in the context of community harmony and stability. The government’s role in fostering a mentally healthy community is pivotal, and the Fengqiao Experience provides a blueprint for other regions to follow.

**Subjects and Methods:** This study employs the Latent Dirichlet Allocation (LDA) model to analyze the influence of the government-led mental health culture construction under the Fengqiao Experience on the mental health of residents. Qualitative coding analysis is utilized to deeply explore the core content of this government-led initiative, summarizing practice cases, and reflecting on the inspiration drawn from the New Era Fengqiao Experience.The study focuses on Henan Province, employing interviews and data collection to understand the factors influencing the application of mental health culture construction measures.The LDA model is particularly useful in revealing the underlying cultural supply logic and the mechanisms by which the Fengqiao Experience affects mental health.

**Results:** The study finds that elements such as government leadership, adherence to the mass line, vivid stories of grassroots governance, and the integration of self-governance, rule of law, and rule of virtue are important component of the government-led Fengqiao Experience.The application of the LDA model reveals that the various measures taken under the Fengqiao Experience significantly improve the mental health of residents, reducing anxiety and enhancing community satisfaction.The data-driven analysis provided by the LDA model offers a solid foundation for policymakers to make informed decisions regarding mental health culture construction.

**Conclusions:** The government-led mental health culture construction under the Fengqiao Experience has proven effective in enhancing the mental well-being of community residents.By leveraging psychological interventions and community engagement, the model not only addresses immediate psychological distress but also fosters a long-term mentally healthy environment.The study concludes that the Fengqiao Experience, with its emphasis on mental health, provides a viable framework for other communities to adopt. Zhejiang Province is a successful practice of primary-level social governance, the government-led construction of mental health culture is one of the key elements. Through the LDA thematic model and qualitative coding analysis, this paper deeply explores the core content of the government-led mental health culture construction of the New Era Fengqiao Experience, summarizes its practice cases, and reflects on the inspiration of the New Era Fengqiao Experience, so as to better promote the mental health of community residents to promote the economic and social harmony and stability.

**Acknowledgements**

This work was supported by the Teaching Research Project of Anhui Provincial Department of Education: “Educational Intervention Path for Cultivating Chinese National Community Consciousness in Colleges and Universities,” (Project No. 2022jyxm488). Anhui Provincial Department of Education: "Traditional Cultural Literacy" (Project No. 2023kcszsf207).

## MHBM24030: RESEARCH ON THE STRATEGIES TO IMPROVE THE PERSONALITY CHARM AND TEACHING ABILITY OF COLLEGE TEACHERS

Dapeng Ding^a^, Xiaoxiong Xu^b^

^a^ *Academic Affairs Office, Ningbo City Vocational and Technical College, Ningbo, 315100, China,* ^b^ *Teacher Education College, Ningbo University, Ningbo, 315100, China.*

**Background:** With the context of the rapid development of artificial intelligence, the improvement of teachers’ personality charm and teaching ability faces great challenges. Not only the moral sentiment, ideological character and social responsibility of teachers need to be improved, but also the means of information teaching and the way of combining with teaching content need to be further strengthened.

**Subjects and Methods:** First of all, this study deeply interprets the connotations of TPACK (Technological Pedagogical Content Knowledge), personality charm and teaching ability. On this basis, six works in Z province that won the first prize in the National Vocational College Teaching Ability Competition in 2020 are selected as cases for analysis, and the overall analysis is carried out from the three levels of pre-class, class-in-class and after-class and online-offline mixed teaching, and from the three dimensions of technical knowledge (TK), teaching knowledge (PK) and subject knowledge (CK). To explore the new form of teachers’ teaching ability, so as to get the methods and strategies to enhance the personality charm and teaching ability of college teachers.

**Results:** With an in-depth analysis of teachers’ personality charm and teaching ability, combined with the teaching quality shown by teachers in the first prize of the National Vocational College Teaching Ability Competition in 2020, this study concludes that teachers’ personality charm and teaching ability can be improved mainly from the following aspects: First, we should strengthen teachers’ personality charm and moral cultivation; Secondly, we should carry out mixed teaching to improve teachers’ information-based teaching ability. At the same time, we should carry out diversified evaluation methods to improve teachers’ teaching evaluation ability. Finally, we should carry out flipped classroom teaching and improve teachers’ teaching organization ability.

**Conclusions:** With the development of big data, Internet of Things, cloud computing and artificial intelligence, higher requirements have been put forward for college teachers, and it is a long way to go to improve teachers’ teaching ability. Under the new situation, teachers not only need to master the use of teaching resource platform, information technology, virtual reality technology, mobile teaching APP, intelligent multimedia classroom and future classroom, but also need to constantly innovate in personality charm, teaching methods and teaching evaluation means to meet the individual needs of students in a diversified way.

**Acknowledgments**

This work was supported by Ningbo City Vocational and Technical College 2022 outstanding young teachers research “leading wild goose growth” project. 2020 Ningbo Education Science Planning Project: Based on the “Internet +” background of university teachers’ informatization teaching ability improvement strategy research -- taking the first prize of the National Vocational College Teaching Ability Competition as an example (No.: 2020YGH067). University-level research Project of Ningbo City Vocational and Technical College in 2019: Research on the Improvement of Teachers’ Information-based Teaching ability based on Teaching Ability Competition - A case study of Ningbo City Vocational and Technical College (No.: ZWX19095)

## MHBM24031: RESEARCH ON CUSTOMER EXPERIENCE OF LIVE STREAMING SHOPPING ON REPEAT PURCHASE INTENTION FROM THE PERSPECTIVE OF PSYCHOLOGICAL NEEDS

Shan Li ^a^

^a^ *Weihai Ocean Vocational College Shandong 264300, Weihai, China.*

**Background and Objective:** Within the sphere of rapid advancements in Internet technology and the transformational upgrade of the e-commerce industry, live shopping has emerged as a significant channel for consumer goods and services. This phenomenon is not solely attributable to technological advancements and the proliferation of the Internet. It also profoundly correlates with consumers’ psychological needs and emotional states, particularly in the context of our current fast-paced, high-pressure living environment. The enhancement of customer retention rates and the amplification of customers’ repurchase inclinations by mitigating their psychological anxieties and fulfilling their psychological needs have emerged as pressing concerns for e-commerce entities and live streaming platforms. Addressing the urgent issue of alleviating customer psychological anxiety, fulfilling their psychological needs, and enhancing customer retention rates by amplifying their willingness to repurchase is paramount for e-commerce companies and live streaming platforms. This study embarks from the vantage point of shopping psychological needs, conducting an in-depth exploration of the influence of customer experience and trust in live shopping on consumers’ willingness to repurchase. The aim is to mitigate consumers’ decision-making anxiety and augment the repurchase rate, thereby providing theoretical references and practical guidance for e-commerce companies.

**Subjects and Methods:** This study commences with a comprehensive review of the literature, aiming to dissect pertinent theories from cognitive psychology, customer experience, and repeated purchase intention. Subsequently, a theoretical model is constructed, along with research hypotheses, that explore the relationshipbetween live shopping customer experience and repeated purchase intention. The live shopping customer experience is distilled into four dimensions: perceived experience, information acquisition, emotional value, and social interaction. Data were gathered using questionnaire survey methodologies, and SPSS and AMOS statistical software were employed to analyze the questionnaire data of 338 consumers from diverse backgrounds. This analysis was conducted to validate the rationality and effectiveness of the proposed theoretical model.

**Results:** Research indicates that the rapid expansion of live commerce has led to a series of transformations in consumer psychological needs. The study variables, namely Perceptual Experience, Emotional Value, and Information Acquisition, all have a positive impact on Customer Trust and Repeated Purchase Intention. Customer Trust serves as a mediator in the relationship between Perceptual Experience, Emotional Value, Information Acquisition, and Repeated Purchase Intention. However, Social Interaction does not significantly influence Customer Trust or Repeated Purchase Intention, thus there is no evident mediating effect of Customer Trust between Social Interaction and Repeated Purchase Intention. Further differential analyses reveal that consumers of varying genders, ages, online spending tiers, and educational backgrounds exhibit distinct differences across the relevant variables.

**Conclusions:** By consistently refining the perceived experience within live streaming rooms, expanding information acquisition channels, and intensifying emotional value transmission, we can more effectively cater to the psychological needs of viewers. This approach enhances the sense of camaraderie in live streaming rooms, mitigates anxieties such as distrust in live shopping, and implements differentiated marketing strategies for various user groups. As a result, this elevates the appeal of live streaming rooms and viewer loyalty, thereby increasing customer repurchase intention.

## MHBM24032: A COMPARATIVE ANALYSIS ON VOCATIONAL AND TECHNICAL EDUCATION DEVELOPMENT BETWEEN CHINA AND FOREIGN COUNTRIES BASED ON HAPPINESS PERSPECTIVE

Kefeng Li^a,b^, Yidian Song^c^

^a^ *School of Education, Taylor’s University, Kuala Lumpur, 47500, Malaysia,* ^b^ *School of Tourism and Service Management, Chongqing University of Education, 400065, Chongqing, China,* ^c^ *Faculty of Arts, University of Melbourne, Melbourne, 3010, Australia.*

**Background:** With further development of globalization and internationalization of education, quality and effect of vocational and technical education as an important way to train skilled talents have attracted great attention. As an important indicator of people’s life quality, happiness is closely related to the quality and effect of vocational and technical education. Students’ happiness affects the enthusiasm and efficiency of their learning and determines their future career development and life quality.

**Subjects and Methods:** In vocational and technical education, students’ happiness not only impacts the enthusiasm and efficiency of their learning, but also affects their career development and quality of life. In terms of happiness, this research compared the development status of vocational and technical education in China and abroad, analyzed their respective advantages and disadvantages, and proposed development strategies for vocational and technical education in China based on happiness perspective.

**Results:** Integration of mental health education into vocational and technical education is of great importance to cultivate students’ psychological quality, improve their adaptability and promote their all-round development. The main measures innovating educational concepts, optimizing curricula, reforming teaching methods, improving evaluation systems, strengthening the cooperation between schools and enterprises, improving the level of teachers, paying attention to mental health, etc.

**Conclusions:** With rapid social development and increasingly harsh professional competition, students not only face the pressures of study and employment, but also bear various challenges of life, emotions, interpersonal relations and other issues. Regarding happiness, through comparative analyses of Chinese and global vocational and technical education systems, other stones can be used to propose the construction of vocational and technical education innovation and development strategies to improve students’ happiness, promote high-quality and sustainable development of vocational and technical education in China, and enhance students’ learning efficiency and skill level to improve their self-confidence and sense of accomplishment, and further enhance their happiness level.

## MHBM24033: APPLICATION OF VR VISUAL INTERACTIVE DESIGN IN IMMERSIVE PSYCHOLOGICAL HEALING

Jianli Wang^a^, Wei Chen^a^, Hongying Ma^b^

^a^ *School of Fashion Art, Shaanxi International Institute of Business & Commerce, Xi’an, 712046, China,* ^b^ *School of Literature and Education, Shaanxi International Institute of Business & Commerce, Xi’an, 712046, China.*

**Research Purpose:** With the help of VR virtual reality technology, 3D visual security simulation can reduce the fear and anxiety avoidance of viewers, overcome psychological barriers, meet the needs of immersive psychological healing experience, and summarize its perceptual effect.

**Research objects and methods:** Taking the Gobi Desert terrain in northern China as an example, 3D model is used to simulate the outline of landforms, mapping technology is used to simulate the scheme of broken stones, and numerical model construction and landform model construction are used to analyze. Taking underground space effect as an example, immersive animation design and modeling analysis are carried out, and the main function of the model is to enhance the sense of substitution. Finally, the visual effect displayed by VR technology is used for immersive psychological experience, and the situation and atmosphere of the virtual environment are adjusted in real time to help the viewer achieve a relaxed state.

**Results:** VR technology can break the limitation of the scene frame, and adopt 360-degree panoramic technology, which brings strong psychological perception to the audience. The screen presents the audience with a clever sense of integration that the plot moves with the viewer, and the audience’s emotions will change with the change of perspective, thus truly achieving immersive experience.

**Conclusion:** Continuous immersive psychological experience with VR can infuse the feeling of personal experience of certain activities, and the flow will produce excitement and fulfillment, thus achieving the effect of psychological acceptance. It provides multi-sensory stimulation, including vision, hearing, motion perception, etc., and brings high-fidelity scene immersion experience to viewers. It can effectively help patients overcome psychological obstacles and improve their mental health.

**Acknowledgments**

This paper is the phased achievement of the special scientific research project of Shaanxi Provincial Education Department: Application Research of VR Simulation Technology in Architectural Animation (21JK0051).

## MHBM24034: EVALUATION OF PROGNOSTIC RISK FACTORS FOR SURVIVAL IN GASTRIC CANCER PATIENTS: A RETROSPECTIVE CLINICAL ANALYSIS

Ruilei Tai^a^, Yunpeng Liu^b^

^a^ *School of Basic Medical Sciences, Shanxi Medical University, Taiyuan, China,* ^b^ *Department of Medical Oncology, Clinical Cancer Research Center of Shenyang, The First Hospital of China Medical University, Shenyang, China.*

**Background:** Postoperative recurrence of gastric cancer is a major causes of treatment failure, leading to poor prognosis for affected patients. This study aims to identify prognostic risk factors associated with survival in patients with recurrent gastric cancer after radical surgery based on clinical factors and to construct a survival prediction model. Additionally, it explores independent risk factors for survival prognosis in gastric cancer patients.

**Subjects and Methods:** Clinical information, surgical pathology results, and test results from before the initial treatment, before surgery, and at the time of recurrence were collected from patients with advanced gastric cancer who experienced recurrence after radical gastrectomy at the Department of Oncology, China Medical University. Univariate and multivariate Cox regression analyses were performed using the “survival” package in R to identify prognostic risk factors for survival. The “rms” package in R was used to construct a predictive nomogram model.

**Results:** Both univariate and multivariate analyses identified T stage, pre-treatment serum cholinesterase levels, absolute CD19+ cell count, absolute eosinophil count, and monocyte percentage as independent risk factors for prognosis in patients with postoperative recurrence of gastric cancer. The nomogram model effectively classified patients into high and low-risk groups for prognosis. Factors such as the number of first-line treatment cycles, site of first recurrence, monocyte percentage at recurrence, absolute CD19+ cell count, mean platelet volume (MPV), and the prognostic nutritional index were found to be in-dependent risk factors for survival after recurrence. The nomogram model demonstrated strong predictive performance.

**Conclusion:** A novel clinical prediction model for postoperative recurrence of gastric cancer patients was constructed based on T stage, pre-treatment serum cholinesterase levels, absolute CD19+ cell count, absolute eosinophil count, and monocyte percentage, showing good predictive performance. Furthermore, a survival prediction model for patients after recurrence was developed using the number of first-line treatment cycles, site of first recurrence, monocyte percentage at recurrence, absolute CD19+ cell count, MPV, and the prognostic nutritional index, also demonstrating good predictive capabilities.

## MHBM24035: DIGITAL TECHNOLOGY DRIVES THE ADVANCEMENT ACROSS THE ENTIRE AGRICULTURAL INDUSTRY CHAIN: AN ANALYSIS OF PSYCHOLOGICAL FACTORS, MENTAL HEALTH IMPACTS, AND EXPLORATION OF APPLICATION SCENARIOS

Xiaoyue Zhang^a^, Xuesong Cui^a^

^a^ *College of Economics and Management, Shenyang Ligong University, Shenyang, 110159, China.*

**Background:** This paper endeavors to delve into the transformative potential of digital technology, illuminating its pivotal role in revitalizing and upgrading the intricate tapestry of the agricultural industry chain. It examines how the seamless integration of the digital economy with modern agriculture fosters resilience akin to a steadfast mind, enabling the sector to weather supply chain disruptions with grace and agility. Critically, this study explores the psychological dimensions, including the impact on mental health and well-being of agricultural practitioners. By addressing psychological needs, reducing anxiety, and bolstering self-esteem, this fusion not only fortifies the industry against external shocks but also nurtures an adaptability reminiscent of a flexible mindset, allowing it to pivot seamlessly amidst the ever-shifting landscape of market demands. The paper portrays digital technology as a catalyst that not only infuses the agricultural sector with dynamic energy but also attends to the psychological and emotional health of its workforce, empowering it to harness the full potential of the digital age and cultivate a vibrant, resilient, and adaptable future.

**Subjects and Methods:** The study examines the achievements of big data in the agricultural industry chain, with a particular emphasis on psychological factors and their interplay with mental health outcomes. It investigates mechanisms and application scenarios where digital technology, guided by insights into psychology and mental health, empowers agricultural transformation. Key areas explored encompass the current state of big data application management, the psychological barriers and facilitators of digital technology integration into agricultural production, the pace of digital transformation within the sector, and its implications for the mental well-being of agricultural workers. By considering factors such as anxiety, stress resilience, and self-efficacy, the study aims to provide a holistic understanding of the digital transformation’s impact.

**Results:** The analysis uncovers challenges hindering the digital transformation of agriculture, notably a low level of big data application management and insufficient integration of digital technology into agricultural processes. These challenges are exacerbated by psychological resistance, lack of awareness, and the potential mental health impacts on agricultural practitioners, including increased stress, anxiety, and feelings of inadequacy. Despite these obstacles, the study highlights significant opportunities where digital technologies, supported by a positive psychological mindset and mental health interventions, can significantly enhance the agricultural industry chain’s agility, resilience, efficiency, and the overall well-being of its workforce.

**Conclusions:** This paper underscores the critical role of psychological factors and mental health considerations in enhancing agricultural production transformation through digital technology. To accelerate this transformation, it is imperative to address psychological barriers, promote the adoption of digital technologies in agricultural production, and leverage big data analytics to optimize farming practices. Furthermore, integrating mental health support systems and fostering a supportive work environment are essential. Establishing a comprehensive big data platform nurtured by a culture of innovation, acceptance, and mental well-being can facilitate better data sharing, predictive analytics, enhanced governance, and a healthier workforce. Ultimately, ensuring the availability of skilled personnel, robust infrastructure, financial investments, and a holistic approach to mental health within a supportive psychological framework will drive sustained progress in integrating digital technology within the agricultural sector.

## MHBM24036: INFLUENCE ON EMOTION REGULATION STRATEGIES ON THE MEMORY OF ENGLISH WORDS AMONG MIDDLE SCHOOL STUDENTS

Tingting Wang ^a^

^a^ *Department of Foreign Languages, Bengbu University, Bengbu, Anhui 233030, China*

**Background:** The aim of this study is to explore the influence on different emotional priming conditions on the memory of neutral English words among middle school students, and further explore the influence on different emotional regulation strategies on the memory of neutral English words among middle school students under different emotional states.

**Subjects and Methods:** 80 students from the First Experimental Education Group in Bengbu City were selected as the research subjects. Among them, there are 40 students in a class for seventh grade and 40 students in a class for eighth grade, with 40 boys and 40 girls respectively, with an average age of 13.5 years old. Two groups were divided: a positive high arousal group of 40 people and a negative high arousal group of 40 people. The questionnaire was distributed to investigate the impact of emotional regulation strategies on English words memory among middle school students.

**Results:** Research has shown that emotional arousal has a significant influence on the memory of neutral English vocabulary among junior high school students. Regardless of the emotional conditions, the memory effect of neutral English vocabulary among junior high school students is higher under low arousal conditions than under high arousal conditions; Under positive arousal conditions, cognitive reappraisal strategies are beneficial for improving individual memory performance, while under negative arousal conditions, both cognitive reappraisal and expression inhibition strategies are beneficial for improving individual memory performance.

**Conclusions:** Under negative arousal conditions, the cognitive reappraisal strategy improves the memory of neutral English words in middle school students under different emotional states. Regarding the expression inhibition strategy, this strategy weakens the memory of neutral English words in middle school students under positive arousal conditions and enhances the memory of neutral English words in middle school students under negative arousal conditions.

## MHBM24037: INNOVATIVE RESEARCH ON PSYCHOLOGICAL HEALTH EDUCATION IN AGRICULTURAL UNIVERSITY LIBRARIES: PRACTICE OF INTEGRATING AGRICULTURAL CULTURE INTO GENERAL EDUCATION CURRICULUM REFORM

Xiaoyu Cao^a^, Min Liu^a^, Xu Li^a^, Ying Tang^a^, Jingjing Wang^a^, Wuxia Xu^a^, Wenjuan Wang^a^, Qingshan Wang^a^, Ben Cao^a^, Ying Li^a^

^a^ *Library of Hunan Agricultural University, Changsha 410128, Hunan, China.*

**Background:** Mental health education activities in agricultural universities aim to improve the mental health level, reduce anxiety and depression, and enhance self-esteem and self-confidence of students. From the perspectives of national spirit and agricultural culture, it is necessary to strengthen the education of the awareness of students of self-improvement, self-reliance, and pursuit of progress in farming and reading, labor concepts of service dedication and courage to take responsibility, agricultural practicability of knowing and doing good agricultural works, and healthy and positive mental health. General education teaching must be positioned in agricultural universities according to the characteristics of agricultural culture and agricultural science. Agricultural culture should be embedded in farming and reading, ideological and political, labor, aesthetic, and mental health education programs and integrated into classrooms. Its characteristics in innovative research on mental health education are not obvious.

**Subjects and Methods:** This research employed a multifaceted method to integrate agricultural culture into general education curriculum reform. A “three lectures and three learning” teaching model was introduced, which included the following concepts: 1) Teachers teach, students learn: Traditional classroom instructions focused on integrating agricultural culture into mental health education; 2) Off-campus teaching collaboration: Engaging external educators in teaching sessions while fostering collaborative learning environments between teachers and students; and 3) Multi-level ecological participation: Encouraging students to share their knowledge with primary and secondary school students involved in learning process, promoting peer-to-peer education.

Furthermore, the curriculum incorporates psychological health topics such as stress management, resilience training, and self-awareness exercises to further support the mental well-being of students.

**Results:** Integration of agricultural culture into general education courses effectively solves innovative mental health education problem in agricultural university libraries, combining the requirements of teachers and curriculum teaching and integrating high-quality resources such as farming and reading education, ideological and political education, labor education, mental health education, and aesthetic education. Providing enhanced individual learning experience for students promoted active and joyful learning, highlighted the practicality, practicality, applicability, and innovation of integrating agricultural culture into agricultural general education curricula, and promoted the realization of self-identity and self-worth in student.

**Conclusions:** Based on “three lectures and three studies” teaching model, agricultural students are encouraged to practice and innovate through “cultivation” and self-reliance in their course learning to establish a solid foundation through reading and to understand books and reasons, innovation of mental health education can be sublimated through “teaching”, “learning”, and “practice” process and learning outcomes are reflected in the learning of curriculum and future work.

**Acknowledgments**

The research was supported by Research on the Inheritance and Promotion of Farming Culture in Hunan Province from the Perspective of Rural Revitalization under the Project of Library and Information Working Committee of Colleges and Universities in Hunan Province (2021L010); the practice of agricultural culture education in agricultural university libraries from the perspective of rural revitalization in the general project of young and middle-aged talent pool of Hunan Library Association in 2022 (XHYB1049); Under the guidance of Xi Jinping’s cultural thought the inheritance path and development mode of farming culture in agricultural colleges and universities under the Key Project of Young and Middle-aged Talent Pool of Hunan Library Association in 2023(XHZD1022).

## MHBM24038: THE INPUT AND OUTPUT MODEL BUILDING OF SCIENCE TECHNOLOGY AND INNOVATION CONSIDERING THE HYSTERESIS EFFECT OF THE EXPLICIT HUMAN AND CAPITAL AND IMPLICIT MENTAL HEALTH

Zhenshan Zhang^a^, Xinli Zhao^b^, Xiuhui Qi^a^

^a^ *School of Economy and Management, Qiqihar University, Qiqihar 161003, China,* ^b^ *School of Management, Harbin Institute of Technology, Harbin 150001, China.*

**Background:** the science, technology and innovation (STI) has increasingly become the key driving force for economic and social development. A lot of work has been done to study STI. The scholars have made in-depth and meticulous research on STI. However, there is few literature about the model building to express the specific relationship between the output of STI and the explicit input and implicit mental health. The traditional C-D production model with the current input elements is generally used by scholars to depict the input and output relationship of the STI.

**Subjects and Methods:** the output of STI is affected not only by the current input elements, but also affected by the previous explicit human and capital input and implicit mental health. And therefore, there are shortcomings to describe the input and output relationship of STI by the traditional C-D production function. Firstly, the approach of cross-correlation coefficient is taken to study the lagged relationship between the output and the previous input elements of STI including the previous explicit human and capital input and implicit Mental Health, and find out that the influence of the previous input elements on the output of STI can be described both linearity and exponential distribution. Similar final results can be obtained from both the linear relationship and the exponential distribution. But it is too complicated to derive the input and output function of STI by using linear relationship between previous input and the output, meanwhile, the stability of the result is inferior to the that of the exponential distribution. By much weighing, this paper selects the exponential relationship to describe the hysteresis influence of input elements on output.

**Results:** on the studies above, the input and output model of STI has been mathematically derived. The implicit mental health is included in the random error of the model. In order to further verify the reliability and credibility of the input and output model of STI, the relevant data over the years are introduced into the model, and the fitness between the calculated and original data is ideal.

**Conclusions:** by introducing the lagged elements of the explicit and implicit inputs such as the mental health, the paper obtains the input and output model of STI, and solves the basic problem of STI. The derived model lays a solid foundation not only for the comprehensive and accurate study of the characteristics of STI, but also provides a powerful help for further research in the other relevant areas.

**Acknowledgments**

This paper is supported by “Research on the Phased Evaluation and Spillover Effect of Enterprise Innovation Efficiency in Heilongjiang Province from the Perspective of Innovation Chain” of the scientific research platform project (No.145109325), and also supported by The Nenjiang River Basin Regional Innovation and Industrial Integration Development Laboratory.

## MHBM24039: CONSTRUCTION AND IMPLEMENTATION OF THE THREE-DIMENSIONAL NINE-COMPETENCY CURRICULUM SYSTEM INTEGRATING AESTHETIC EDUCATION AND MENTAL HEALTH EDUCATION FOR COLLEGE STUDENTS

Suitai Sun^a^, Guoxia Hu^b^

^a^ *College of Art, Guangxi Minzu Normal University, Chongzuo 532200, Guangxi, China,* ^b^ *College of Marxism, Guangxi Minzu Normal University, Chongzuo 532200, Guangxi, China.*

**Background:** The aim is to enhance the mental well-being of college students, enrich the content of mental health education, this endeavor seeks to explore vital pathways for the seamless integration and substantial development of aesthetic education and mental health education, Additionally, it addresses educational challenges within higher education and fosters the comprehensive and harmonious development of modern college students.

**Subjects and Methods:** This study employs a combination of literature review and questionnaire surveys. The research encompasses a wide range of literary materials and individuals, including college teachers from diverse disciplines, school administrators, parents, and students of varying ages and genders.

**Results:** The mental health education of college students is highly susceptible to influences from various factors such as family environment, educational setting, and societal conditions. The integration of aesthetic education and mental health education at the college level demonstrates a notably significant impact. Diverse perceptions regarding its efficacy exist among different groups. Specifically, the average improvement rates in teaching effectiveness are as follows: 90.57% for teachers, 92.43% for students, 77.86% for administrators, and 75.43% for parents, revealing a maximum disparity of 15.14%. Among the various groups, there is a consensus that the construction of the three-dimensional nine-competency curriculum system holds significant importance, with an average endorsement rate reaching 83.25%. It is deemed feasible across various levels, encompassing objectives, theory, practice, and technology. Regarding the construction of the three-dimensional nine-competency curriculum system integrating aesthetic education and mental health education: firstly, there is an acceleration in the balanced construction of educational objectives based on the three dimensions of aesthetic education and mental health education. Secondly, a focal emphasis is placed on the goal-oriented horizontal classification, hierarchical vertical stratification, the interlacement of learning modes, and three-dimensional framework based on the implementation mode. This involves the exploratory establishment of the three-dimensional nine-competency curriculum system characterized by its three-dimensional, coordinative, and advantageous features. Lastly, a steady progression is made in the construction of the FCL model’s teaching practice system and the three-dimensional nine-competency curriculum teaching practice system based on the educational concept of three-dimensional nine-competency education.

**Conclusions:** The research has facilitated the systematic construction of the curriculum system, enhanced the institutional nature of curriculum establishment, brought about a breakthrough in terms of educational philosophy, fostered innovation in pivotal measures for educational reform, and achieved widespread equilibrium development of aesthetic education and mental health education. Consequently, schools have not only redefined their curriculum objectives, cultivated students with heightened aesthetic sensibilities, outstanding core competencies, and robust mental health, but also fostered the establishment of distinctive school-based curriculums under the banner of “unique brand for each school and distinct characteristics for each faculty”.

**Acknowledgments**

This work was financially supported by Class B Project of the 2024 Guangxi Higher Education Undergraduate Teaching Reform Project: Research and Practice in the Construction of the Three-dimensional Nine-Competency Curriculum System of “Aesthetic Education” in Ethnic Colleges Based on Educational Ecology (Project Number: 2024JGB382); 2024 Teaching Reform Project of Guangxi Minzu Normal University for Nationalities: Construction and Practice Research on Targeted Classroom Teaching Mode of Marxist Basic Principles (Project Number: JGYB202401); 2022 School-level Research Project of Guangxi Minzu Normal University for Nationalities: Targeted Research on Ideological and Political Theory Courses in Ethnic Normal Colleges Based on Big Data Mining (Project Number: 2022YB030).

## MHBM24040: THE PSYCHOLOGICAL IMPACT OF VOCATIONAL EDUCATION ON RURAL REVITALIZATION AND TALENT DEVELOPMENT: A CASE STUDY OF SHANGHAI INSTITUTE OF TOURISM SERVING FENGXIAN DISTRICT

Xiaoyun Li^a^, Haiwei Gao^a^, Zhiming Shao^a^

^a^ *Shanghai Institute of Tourism, Shanghai 201418, China*

**Objective:** This study aims to explore how vocational education contributes to rural revitalization and high-quality talent development, focusing on the psychological impacts on students’ motivation, identity, and engagement in rural construction. Using the Shanghai Institute of Tourism’s service to Fengxian District as a case study, the research investigates how vocational education strategies can enhance talent cultivation while fostering a deeper psychological connection between learners and rural communities.

**Methods:** A mixed-methods approach was adopted, combining qualitative and quantitative data collection. Interviews and focus groups were conducted with vocational educators, students, and rural community members to understand their perspectives on the integration of local cultural elements and shared resources in educational strategies. Additionally, a survey was administered to students to measure psychological factors such as motivation, self-efficacy, and aspirations related to rural development. Statistical analysis was performed to assess correlations between educational interventions and psychological outcomes.

**Results:** The findings reveal that vocational education strategies incorporating local cultural heritage and resource sharing significantly enhance students’ psychological engagement. Students reported increased motivation and a stronger sense of identity and belonging toward rural revitalization projects. Moreover, the inclusion of career counseling and culturally relevant curricula improved students’ self-efficacy and enthusiasm for participating in rural development. Vocational educators noted that aligning teaching goals with local needs also resulted in greater adaptability and success in fostering technical and psychological readiness among students.

**Conclusion:** Vocational education plays a critical role in rural revitalization by addressing both technical and psychological aspects of talent development. Incorporating local culture and psychological support into educational strategies fosters motivation and engagement, ensuring that students are not only skilled but also emotionally invested in rural construction. This study highlights the importance of integrating psychological theories into vocational education to maximize its impact on rural revitalization. Future efforts should focus on scaling these strategies and evaluating their long-term effectiveness in diverse rural settings.

**Acknowledgements**

Action Plan for Improving the quality and Training of Culture and Art Vocational Education and Tourism vocational Education of the Ministry of Culture and Tourism “Reconstruction Design and brand Operation of Shanghai Agricultural reclamation Catering Products under the background of integration of production and Education” (No. 70, 2021); Shanghai Educational Science Research Project “Collaborative Research of Vocational Education Professional Group and Industrial Group Based on Shanghai Style Catering Non-genetic Innovation “(C2023158)

## MHBM24041: RESEARCH ON INFLUENCING FACTORS OF SHORT VIDEO PLATFORM USERS’ TRAVEL MENTAL HEALTH

Zhifeng Huang^a^, Jianfu Wang^a^, Zhixuan Huang^b^, Haizhan Huang^a^, Miaoling Zhan^a^, Yuan Zhang^a^

^a^ *Quanzhou Normal University, Quanzhou 362000, Fujian, China,* ^b^ *Xiamen University, Xiamen 361102, Fujian, China.*

ZH and JW contributed equally to this study.

**Background:** As a leisure way full of fun and rich culture, travel has a far-reaching impact on people’s mental health and social development. Tourism is crucial to shaping residents’ mental health. In the era of rapid development of Internet+technology, tourism short video plays a very important role in the popularity of scenic spots and tourism destinations. Compared with traditional media, short video provides a more vivid and detailed psychological experience, which makes it easier for users to maintain good mental health, and allows users to have psychological resonance with the content promoted by businesses in a more intuitive and diverse way. Using short video platform to publicize the characteristics and content of tourism destinations has become an important communication tool today.

**Subjects and Methods:** This paper will analyze how attitudes consciously affect individual behavior and promote mental health, focusing on the formation process of attitudes based on cognitive information. According to the social and entertaining characteristics of mobile short video, this paper analyzes how the use of mobile short video affects the formation of tourists’ cognitive information and tourists’ behavioral intention in the new media environment by taking interactivity as the external influencing factor and entertaining as the internal influencing factor. In this paper, perceived usefulness, interactivity and entertainment are used as predictors of users’ travel intention, and the corresponding research model is established. The results show that perceived usefulness, perceived interactivity and perceived entertainment have a significant positive impact on tourism intention, which can better promote tourists’ mental health and relieve psychological pressure.

**Results:** This paper combines tourism intention with short video new media, and uses the questionnaire survey method to investigate and analyze. The results show that: first, perceived usefulness has a significant positive impact on the travel intention of users who watch short tourism videos, and the psychological pressure on tourists has been greatly reduced, which effectively promotes the mental health of tourists. Second, perceived interactivity has a significant positive impact on the travel intention of users who watch short tourism videos, and can effectively provide a channel for psychological stress relief. Finally, entertainment perception has a significant positive impact on the travel intention of short video users.

**Conclusions:** Tourism has a positive impact on mental health. Tourism can bring pleasant and happy experience and enhance people’s sense of happiness. Exploring new scenic spots, trying new food and experiencing new activities can bring happy emotions and satisfaction, and improve people’s quality of life. This study suggests that in order to encourage users to travel, tourist destinations should adjust their promotional strategies by continuously enhancing the content of travel videos and producing high-quality products. Through targeted interactive marketing strategies, interact with the target audience’s psychology, generate psychological resonance, and guide their psychological confirmation. Infuse entertainment elements to elevate the comfort level of tourists.

## MHBM24042: STUDY ON THE COMPREHENSIVE EFFECT OF CULTURAL INTELLECTUAL PROPERTY ON RESIDENTS’ MENTAL HEALTH IN THE BRAND BUILDING OF CHARACTERISTIC TOWN

Qian Zhao ^a^

^a^ *Changchun University of Architecture and Civil Engineering, Changchun 130000, China.*

**Background:** Characteristic towns are an important way for China’s new urbanization construction and rural revitalization, as well as a key platform for high-quality economic development. However, with the acceleration of the marketization process, as well as the lack of innovation and excessive imitation, the characteristic town faces the risk of homogenization in the development process. The lack of distinctive brands in small towns can lead to mental health problems for residents and businesses, such as increased stress, psychological anxiety and a lack of cultural identity. Therefore, branding strategy is particularly important in the construction of characteristic towns. Through the construction of unique cultural Intellectual Property, it can not only enhance the brand recognition and market competitiveness of the town, but also effectively promote mental health by meeting the psychological needs of residents, relieving anxiety and enhancing self-esteem.

**Subjects and Methods:** This study takes Wuzhen as a case to explore the path and strategy of characteristic town brand building based on cultural Intellectual Property. Through literature review, case analysis and mental health questionnaire survey, this paper studies the application of cultural celebrity Intellectual Property, drama Intellectual Property and digital Intellectual Property in town brand building. Specific methods include field investigation, interview and mental health assessment, focusing on the impact of cultural Intellectual Property brand construction on residents’ psychological needs, anxiety relief, self-esteem enhancement and overall mental health.

**Results:** The research results show that by mining and utilizing local cultural resources, characteristic towns successfully build brand Intellectual Property with unique cultural connotations, and significantly improve the mental health level of residents. Specifically, cultural celebrities such as MAO Dun’s former residence and Mu Xin Art Museum enhance residents’ cultural identity and psychological belonging through the combination of history and modernity; Through the holding of Wuzhen Drama Festival, Drama Intellectual Property not only improves the cultural atmosphere of the town, but also significantly relieves the anxiety of residents and enhances the sense of cultural participation and community belonging. Relying on the World Internet Conference, Digital Intellectual Property has promoted the development of the local digital economy, enhanced the town’s international visibility and technological identity, and improved the self-esteem and happiness of residents.

**Conclusions:** Brand building based on cultural Intellectual Property not only improves the brand image and market competitiveness of characteristic towns, but also has a significant comprehensive effect on residents’ mental health. Through the construction of unique cultural Intellectual Property, characteristic towns can meet the psychological needs of residents, relieve anxiety and stress, enhance self-esteem and cultural identity, and enhance psychological well-being and community belonging. These positive mental health effects are reflected in residents’ identification with the local culture, increased pride, and improved quality of life. The case of Wuzhen shows that Intellectual Property of cultural celebrities, drama Intellectual Property and digital Intellectual Property have significant effects in improving residents’ mental health. Other characteristic towns can learn from this experience, through the establishment of Intellectual Property innovation design laboratory, multi-channel communication and marketing, integration of cultural and tourism resources, enhance brand value, promote win-win economic and social benefits, and effectively enhance residents’ mental health and psychological identity.

## MHBM24043: RESEARCH ON THE INFLUENCE OF THE NEW GENERATION EMPLOYEES PERCEIVED ORGANIZATIONAL SUPPORT ON KNOWLEDGE SHARING

Zhongze Guo^a^, Hao Dong^a^, Mengmeng Xu^a^, Caiyi Xu^a^, Jiwen Chen^a^, Xiaoqi Xu^b^

^a^ *School of Business, Beijing Information Science and Technology University, Beijing, 102206, China,* ^b^ *School of Law and Humanities, China University of Mining and Technology, Beijing, 100083, China.*

**Background:** In the era of knowledge economy, knowledge sharing behavior has become the key factor to realize the goal and improve the competitiveness of the organization. How to effectively promote the knowledge sharing behavior of the employees is an important issue for the sustainable development of the organization. This study focuses on whether the new generation of employees are willing to share knowledge with colleagues and the mechanism of perceived organization support. Based on the self-determination theory, a mediating model was constructed to explore the mechanism of the influence of organizational support on knowledge sharing behavior, with task-contingent conscientiousness as the mediating variable and empowering leadership as the moderating variable.

**Subjects and Methods:** In order to reflect the actual situation of knowledge sharing behavior under the background of China, employees of two manufacturing and technology enterprises with dense distribution of new generation employees were selected as the survey objects. Twice online questionnaires were conducted four weeks apart. The first questionnaire included perceptual organization support scale, task-contingent conscientiousness scale and empowering leadership scale, the second questionnaire included knowledge sharing scale. A total of 338 new generation employees were collected as valid samples. The empirical analysis with SPSS27.0 and Mplus8.3 to test a mediated moderation model.

**Results:** The results found that: First, with the new generation of employees have personalized characteristics, such as the pursuit of self-value, preference for diversification, good at technology application, etc. Perceived organizational support has a positive impact on knowledge sharing. Second, Task-contingent conscientiousness has a positive influence on knowledge sharing. Third, task-contingent conscientiousness plays a mediating role between perceived organizational support and knowledge sharing. Finally, empowering leadership plays a moderating role in perceived organizational support and knowledge sharing, and the moderating effect of empowering leadership on perceived organizational support and knowledge sharing can be played through the mediating effect of task-contingent conscientiousness.

**Conclusions:** This study reveals the black box of knowledge sharing among the new generation of employees in China. Through theoretical and empirical research, enriches the application of self-determination theory in organizational management, explores the influence of task-contingent conscientiousness and empowering leadership on knowledge sharing behavior is explored, and proposes theoretical and practical implications for enterprises to effectively improve knowledge sharing through various ways.

**Acknowledgments**

This research was funded by the National Natural Science Foundation of China Project (Grant No. 72002017, 72002035, 72402232), Humanities and Social Science Fund of Ministry of Education (Grant No. 24YJC630063), The Project of Beijing Social Science Fund (Grant No. 18JDGLB032), and the Education Reform Project of Beijing Information Science and Technology University (Grant No. 2024JGSZ17).

## MHBM24044: A STUDY ON THE CONSTRUCTION OF YOUTH MOVIES ON THE PROMOTION OF ADOLESCENTS’ MENTAL HEALTH AWARENESS: A CASE STUDY OF THE MOVIE “BETTER DAYS”

Huan Zhou^a^, Xiaomin Xu^b^

^a^ *Nanchang Institute of Technology, Nanchang 330000, China,* ^b^ *Jiangxi Normal University, Nanchang 330000, China.*

**Background:** Since its release, the film “Better Days” has aroused widespread concern about school bullying and youth mental health issues. By showing the phenomenon of bullying in schools, the film reflects the mental health challenges teenagers face as they grow up. It is of great social and academic significance to study how the youth film “Better Days” promotes teenagers’ mental health awareness through the film narration and character shaping.

**Subjects and Methods:** By analyzing the narrative structure and characters of “Better Days”, this study explores the effect of “Better Days” on the promotion of adolescents’ mental health awareness. Using the “Actant Model” of structuralist semiotic analysis, this paper analyzes the main characters in the film and reveals the deep meaning of the film at the emotional and psychological levels. In the research process, the key scenes and dialogues in the film are selected, and the mental health information and educational significance conveyed by the film are interpreted through text analysis and semiotics.

**Results:** The study found that “Better Days” successfully presented the impact of school bullying on the mental health of teenagers through complex character Settings and insightful plots. The film’s protagonists, Chen Nian and Xiao Bei, represent the roles of victim and protector respectively, and through their interaction and growth, the audience can intuitively feel the psychological trauma and healing process. Chen Nian’s introversion and tenacity, and Xiao Bei’s protection and sacrifice, show the helplessness and resistance of teenagers in the face of campus violence. At the same time, through the in-depth portrayal of the villain Wei Lai, the film reveals the psychological motivation and family background behind the bully, and emphasizes the influence of social environment on the behavior of teenagers.

**Conclusions:** “Better Days” serves as an important education and warning by providing a realistic representation of bullying and the psychological state of young people. The film not only resonates with the audience at the visual and emotional level, but also raises the audience’s concern and understanding of adolescent mental health issues at a deep level. The research shows that youth movies, as a mass cultural medium, have the potential to play a positive role in raising the mental health awareness of adolescents. Future film creation should pay more attention to social reality and help teenagers better understand and cope with psychological challenges in the process of growing up through authentic and delicate narratives.

## MHBM24045: THE INFLUENCE OF WESTERN PERCUSSION MUSIC ON THE DEVELOPMENT OF CHINESE PERCUSSION MUSIC FROM THE PERSPECTIVE OF IMPROVING AUDIENCE PSYCHOLOGICAL NEEDS

Qi Lu ^a^

^a^ *Music School of Henan University, Kaifeng 475001, Henan, China.*

**Background:** This project introduces the historical background of Chinese percussion, as well as the changes and development of Chinese percussion after the entry of Western percussion into China and analyzes the influence of Western percussion on the development of Chinese percussion. This article reviews the development of Chinese percussion music from the early days of the People’s Republic of China to the present from the perspective of audience psychological needs and studies the room for improvement that Chinese percussion music can draw from Western percussion music. Through the musical phenomenon of Chinese percussion and Western percussion exchanges to explore its development direction.

**Subjects and Methods:** In the process of social and historical development, the Chinese people have been constantly exposed to the mature music system of Western symphony and Western chamber music, which has led to the increasing psychological needs of the Chinese people for the creation of excellent musical works of Chinese percussion instruments, and promoted the thinking of Chinese percussion practitioners and composers on their own development, and then absorbed the music styles, music themes and instruments of Western countries. This paper will examine the historical background of the development of Chinese music and explain its influence from the aspects of China and the Western education system and music performance style. The research sources include Chinese news reports, influential newspapers and periodicals published in China, and historical books. Through the above analysis, it explores how the arrival of Western percussion instruments has brought a new situation to Chinese percussion instruments, how some elements of the Chinese percussion tradition have gradually been integrated into the Western percussion tradition, and how to innovate and create excellent works to meet the psychological needs of the Chinese people for excellent Chinese percussion art.

**Results:** The assignment is divided into several chapters to examine separately, the establishment of Western and Chinese orchestras in the end of the 19th century and the beginning of the 20th century and the psychological needs of audiences for Chinese percussion works in different periods. In daily social interactions, people often respond psychologically to what is happening in their surrounding environment to effectively control and adapt to the environment. All social behaviors are interpreted to some extent, consciously or unconsciously. Each chapter examines different periods with their own social backgrounds, and audiences at different times have different psychological needs for music-making works. China has established Chinese Western orchestras and national orchestras based on the organization of Western orchestras. Some Western percussion instruments have also been used in China with the establishment of the orchestra. The reform of percussion teaching system after the founding of New China, Western percussion has also been established in the teaching system. Due to differences in language logic and historical backgrounds, Chinese percussion and Western percussion have formed different musical styles. After Western music entered China, Chinese composers also incorporated elements of Western music in their creations. And in terms of instrument types and performance, Western percussion instruments are also used by Chinese composers in their own creations, playing together with Chinese percussion instruments.

**Conclusions:** From the analysis of the above chapters, it can be foreseen that due to the Chinese people’s psychological needs for excellent musical works, while Chinese percussion music is influenced by Western percussion music, Chinese music workers are also actively learning and studying Western percussion music paradigms, and how this positive interaction shapes individual behavior and thinking progress. Therefore, Western percussion music has a profound impact on Chinese percussion music, and in the future both sides will learn from each other and make progress together.

## MHBM24046: CONSTRUCTION OF A THREE-ALL EDUCATION CURRICULUM SYSTEM FOR “CURRENT AFFAIRS AND POLICIES” COURSE IN NEW BUSINESS SCHOOLS FROM THE PERSPECTIVE OF POSITIVE PSYCHOLOGY

Yuan Ding^a,b^, Dazhuang Lu^c^, Wanyao Wu^c^

^a^ *School of Marxism, Guizhou University of Commerce, Guizhou 550014, China,* ^b^ *School of Marxism, Dalian University of Technology, Dalian 116023, China,* ^c^ *College of Cultrue, Art and Media, Guizhou University of Commerce, Guizhou 550014, China.*

**Background:** Traditional educational models in business schools often focus on theoretical instruction, neglecting the cultivation of practical skills and comprehensive qualities, resulting in graduates struggling to adapt to the complex business environment. Positive psychology emphasizes individuals’ positive qualities and potential, aligning well with the Three-all education concept. This paper explores integrating positive psychology with the Three-all education concept to construct a Three-all education curriculum system for the “Current Affairs and Policies” course in new business schools, aiming to enhance students’ ideological and political quality and comprehensive ability.

**Subjects and Methods:** This paper adopts a combination of theoretical exploration and practical application. By deeply analyzing positive psychology and the Three-all education concept, we propose course design and implementation strategies and explore these in actual teaching. Specific methods include modular course design, the combination of theory and practice, a multi-faceted Education by All Members strategy, a continuous support Education through All Processes strategy, and a comprehensive Education in All Dimensions strategy.

**Results:** Research shows that the curriculum system integrating positive psychology with the Three-all education concept effectively enhances students’ comprehensive qualities and ideological and political awareness. Specific outcomes include significantly improved student participation, learning motivation, and practical skills; teachers can better meet students’ personalized needs through diverse teaching methods and continuous feedback mechanisms; the course content is closely linked to the actual policy environment, enhancing its realism and practicality.

**Conclusions:** This paper constructs a Three-all education curriculum system for the “Current Affairs and Policies” course in new business schools, integrating positive psychology and the Three-all education concept, demonstrating significant theoretical and practical value. The study shows that this curriculum system effectively compensates for the deficiencies of traditional business education models, enhancing students’ comprehensive qualities and practical abilities. Future research should further deepen and optimize educational strategies, verify their effects through large-scale empirical studies, and strengthen teacher training and interdisciplinary cooperation to ensure the continuous improvement and promotion of the curriculum.

**Acknowledgements**

This work was supported by a project grant from Guizhou Province Higher Education Teaching Content and Curriculum System Reform Project (Grant No.2021218).

## MHBM24047: RESEARCH ON MULTIMODAL EDUCATION PATHWAYS FOR IDEOLOGICAL AND POLITICAL EDUCATION OF PROFESSIONAL COURSES IN UNIVERSITIES IN THE DIGITAL ERA: FROM THE PERSPECTIVE OF STUDENT MENTAL HEALTH

Dazhuang Lu^a^, Yuan Ding^b,c^, Jiejie Shang^a^

^a^ *College of Cultrue, Art and Media, Guizhou University of Commerce, Guizhou 550014, China,* ^b^ *School of Marxism, Guizhou University of Commerce, Guizhou 550014, China,* ^c^ *School of Marxism, Dalian University of Technology, Liaoning 116023, China.*

**Background:** In the digital era, the rapid development of digital technology has significantly transformed teaching methods, educational systems, and course content in higher education. The integration of digital technology provides crucial support for the implementation of Ideological and Political Education of Professional Courses (IPEPC). Simultaneously, the increasing prominence of student mental health issues directly affects their learning outcomes and quality of life. This study explores the multimodal education pathways for IPEPC from the perspective of student mental health.

**Subjects and Methods:** This study applies various mental health theories, including Maslow’s Hierarchy of Needs, Erikson’s Psychosocial Development Theory, and Positive Psychology Theory, to construct a multimodal IPEPC education model. An online questionnaire survey was conducted among 200 undergraduates, yielding 183 valid responses. Subsequently, semi-structured interviews were conducted with 20 students from different disciplines, and a comparative experiment was conducted with two classes, one adopting a traditional teaching model and the other employing a multimodal IPEPC teaching model.

**Results:** The survey results indicated that 87% of students believed that multimodal IPEPC education enhanced their interest and enthusiasm for learning, 82% stated that multimodal teaching helped them better understand and remember course content, and 78% felt that multimodal teaching helped alleviate learning pressure and improve their mental health. Interview analyses showed that most students found multimodal IPEPC education made the learning process more vivid and engaging, increased classroom interaction and participation, and provided emotional support and psychological assistance. Comparative experiment results showed that the class adopting the multimodal IPEPC teaching model had significantly higher scores in mental health levels (average score: 4.5 out of 5) and course content comprehension and memory tests (average score: 85 out of 100) compared to the control class (average scores: 3.8 and 76, respectively).

**Conclusions:** This study confirms that the multimodal IPEPC education model based on student mental health significantly improves learning outcomes and mental health levels. The findings support the promotion and application of this model in universities to enhance students’ psychological resilience, interest in learning, and overall educational effectiveness.

**Acknowledgements** This work was supported by a project grant from Guizhou Province Higher Education Teaching Content and Curriculum System Reform Project (Grant No.2021222).

## MHBM24048: INTEGRATION OF MENTAL HEALTH AND AIGC TECHNOLOGY IN 3D ANIMATION CURRICULUM REFORM: INNOVATIVE APPROACHES AND ENHANCEMENT OF TEACHING EFFECTIVENESS

Junli Wang^a^, Zhaohui Yuan^a^

^a^ *School of Fine Arts, Huanggang Normal University, Huanggang, China.*

**Background:** Mental health plays a vital role in education, especially in the challenging field of 3D animation, which requires a high level of technical skill, creativity, and the ability to manage complex projects under tight deadlines. Students face stress, creative blocks, anxiety, and strict deadlines, which can hinder both well-being and creativity. Addressing these challenges is key to helping students unlock their creative potential and focus better, leading to more innovative work and a rewarding educational experience. AIGC (Artificial Intelligence Generated Content) technology, which uses AI to automatically create media such as images, videos, and text, offers new ways to support students’ mental health. By encouraging emotional expression, reducing stress, and providing personalized creative prompts, AIGC tools help students overcome obstacles, build confidence, and boost their creative output. AIGC helps create a supportive and flexible learning environment, effectively reforming the 3D animation curriculum to better meet students’ needs while fostering their growth.

**Subjects and Methods:** This study examines the impact of incorporating AIGC technology into 3D animation education, focusing on its potential to enhance students’ mental well-being, foster richer emotional expression, and encourage more effective creative learning by providing them with advanced tools and interactive learning experiences. Qualitative and quantitative analyses were conducted to explore how AIGC-assisted activities affected students’ engagement, emotional dynamics, creative capabilities, and overall satisfaction throughout the course, aiming to determine the extent to which such technology can contribute to deeper understanding, better collaboration, and enhanced artistic output among students.

**Results:** The integration of AIGC technology in the 3D animation course led to significant improvements in students’ mental health, emotional expression, and creative engagement. The use of AIGC tools created a more supportive learning environment, reducing anxiety and enhancing psychological resilience. This positive atmosphere allowed students to express themselves more freely and explore creative ideas with greater confidence, leading to deeper engagement in creative tasks. The fusion of mental health support with AIGC technology not only enriched the students’ emotional well-being but also improved their overall creative output, demonstrating a promising approach to enhancing teaching effectiveness and fostering meaningful curriculum reform in 3D animation education.

**Conclusions:** The integration of AIGC technology into the 3D animation curriculum has the potential to enhance both the emotional and creative dimensions of students’ learning experiences by offering new tools and interactive learning opportunities. By promoting mental well-being, emotional expression, and creativity, AIGC serves as an innovative approach to curriculum reform, contributing to a more holistic and enriching educational process in creative disciplines, empowering students to engage deeply with their projects and explore diverse artistic perspectives in a supportive environment.

**Acknowledgments**

This research was supported by the [Ministry of Education’s Industry-University Cooperative Education Program] under Grant number [230817344807061]. Project title: Research on Teaching Reform of 3D Animation Design Course in Local Universities under the Background of Artificial Intelligence.

## MHBM24049: HEGEL’S DIALECTICAL LOGIC AND NEW UNDERSTANDING OF PHILOSOPHY BASED ON PSYCHOLOGICAL NEEDS

Yike Wang^a^, Yifan Wei^b^, Yiyou Hao^c^

^a^ *Guangxi MinZu University, Nanning 530006, China,* ^b^ *South China Normal University, Guangzhou 510006, China,* ^c^ *Faculty of Language of Shenzhen MSU-BIT University, Shenzhen 518000, China.*

**Background:** Hegel’s dialectics is the theoretical condensation of German classical philosophy and one of the three theoretical sources of Marxist philosophy. Through the universal connection and eternal development of things, dialectics is characterized by the “negation” of things, which is a consistent method of Hegel in his philosophical system, and such a concrete development path from “positive” to “negative” and then to “negative of negative” is critical and dialectical. However, the philosophical question of why dialectical logic can not start with “negation” has not been deeply investigated by human cognitive psychological needs. patients after thoracoscopic surgery, and to summarize the application experience.

**Subjects and Methods:** Hegel’s dialectics takes “logic” as its model and the self-dialectical development of consciousness as its historical law. This study combs Hegel’s dialectical logic, and carries out a bold deduction and logical argument of psychological needs to explore the differences between “negative” and “positive” as the beginning of dialectical logic, and what results will be brought by the two as the beginning of dialectical logic, that is, to explore whether “negative” can be used as the beginning of dialectical logic? And why must “certainty” be the beginning of dialectical logic? These questions concern the rationality of dialectics itself and the basic problems of dialectical logic.

**Results:** Through a series of derivations and arguments, only the thorough explanation of Hegel’s “negative” can not be used as the beginning of dialectical logic, and “positive” must become the beginning of dialectical logic, is the most powerful proof of dialectics. In human cognitive needs, rationality itself is contradictory, dialectical and inconsistent. The test of rational logic confirms that “positive” as the beginning of dialectical logic has certain realistic rationality and feasibility, “negative” as the beginning can not withstand the logic and time test.

**Conclusions:** Whether it is the law of unity of opposites, the law of mass tautometry, or the law of negation of negation, it all reveals the transcendence of Hegel’s negation dialectics. Hegel’s “negative dialectics” also reveals the contradictory movement of nature, the world and society, and lays a solid psychological cognitive foundation for putting forward dialectical materialism and historical materialism.

## MHBM24050: CONSTRUCTION OF ONLINE TEACHER-STUDENT COLLABORATIVE COMMUNITY MODEL IN CLINICAL MEDICINE PROFESSIONAL MASTERS CULTIVATION

Xiaomei Wang^a,#^, Yun Chen^a,#^, Ge Jing^a^, Mingzhe Li^a^, YanE Xiong^a^, Qiang Wang^a^, Xiaoliu Liu^a^

^a^ *Wuhan University of Science and Technology, Wuhan 430065, China.* #*XW and YC contributed equally to this study.*

**Background:** The relationship between supervisors and professional masters is the most basic educational relationship in the cultivation of graduate students in clinical medicine. The new era calls for a breakthrough in the traditional teacher-student relationship. In light of the actual needs of clinical medicine professional master cultivation and standardized residency training, this study explores the value of the online teacher-student collaborative community model in the cultivation of clinical medicine professional master.

**Subjects and Methods:** In this study, the concept of online teacher-student cooperative community is systematically proposed, and the construction path is established, including improving the construction of school information resources, building online learning space for teacher-student collaboration, establishing consensus on online goals, clarifying responsibilities, and ensuring that institutional frameworks meet the common needs of teachers and students.

**Results:** Practical research shows that the online teacher-student collaborative community model is highly recognized for its value in the cultivation of clinical medicine professional masters.

**Conclusion:** With the school providing a rich learning space, supervisors and professional masters building an online collaborative learning community model, and fully utilizing their respective responsibilities and initiatives, it is certain that clinical medicine professional masters with solid clinical skills and outstanding research abilities can be cultivated.

**Acknowledgements**

This work was supported by the key project of “2024 Higher Education Scientific Research Planning Project” of the Chinese Association of Higher Education (24YJ0303); Research Project on the Construction and Teaching of Online Courses for National Medical and Pharmaceutical Graduate Students (YXC2022-02-02); Wuhan University of Science and Technology Teaching Project (2023Z017); Wuhan University of Science and Technology Teaching Project (2023X047). 2024 Provincial Teaching Reform Research Project of Hubei Undergraduate Universities.

## MHBM24051: TRAINING OF PRACTICAL TEACHING ABILITY OF NORMAL COLLEGE STUDENTS IN HIGHER VOCATIONAL COLLEGES UNDER THE BACKGROUND OF HIGH-QUALITY DEVELOPMENT: AN EXPLORATION OF MENTAL HEALTH STRATEGIES

Baoyi Cheng ^a^

^a^ *Wuhan City Polytechnic, Wuhan, 430000, China.*

**Background:** In the context of the country’s macro strategy to promote high-quality economic development, the demand for talent is undergoing profound changes, shifting from a focus on quantity to an emphasis on quality. As a crucial part of the education system that directly serves socio-economic development, higher vocational education shoulders the responsibility of cultivating high-quality technical and skilled talents that meet the requirements of the new era. Primary education, as the cornerstone of basic education, directly influences the quality of future national talent cultivation through its teaching workforce. Therefore, under the background of high-quality development, enhancing the practical teaching abilities of normal students in higher vocational primary education programs is a key element for improving the quality of basic education and ensuring long-term development. Addressing the psychological well-being and mental health of these students is also critical, as it directly impacts their ability to learn, adapt, and perform under pressure, which are crucial for their future roles in educating the next generation.

**Subjects and Methods:** Through systematic research, it aims to provide theoretical support and practical guidance for improving the practical abilities and professional quality of these students. The research methodology includes a literature review to sort out related theories and practices on cultivating practical teaching abilities of normal students, and empirical research methods such as questionnaires and in-depth interviews to collect data and information from various aspects, including teachers and students of higher vocational primary education programs and employers. The study also evaluates the impact of mental health on learning outcomes and teaching effectiveness, recognizing that students’ psychological well-being is integral to their professional growth.

**Results:** By conducting an in-depth investigation and analysis of the current situation, this paper is expected to reveal the main problems in the cultivation of practical teaching abilities in higher vocational primary education programs, such as insufficient practical teaching resources, incomplete practical teaching systems, and the disconnection between practical teaching and industry needs. The study also addresses the mental health challenges faced by normal students and explores how mental well-being can be integrated into the teaching process to enhance learning and teaching effectiveness. Additionally, based on the actual situation of higher vocational education in China, it explores a series of targeted and highly operational strategies and suggestions, including strengthening school-school cooperation, building a systematic practical teaching system, optimizing curriculum settings and teaching content, and enhancing the construction of the teaching workforce.

**Conclusions:** In summary, this paper centers on the cultivation of practical teaching abilities of normal students in primary education programs in higher vocational colleges under the background of high-quality development, with a special emphasis on mental health and well-being. Through comprehensive and in-depth analysis and discussion, it aims to provide theoretical support and practical guidance for the reform of practical teaching in higher vocational education, especially in primary education programs. Implementing effective strategies and suggestions is expected to significantly enhance the practical abilities and professional quality of these students, thereby contributing to the continuous and healthy development of the national education cause by delivering more high-quality educational talents to basic education in China.

**Acknowledgments**

This paper is a phase result of the research project “Exploration of School-Enterprise Cooperation Model Innovation in Normal Programs under the Background of High-Quality Development in Vocational Education - Taking the Primary Education College of Wuhan City Polytechnic as an Example” (HBZJ2023784) funded by the 2023 Hubei Province China Vocational Education Society Research Project.

## MHBM24052: RESEARCH ON PICTURE BOOK BINDING DESIGN BASED ON CHILD PSYCHOLOGY

Yingli Yang^a^, Daiyao Chen^b^

^a^ *School of Fine Arts and Design, Guangdong University of Finance & Economics, Guangzhou 510320, Guangdong, China,* ^b^ *Shenzhen Polytechnic University, Shenzhen 518055, Guangdong, China.*

**Background:** Children’s picture books use a combination of pictures and text to capture children’s attention with rich visual elements, making them a beloved reading medium. As an essential bridge connecting content and readers, picture book binding design profoundly impacts children’s reading experience and mental development. This study aims to explore how to design picture book bindings based on children’s psychological development characteristics to better serve their cognitive needs and aesthetic growth.

**Subjects and Methods:** This study focuses on children aged 0-11 years old and employs literature research, case analysis, observation, and interviews to systematically examine the key characteristics of children’s cognitive, emotional, and social development at different ages. It summarizes the principles, visual elements, and presentation techniques that should be considered in the binding design of children’s picture books. The study emphasizes aspects such as fonts, colors, composition, materials, and binding forms, analyzing the psychological implications and educational significance conveyed by these design elements.

**Results:** The study finds that the binding design of children’s picture books should be based on the principles of children’s psychological development, aligning closely with their cognitive abilities, emotional experiences, and social awareness. As children grow older, their logical thinking and self-awareness gradually strengthen. The binding design can appropriately increase information complexity, focus on cultivating children’s curiosity and problem-solving skills, and guide them to develop a confident and optimistic attitude towards life. Additionally, children’s picture books must strictly adhere to safety principles, balance design interest with aesthetics, and maximize reading enjoyment through innovation in form and materials.

**Conclusions:** Creating high-quality picture books that align with children’s physical and mental characteristics, and have educational significance and artistic appeal, requires binding designers to deeply understand the principles of children’s psychological development. In design practice, visual information should be reasonably organized according to children’s cognitive levels, a warm and positive aesthetic atmosphere should be created based on their emotional needs, and interpersonal communication elements should be cleverly integrated in line with their social development needs.

**Acknowledgements**

This work was supported by Research Projects of Department of Education of Guangdong Province -2024CJPT002; the 2022 National Ministry of Education Industry-University Contract Collaborative Education Project (Grant No.220500247312131); the 2023 Guangdong Provincial Philosophy and Social Science Planning Project (Grant No.GD23XYS036).

## MHBM24053: A STUDY OF THE IMPACT OF TRANSFORMATIONAL LEADERSHIP ON JOB SATISFACTION OF COLLEGE PHYSICAL EDUCATION TEACHERS FROM A MENTAL HEALTH PERSPECTIVE

Bin Wen ^a^

^a^ *College of Modern Economics & Management, Jiangxi University of Finance and Economics, Nanchang, Jiangxi, 332020, China.*

**Background:** With the intensification of competitive pressure in modern society, teacher burnout has evolved into a global mental health problem, and a large number of studies have shown that teachers are a high-risk group for burnout. Burnout has become a prevalent mental health problem in the world, especially among helping professions, and teachers, as a special helping profession, should not be underestimated. This study delves into the psychological energy and state level of college physical education teachers from the research perspective to explore the role mechanism between the transformational leadership perceived by college physical education teachers and their job satisfaction, in order to stimulate the work energy of college physical education teachers, to change their work attitude, to promote the change of the headmaster’s leadership style, and to promote the school operation towards a more humane mode and higher value orientation.

**Subjects and Methods:** Through convenience sampling, scales were selected that were consistent with mental health surveys. It was finally decided to use the the Transformational Leadership Scale, the Job Satisfaction Scale, the Burnout Scale, and the Sense of Organizational Support Scale to conduct a questionnaire survey on a total of 500 physical education teachers in five universities in Jiangxi Province.

**Results:** Parsing research findings from a mental health perspective. The explained variance of perceived transformational leadership and burnout on job satisfaction was 76.0%, and the β value of perceived transformational leadership decreased from 0.771 (p<0.001) to 0.411 (p<0.001), which reached the statistically significant level, and the β value of burnout was -0.545 (p<0.001), and burnout played a partial mediating role. Perceived organizational support had a moderating role between transformational leadership and job satisfaction (B=-0.021, p<0.001). For college physical education teachers with low levels of perceived organizational support, job satisfaction increased extremely significantly as college physical education teachers perceived transformational leadership (B=0.524, T=18.255, p<0.001). For college physical education teachers with a high level of perceived organizational support, job satisfaction increased significantly (B=0.444, T=12.621, p<0.001) with the increase in perceived transformational leadership among college physical education teachers.

**Conclusions:** From a mental health perspective, college physical education teachers perceive that transformational leadership has a positive impact on job satisfaction; college physical education teachers perceived transformational leadership has a positive effect on burnout; college physical education teachers burnout mediates the relationship between transformational leadership and job satisfaction; college physical education teachers sense of organisational support moderates the relationship between transformational leadership and job satisfaction. By caring for and valuing college physical education teachers, taking the initiative to provide support, and establishing a relationship of mutual trust with college physical education teachers, transformational leaders provided instrumental and emotional support for college physical education teachers, improved college physical education teachers psychological health, and thus increased college physical education teachers job satisfaction.

## MHBM24054: ON THE COMMUNICATION PATH OF PSYCHOLOGICAL ELEMENTS IN MULAN LITERARY WORKS IN THE ENGLISH WORLD

Lijun Tang^a^, Hanqiu Yin^a^

^a^ *School of Foreign Languages, Guizhou University of Finance & Economics, Guiyang, China*

**Background:** In recent years, multicultural symbiosis has become the mainstream trend of cultural development in the world, but there emerge many difficulties in the process of overseas communication of Chinese traditional folk literature, which can lead to psychological distress and a sense of cultural isolation among individuals. This requires us to fully leverage the media role of translation and adaptation to expand the communication path of tradition culture. Mulan literary works are one of the models of diversified forms of overseas communication of Chinese traditional folk literature.

**Subjects and Methods:** With combination of literature research, comparison research and data statistics, this study studies the communication pattern of psychological elements in Mulan literary works to spread abroad.

**Results:** On the one hand, it adopts translation, novel adaptation, literary adaptation of children’s picture books and novels in the overseas communication and globalization of Mulan literary works. On the other hand, according to the target readers and audiences, the psychological elements of translation have been adapted on the aspects of the narrative mode, the rewriting strategy of detailed content, while the adult’s novels, children’s picture books and novels are presented with the adaptation of the filial-morality, heroine value, the female independence consciousness. On the whole, they have expanded into various types from the theme of Mulan with loyal and filial, strong and brave character to different new stories with the quality of individualism, heroism and so on. The adaptation of translation, literary and artistic works is proved to be an effective way for the oversea communication of Chinese traditional literary works.

**Conclusion:** These works are empowered with renewed vigor, upon which experience and reference can be drawn for the oversea translation, introduction and communication of other folk literature alike. Under the standards of openness and inclusiveness, the implementation of translation, literary and artistic adaptation from the cultural adaptation with psychological elements will contribute to the “going out” strategy of Chinese culture and promote the integration of Chinese traditional folk literature into the circle of world literature.

**Acknowledgements**

This work was supported by Guizhou Provincial Higher Education Reform Project on Teaching Content and Curriculum System “The integration path of ideological, political education into the curriculum of English-Chinese/Chinese-English Translation based on POA Theory” (No. 2023107); The Key Project of the Planning of Philosophy and Social Sciences in Guizhou Province “Research on the Intelligent Media Construction and Communication Path of Guizhou Revolutionary Memory in the Long March National Cultural Park” (21GZZD02).

## MHBM24055: THE IMPACT OF IDEOLOGICAL AND POLITICAL CURRICULUM REFORM FROM A PSYCHOLOGICAL PERSPECTIVE ON THE CONSTRUCTION OF TEACHER ETHICS AND CONDUCT

Si Li^a^, Wenyan Chen^a^

^a^ *Hainan College of Vocation and Technique, Haikou 570216, China.*

**Objective:** In the broad context of higher education in the new era, the ideological and political (I&P) curriculum stands as a pivotal platform for cultivating students’ moral character and fostering correct values. This curriculum is undergoing a profound and comprehensive reform that extends beyond mere reshaping of teaching content and methods. It delves deeply into the core of teacher ethics and professional conduct, integrating teachers’ personal growth with students’ mental health. The introduction of cognitive psychology brings new vitality and perspectives, emphasizing the fulfillment of psychological needs, regulation of anxiety, and promotion of personality development for both teachers and students, which are crucial for enhancing teaching quality and shaping students’ correct values.

**Subjects and Methods:** From a cognitive psychology perspective, teachers are not merely knowledge transmitters; they are also guides for students’ spiritual growth. Teachers’ psychological cognitive abilities, including a profound understanding of I&P curriculum content, a strong sense of identification with socialist core values, and effective techniques for managing personal emotions and student anxiety, directly determine the depth and breadth of educational practice. Through professional training in self-reflection and emotion regulation, teachers can continuously enhance their moral and professional ethics, while increasing their sensitivity to students’ psychological changes and adopting more scientific and humane response strategies to create a safe and healthy learning environment for students. Within this framework, teachers’ emotional management skills and emotional investment become key factors in enhancing teaching interaction and stimulating student engagement. Teachers who actively express positive emotions and effectively manage negative ones can inspire students’ enthusiasm for learning, promote emotional resonance between teachers and students, and thereby achieve the transmission and internalization of values through mutual learning and growth. Teachers need to learn to listen to students’ voices, understand their confusions and challenges, and respond to each unique individual with empathy, making I&P education a process of bidirectional interaction and shared growth rather than unilateral indoctrination.

**Results:** The design and implementation of the I&P curriculum must closely align with students’ psychological development stages and personality traits. By utilizing psychological research findings, teachers can more accurately identify students’ cognitive preferences and emotional regulation abilities, and accordingly adjust teaching strategies. Diverse teaching methods, such as case analysis, group discussions, and role-playing, are employed to cater to different learning needs and stimulate students’ learning potential. Especially when facing students’ common anxiety and personality development issues, teachers should apply psychological theories, such as cognitive-behavioral therapy and emotion-focused therapy, to design targeted interventions that help students establish positive coping strategies and promote their mental health and personality development.

**Conclusions:** The application of psychology not only innovates teaching methods in the I&P curriculum but also significantly optimizes teacher-student interaction modes, enhancing students’ learning motivation and sense of participation. This teaching mode based on psychological principles contributes to building harmonious teacher-student relationships and continuously improving teacher ethics. More importantly, it effectively promotes students’ comprehensive development, helps them establish correct worldviews, outlooks on life, and values, enhances their sense of social responsibility, and cultivates new-era talents with both solid professional knowledge and noble moral character. In this process, teachers are not only knowledge transmitters but also shapers of students’ souls, jointly writing a brilliant chapter in higher education in the new era.

**Acknowledgements**

This article is a phased achievement of the 2023 Hainan Provincial Philosophy and Social Sciences Planning Project (Ideological and Political Special Project) titled “Research on the Current Situation and Countermeasures of Teacher Ethics and Conduct Construction in Hainan Universities in the New Era” (No. hnsz2023-35).

## MHBM24056: RESEARCH ON EMOTION RECOGNITION OF PIANO MUSIC BASED ON MOBILENETV3-LSTM FOR EMOTIONAL REGULATION

Yaoyao Zhu^a^, Shimin Wang^b^, Ming Zhao^c^

^a^ *Jiangxi Vocational College of Mechanical & Electrical Technology, Nanchang, Jiangxi, 330013, China,* ^b^ *Jiangxi Normal University, Nanchang, Jiangxi, 330000, China,* ^c^ *Jiangxi Institute of Applied Science and Technology, Nanchang, Jiangxi, 330000, China.*

**Background:** With the rapid development of audio technology and artificial intelligence, the amount of music data continues to increase. The essence of music is the carrier of human emotional expression, how to achieve more accurate recognition of music emotions has become a focus of attention, and the music plays an important role in regulating emotions.

**Subjects and Methods:** In order to better extract the original features of music, this paper converts audio files into MIDI files. Firstly, the audios are preprocessed and the audio files are organized. Then, through music signal analysis techniques, the audio format is converted into a Mel spectrogram. During the audio conversion process, the processed Mel spectrogram is transcribed into MIDI format containing a series of note events using an automatic piano transcription neural network. Then, by calculating the multidimensional temporal music features of piano music, the melodies of different time nodes in piano music information are obtained. Based on MobileNetV3 and LSTM deep learning networks, a new model is designed, trained and tested for music emotions, achieving the effect of piano music emotion recognition for emotional regulation.

**Results:** Through experiments and analysis, the method proposed in the paper can effectively recognize emotions in piano music. The main purpose of the paper is to study and analyze piano music emotion recognition, accurately locate the emotions expressed in music, and provide assistance for regulating people’s emotions. 1) For 8 different emotions, 10 styles of music were collected for each emotion, and the differences between each type can also be felt. 2) In order to objectively identify music emotions, an automatic piano transcription neural network is used to convert audio into MIDI audio files and resample a large number of music samples. 3) A new model is designed based on MobileNetV3 and LSTM deep learning network, and the training and recognition of music emotions are completed.

**Conclusions:** The model constructed in the paper has shown good performance in emotion recognition, but there are still some shortcomings. The model proposed in the paper relies on audio to MIDI conversion and cannot be used universally for all audio files. In the future, the input audio file should be changed into a universal audio file to better regulate human emotions.

**Acknowledgments**

This work was supported by projects grant from Jiangxi education science and technology research (Grant No.GJJ2203304).

## MHBM24057: COST MANAGEMENT PRESSURE OF GREEN BUILDING IN NEW CAMPUS OF COLLEGES AND UNIVERSITIES

Xi Chen^a^, Benzheng Yang^a^, Xiaorui Chen^a^, Yongqi Liu^a^

^a^ *Construction Department, Renmin University of China (Tongzhou Campus), Beijing, China.*

**Background:** To do a good job in the new campus of colleges and universities green construction cost management, to reduce the effects of stress and maintain mental health, achieving the green construction cost management target, the research object is the green construction life-cycle period in the new campus of colleges and universities.

**Subjects and Methods:** Based on the AHP-FUZZY theory to multistage fuzzy comprehensive operations, by establishing a multi-level index system, the cost management evaluation model of the degree of psychological stress is established in the new campus of colleges and universities green construction. According to the evaluation standard of green construction, the index of land cost, building structure cost, water supply cost, drainage cost, HVAC cost, electrical cost and environmental protection cost is selected as the index of the standard layer.

**Results:** Based on the five standard layer indexes, 36 evaluation layer indexes are selected according to the pressure of cost management. By means of empirical analysis, the model’s operating program is explained. And the professional cost of building structure, water supply, drainage, HVAC, electrical are “excellent”.

**Conclusions:** The scientific and reasonable evaluation result proves the feasibility of the method. Facing challenges head-on is the best way to relieve psychological stress, this paper provides a new comprehensive evaluation method for green construction cost management of new campus in colleges and universities. In order to steadily improve the building energy utilization efficiency, gradually optimize the building energy use structure, effectively control the growth trend of building energy consumption and carbon emissions, and realize the high-quality development of green buildings on new campuses of colleges and universities, it is imperative to carry out reasonable cost management of green buildings on new campuses of colleges and universities.

## MHBM24058: THE IMPACT OF COGNITIVE INTERVENTION ON EMOTIONAL REGULATION IN DEAF AUDIENCES FOR ANIMATED FILMS

Linglan Zhou ^a^

^a^ *Zhejiang Vocational College of Special Education, Hangzhou, Zhejiang, China.*

**Background:** This study aims to explore how animated films can regulate the emotional responses of deaf audiences through cognitive intervention. Based on the Cognitive Theory of Emotion, this study uses experimental analysis to examine the role of different cognitive interventions in promoting emotional regulation among deaf audiences, aiming to identify effective intervention methods and provide theoretical support for optimizing the viewing experience of deaf audiences.

**Subjects and Methods:** A total of 60 deaf participants aged between 18 and 25 were recruited for this study. Participants were systematically divided into four groups according to the specific cognitive intervention implemented: Control Group (no intervention), Explanatory Subtitle Group, Background Information Group, and Combined Intervention Group (including both background information and explanatory subtitles). After watching an animated film, participants’ emotional response levels, subtitle effectiveness, and visual experience were evaluated using a Likert scale. The collected data were statistically analyzed to identify the impact of different interventions on the emotional regulation of participants.

**Results:** The results showed that the Combined Intervention Group had the highest emotional response score, recorded as 4.07 ± 0.37, significantly higher than the Control Group’s score of 2.00 ± 0.37. Additionally, the implementation of cognitive interventions positively impacted subtitle effectiveness, with the Combined Intervention Group’s subtitle effectiveness score at 3.80 ± 0.46. However, the differences in visual experience scores among the groups were relatively small, with the Combined Intervention Group’s basic visual experience score being 3.57 ± 0.18, indicating that cognitive interventions had no significant impact on visual experience.

**Conclusions:** Based on the Cognitive Theory of Emotion, this study experimentally explores the impact of cognitive intervention in animated films on emotional regulation among deaf audiences, revealing the key role of cognitive factors in enhancing the emotional experience of deaf audiences. The results indicate that combined interventions significantly improved the emotional responses of deaf audiences, particularly the intervention method that combines background information and explanatory subtitles, effectively promoting audience understanding of film content and emotional engagement, thus helping to release negative emotions and stimulate positive emotions for emotional regulation. Furthermore, explanatory subtitles not only improved subtitle effectiveness but also further enhanced emotional resonance by supplementing positive emotional cues. However, the impact of cognitive interventions on visual experience was limited, as deaf audiences’ visual experience remained relatively stable.

## MHBM24059: A REVIEW AND OUTLOOK ON THE INFLUENCING FACTORS OF HIGH-QUALITY DEVELOPMENT OF ENTERPRISE BASED ON THE PSYCHOSOCIAL ENVIRONMENT

Anan Wang^a^, Muyi Yang^b^

^a^ *Dali University, Dali, Yunnan, China, 671003,* ^b^ *Housing and Urban-Rural Development Bureau of Ma‘anshan, Ma‘anshan, Anhui, China, 243000.*

**Background:** Higher-quality development is crucial in academic and practical fields, notably for businesses. Although several studies have addressed this topic, there remains a lack of systematic analysis and integration in the area of influencing factors of high-quality development of enterprise. Therefore, the primary objectives of this review are to summarize existing research findings, compare different research content, identify key factors that influence high-quality development of enterprise, explore society psychological factors, and provide guidance for future research. Through these efforts, this paper hope to offer valuable references for both academia and practical applications.

**Subjects and Methods:** This review adopts the method of narrative review, aiming to synthesize the research results in the field of influencing factors of high-quality development of enterprise in recent years including psychology in recent years. Literature search is mainly conducted through Scopus databases, and the search terms include“ high-quality development of enterprise” to identify key factors that including management, finance, and green technology advances, as well as government incentives like tax reductions or subsidies, psychological and ESG and Influence mechanism.

**Results:** The significance, scale, and measurement of enterprise’s high-quality development have been extensively studied. Higher-quality development is crucial in academic and practical fields, notably for businesses. This research find: (1) Most research on ESG aspects has examined their impact on firm green innovation, efficiency and the impact of ESG on a company’s value can be both positive and negative. (2) Fiscal subsidies have a comprehensive influence on both R&D investment and the high-quality growth of enterprises. Nevertheless, their influence remains limited in the interaction, and the overall impact may not be readily apparent. (3) Strict environmental rules encourage businesses to invest in green technologies to adapt to policy changes, gain competitive advantages, and achieve high-quality growth. (4) Companies that invest in staff development can better foster a culture that encourages continuous learning and advancement, which eventually boosts creativity. (5) Social and psychological factors are the potential things that can influence the growth of a company.

**Conclusions:** Green innovation is directly related to the high-quality development of enterprises. ESG management plays an indirect role through green innovation, and the relationgship between corporate innovation culture, social and psychological factors and government subsidies is not clear and needs to be further confirmed.

## MHBM24060: THE SUBSTITUTION EFFECT OF SCIENTIFIC AND TECHNICAL PERSONNEL’S GOAL SELF-CONCORDANCE ON SUPERVISOR INNOVATION SUPPORT: IMPLICATIONS FOR INNOVATION BEHAVIOR AND PSYCHOLOGICAL WELL-BEING

Ting Wen^a^, Yu Wang^a^, Sainan Mao^a^

^a^ *Nanjing University of Finance & Economics, Nanjing, 210046, China.*

**Background:** Scientific and technical personnel are crucial drivers of organizational innovation, and understanding how to stimulate their innovation behavior remains a key area of research. This study introduces Goal Self-Concordance to explore its impact on the innovation behavior of scientific and technological personnel. It also examines its substitution effect on the influence of supervisor innovation support on innovation behavior, drawing on the motivational implications of Leadership Substitution Theory. Additionally, the mediating role of autonomous motivation is explored, while also highlighting the importance of psychological well-being in shaping these relationships. Psychological well-being is posited as a key factor influencing how individuals respond to innovation support and motivation in the workplace.

**Subjects and Methods:** This is an empirical study based on surveys conducted with scientific and technical personnel across multiple industries in China. The research begins by formulating hypotheses grounded in relevant theories and prior studies, followed by the development of a theoretical model. To validate the hypotheses, pre-tested questionnaires were used for data collection. Two rounds of surveys were conducted to assess the appropriateness of the instruments within the cultural context, ensure sample quality, and minimize non-response bias. Stratified random sampling was employed, with participants drawn from scientific and technological enterprises, research institutes, and universities. Data were collected from 16 cities over an eight-month period, starting in January 2024, resulting in 520 valid responses. The data were analyzed using SPSS 26 and AMOS 23 to verify the model and hypotheses, followed by a discussion of practical and social implications, research limitations, and directions for future studies. Psychological well-being was assessed as a moderating variable in the relationships among supervisor support, goal self-concordance, motivation, and innovation behavior.

**Results:** The findings show that supervisor innovation support positively influences employees’ autonomous motivation (internal and identified regulation) and innovation behavior. Goal self-concordance moderates these effects, such that a higher degree of goal self-concordance weakens the positive impact of supervisor innovation support on both motivation and innovation behavior. Importantly, psychological well-being plays a key role in this process: employees with higher well-being show a stronger response to supervisor innovation support and are more likely to engage in innovative behavior. Moreover, autonomous motivation mediates the relationship between supervisor innovation support, goal self-concordance, and innovation behavior, while psychological well-being enhances this mediation by fostering a more supportive psychological environment for innovation.

**Conclusions:** The study supports all the proposed hypotheses. Supervisor innovation support influences innovation behavior through autonomous motivation, with goal self-concordance acting as a moderator in this relationship. Furthermore, psychological well-being emerges as a significant factor that strengthens the relationship between innovation support, motivation, and innovation behavior. These results contribute to the theoretical understanding of the interaction between supervisor behavior, individual goal characteristics, and innovation motivation. By incorporating psychological well-being, this study provides a more holistic view of how organizational environments can enhance innovation, offering practical insights for management practices that aim to improve employee well-being, motivation, and innovative behavior.

**Acknowledgments**

National Social Science Fund General Program, “Research on Mechanisms, Pathways, and Strategies of Digital Technology Empowering the Upgrading of the Environmental Protection Industry” (Project No.: 23GBL006).

## MHBM24061: IMPACT OF SPIRITUAL MENTOR ON THE PSYCHOLOGICAL NEEDS ON INNOVATION BEHAVIOUR OF STUDENTS IN UNIVERSITIES: A MODERATED MEDIATION MODEL

Ting Wen^a^, Sainan Mao^a^, Na Li^b^

^a^ *Nanjing University of Finance & Economics, Nanjing, Jiangsu 210023, China,* ^b^ *Hohai University, Nanjing, Jiangsu 210013, China.*

**Background:** Innovation is critical to a country’s scientific and technological development, with universities playing a pivotal role in cultivating innovative talent, but how to foster or meet their psychological needs on innovation behaviour is crucial point. While spiritual leadership has been identified as a factor positively influencing to satisfy the psychological needs on innovation behavior, the exact mechanisms behind this relationship remain unexplored. According to self-determination theory (SDT), the psychological needs of competence, relatedness, and autonomy are essential for optimal functioning, including creativity. When these needs are met, individuals are more likely to internalize their goals, thereby increasing intrinsic motivation and, as a result, generating innovative behavior. This study investigates how spiritual mentorship affects the psychological needs on innovation behavior of university students, focusing on the mediating role of goal self-concordance - the psychological needs, and the moderating role of creative self-efficacy. Based on Self Determined Theory, the purpose of this article is to examine the relationship among spiritual mentor, psychological needs, innovation behavior, goal self-concordance, creative self-efficacy.

**Subjects and Methods:** A total of 324 mentor-student dyads from nine universities in China participated in the study. Data were collected through separate questionnaires for students and their mentors, which assessed variables such as spiritual mentorship, goal self-concordance, creative self-efficacy, and innovation behavior. The study used hierarchical regression analysis to test the direct and indirect relationships between these variables. Structural equation modeling was also employed to examine the fit of the proposed moderated mediation model, where spiritual mentorship influenced innovation behavior through the mediating effect of goal self-concordance - the psychological needs, moderated by creative self-efficacy.

**Results:** The analysis revealed a significant positive relationship between spiritual mentorship and students’ innovation behavior. This relationship was fully mediated by goal self-concordance - the psychological needs, indicating that students whose personal goals aligned with the innovation objectives fostered by their mentors were more likely to engage in innovative behavior. Additionally, creative self-efficacy moderated the strength of this mediation. Students with higher levels of creative self-efficacy exhibited a stronger connection between goal self-concordance and innovation behavior. These results suggest that both goal self-concordance and creative self-efficacy are critical for fostering innovation.

**Conclusions:** This study provides valuable insights into the role of spiritual mentorship which how to foster and satisfy the psychological needs on innovative behavior among university students. It highlights the importance of aligning students’ goals with goal self-congruence and enhancing their creative self-efficacy. Universities aiming to promote innovation should consider implementing spiritual mentorship programs that emphasize personal goal congruence and support students’ creative self-efficacy. This approach could foster a more innovative student body and has practical implications for both psychological educational policy and curriculum development.

**Acknowledgments**

This research was funded by Jiangsu Province Degree and Graduate Education Teaching Reform Project, Project No.: JGKT24_C030, Jiangsu Province Social Science Foundation, grant number 21GLB018, and Industry-University-Research Collaborative Education Project, Department of Higher Education, Ministry of Education of China, grant number 202101152023.

## MHBM24062: EFFECT OF MENTAL HEALTH ON THE EFFICIENCY OF RUTILE, SRTIO _3_ AND NB: SRTIO _3_ PREPARATION IN WORKERS

Yingnan Dong^a^, Jian Tang^a^, Wei Niu^a^, Changyong Yin^a^

^a^ *College of New Energy, Shenyang Institute of Engineering, Shenyang, 110000, China.*

**Background:** Based on the important influence of mental health on scientific research efficiency, this paper aims to explore how the mental health status of workers affects their work efficiency during the preparation of high purity powder. By focusing on and improving mental health, it can not only enhance the innovation ability of workers, but also promote the creativity of the team, thereby promoting the independent research and development and technological progress of key materials such as rutile, SrTiO_3_ and Nb: SrTiO_3_ single crystals.

**Subjects and Methods:** This study adopts the method of questionnaire survey and in-depth interview. The participants’ mental health status was assessed through questionnaires, which mainly covered the dimensions of emotional state, stress level and job satisfaction. 150 workers were randomly selected to conduct in-depth interviews to understand their psychological experience in actual work and its impact on work productivity. The relationship between mental health and scientific research efficiency was discussed by means of statistical analysis based on questionnaire data and interview results.

**Results:** The results of the study showed that there was a significant association between the mental health status of workers and the work efficiency of material preparation. After a questionnaire survey of 150 participants, it was found that about 72% of workers reported good mental health, and this group of workers increased their productivity by an average of 25%. In in-depth interviews, respondents generally reported that mental health problems can lead to decreased job satisfaction and reduced productivity by 20% to 30%. After implementing the mental health improvement strategy, the team’s overall performance improved by 15%.

**Conclusions:** The results show that workers’ mental health has a significant impact on the work efficiency of material preparation. Good mental health can improve job satisfaction and productivity, while mental problems can lead to lower productivity. According to the survey data, it is suggested that relevant units pay attention to the mental health of employees, regularly carry out mental health education and consultation, and improve the overall performance of the team and scientific research results.

**Acknowledgments**

Liaoning Provincial Department of Education Fund Project (LJKZ1097), Liaoning Provincial Department of Science and Technology Fund Project (2023030488-JH2/107).

## MHBM24063: INTEGRATION OF INDUSTRY AND EDUCATION: THE CASE OF THE SMART HEALTHCARE NURSING INDUSTRY COLLEGE AT HENAN UNIVERSITY OF TRADITIONAL CHINESE MEDICINE

Shan Cao^a^, Shan Gao^b^, Juan Qiao^b^, Jitong Hou^b^, Jian Chen^a^

^a^ *College of Medicine, Henan University of Traditional Chinese Medicine, Zhengzhou, Henan 450046, China,* ^b^ *School of Nursing, Henan University of Traditional Chinese Medicine, Zhengzhou, Henan 450046, China.*

**Objective:** This study explores the significance and pathways of establishing characteristic industry colleges under the background of industry-education integration. It aims to demonstrate how such initiatives can promote the sustainable development of the economy and society and the innovation and reform of higher education. The research focuses on the case of the Smart Healthcare Nursing Industry College at Henan University of Traditional Chinese Medicine to highlight the necessity of industry college development and its long-term benefits for industry growth, contributing to the construction of a healthy China.

**Subjects and Methods:** The subjects of this study are the stakeholders involved in establishing and operating the Smart Healthcare Nursing Industry College at Henan University of Traditional Chinese Medicine. The methods include a comprehensive analysis of the college’s development process, exploration, and practices. The study employs qualitative research methods such as case studies, interviews, and document analysis to understand the synergistic education model and its impact on industry collaboration and talent cultivation.

**Results:** The results section details the findings from the case study, which reveal the successful integration of industry and education through the Smart Healthcare Nursing Industry College. It showcases how the college has facilitated collaboration between government, enterprises, and educational institutions, leading to a mutually beneficial and coordinated approach to talent development. The study also identifies the long-term benefits for the healthcare industry, such as enhancing professional standards and promoting innovative practices in healthcare services.

**Conclusions:** The conclusions drawn from this study emphasize the critical role of industry colleges in transforming local undergraduate institutions in the new era. They conclude that establishing characteristic industry colleges is essential for achieving sustainable socio-economic development and higher education reform. The study also highlights the significant contributions of such colleges to the healthcare industry, suggesting that they are instrumental in advancing the goals of a Healthy China initiative. The findings advocate for replicating such models in other sectors to foster innovation and enhance the quality of higher education.

**Acknowledgments**

This work was supported by Henan Province Key Topic of Educational Teaching Plan for the Year 2025 (Grant No. 2025JKZD26); Henan Province Project for the Study and Practice of Higher Education Teaching Reform (Graduate Education Category) (Grant No. 2023SJGLX231Y); Henan University of Traditional Chinese Medicine Project for the Study and Practice of Graduate Education Teaching Reform (Grant No. YJSJ2023-002); Research topic on teaching and education of graduate students in Chinese medicine (Grant No. BYXC2024-02-0106).

## MHBM24064: THE DEVELOPMENT SPACE AND IMPROVEMENT STRATEGIES OF CHAOZHOU EDUCATION AND CULTURE FROM THE PERSPECTIVE OF POSITIVE PSYCHOLOGY AND MENTAL HEALTH

Jichong Tang^a^, Hehua Tang^b^, Mingyue Qu^c^, Xinxin Zhang^d^

^a^ *School of Literature, Jinan University, Guangzhou 510632, Guangdong, China,* ^b^ *School of Literature and Media, Dongguan University of Technology, Dongguan 523015, Guangdong, China,* ^c^ *Heze Home Economics College, Heze 274300, Shandong, China,* ^d^ *School of Criminal Investigation, Shandong Police College, Jinan 250200, Shangdong, China.*

JT and HT contributed equally to this work.

**Background:** Since the 21st century, Chaozhou education and culture have made significant breakthroughs in concepts and methods by continuously absorbing advanced experiences and excellent achievements. However, only by integrating positive psychology and mental health awareness can Chaozhou education truly flourish. As service institutions, education and training entities will face challenges such as unstable student enrollment, impacting their long-term viability. Specialization and precise positioning will be key trends for their growth. To this end, Chaozhou rural primary and secondary school extracurricular training institutions will focus on fostering mental health through the following strategies: 1) cultivating high-quality teaching resources (teachers, classrooms, equipment) that also prioritize mental well-being; 2) establishing a comprehensive teacher-student service model that includes mental health support; 3) employing modern educational technology methods that promote positive psychological outcomes; and 4) implementing efficient marketing strategies that highlight their commitment to mental health. Such off-campus education and training will not only cater to the economic and social development of rural areas in Chaozhou but also enhance local information technology education, thus enabling rural primary and secondary school students to access the same quality of teaching as their urban counterparts, thereby supporting their mental well-being.

**Subjects and Methods:** Psychologists like Martin Seligman emphasize that positive psychology, encompassing positive subjective experiences, personal traits, and institutional support, plays a crucial role in mental health education. The uneven development of basic education in Chaozhou, characterized by urban-rural disparities and variations in educational stages and outcomes, underscores the urgent need for improvement in mental health services. Extracurricular training institutions should recruit qualified teachers, adopt advanced teaching methodologies, and utilize modern educational tools to facilitate significant advancements in mental health and information technology in rural contexts. Furthermore, educators must adapt their teaching approaches to leverage the benefits of Internet education, effectively combining online and offline learning modalities to enhance students’ mental well-being.

**Results:** Positive psychology serves as a vital framework for educators to foster constructive educational philosophies that promote mental health. As societal awareness grows regarding the multifaceted role of education—not just in transmitting knowledge but also in nurturing psychological resilience and professional skills—families increasingly prioritize their children’s mental health and education. This shift drives investments in high-quality, differentiated educational offerings, particularly in economically disadvantaged rural areas of Chaozhou, where there is a pressing need to improve basic education standards and mental health resources.

**Conclusions:** Utilizing positive psychology and a focus on mental health to enhance Chaozhou’s educational and cultural landscape is of paramount importance. With rising market demand for educational services, competition within the training industry intensifies, fueled by low entry barriers and high demand. In this competitive environment, securing a share of the training market, cultivating strong brands that emphasize mental health, and enhancing visibility and reputation through mental well-being initiatives are essential strategies for success.

**Acknowledgments**

This project was supported by the Philosophy and Social Sciences Planning of Guangdong Province GD24CW12; China Postdoctoral Science Foundation under Grant Number 2024M751112; Philosophy and Social Sciences Planning of Guangdong Province GD23YZW05.

## MHBM24065: POSTURE ADJUSTMENTS AND THE PREVENTION OF REPETITIVE STRAIN INJURIES IN PIANO PERFORMANCE: HEALTH STRATEGIES FROM PRACTICE TO PERFORMANCE

Yusheng Gao^a^, Jing Zhang^a^

^a^ *School of Literature Arts and Media, Tongling University, Tongling 244000, Anhui, China.*

**Background:** Occupational health issues are a growing concern for professional pianists, with musculoskeletal disorders (MSDs) and repetitive strain injuries (RSIs) being particularly prevalent. These injuries are often the result of extended practice sessions and repetitive hand movements, compounded by poor posture and lack of ergonomic support. As these conditions can significantly impact the career longevity and performance quality of musicians, the need for effective preventive strategies is critical. This study explores the role of posture adjustments and ergonomic interventions in promoting occupational health and preventing MSDs in pianists.

**Subjects and Methods:** This qualitative study involved 30 participants, including 15 professional pianists and 15 advanced students, all with a minimum of five years of playing experience and a practice routine of at least 15 hours per week. Participants were selected based on their experience with posture-related musculoskeletal discomfort. Semi-structured interviews were conducted to gather detailed insights into their posture adjustment strategies, practice habits, and experiences with physical discomfort. A literature review of existing quantitative data on ergonomic interventions and occupational health in musicians was also conducted to support the findings.

**Results:** Participants reported significant improvements in occupational health after adopting posture adjustments, such as maintaining neutral wrist positions, proper spinal alignment, and using ergonomic equipment like adjustable benches and chairs with lumbar support. Those who incorporated preventive measures, such as warm-up exercises and regular breaks, also experienced reduced muscle fatigue and improved performance sustainability. However, pianists with smaller hand spans continued to experience strain during demanding pieces, indicating the need for more personalized ergonomic solutions. Some participants noted that posture adjustments alone were insufficient to completely prevent injuries, particularly during intensive practice sessions.

**Conclusions:** This study highlights the critical role of posture adjustments and ergonomic strategies in protecting the occupational health of pianists. Proper posture and the use of ergonomic supports were shown to reduce the risk of musculoskeletal injuries and improve long-term performance. However, these adjustments should be part of a broader health strategy that includes warm-up exercises, practice breaks, and individualized ergonomic interventions. Ensuring the occupational health of pianists requires a comprehensive approach, with attention to both preventive and corrective measures. Future research should explore more objective assessments of ergonomic interventions and consider adaptive piano designs for musicians with varying physical characteristics.

**Acknowledgments**

The author’s research was supported by the Provincial Quality Engineering Projects of Higher Education in Anhui Province (Project Numbers: 2022jxgl073 and 2023jcjx020).

## MHBM24066: RESEARCH ON THE STANDARD SYSTEM OF TERRITORIAL SPATIAL PLANNING ORIENTED TO RESIDENTS’ MENTAL HEALTH

Yifan Zheng^a^, Zhanpeng He^a,b^, Dong Meng^a^, Qibin Zhao^a^, Ling Deng^a^

^a^ *Chinese Academy of Natural Resources Economics, 101149 Beijing, China,* ^b^ *China University of Geosciences (Beijing), 100083 Beijing, China.*

**Background:** Territorial spatial planning directly or indirectly affects people’s living environment and mental health conditions through planning and management of land use, urban construction, and ecological and environmental protection. Reasonable territorial spatial planning can provide a comfortable, safe and convenient living environment and promote people’s mental health; while unreasonable planning may lead to environmental deterioration, traffic congestion, residential crowding and other problems, bringing psychological pressure and negative impacts to people; Analyzing the standard system of territorial spatial planning from the perspective of mental health is an important way to realize the coordinated development of territorial spatial planning and mental health. By rationally planning land use, urban construction and ecological environmental protection, and constructing a national land space planning standard system based on the perspective of mental health, we can provide residents with a comfortable, safe and convenient living environment, promote their mental health, and realize the sustainable development of national land space. In order to meet the demand for standardization of territorial spatial planning and to achieve dynamic updating of the standard system.

**Subjects and Methods:** This paper discusses the problems of the existing land spatial planning standard system, analyzes the coordination and supporting relationship between the land spatial planning standards, and puts forward a proposal to improve the standard system framework of “vertical layering and horizontal classification”, which focuses on the whole business chain of the preparation and management of land spatial planning and takes into account the standardization needs of informationization construction to optimize the classification of standards at each level of the standard system. The study proposes to improve the framework of “vertical layering and horizontal classification” of the standard system.

**Results:** On the basis of an in-depth analysis of the progress of standardization and revision included in the standard system, it is proposed to accelerate the formulation of standards in key areas, strengthen the coordination of standards, promote the supervision of the implementation of standards, actively attract the participation of many parties, optimize the standardization organization, and dynamically improve the standard system and other measures.

**Conclusions:** Promote the high-quality development of standardization of territorial spatial planning and give full play to the supporting and leading role of standardization in territorial spatial planning.

**Acknowledgements**

This work was supported by a project grant from Operational support for standardisation of natural resources (Grant No. 121102000000190027), National Key Research and Development Project (Grant No. 2022YFE0115200), Study on Natural Resources Support Policies for Coordinated Regional Development (Grant No. 102121201020000009010), System for the Preparation and Implementation of the 14th Five-Year Plan for Natural Resources (Grant No. 102121201020000009001).

## MHBM24067: PSYCHOLOGICAL DETERMINANTS OF UNIVERSITY STUDENTS’ ACCEPTANCE AND USE OF GENERATIVE AI TECHNOLOGIES

Ying Sun^a^, Jiyon Lee^b^

^a^ *Department of English, Xi’an International University, Xi’an, Shaanxi 710077, China,* ^b^ *Department of Teaching Profession, Sehan University, Yeongam-gun, Jeollanam-do 58447, Korea*

**Background:** Generative AI (GAI) technologies are revolutionizing educational environments, especially in higher education, where they offer tools to support creative tasks, writing assistance, and content generation. Understanding the psychological factors that influence university students’ adoption and continued use of these tools is critical. This study explores these factors using the Unified Theory of Acceptance and Use of Technology (UTAUT2) model, focusing on the psychological and behavioral determinants that drive both the behavioral intention and actual use of GAI apps among students.

**Subjects and Methods:** A total of 1,201 valid responses were collected from students at 10 universities in various regions of China. The survey employed the UTAUT2 model to assess key variables such as Performance Expectancy (PE), Effort Expectancy (EE), Social Influence (SI), Facilitating Conditions (FC), Hedonic Motivation (HM), Price Value (PV), and Habit (HT). Descriptive statistics, correlation analysis, and structural equation modeling (SEM) were used to analyze the relationships between these variables and their impact on students’ behavioral intention (BI) and use behavior (UB) of GAI apps.

**Results:** The analysis revealed that psychological factors such as PE, EE, SI, FC, HM, PV, and HT have significant positive effects on BI, while FC, HT, and BI directly affect UB. Furthermore, PE, EE, SI, HM, and PV exhibit direct positive effects on UB, emphasizing the utility of GAI apps in academic contexts. Six primary AI functions were identified, with creative tasks, writing assistance, and content generation showing the highest factor loadings, indicating their importance in supporting students’ academic activities.

**Conclusions:** This study validates the UTAUT2 model in the context of higher education, with the findings highlighting the significant role of psychological factors in shaping university students’ acceptance and use of GAI technologies. Positive perceptions of the technology’s usefulness and ease of use, combined with the influence of peers and intrinsic motivations, drive both the intention to adopt and the actual use of GAI apps. These insights can guide educators and developers in designing AI apps that are psychologically aligned with students’ academic needs, ultimately fostering greater adoption and enhancing learning outcomes in higher education.

## MHBM24068: EXPLORING THE REASONS FOR THE SPATIAL MISMATCH BETWEEN COASTAL INTANGIBLE CULTURAL HERITAGE AND TRADITIONAL VILLAGES: INTEGRATING INTANGIBLE CULTURAL HERITAGE TO HEAL MENTAL HEALTH STRATEGIES AND REGAIN CULTURAL IDENTITY

Feng Xue^a^, Weiheng Lin^a^, Qi Jia^a^

^a^ *Zhengzhou University of Light Industry, Zhengzhou 45000, China*

**Objective:** This paper aims to analyze the distribution pattern and influencing factors of intangible cultural heritage (intangible cultural heritage) and traditional villages in coastal areas of China. It provides targeted strategies for the protection and inheritance of intangible cultural heritage and traditional villages, enhances the sense of cultural identity, and explores the positive impact of intangible cultural heritage activities on the mental health of people in traditional village communities. The excavation of intangible cultural heritage has had a positive impact on the mental health of traditional village community members.

**Subjects and Methods:** This article aims to protect ethnic cultural memory and enhance the sense of belonging to ethnic culture. Developing intangible cultural heritage has a therapeutic effect on mental health. This paper uses GIS technology and applies methods such as the center of gravity model, location quotient, spatial mismatch index, geographic detector, and kernel density analysis to analyze and explain the spatial location information of ICH and traditional villages in coastal regions.

**Results:** The spatial distribution characteristics of ICH and traditional villages in coastal areas are “dense in the middle and sparse around”, and the provinces (cities) show spatial dislocation in the center of between the ICH and traditional villages, and form unique differences between different provinces (cities). Both intangible cultural heritage and traditional villages are influenced by the relationship between the natural and social environments. The dominant factors consist of multiple influencing elements, among which the GDP has the most significant impact on ICH, while rainfall has the most significant impact on traditional villages. Both factors can effectively promote the coordinated development of ICH and traditional villages, providing favorable evidence for fostering cultural resonance. This research enables a better understanding of the spatial distribution characteristics and influencing factors of ICH and traditional villages, helping to break the constraints of each influencing factor and promote rural cultural revitalization. The integration of intangible cultural heritage in traditional villages can enhance the sense of belonging and identity of ethnic culture. The intervention activities related to intangible cultural heritage have promoted the positive impact of improving the mental health of left behind personnel in villages and laid a solid foundation for inheriting and developing traditional Chinese culture.

**Conclusions:** This article, from a geographical perspective, explores the spatial distribution characteristics and influencing factors of ICH and traditional villages in China’s coastal regions. It aims to promote the integrated development of ICH and traditional villages and restore cultural identity. It reduces the sense of loneliness among the left behind people in traditional villages

**Acknowledgements**

This work was supported by a project grant from General Humanities and Social Sciences Project of Feng Xue Education Department (Grant NO.16602050007/0150)

## MHBM24069: CONTRADICTIONS AND RESILIENCE: EXPLORING FEMALE PERSONALITIES IN THE CANTERBURY TALES THROUGH THE LENS OF MENTAL HEALTH

Ran Jiang ^a^

^a^ *North China Electric Power University, Baoding, Hebei, China.*

**Background:** Feminist literary criticism is a critical approach that emerged in Europe and America in the late 1960s and early 1970s. It is the result of the Western feminist movement’s deepening cultural and literary fields, deeply rooted in the cultural and literary realms as a result of the Western feminist movement’s expansion. This movement not only sought to challenge and reshape societal structures but also addressed the psychological well-being and mental health of women.

**Subjects and Methods:** This article delves into the intricate portrayal of female personalities within Geoffrey Chaucer’s seminal work, “The Canterbury Tales,” with a particular emphasis on the mental health ramifications of the societal constraints imposed on women during the Middle Ages. Through the lens of feminist literary criticism and psychological analysis, the study meticulously dissects how Chaucer’s characters, notably Prudence and the Wife of Bath, encapsulate the profound psychological tension that exists between adhering to societal expectations and asserting one’s personal identity.

**Results:** The juxtaposition of these two characters reveals the complex interplay between medieval gender roles and the psychological conflicts they engendered, resonating with contemporary dialogues on female mental health. Chaucer’s nuanced depiction underscores the ongoing battle between societal constraints and the pursuit of self-actualization, highlighting the resilience required by women to assert their autonomy and the psychological toll that this endeavor can exact.

**Conclusions:** Through the Wife of Bath and Prudence, Chaucer offers a multifaceted exploration of the female psyche, illustrating the ways in which women navigated the intricate web of societal expectations and personal desires. Their stories serve as a testament to the enduring human struggle for identity and self-expression, a theme that continues to resonate in modern times. The psychological depth of these characters not only provides insight into the medieval world but also offers a mirror to contemporary challenges faced by women in their quest for mental well-being and self-determination. Thus, Chaucer’s work remains relevant, offering timeless commentary on the universal human experience and the eternal quest for personal freedom.

## MHBM24070: RESEARCH POTENTIAL EMPLOYEES IN CHINA AFFECTED BY CORPORATE SOCIAL RESPONSIBILITY INFORMATION: AN EMPIRICAL RESEARCH ON THE PSYCHOLOGICAL NEEDS OF UNIVERSITY GRADUATES

Wei Yang^a^, Yu Tao^b^

^a^ *Chongqing Business Vocational College, Chongqing 401331, China,* ^b^ *Chongqing University of Science and Technology, Chongqing 401331, China.*

**Background:** Meeting the psychological needs of university graduates is essential for fostering positive responses to corporate social responsibility (CSR) activities. Graduates’ perceptions of a company’s CSR efforts directly impact their psychological satisfaction, influencing their attitudes toward potential employers. High-quality CSR information can enhance graduates’ sense of social purpose, fairness, and security, which are vital psychological needs for individuals entering the workforce. Despite an increasing focus on CSR’s influence on corporate reputation and employee attraction, research remains limited on how CSR information specifically fulfills graduates’ psychological needs.

**Subjects and Methods:** This study is based on theories from psychology and organizational behavior, including stakeholder theory, organizational identification theory, and signaling theory. Together, these frameworks support the idea that clear, positive CSR information can satisfy graduates’ psychological needs for belonging, purpose, and self-worth, which subsequently influence their job intentions. Two surveys were developed, each presenting a unique CSR scenario to assess different psychological responses: one scenario depicted positive CSR information, while the other presented negative CSR information. By controlling for these variables, the study aimed to capture the psychological impact of CSR on graduates. The sample included senior students from universities in Chongqing, China, yielding 402 valid responses, which provided valuable insights into how CSR information affects their mental and emotional engagement with potential employers.

**Results:** Data were analyzed using SPSS, AMOS, and other statistical software to examine the psychological responses of graduates to CSR information. The independent sample T-test and regression analysis revealed significant relationships between CSR perception and psychological satisfaction, showing that CSR positively influences graduates’ feelings of trust, respect, and alignment with company values. These psychological effects were found to play a crucial role in shaping corporate reputation and job intentions, with graduates displaying more interest in companies that met their psychological needs through positive CSR engagement.

**Conclusions:** This study concludes that CSR information fulfilling graduates’ psychological needs has a positive impact on corporate reputation and job search intentions. Graduates’ desire for meaningful work, social responsibility, and a sense of alignment with employer values are all enhanced by CSR information that resonates with their psychological expectations. When CSR efforts satisfy these internal needs, companies are seen as more reputable and desirable employers. This underscores the importance for companies to communicate CSR authentically and transparently, as doing so not only enhances reputation but also fulfills the critical psychological needs of young job seekers.

**Acknowledgements**

This work was supported by the project grants from the Chongqing Business Vocational College project (Grant No. 2024XJKTYB30).

## MHBM24071: HOW AND WHEN INDIVIDUAL ARTIFICIAL INTELLIGENCE USAGE INFLUENCES PRESSURE APPRAISAL

Chen Yu^a^, Ziyan Peng^a^, Yuhan Shen^b^

^a^ *School of Business Administration, Zhejiang Gongshang University, Hangzhou 310012, China,* ^b^ *Division of Psychology and Language Sciences, University College London, London, WC1N 1PF, U.K.*

**Background:** Artificial intelligence does not always bring some benefits and no drawbacks to users in the workplace. Due to privacy exposure, fear of substitution and other reasons, artificial intelligence brings users some negative pressure appraisal. This study attempted to explore whether individual artificial intelligence usage related to self-objectification at workplace and what role do learning goal orientation play in the relationship between individual artificial intelligence usage and pressure appraisal at workplace.

**Subjects and Methods:** We surveyed a sample of 425 employees from 34 high-tech enterprise in Zhejiang province in China, who agreed to participate in the study, a wide range of position, regarding their perception of pressure appraisal and self-objectification. Regression analyses and correlate analyses were used to test the hypotheses.

**Results:** Individual artificial intelligence usage promoted positively and negatively employees’ pressure appraisal, that is artificial intelligence can lead to hate and love for users. At the same time, employees’ learning goal orientation also moderated the relationship between individual artificial intelligence usage and pressure appraisal.

**Conclusions:** Artificial intelligence brings users a combination of love and hate, because of the fear of privacy exposure, the crisis of being replaced, feeling inferior and self-abasement brought by users will have negative pressure appraisal, artificial intelligence as a tool, but also bring users positive pressure appraisal. When individuals have a high degree of learning goal orientation, the positive pressure assessment of artificial intelligence will dominate, and users can reduce the sense of pressure and anxiety. Therefore, organizations should teach employees who use artificial intelligence have high level of learning goal orientation.

**Acknowledgements**

This work was supported by two project grant from 1. Improving the quality and model optimization of new liberal arts graduate courses under the interdisciplinary integration of Zhejiang Gongshang University Graduate Education Reform Project in 2021 (No. YJG2021215); 2. Philosophy and social sciences General Project of Zhejiang Province: Research on the influence mechanism of compensation co-enrichment on innovation performance (No. 24NDJC136YB).

## MHBM24072: PSYCHOLOGICAL INFLUNENCING FACTORS AND IMPROVEMENT STRATEGIES FOR SPORTS PARTICIPATION IN CHINESE FEMALE COLLEGE STUDENTS

Yuhui Shao^a^, Yuzhen Wang^a^

^a^ *Shandong Police College, Jinan, Shandong 250200, China.*

**Background:** The physical health level of Chinese college students has significantly declined in recent years, and female college students have become the hardest hit area for physical health decline. One important reason for the decline in physical health of female college students is their insufficient participation in sports during their studies and daily lives, which is influenced by factors such as sports participation awareness and health cognition. Female college students are not only influenced by their awareness of sports participation and health cognition, but also by factors such as gender roles and self-efficacy. General Secretary Xi Jinping has clearly pointed out in his speech the importance of sports for national rejuvenation and national strength. The aim of this study is to explore in depth the impact mechanism of sports participation among Chinese female college students, clarify the psychological elements that affect their awareness of sports participation and health cognition, then adopt positive and effective intervention measures, to promote the formation of good health cognition and sports participation awareness among Chinese female college students, improve their physical fitness, and promote the construction of a healthy China.

**Subjects and Methods:** This paper comprehensively considers factors such as the distribution of men and women in universities and the distribution of humanities and engineering majors. Four representative provinces in different regions of China (Beijing Institute of Technology, Shandong Normal University, Henan Agricultural University, and Inner Mongolia Normal University) were selected to distribute 1400 survey questionnaires, and 1285 were collected. After removing invalid documents, there were 1218 valid questionnaires remaining. The questionnaire survey mainly includes general demographic characteristics, health cognition questionnaire, general self-efficacy scale, and sports participation awareness survey scale.

**Results:** The psychological factors that affect the participation of Chinese female college students in sports mainly include health cognition, awareness of sports participation, and self-efficacy. The Chinese education authorities should take measures to enhance the health awareness and sports participation consciousness of female college students, attach importance to the theoretical knowledge education of female college students in sports and health courses, increase support and encouragement for female college students’ sports participation, strengthen guidance on their self-efficacy, and pay attention to their gender role characteristics in sports participation to promote female college students’ sports participation.

**Conclusions:** The overall development of health awareness among Chinese female college students is unbalanced and insufficient; The awareness of sports participation among Chinese female college students is significantly insufficient; Health cognition has a positive predictive effect on the formation and development of female college students’ awareness of sports participation and self-efficacy; Good self-efficacy has the effect of regulating female college students’ health cognition and enhancing their awareness of sports participation; Female college students lack corresponding support and encouragement for their participation in sports.

## MHBM24073: ANALYSIS AND DEVELOPMENT SUGGESTIONS ON THE IMPACT OF COMPREHENSIVE TOURISM IMPLEMENTATION ON RESIDENTS’ MENTAL HEALTH IN WULONG, CHONGQING

Qinhou Zhu ^a^

^a^ *Chongqing Business Vocational College, Chongqing 401331, China.*

**Background:** As one of the important entities in comprehensive tourism development, the perception and mental health of local residents will also be affected by comprehensive tourism development. As an important indicator for evaluating the promotion of local development through comprehensive tourism, the mental health of residents also needs special attention. This article aims to analyze the development of comprehensive tourism in Wulong District, Chongqing, study the changes in the mental health level of local residents, and provide more suggestions for the sustainable development of comprehensive tourism and the continuous improvement of local residents’ mental health.

**Subjects and Methods:** The positive psychological perception of local residents helps to promote the development of tourism, but their negative perception will hinder the development of local tourism and affect their mental health level. This study takes the urban residents of Wulong city as the research object, and uses the questionnaire survey method to explore their perception of tourism economy, environment, social culture, satisfaction, tourism participation and other aspects, so as to comprehensively evaluate their mental health status. On this basis, further research on the similarities and differences of residents’ perceptions in different regions of Wulong city has practical guidance and reference significance for government decision-making, tourism enterprise management, residents and tourists, developers, etc. This can provide support for analyzing the impact of comprehensive tourism on Residents’ mental health.

**Results:** According to the statistical analysis of 1560 survey questionnaires, 1086 of them showed that local residents have a positive perception of the overall impact of comprehensive tourism development, believing that it can effectively promote local economic development while improving residents’ living standards and mental health; 352 respondents believe that the impact of comprehensive tourism development on themselves is not significant; Another 122 survey results suggest that the development of integrated tourism has affected the local environment, created a burden on the local area, and also affected the continuous improvement and enhancement of the mental health level of local residents.

**Conclusions:** The residents of Wulong generally have a supportive attitude towards the development of local tourism, which can effectively improve the living standards and mental health of local residents. However, due to the different stages of the life cycle of tourist destinations, there are also some residents who have a negative perception of the development of local comprehensive tourism. Wulong should formulate reasonable tourism support policies based on the development and tourism resources of different regions, and do a good job in coordinating government guidance, enterprise participation, and public support. This can not only further promote the development of the local tourism industry, but also continuously improve residents’ positive cognition and enhance their mental health level.

## MHBM24074: THE IMPACT OF UNDERGRADUATE CAREER PLANNING CURRICULUM REFORM ON STUDENTS’ MENTAL HEALTH IN SOUTH CHINA BASED ON DATA VISUALIZATION

Wen-Lung Chang ^a^

^a^ *School of Teacher Education, Guangzhou Huashang College, Guangzhou, Guangdong, 511300, China.*

**Background:** The latest round of career planning curriculum reforms in higher education institutions is profoundly transforming the educational functions of universities. It necessitates adherence to a student-centered approach, with a focus on problem orientation. This reform is critical to accelerating undergraduate educational reform, especially in the context of high-quality development. The integration of psychological well-being into career planning education is essential, as it directly impacts students’ mental health and happiness. This study aims to understand the current status of career planning curriculum reform for undergraduate education in South China, focusing on the psychological needs and happiness of students.

**Subjects and Methods:** The subjects were randomly selected from three higher education institutions in South China, with 430 students surveyed and 410 valid questionnaires collected. The study employs a mixed-method approach, utilizing data visualization analysis to evaluate the effectiveness of the curriculum reform. Psychological surveys were conducted to assess students’ perception of collaboration, cognitive workload, and stress levels. The study also incorporates the use of the Self Rating Anxiety Scale (SAS) and the Self Rating Depression Scale (SDS) to measure the impact of the curriculum reform on students’ mental health.

**Results:** The results indicate that the career planning education curriculum suffers from vague goal setting and outdated content, which can lead to increased anxiety and depression among students. The integration of data visualization and psychological support in the curriculum reform significantly improves stakeholder collaboration and reduces cognitive stress by providing a clear visualization of project goals and progress. The study reveals that poor model connectivity and lagging schedules negatively influence team morale and efficiency. Addressing these issues through early engagement, accurate positioning, and process control enhances both psychological and operational outcomes.

**Conclusions:** The study concludes that the integration of data visualization and psychological well-being into the career planning curriculum reform positively influences the psychological dynamics of undergraduate students. It improves collaboration, reduces stress, and enhances the overall mental health of students. Future Development Suggestions include establishing undergraduate or graduate programs related to career planning counseling; allocating special funds for career planning education; and providing systematic training related to career planning courses. These efforts aim to provide effective paths for improving the practical teaching abilities of normal students in primary education programs in higher vocational colleges, with a focus on fostering a mentally healthy and happy learning community.

## MHBM24075: EVALUATION OF THE EFFECTIVENESS OF A JOINT FAMILY-SCHOOL INTERVENTION ON STUDENTS’ PHYSICAL FITNESS AND PERFORMANCE

Shengmin Yuan^a^, Jian Wu^a^, Fangli Liu^a^, Xin Chen^b^

^a^ *National Institute of Education Sciences, Physical, and Art Education Research Center, Beijing, China,* ^b^ *Beijing Normal University, Beijing, China.*

**Background:** Both the school and family environments contribute to the development of healthy lifestyles in students. Our aim in this study was to explore the effectiveness of a joint family–school intervention on the physical fitness and performance of primary school students.

**Subjects and Methods:** The intervention consisted of a 12-week program of combined parental and teacher feedback on the physical activity levels of students in grades 1 through 6. The study group included 147 China’s students, 77 in the intervention group and 70 in the control group. Students in both groups completed the school-based physical activity program. The change from baseline was calculated for parameters of physical fitness (body mass index [BMI] and maximum heart rate) and exercise performance (rope skipping, sit-ups, sit and reach test), and compared between the two groups (independent-sample t-test) and within each group (paired-sample t-test). An ordinal logit regression model was used to evaluate the impact of parental monitoring and feedback on physical fitness and performance.

**Results:** Combining parental and teacher feedback yielded significant benefits, with a decrease in BMI and an increase in exercise performance for the experimental compared to the control group (*P*<0.01). This was confirmed by a significant improvement for all outcomes of the intervention program (*P*<0.01). On regression analysis, the frequency of parental feedback, student’s age, and the mother’s physical activity level were predictive of measured improvements. The regression coefficient for family sport participation was 0.064, with an odds ratio of 0.938. The regression coefficient of age was -0.373 and an odds ratio of 1.452. The third factor identified was mothers’ physical activity level, with a regression coefficient of 0.581 and odds ratio of 0.559.

**Conclusions:** Students’ lifestyle is influenced by both school and family environments. Specifically, parental monitoring and feedback can enhance the physical fitness and Psychological health performance of student in primary school. A 12-week intervention of parent feedback on their child’s physical activity at home, in combination with a school-based physical activity program, was effective in improving BMI and selected indices of physical performance (rope skipping, sit-ups, and sit and reach).

**Acknowledgments**

This project funded by 2023 Chinese General Education Project of National Social Science Fund “Research on the integration of intelligent management system to improve students’ physical health under the background of digital education in the new era” (Project No.: BLA230101).

## MHBM24076: A STUDY ON EMERGENCY RESPONSE CAPABILITY EVALUATION AND ENHANCEMENT STRATEGIES OF NANJING A COMMUNITY BASED ON RESIDENT SATISFACTION

Xiwen Jiang^a^, Shangxin Chang^b^, Shuangjun Hu^a^, Yuanyuan Wang^c^

^a^ *Finance Department, SINOPEC Yangzi Petrochemical Company Ltd, Nanjing, 210048, China,* ^b^ *School of finance and economics, Nanchang Institute of Technology, Nanchang, 330044, China,* ^c^ *School of Economics and Management, Jiangsu University of Science and Technology, Zhenjiang, China, 212100.*

**Background:** Community is the basic unit of a city, and its security situation is of great significance to the construction of a socialist harmonious society. Community emergency management aims at serving community residents, ensuring their life and property safety, maintaining social stability, and promoting the overall development of society. From the perspective of residents’ satisfaction, this paper takes A community as the research object.

**Subjects and Methods:** Based on the existing evaluation index system of community emergency response capability, this paper constructs a multi-dimensional evaluation system of community emergency response capability, including first-level indicators such as prevention and preparation, monitoring and early warning, disposal and rescue, recovery and reconstruction, as well as 18 specific second-level indicators. In the process of evaluation, the fuzzy evaluation method is adopted to investigate and analyze the satisfaction scores of residents on various aspects of community emergency management.

**Results:** the paper found that the residents of A community in Nanjing are satisfied with the evaluation of their own emergency response capacity, but there are still weak links in the community emergency management organization, emergency materials reserve, monitoring and early warning and psychological intervention capabilities, and some targeted countermeasures and suggestions are proposed.

**Conclusion:** (1) To strengthen the community’s prevention capacity, it is necessary to establish a complete emergency management leadership and equip adequate community staff. And improve the community emergency information management platform, expand the number of community residents using the platform. (2)The community should attach importance to the construction of emergency plans and exercises, and formulate scientific and operational emergency plans according to the community situation. Community residents are actively encouraged to participate in emergency preparedness drills to enhance their effectiveness. Publicity and popularization of emergency management activities, including posters, lectures and household publicity.

**Acknowledgements**

This study was supported by the Social Sciences of Jiangxi Province 14th Five-Year Plan (2023) Fund Project (23YJ15), and the 2024 university-level Higher Education Reform Project, Guangzhou Xinhua University (2024J063) and [2]“Macroeconomics”, 2024 first-class undergraduate course of Guangzhou Xinhua University, project (2024YLKC084).

## MHBM24077: VOICES OF CHINESE AMERICAN FEMALE: FAE MYENNE NG’S BONE FROM THE PERSPECTIVE OF FEMALE PERSONALITY

Fang Xiao ^a^

^a^ *Xi’an University of Architecture and Technology Huaqing College, Xi’an, Shaanxi 710000, China.*

**Background:** When it is said that Chinese American male is often classified in the lower class in the whole American society, Chinese American female is left to the more inferior space pitifully. Such situations bring about the distortion of patriarchal society and the pressure of female. In consequence, with respect to the minority female, invisibility and silence become the only living approach on account of their psychological split. The development of female personality seems still to be under way.

**Subjects and Methods:** Silence, a way of expressing women’s psychological needs and manifesting female personality, can be used as arms to struggle against various forms of exotic aggression. Silence can be said to result from the suppression of race, class, gender, culture and social customs on the various weak groups, particularly the female minorities. It is likely to be devastating and even destructive. But this sort of silence might also be their active and distinctive countermeasure to struggle against the dominating discourse as well as to construct a brand-new female personality. Silence is the voiceless resistance against social consciousness and norm represented by discourse. Moreover, not only silence but also silence breaking would be endowed with resistant power. Both of them reflect the generating process of the symbol of female personality, thus bringing about changes in female spirit. Once silence was broken up, the dominating destructive force would immediately die away, the long depressed human nature would get resuscitated, and therefore the balanced and harmonious relationship would return on the right track. Mah, in the fiction *Bone*, is rightly the animated carrier of the voice and voiceless. The mother in *Bone*, though a traditional Chinese female, strives to fight against the unfairness and prejudice on the female in the male-dominated society. As a representative of classic female, she has a clear and distinctive female personality, which enables her to find her own self-identity at the end.

**Results:** By the unique way of language silence and subjective silence, Mah tries all means to break silence to weaken the oppressing power in community. She succeeds in managing both of them in life with her intelligence and strength, thus realizing her subjective reconstruction and achieve her goal of happiness.

**Conclusions:** Based on the analysis of double-voiced discourse, personality as well as female personality, the article demonstrates that double-voiced discourse plays an important role in the development of female personality. Though Mah is oppressed by the male power and unfortunately swallows her pain in silence, she remains an unyielding feminist pioneer who successfully achieves her female integrity.

## MHBM24078: INNOVATIVE APPLICATION OF PARTICLE SWARM OPTIMIZATION GENETIC ALGORITHM BASED ON MENTAL HEALTH THEORY IN OFFSHORE WIND POWER SYSTEM

Tai Li^a,b^, Mengjie Li^b^, Sunan Sun^b^, Leqiu Wang^b^, Zhicheng Ji^c^

^a^ *WuXi University, Wuxi 214105, Jiangsu, China,* ^b^ *Jiangsu University of Science and Technology, Zhenjiang 212003, China,* ^c^ *Jiangnan University, Wuxi 212122, China.*

**Background:** To improve the adaptability and flexibility of mental health particle swarm optimization (PSO) algorithm, combined with the characteristics of genetic algorithm, a virtual inertia active disturbance rejection control (ADRC) system for offshore wind power is proposed, after incorporating the factors of mental health theory, the performance of the improvement algorithm is significant, compared with the traditional algorithm, the advantages are obvious, and the actual cases verify their effectiveness.

**Subjects and Methods:** Exploring the application of particle swarm optimization genetic algorithm in offshore wind power systems based on mental health theory has important theoretical and practical significance. Firstly, based on the system frequency dynamic response equation, an active disturbance rejection controller is designed according to the nonlinear feedback control law and extended state observer (ESO) to improve the anti-interference ability of the virtual inertia control system. Secondly, considering the characteristics of basic genetic algorithm (GA) parameters, the kernel fuzzy clustering algorithm is applied to the dual population division of genetic algorithm, and different adaptive crossover and mutation strategies are adopted for the dual populations obtained from clustering, effectively avoiding the phenomenon of “premature” in traditional genetic algorithms. At the same time, the performance of GA is verified by the performance test based on the test function, and the parameters of ADRC system are adjusted by PSOGA to achieve power sharing and adaptive noise elimination.

**Results:** The mental health theory emphasizes an individual’s ability to adapt and self-regulate when facing complex environments. Introducing it into particle swarm optimization genetic algorithm can provide a new optimization approach for the algorithm. For example, in the iterative process of algorithms, methods such as cognitive adjustment and emotional management in mental health theory can be used to improve the convergence speed and stability of the algorithm.

**Conclusions:** The proposed control method is effectively verified based on Matlab/Simulink. The results show that compared with the traditional algorithm, the proposed PSOGA- ADRC can adjust the frequency of the power system by effectively controlling the rotor speed, reduce the complexity of parameter setting, which is conducive to the safety and stability of the power grid in frequency events, not only improve the anti-interference ability, but also enhance the adaptability of the system.

**Acknowledgements**

This work was supported by project grant from the Start-up Fund for Introducing Talent of Wuxi University (no. 2023r027, no. 2022r025, no. 2021r045), Wuxi University Science and Technology Research and Development Fund (no. 2024h053).

## MHBM24079: EXPLORING THE ROLE OF TEACHER LEARNING COMMUNITY AS AN EMOTIONAL REGULATION IN FOSTERING QUALITY ENGLISH TEACHING IN WEST AFRICAN ESL CONTEXT: CASE OF MALI

Cheick Amadou Tidiane Ouattara^a^, Yige Zhu^a,b^

^a^ *Sanming University, Sanming, Fujian 365004, China,* ^b^ *Shanghai Normal University, Shanghai 200234, China.*

**Background:** While ESL education seems to be moving at a lightning speed, teacher continuing learning appears to be a response to assisting teachers in keeping up with this revolution. Teacher learning community is one noticeable PD paradigm aiming to capacitate the teachers. However, voices from Francophone Western Africa in PD debates are almost non-existent. Little scholarship has addressed the role of teacher learning community as an affective harness in fostering quality ESL teaching. This ethnography explored how teacher community of practice contributes to promoting effective ESL pedagogy in English-scarce areas like Mali.

**Subjects and Methods:** Using PLCs and teachers’ networks theories, we observed and interviewed (n=16) teachers of the Malian ESL teacher learning community in this qualitative ethnography. We aimed to describe, learn, and theorize the attitudes, experiences, emotions, and shared beliefs of the said teacher community, a culture-sharing group.

**Results:** Our findings unpack that the community of practice significantly contributes to reshaping ESL teachers’ pedagogical belief, experience, and attitude towards effective ESL teaching. It also appears to be a two-way reinforcer regulating the teachers’ disposition and emotions. The first set of reinforcement clears tediousness, demotivation, and other similar negative feelings among teachers. This brings about the second set of reinforcement that establishes serenity, confidence, and suchlike positive feelings among the teaching crew. The community of practice, therefore, equips teachers with emotional security stimulating their inclination towards best ESL pedagogy. It empowers the teachers to steer bountiful extracurricular activities, reinforcing the notion of ESL teachers as leaders, and widening teacher agency. It largely boosts English as it directs profuse activities surrounding this lingua franca in African ESL context. CLT, TBI and related paradigms appeared central to the effectiveness narrative of ESL teaching in the West African zone reinforcing the “communicative turn” in this area. For better practices, the community is in need for more upskilling in AI-powered ESL pedagogy that may be achieved through a strengthened continental and international networking.

**Conclusions:** This work is a tentative contribution to emphasizing the voice from French-speaking West Africa in debates on PD and ESL education research. It may spark more discussion opportunities and mold the way forward.

**Acknowledgements**

This work was supported by Sanming University High-Level Talent Research Startup Project (Grant No. 23YG13S, and a project grant from the Institute for Translation and Local Culture Dissemination of Sanming University (Grant No. KCP 2403).

## MHBM24080: AN EXAMINATION OF THE TRANSFORMATION OF MORAL EDUCATION FROM A PSYCHOLOGICAL PERSPECTIVE THROUGH THE INTEGRATION OF DIGITAL TECHNOLOGY

Wen Zhu^a^, Wenguang Xu^a^

^a^ *Bingtuan Spirit Research Center, Shihezi University, Shihezi 832000, China.*

**Background:** Digital technology is undergoing significant transformations, and the wave of digitalization increasingly influences society, profoundly affecting Digital technology is undergoing profound changes, and the tide of digitalization is increasingly affecting society and deeply impacting every aspect of people’s lives. Moral education is an education that helps people develop good moral qualities. In order to understand the impact of digitalization on moral education and deeply understand people’s psychological cognition, attitudes towards moral education digitalization, and observe and understand from a psychological perspective whether there are psychological changes, whether people psychologically accept, and whether they are willing to accept moral education digitalization, based on the survey, analyze potential problems and risks, and propose targeted measures to improve the digital transformation of moral education.

**Subjects and Methods:** Between February and June 2024, we conducted face-to-face interviews with 100 moral education practitioners from universities in northwest China, focusing on their psychological perception, acceptance, and acceptance level of moral education digital transformation, as well as their actual behavioral performance. We hope to gain a comprehensive understanding of their psychological perception, acceptance level, and actual behavioral performance towards moral education digital transformation, thereby laying a solid foundation for the digital transformation of moral education.

**Results:** The moral educator psychologically believes that digitalization has had a significant impact on moral education, affecting multiple aspects of moral education. They truly feel the profound changes brought about by digitalization in moral education, so they psychologically believe that the development of digitalization has driven the diversification and innovation of the means of moral education dissemination, and has brought about major changes in the ways of moral education. Moral education has broken through spatial and temporal limitations with the empowerment of digital technology and achieved instant information transmission and sharing. At the same time, digital technology can accurately analyze the individual characteristics and needs of moral education recipients and provide personalized push and intelligent matching of moral education resources, providing customized learning paths and content for recipients. It also improves the speed and ability of recipients to integrate information, enabling the rapid and precise dissemination and utilization of all kinds of high-quality moral education resources, breaking through spatial and temporal constraints, and achieving personalized and dynamic resource allocation. Besides, they psychologically identify and accept the digital transformation of moral education, and actively participate in it.

**Conclusions:** It has had a significant impact on the psychology of moral educators, who believe that digitalization has affected the entire society and has brought about important changes in people’s psychological cognition and psychological identification. Therefore, it is imperative to reform moral education digitally. However, there are also some risks that need to be taken seriously, and we should actively avoid them from a psychological and ideological perspective to continuously promote the digital transformation of moral education.

## MHBM24081: AN ANALYSIS OF THE TEMPORAL AND SPATIAL DISTRIBUTION CHARACTERISTICS AND INFLUENCING MECHANISMS OF CULTURAL RELICS IN ANYANG CITY FROM THE PERSPECTIVE OF MENTAL HEALTH

Chaofang Ming^a^, Yahan Zhang^a^, Xiaojing Ren^a^

^a^ *Anyang Normal University, Anyang, Henan, China.*

**Background:** The exploration of the temporal and spatial distribution characteristics and influence mechanisms of historical sites from the prism of mental health is conducive to revealing their latent potency in molding the regional spirit and impacting the psychological state of the populace. It aims to provide a profound understanding of the intricate relationship between historical relics and human psychology, thereby laying a theoretical and practical foundation for the preservation and psychological-cultural construction of cultural heritage in Anyang City.

**Subjects and Methods:** This research principally resorts to Excel software and GIS spatial analysis technology to conduct an all-round quantitative and visual scrutiny of the quantity, regional dispersion, and spatio-temporal distributional idiosyncrasies of 108 national and provincial cultural relics protection units in Anyang City from the standpoint of mental health. The root causes and determinants underlying these distributional traits are exhaustively and meticulously pondered over, considering the aspects of the physical geographical environment, the tiers of social and economic development, and political elements.

**Results:** The research findings manifest that the distribution of ancient city sites in Anyang is asymmetrical and congregated, preponderantly amassed in municipal districts, and in plain and hilly areas, they are mainly situated along rivers. These sites traverse a broad spectrum of historical epochs, and the number of sites dwindles with the escalation of the protection level. From a psychological vista, the copious and variegated historical sites in Anyang City serve as witnesses to the local history, facilitating the formation of regional cultural identity and psychological cohesion. They act as a catalyst for the crystallization of a shared cultural consciousness and a sense of unity among the local inhabitants.

**Conclusions:** The distribution characteristics of historical sites in Anyang are the consequence of the mutual interaction among a multiplicity of factors, encompassing the natural environment, social cultural psychology, and historical political and economic contexts. These sites, in return, exert a favorable influence on local residents’ cultural identity, sense of belonging, historical memory, and aesthetic experiences. In the forthcoming urban development and cultural construction, it is of utmost importance to accord significant precedence to the protection and utilization of cultural relics, to deeply excavate their psychological health value, and via rational planning and display, enable these relics to more effectively meet the psychological health requisites of residents, thus propelling social harmony and sustainable development in Anyang City.

## MHBM24082: AN ANALYSIS OF SALMAN RUSHDIE’S “MIDNIGHT’S CHILDREN”: EXPLORING THE INFLUENCE OF RACE, ENVIRONMENT AND TIME ON LITERATURE

Huarui Jiang ^a^

^a^ *Xi’an University of Architecture and Technology Huaqing College, Xi’an, Shaanxi 710000, China.*

**Background:** Salman Rushdie is highly influential in the international literary cicrle, and his representative work “Midnight’s Children” is considered as a classic in postcolonial literature. This novel has received widespread acclaim on the world literary stage, vividly portraying the complex historical changes and social landscape of India from the colonial period to independence through a unique perspective and narrative technique.

**Subjects and Methods:** This paper explores Salman Rushdie’s literary works through Taine’s three factors: race, environment, and time, focusing on the cultural richness of India as a foundational context for his narratives. “Midnight’s Children,” in particular, serves as a vivid national allegory of India’s history around the time of its independence, illustrating the profound influence of these factors on Rushdie’s writing. Environmental factors, such as India’s political instability and social change, profoundly affect the characters, triggering a spectrum of psychological needs from basic safety to self-actualization and cultural belonging. The narrative captures the characters’ anxiety and confusion, reflecting the social environment’s psychological impact. Racially, the novel delves into India’s diverse racial fabric and the psychological dynamics of racial identity and cross-cultural interactions. Characters grapple with the desire for acceptance and understanding, while also dealing with the psychological scars of racial conflict, showcasing the pivotal role of race in shaping the narrative. Time, the transition from colonialism to independence is a significant backdrop, influencing characters’ psychological shifts from traditional to modern value systems. This transition is marked by the characters’ struggles and ambiguities, which are crucial for understanding the broader impact of historical changes on Rushdie’s literary themes.

**Results:** In summary, “Midnight’s Children” is a testament to the intricate interplay of environment, race, and time in shaping Rushdie’s literary exploration of psychological and cultural identity, underscoring the depth and relevance of his work.

**Conclusions:** Salman Rushdie’s literary contributions, particularly in “Midnight’s Children,” offer profound insights into the Indian experience, illustrating how race, environment, and time shape literature. His innovative storytelling not only reflects the spirit of the times but also enriches the global literary landscape, making him a pivotal figure in contemporary literature.

**Acknowledgements**

This paper is supported by a project grant from Scientific Research Plan Projects of Shaanxi Provincial Department of Education. The project name is Research on the Influence of Environment, Race and Time on Salman Rushdie and *Midnight’s Children* (Grant No. 20JK0219).

## MHBM24083: A REVIEW ON RELATIONSHIP BETWEEN CAREER COMPETENCE, ARTIFICIAL INTELLIGENT, PSYCHOLOGICAL CAPITAL AND INNOVATION BEHAVIOR

Muyi Yang^a^, Anan Wang^b^

^a^ *Housing and Urban-Rural Development Bureau of Ma‘anshan, Ma‘anshan, Anhui, 243000, China,* ^b^ *Dali University, Dali, Yunnan, China.*

**Background:** The study examines the impact of career competence, psychological capital, and AI threat perception. The primary objectives of this review are to summarize existing research findings, compare different research content, identify key factors that influence innovation behavior, explore society psychological health factors, and provide guidance for future research. Through these efforts, this paper hope to offer valuable references for both academia and practical applications.

**Subjects and Methods:** This review adopts the method of narrative review, aiming to synthesize the research results in the field of influencing factors of innovation behavior in recent years including psychology in recent years. Literature search is mainly conducted through Scopus databases, and the search terms include“ innovation behavior” to identify key factors that including career competence, artificial intelligent and psychological capital.

**Results:** The significance, scale, and measurement of innovation behavior have been extensively studied. Innovation behavior is crucial in academic and practical fields, notably for businesses. This research find: (1) A positive correlation between career competence and job performance, in turn, has a significant impact on innovation behavior. (2) Psychological health capital positively influences job performance, which, in turn, has a significant positive impact on innovation behavior. (3) AI threat perception is characterized by positive, negative, and double perception.

**Conclusions:** In today’s fast-paced and highly competitive business environment, the role of psychological health capital and a positive attitude cannot be overstated. These intangible assets are crucial for fostering a work culture that not only enhances work efficiency but also encourages innovation behavior among employees. The paper in question underscores this significance, highlighting how a healthy psychological state and a proactive mindset can lead to breakthroughs and improvements in the workplace. The research presented in the paper explores the intricate relationship between an individual’s mental well-being and their capacity for innovation. It suggests that when employees feel psychologically secure and maintain a positive outlook, they are more likely to engage in creative thinking and take risks, which are essential components of innovation. This is because a positive attitude can reduce stress, increase job satisfaction, and promote a sense of autonomy and mastery, all of which are conducive to innovative behavior. The findings of this study offer practical recommendations for managers and leaders who are looking to foster a more innovative workforce. For instance, the paper might suggest implementing mental health programs, providing a supportive work environment, and encouraging a culture of open communication where new ideas are welcomed and rewarded. By doing so, managers can help to build psychological health capital within their teams, which in turn can lead to increased innovation.

## MHBM24084: RESEARCH ON THE IMPROVEMENT OF CULTURAL LITERACY AND MENTAL HEALTH OF RURAL LEFT-BEHIND WOMEN FROM THE PERSPECTIVE OF RURAL REVITALIZATION: A CASE STUDY OF H TOWN, JIAN COUNTY, JIANGXI PROVINCE

Junwen Wang^a^, Borong Wu^a^

^a^ *East China Jiaotong University, Nanchang, Jiangxi, China.*

**Objective:** Under the background of rural revitalization, this study explored the improvement of cultural literacy of rural left-behind women and its impact on rural development, and further studied the changes in mental health of left-behind women with improved cultural literacy. This paper aims to reveal the difficulties faced in improving the cultural literacy of rural left-behind women, analyze the influencing factors and potential mechanisms of improving the cultural literacy of rural left-behind women, and put forward practical countermeasures and suggestions to promote the improvement of their cultural literacy and mental health.

**Subjective and Methods:** Taking Jianxian H town in Jiangxi Province as an example, to study the impact of rural left-behind women’s cultural literacy improvement on their mental health improvement under the Rural Revitalization Strategy. First of all, this paper analyzes the current situation and main influencing factors of improving the cultural literacy and mental health of rural left-behind women by consulting relevant literature; Secondly, through the field investigation and the exploration of specific cases, this paper discusses the ways to improve the cultural literacy of rural left-behind women, and tries to put forward the reference experience and methods to improve the mental health status.

**Results:** Under the current situation of implementing the Rural Revitalization Strategy, the cultural literacy of rural left-behind women in H town has been improved to a certain extent, and their mental health has also shown positive changes. However, due to the influence of various factors, there are still many problems in the comprehensive improvement of the mental health of rural left-behind women in H town. In general, the cultural literacy of rural left-behind women in H town is relatively low and weak, and the lack of psychological counseling institutions is obvious. There are three main problems: Low enthusiasm for active learning, poor atmosphere for receiving education, and difficulties in implementing vocational skills training. These problems need to be further solved and improved to improve the mental health literacy of rural left-behind women.

**Conclusion:** This study evaluated the current situation and main strategies of improving the cultural literacy and mental health of rural left-behind women in H Town, Jianxian County, Jiangxi Province. From the perspective of endogenous demand, this paper puts forward the following three suggestions from the three dimensions of ideology, government leadership and training system: first, enhance the awareness of knowledge acquisition of left-behind women and change traditional ideas; Second, strengthen the leading role of the government and create a positive social atmosphere; The third is to improve the training and mental health consultation system to ensure the smooth channels of vocational training.

**Acknowledgments**

This paper is a phased result of the 2022 Jiangxi Province Social Science Planning Project “Research on the Improvement of Public Service Capacity of Township Governments in Counties Lifted Out of Poverty under the Rural Revitalization Strategy” (Project No.: 22GL51D).

## MHBM24085: RETURNING TO RATIONALITY: CONSTRUCTING A SOCIAL ANXIETY ALLEVIATION PATH FOR CULTIVATING E-COMMERCE LIVE STREAMING TALENTS

Huijin Li^a,b^, Wenjuan Fan^a,b^

^a^ *Yangzhou Polytechnic Institute, Yangzhou, 225000, China,* ^b^ *School of Education Science, Yangzhou University, Yangzhou, 225000, China.*

**Background:** E-commerce live streaming has entered the second half, and the industry has put forward higher requirements for the professional and standardized talent cultivation of e-commerce live streaming. However, the current wild growth of e-commerce live streaming has led to a significant increase in various risk factors, such as ruin the top network anchors public image, making quick money from traffic, and lack of respect for e-commerce live streaming talent cultivation... This can easily trigger social prejudice and distrust, leading to an expanding trend of anxiety towards e-commerce live streaming among members of society. It is worth pondering how to scientifically cultivate e-commerce live streaming talents to alleviate social anxiety.

**Subjects and Methods:** This study adheres to the concept of “people-centered” and comprehensively analyzes the live streaming e-commerce ecosystem industry chain from the perspective of school enterprise cooperation and integration of science and education. Taking the talent training of anchor positions as an example, it further sorts out how e-commerce live streaming talent training can use data to empower manufacturing enterprises to transform and upgrade their products, create new consumer demands, construct new consumption scenarios, enhance consumer experience, etc. It attempts to reconstruct the relationship between e-commerce live streaming talent training and society around the industry chain, and alleviate social anxiety.

**Results:** The rampant growth of e-commerce live streaming in a “rush for quick success and instant benefits” and the imbalance between talent demand and supply in the live streaming e-commerce industry are the real factors that breed and amplify social anxiety. Social anxiety is a situation that is difficult to completely avoid and a psychological cost that the live streaming e-commerce industry needs to bear after reaching a certain stage of development.Clarified the talent cultivation positioning of e-commerce live streaming based on the industry chain of e-commerce live streaming industry, combined with career development needs and talent growth dimensions.Closely connecting with the entire development chain of the live streaming e-commerce industry, a modular curriculum system for e-commerce live streaming has been constructed with vertical key position capabilities and horizontal comprehensive business literacy as the logical starting point.Innovatively propose to integrate the cultivation of “craftsmanship spirit” and labor education into the training of e-commerce live streaming talents.Guide the cultivation of e-commerce live streaming talents to break out of the vicious circle of “traffic”, and empower the sustainable, orderly, and healthy development of new digital economy formats with high-quality talent cultivation.

**Conclusions:** This study proposes a solution to the high-quality cultivation of e-commerce live streaming talents, constructs a new model for e-commerce talent cultivation in the era of digital media new economy, guides the public to build a rational and peaceful mentality towards the cultivation of e-commerce live streaming talents, and helps promote high-quality development.

**Acknowledgements**

This work was supported by the Special Project of the Jiangsu Higher Education Association Counselor Work Research Association in 2023, No. 23FYHYB038.

## MHBM24086: THE DESIGN AND PRACTICE OF SCHOOL-ENTERPRISE COOPERATIVE COLLABORATIVE EDUCATION TO PROMOTE THE MENTAL HEALTH OF STUDENTS IN PROVINCIAL UNIVERSITIES IN CHINA

Tao Men^a^, Hongmei Tang^b^, Leyi Men^c^, Peng Cao^a^, Yuting Ma^a^

^a^ *Leshan Normal University, LeShan, 614000, China,* ^b^ *Sichuan University of Media and Communications, Chengdu, 611745, China,* ^c^ *ShanghaiTech University, Shanghai, 201210, China.*

**Background:** In the current social context, the mental health issues of college students present a complex and changing trend. School-enterprise cooperation has a positive and multi-dimensional impact on the mental health of college students, such as providing practical opportunities, enhancing social support, and improving psychological adjustment abilities. However, there are also problems in the cooperation model. Under the trend of building a digital China in the whole society, there is a shortage of digital talents in China. Colleges and universities face many challenges in cultivating digital technology talents. It is difficult to cultivate high-quality digital technology talents only relying on colleges and universities. School-enterprise cooperation and collaborative education is a very good way of talent cultivation. Therefore, Leshan Normal University and Lifang Group have carried out long-term cooperation and explored an effective talent cultivation path.

**Subjects and Methods:** The school and enterprise have clearly defined the cooperation objective as cultivating high-quality applied talents and promoting teaching reform. The cooperation content covers various aspects such as the formulation of talent cultivation programs, curriculum construction, practical teaching, teaching staff construction, employment and entrepreneurship, and mental health education. They have established a cooperation mechanism including a cooperation leading group, signing agreements, setting up a management institution, and establishing a funding guarantee mechanism, and implemented it in the preparation, implementation, and summary stages. At the same time, measures such as strengthening organizational leadership, communication and coordination, funding guarantee, and assessment and evaluation have been taken.

**Results:** School-enterprise cooperation runs through the entire process of university talent cultivation. A dual-tutor system has been constructed, and a variety of practical activities have been carried out. Provincial-level high-level practice bases have been built, and the organizational management system has been improved. The school and enterprise have jointly reformed the practical teaching system, jointly formulated the training program, carried out practical projects, and established a quality monitoring system. They have jointly strengthened the construction of the teaching staff and improved the practical and teaching abilities of teachers. They have promoted the construction of digital teaching resources. Many courses have won awards and been selected as first-class courses, and the resources have been widely used. A large number of achievements have been made in teaching reform, team building, curriculum resource construction, discipline competitions, and innovation and entrepreneurship.

**Conclusions:** The success of the school-enterprise cooperation between Leshan Normal University and Lifang Group stems from close relationships, clear objectives, resource investment, teaching staff construction, and attention to student psychological counseling. This enables the school to cultivate talents in line with industry needs and improve students’ practical and innovative abilities. In the next step, both sides should increase investment in practice bases, strengthen publicity and guidance for students, deepen and expand cooperation, and strengthen exchanges and cooperation to improve the construction level of practice bases and the quality of talent cultivation, provide a better development platform for students, help them become professional talents, and continue to strengthen cooperation in the future to cultivate more digital media technology talents.

**Acknowledgments**

This work was supported by a project grant from the Innovative Experimental Project of Provincial Ordinary Undergraduate Universities in Sichuan Province in 2024, the Undergraduate Major Construction Progressive Cultivation Project of Leshan Normal University (Grant No. ZYDJ-2023-29), the 2021 Sichuan University Student Off Campus Practical Education Base Construction Project, the 2022 Leshan Normal University “Integrated Development” Teaching Reform Project (Grant No. RHJG-2022-07), the 2022 Leshan Normal University Teaching Team Construction Project (Grant No.JXTD-2022-03).

## MHBM24087: INNOVATIVE APPROACHES TO COURSE REFORM IN ENTREPRENEURSHIP MANAGEMENT: EMPHASIZING ON PSYCHOLOGICAL HEALTH AND STRESS RESILIENCE

You Ren ^a^

^a^ *Jianghan University, Wuhan, Hubei, 430056, China.*

**Background:** This research explored the significance of entrepreneurship education in economic development and cultivation of innovative talent, particularly focusing on how to reform traditional teaching models to better meet societal demands for the psychological health and resilience of entrepreneurs in the context of globalization and rapid technological advancement. The study proposed an innovative H3M (Hyperspace Multi-dimensional Maneuvering Module) teaching model to enhance the psychological resilience and practical abilities of students, thereby promoting educational quality and socio-economic development.

**Subjects and Methods:** The research adopted entrepreneurship management courses from multiple higher education institutions as subjects and systematically analyzed the goals, implementation methods, and specific contents of curriculum reform. Data related to the psychological health, anxiety management, and emotional intelligence of students were collected through surveys and interviews to evaluate the effectiveness of current courses in meeting the psychological needs of students and examine the efficiency of H3M teaching model in practical applications.

**Results:** Research findings indicated that while current entrepreneurship education courses focused on professional knowledge and skills, they often overlooked the importance of the psychological health of students. By integrating H3M teaching model, courses could more effectively promote the psychological well-being and resilience of students, enhancing their practical skills and entrepreneurial confidence. Student feedbacks suggested that the integration of psychological health support and anxiety management strategies had made them more resilient and adaptable in the face of entrepreneurial challenges.

**Conclusions:** Emphasizing on the cultivation of psychological health in entrepreneurship education is crucial for improving entrepreneurial success rates of students. This research recommended that higher education institutions combine psychological health education with innovation and entrepreneurship courses, applying H3M teaching model to create a supportive learning environment that helps students enhance their emotional intelligence and resilience. This approach will not only facilitate their personal development but also contribute to the overall progress of economy in the society, aligning with the current demand for innovative talents.

**Acknowledgments**

This research was supported by Department of Higher Education, Ministry of Education, industry-university cooperative education project “Innovation and entrepreneurship curriculum system construction project”, 201801198040.

## MHBM24088: EFFICIENCY EVALUATION OF CHINA’S PILOT CARBON MARKETS WITH UNDESIRABLE OUTPUTS UNDER CLIMATE CONCERNS

Zhipeng Yan^a^, Lu Jiao^a^, Shan Wang^b^, Ying Wang^c^

^a^ *School of Economics and Management, North University of China, Taiyuan, Shanxi, China,* ^b^ *Beijing Aerospace Institute for Metrology and Measurement Technology, Beijing, China,* ^c^ *School of Finance, Nanjing University of Finance and Economics, Nanjing, Jiangsu, China.*

**Objectives:** The increasingly severe climate problem has caused widespread anxiety among people. Carbon trading market is an important platform to fight against climate change, but undesirable outputs are associated with China’s eight pilot carbon markets, which puzzles their efficiency evaluation. This study tries to assess the efficiency of China’s eight pilot carbon markets and identify key factors influencing their performance, aiming to provide insights into how these markets can be optimized and achieves healthy condition.

**Methods:** A super-efficiency model is employed to measure China’s eight pilot carbon markets efficiency with undesirable output. Three indicators are chosen as input elements. The first is the number of participating enterprises, the second is the industry coverage quantity, and the last is the average carbon emission quota. The desirable output measures focus on turnover rate, and the concentration degree of transaction is chosen as undesirable output. Subsequently, a Tobit analysis is conducted to investigate the determinants of market efficiency, focusing on market liquidity, quota, transaction fee, volatility, and pre-distribution ratio.

**Results:** On the whole, there is an obvious increase in the average efficiency of China’s carbon markets over time, with values of 0.2728 in 2018 and 0.6376 in 2022. The Beijing, Shenzhen, and Guangdong markets are leading, whose average efficiency reaches 0.5812; Fujian and Tianjin are in the middle, with an average efficiency of 0.3610; Shanghai, Hubei and Chongqing fall behind, and their average efficiency is only 0.1505. Of the five variables used in the Tobit regression model, the coefficient of market liquidity is positive and statistically significant at the 1% level; the coefficient of quota is negative and significant; other coefficients are not statistically significant.

**Conclusions:** There is a positive relationship between market efficiency and liquidity; but a negative relationship between efficiency and quota, showing that markets with less quota and more liquidity are more efficient and healthy. Effects of transaction fees, price volatility and pre-distribution ratio are not important. It is suggested that relevant departments scientifically set carbon emission quotas, further expand participants, and improve the activity of the carbon market, contributing more to alleviate the anxiety caused by climate change and achieve carbon peak and carbon neutrality targets.

**Acknowledgments**

This paper is supported by the Philosophy and Social Sciences Planning Project of Shanxi [2022YJ078].

## MHBM24089: RESEARCH ON INVESTOR ANXIETY AND STOCK MARKET VOLATILITY IN THE DIGITAL AGE

Ziyang Zhou^a^, Qing Zhang^a^

^a^ *School of Economics and Finance, Zhanjiang University of Science and Technology, Zhanjiang, 524094, China.*

**Background:** In the digital age, the speed of information dissemination is growing exponentially, making it easier for investors to access stock market information. However, this ease of access also exposes them more readily to various messages, which can trigger anxiety. The interplay between investor anxiety and stock market volatility significantly impacts both financial market stability and investors’ mental health.

**Subjects and Methods:** This study focuses on the relationship and influence mechanisms between investor anxiety and stock market volatility, using case analysis and empirical research methods. From a psychological perspective, investor anxiety manifests as tension over uncertain and uncontrollable situations, leading to fatigue, difficulty in focusing, and even physical discomfort. When anxious, investors may overemphasize short-term market fluctuations, overreacting and thus increasing risk and uncertainty. The study also deeply analyzes changes in information dissemination and investor behavior in the digital age, including the effects of information overload, spread of negative information, and shifts in trading tools on investor anxiety.

**Results:** Empirical research indicates a positive correlation between investor anxiety and stock market volatility. When investors experience anxiety, they may engage in irrational trading behaviors, such as overtrading and following trends blindly. This not only raises transaction costs but also disrupts market liquidity, intensifying price fluctuations. A vicious cycle forms as market volatility triggers investor anxiety, which then drives irrational trading, further exacerbating market volatility. This volatility disrupts long-term investment plans, reduces asset values, and affects the predictability of future market trends, prompting investors to adjust their strategies and thereby increasing anxiety.

**Conclusions:** To address investor anxiety and market volatility, this paper proposes several strategic recommendations. These include strengthening investor education to raise risk awareness and investment skills, helping investors establish sound investment principles and risk understanding. Improving information disclosure policies, enhancing regulation of corporate disclosures, and boosting transparency and accuracy can reduce information asymmetry. Furthermore, market oversight should be reinforced to combat illegal practices like market manipulation and insider trading, ensuring fairness and transparency. Finally, psychological intervention mechanisms could be established to provide investors with counseling services, alleviating anxiety and improving psychological resilience and coping ability.

**Acknowledgments**

This research was financially supported by the Guangdong Province Philosophy and Social Sciences Planning Project [Project No.: GD24XYJ46]; and the Zhanjiang Philosophy and Social Sciences Planning Project [Project No.: ZJ23YB17].

## MHBM24090: CORPUS-BASED STUDY ON THE COGNITIVE PARADIGM DIFFERENCES IN CHINESE AND ENGLISH TOURISM TEXTS

Jianzhou Cui ^a^

^a^ *Wuxi City College of Vocational Technology, Wuxi, Jiangsu, 214153, China.*

**Background:** In the context of accelerating globalization and the booming tourism industry, effective communication through travel texts is crucial for attracting international tourists. The objective of this study is to investigate the cognitive paradigm differences in Chinese and Western tourism texts, with a specific focus on the linguistic features and stylistic strategies employed in Jiangsu tourism webpages compared to those from the UK and the USA. Cognitive psychology, which examines how individuals perceive, process, and interpret information, provides a theoretical framework for this analysis. The study aims to understand how these cognitive differences influence the effectiveness of travel texts in appealing to diverse tourist demographics and to offer insights for optimizing Jiangsu’s tourism promotion strategies.

**Subjects and Methods:** The study utilized a self-created corpus of English-translated Jiangsu tourism webpages, encompassing various tourist attractions and cultural highlights. This corpus was compared with comparable texts from the UK and the USA to identify cognitive paradigm differences. Quantitative and qualitative methods were employed, including corpus annotation and retrieval analysis software, to assess linguistic features such as lexical complexity, sentence structure, and thematic emphasis. The subjects of the study include the cognitive styles and predispositions of Chinese and Western tourists, as well as the linguistic manifestations of these cognitive differences in tourism texts.

**Results:** The findings indicate that Jiangsu tourism texts exhibit specific linguistic trends that reflect the holistic and context-oriented cognitive style prevalent in Chinese culture. Jiangsu texts use simpler vocabulary but with lower lexical variability compared to Anglo-American texts. The frequent use of complex sentence structures in Jiangsu texts may increase reading difficulty. Historical and cultural narratives, particularly those introducing significant dynasties and historical figures, are emphasized, which may create a sense of distance for tourists seeking lighter narratives. The higher frequencies of verbs, explicit structures, less use of nouns, articles, pronouns, and conjunctions, and frequent use of passive voice and “there-be” structures are observed. These features contrast with the analytical and object-oriented cognitive style common in Western culture.

**Conclusions:** The research underscores the need for Jiangsu’s tourism promotion strategies to adopt more diversified vocabulary and engaging narrative styles to enhance its tourism image and international appeal. Cognitive psychology provides valuable insights into how different cognitive paradigms can influence the perception and reception of tourism texts. By understanding these cognitive differences, Jiangsu can adjust its linguistic and stylistic strategies to optimize its international tourism communication strategies, promoting more effective global dissemination. This study not only offers concrete recommendations for Jiangsu’s tourism promotion but also provides implications for global tourism marketing practices, emphasizing the importance of considering cognitive paradigms in cross-cultural communication.

**Acknowledgments**

This paper is supported by “Qinglan Project” (WXCYQL20240502) of Wuxi City College of Vocational Technology.

## MHBM24091: THE IMPACT OF DIGITAL FINANCIAL INCLUSION ON HOUSEHOLD RISKY FINANCIAL MARKET PARTICIPATION BEHAVIOR: MEDIATING EFFECTS BASED ON PSYCHOLOGICAL COGNITION AND RISK PREFERENCES

Zhihui Li^a,b,c^, Xiaoming Guo^b^, Junliang Zhang^c^, Jie Tong^a^

^a^ *Business School, Chengdu University of Technology, Chengdu, Sichuan, 610059, China,* ^b^ *Rural Development Institute, Sichuan Academy of Social Sciences, Chengdu, Sichuan, 610071, China,* ^c^ *Institute of Social Development, Southwest University of Finance and Economics, Chengdu, Sichuan, 611130, China.*

**Background:** The cognitive, emotional, attitudinal and other psychological characteristics of residents involved in the investment decision-making process will result in the manifestation of bias in household risky financial market participation behaviour.

**Subjects and Methods:** This paper employs a multi-model approach, using the Probit model, Tobit models, and Poisson model, as well as the mediation effect model, to investigate the mechanism through which digital inclusive finance affects household risky financial market participation behavior. The analysis is conducted from the perspective of psychological cognition and risk preference. The data set is drawn from the China Household Finance Survey (CHFS) database, which comprises three years of data on 12,597 households.

**Results:** The regression coefficients of digital inclusive finance on the breadth, depth and diversification of family risky financial market participation are 1.0182, 0.1435 and 1.4758, respectively. All of these coefficients pass the 1% significance level test, indicating that digital inclusive finance has a significant positive effect on the breadth, depth and diversification of family risky financial market participation. The regression coefficients of digital inclusive finance and psychological cognition are significantly positive, and the regression coefficients of psychological cognition and digital inclusive finance and the three dimensions of family risky financial market participation are also significantly positive. This indicates that digital inclusive finance promotes participation in the family risky financial market by enhancing residents’ psychological cognition. The regression coefficients of digital inclusive finance and household risk preference are significantly positive, as are the regression coefficients of household risk preference and digital financial inclusion and household risky financial market participation. This indicates that digital financial inclusion can positively affect risky financial market participation behaviour of households through improving household risk preference.

**Conclusions:** Digital inclusive finance can significantly promote the breadth, depth and diversity of household risky financial market participation, and family members’ psychological cognition and risk preference play a mediating role.

**Acknowledgments**

This paper was supported by Sichuan Province Social Science Fund major project (Grant No. SCJJ24ZD24), Chengdu University of Technology Graduate Quality Engineering Key Project (Grant No. 2023YJG107) and Sichuan Science and Technology Program (Grant No. 2023NSFSC0523).

## MHBM24092: EMOTIONAL EXPRESSIONS AND MENTAL REFLECTIONS IN THE ENGLISH TRANSLATION OF DU FU’S *SPRING VIEW* FROM A DECONSTRUCTIVIST PERSPECTIVE

Guizhi Zhang^a,b^, Huanhuan Jiao^a^

^a^ *School of Foreign Languages, Henan University of Technology, Zhengzhou, Henan 450001, China,* ^b^ *School of Foreign Languages, Henan University of Technology, Zhengzhou, Henan 450001, China*

**Background:** In the era of globalized Chinese culture, English translations of Chinese classical poetry play a crucial role in cross-cultural communication. These translations not only promote traditional Chinese culture but also provide insight into the emotional and psychological dimensions of the original text. This paper explores the emotional expressions and mental reflections in the English translations of Du Fu’s *Spring View* from a deconstructivist perspective, with a focus on how these translations reflect Du Fu’s psychological experiences, particularly his suffering, grief, and trauma in response to national turmoil and personal loss.

**Subjects and Methods:** Using a self-built small corpus, this study analyzes two English translations of *Spring View* by Xu Yuanchong and W.J.B. Fletcher through discourse analysis. The corpus includes the original text, along with these two translations, to examine how the translators convey Du Fu’s emotional distress and mental health, especially in the context of war and displacement.

**Results:** The analysis reveals how translation choices reflect Du Fu’s emotional suffering, particularly his grief, displacement, and psychological distress in response to both national and personal crises. Xu’s translation uses concrete and imaginative expressions, which deconstruct and reconstruct the original text. His choice of vivid language brings Du Fu’s emotional turmoil into sharper focus, allowing readers to more directly engage with the poet’s internal suffering and psychological vulnerability. In contrast, Fletcher’s translation retains a more classical tone, using vocabulary that preserves the original text’s emotional depth while offering readers a more complex and multi-layered interpretation. Fletcher’s approach highlights the ambiguities of Du Fu’s grief, suggesting that mental suffering is multifaceted and open to interpretation. Both translations adhere to deconstructivist principles, emphasizing the polysemy and openness of the text, reflecting the continuous reconstruction and reinterpretation of language. These choices not only reflect Du Fu’s emotional suffering but also emphasize the transformative nature of mental health experiences, which are constantly evolving and subject to reinterpretation.

**Conclusions:** The two English translations of *Spring View* offer differing interpretations of Du Fu’s emotional and mental states, illustrating the shifting nature of meaning and the translator’s role in shaping these interpretations. From a mental health perspective, these translations provide insight into how emotional suffering, particularly displacement, grief, and trauma, transcends cultural boundaries. Xu’s translation creates an immediate emotional connection, while Fletcher’s version allows for more reflection and complexity, leaving space for readers to interpret the poem’s emotional and psychological dimensions. This research highlights how the psychological well-being expressed in Du Fu’s poem resonates across cultures, offering a universal understanding of mental health struggles. These translations show how literature serves as a bridge for cross-cultural empathy and understanding, revealing how mental health challenges are both individual and collective experiences.

**Acknowledgment**

This work was supported by a project grant from Department of Social Sciences, Ministry of Education (Grant No. 24YJA740009).

## MHBM24093: THE ROLE OF DIGITAL DISCOURSE IN SHAPING INTERGROUP FORGIVENESS AND MENTAL RECOVERY: A STUDY ON THE IMPACT OF A HISTORICAL TRAUMATIC EVENT

Tian Zhang^a^, Hui Sun^b^

^a^ *Department of Sociology, Nanjing University of Science & Technology, Nanjing, 210094, China,* ^b^ *School of Pre-school Education, Jiangsu Second Normal University, Nanjing, 210013, China.*

**Background:** In the realm of collective memory and historical trauma, certain events can cast long shadows over societal psyche, influencing interpersonal and intergroup relationships. This study explores how digital discourse surrounding a historical traumatic event (herein referred to as “the Event”) shapes intergroup forgiveness and its subsequent impact on mental health outcomes. The Event, while not specified directly, has been a source of ongoing anger, distrust, and psychological distress for many, highlighting the need to distinguish between perpetrators and the broader group they represent. The intersection of this topic with intergroup forgiveness and mental health, particularly regarding psychological needs, anxiety, and sensitivity, is of paramount importance.

**Subjects and Methods:** The research employed a quantitative approach, utilizing a sample randomly selected from shopping malls, residential areas, and cafés over a period of three months. A total of 152 participants, including 72 residents and 80 non-residents of a city significantly affected by the Event (hereafter referred to as “Affected City Residents” and “Non-Affected City Residents”), were surveyed. Participants were first exposed to news articles and online reviews concerning the Event, sourced from an official memorial account, and then completed the Intergroup Forgiveness Questionnaire. The online reviews were categorized as positive, neutral, or negative based on their directional characteristics, aiming to assess their influence on participants’ forgiveness levels and subsequent mental health perceptions.

**Results:** The findings revealed several significant correlations. Firstly, the directionality of online reviews (positive, neutral, or negative) had a notable impact on participants’ intergroup forgiveness levels. Secondly, the influence of these reviews on forgiveness varied between Affected City Residents and Non-Affected City Residents, indicating regional differences in psychological processing and forgiveness. Notably, compared to non-Chinese groups, Chinese respondents exhibited lower levels of intergroup forgiveness, which could be attributed to their perceptions of the historical event, cultural and political differences, and possibly deeper-seated psychological wounds. These findings suggest that the Event has had long-lasting psychological repercussions, influencing not just forgiveness but also mental health outcomes such as anxiety and sensitivity towards the group associated with the Event.

**Conclusions:** This study contributes to the understanding of how digital discourse shapes intergroup forgiveness and its impact on mental health outcomes following historical traumatic events. The results emphasize the need for future research to delve deeper into cultural backgrounds, the development of new measurement tools for intergroup forgiveness and mental health assessments, and the creation of intervention models tailored to address psychological needs arising from such events. Additionally, the study highlights areas for improvement, such as exploring the multidimensional nature of online reviews (including objective facts versus subjective sentiments) and expanding the sample size and geographical representation to enhance the study’s representativeness and generalizability. By acknowledging and addressing these factors, researchers can contribute to fostering a more forgiveness-oriented society and promoting mental health and well-being among those affected by historical traumas.

**Acknowledgments**

This work was supported by a project grant from Nanjing Massacre History Research Association (Grant No. YJH202306).

## MHBM24094: IMPROVED RESIDUAL MODEL FOR RECOGNITION OF STUDENTS’ EMOTIONAL AND PSYCHOLOGICAL STATES

Hanqing Hu^a^, Tong Gao^a^, Yuanyuan Jin^b^, Huizhi Lu^c^, Tianmu Tian^a^, Hongxi Zhang^d^

^a^ *Beijing University of Information Science and Technology, Beijing, 102200, China,* ^b^ *Beijing Vocational School of Information Technology, Beijing, 100070, China,* ^c^ *Fudan University, Shanghai, 200433, China,* ^d^ *Guizhou Minzu University, Guiyang, 550025, China.*

**Objective:** To address the lack of datasets about students’ emotional and psychological states in classroom environments and the limited applicability of existing expression recognition models in specific educational contexts. We propose an improved residual network model, Fer-IFPN, built on the ResNet50 architecture, tailored to recognize students’ emotional and psychological states in the classroom. This model aims to provide a foundation for timely optimization and adjustment of teaching strategies based on real-time emotional insights.

**Subjects and Methods:** To enhance the model’s performance, we designed a multi-scale feature fusion module that extracts students’ facial expression features at four scales. This approach allows the model to capture both minor local changes and overall expression characteristics, improving sensitivity to the nuances of varying expressions. Furthermore, we incorporated a hybrid attention module, which enables the model to concentrate on critical areas of the expressions, thereby minimizing interference from background noise and irrelevant information. In addressing the scarcity of existing datasets, we constructed a new student expression dataset, SFIC, specifically designed for training and testing our model.

**Results:** The experimental findings demonstrate that the Fer-IFPN model achieves a significant accuracy improvement of 6.6 percentage points compared to the original method. This enhancement validates the effectiveness of our model in accurately identifying students’ emotional and psychological states during classroom activities. The results suggest that the model can reliably distinguish between various emotional expressions, thereby facilitating a better understanding of students’ needs.

**Conclusions:** The Fer-IFPN model presents a substantial advancement in the recognition of students’ facial expressions, significantly improving classification accuracy in educational settings. By incorporating a multi-scale feature fusion and a hybrid attention mechanism, the model effectively captures essential features while reducing noise interference. The construction of the SFIC dataset further supports the development of robust expression recognition methods tailored to classroom environments. Overall, this research underscores the potential for improved emotional and psychological recognition of students, which can lead to more responsive and adaptive teaching strategies.

**Acknowledgements**

This work was supported by projects grant from Teaching Reform Project of School of Management Science and Engineering, Beijing University of Information Science and Technology (Grant No. 5112410819) and Beijing Information Science and Technology University Promotes the Development of Colleges and Universities, Base Construction, and the Construction of Innovation Practice Base in the School of Management Science and Engineering (Grant No. 5112410821).

## MHBM24095: RESEARCH ON THE IMPACT OF GLOBAL LY IMPORTANT AGRICULTURAL CULTURAL HERITAGE AND RURAL TOURISM ON TOURISTS’ MENTAL HEALTH

Ting Zhang^a^, Hongjuan Chen^a^, Yawen Xue^a^, Yang Chen^a^, Zhuyuan Li^a^

^a^ *Jiangsu Agri-animal Husbandry Vocational College, Jiangsu, Taizhou 225300, China*

**Background:** Globally Important Agricultural Heritage Systems (GIAHS) is a valuable resource for the development of rural tourism, and tourism development is a key driver in revitalizing both the rural economy and culture. Previous studies have shown that globally important agricultural heritage and rural tourism not only have significant implications for the economic development and cultural heritage of local communities, but also have corresponding effects on the mental health of tourists. However, the effects and pathways of these effects have not been clearly identified.

**Subjects and Methods:** The protection and inheritance of agricultural cultural heritage must be combined with the psychological needs of tourists. In order to elaborate on the effects and pathways of the psychological impact of globally important agricultural heritage systems and rural tourism on tourists, based on the representativeness, richness, inheritance effect, resource endowment, and local characteristics, 18 Chinese globally important agricultural cultural heritage sites are selected as analysis samples. Taking these 18 globally important agricultural and cultural heritage sites in China as samples, this study employs quantitative research as the main method and qualitative analysis as a supplementary to analyze the coupling and coordinated development trend, effectiveness, and experiences of integrating various agricultural cultural heritages with rural tourism, promoting rural cultural inheritance and industrial revitalization.

**Results:** The results show that the globally important agricultural cultural heritage sites in China are scattered, and the specific direction is still controlled by the central government. The tourism management and development modes of districts and counties vary depending on their geographical locations, resource endowments, economic foundations, and policy guidance. In addition, a long-term balanced and coordinated mutually supportive relationship is observed between these heritage sites and the rural tourism industry, with the coupling coordination degree evolving from severe imbalance to high-quality coordination. Furthermore, the coupling and coordination development of the wetland agricultural systems, vegetables and fruits, tea, and rural tourism industry is better, while the dry farming system remains underdeveloped. At last, the protection and transmission of globally important agricultural heritage systems must be combined with the psychological needs of the population, visitors’ experiences at agricultural cultural heritage sites are not only an exploration of history, but also a pursuit of their own cultural identity, psychological needs and emotional fulfillment.

**Conclusions:** Based on the study results and analysis of local practical experience, a sustainable system must be constructed for the protection and inheritance of globally important agricultural cultural heritage sites and industrial development. The system must consider the integration of policies and regulations at the political level, the integration of characteristic business forms at the economic level, and multicollaborative governance at the social level in order to catering to the psychological needs of tourists to “go to the countryside”.

**Acknowledgements** This work was supported by a project grant from Research on digital development of agricultural cultural heritage based on AR technology (Grant No. NSF2024ZR07).

## MHBM24096: AN EMPIRICAL ANALYSIS OF THE FACTORS AFFECTING THE PSYCHOLOGICAL TENDENCY OF B2C ONLINE SHOPPING CUSTOMER

Jiewen Xiang^a^, Bing Li^a^, Xia Jiang^a^, Yanyan Huang^a^, Qinqin Yao^a^, Liyan Xu^a^, Zhen Luo^a^

^a^ *Hangzhou Normal University, Hangzhou, Zhejiang, 311121, China.*

**Background:** The rapidly developing B2C online shopping market is still facing many challenges. A considerable number of Chinese customers have experienced dissatisfaction with online shopping, which causes complaints from time to time. “How to make customers more satisfied” is one of them when major e-commerce platforms are growing rapidly. Although customer satisfaction is a kind of psychological tendency that seems very difficult to hold, it is quite important for B2C websites to pay more attention to it and get the factors affecting online customer satisfaction. Therefore, to research the psychological tendency of B2C online shopping customer is quite significance.

**Subjects and Methods:** In this paper, the factors affecting online customer satisfaction in B2C e-commerce are studied from seven latent variables and twenty-seven observation variables. The latent variables include perceived quality, perceived value, customer expectations, B2C website image, online customer loyalty and complaints, online customer satisfaction. An indicator system and the Customer Satisfaction Index Model (OCSI) are constructed according to prior research. Then factor analysis and structural equation model (SEM) are used to analyze affecting factors from the survey data which is investigated by the research group. The respondents of the survey are customers who shopped on B2C e-commerce platforms in China.

**Results:** Firstly, the three most important common factors, the perceived factor, the credit factor, and the website security factor, are the key factors affecting online customer satisfaction. Secondly, seven hypotheses are tested and the results are consistent with all hypotheses. H1: Perceived value has a positive impact on online customer satisfaction. H2: Perceived quality has a positive impact on the perceived value of the online customer. H3: The expectations of online customers have negative impacts on perceived quality and perceived value.H4: The website images of B2C have positive impacts on expectations, loyalty, and the perceived value of online customers. H5: Online customer satisfaction has a negative impact on customer complaints. H6: Online Customer satisfaction has a positive impact on customer loyalty. H7: Customers complaints have negative impacts on customer loyalty.

**Conclusions:** As the perceived quality, merchant credit rating and network security issues have critical positive impacts on online customer satisfaction, where credit rating and customer service are two important variables that affect perceived quality, online customer satisfaction could be improved if B2C websites and companies could make improvements and complete measurements as shown on countermeasures according to these factors.

## MHBM24097: LANGUAGE SCHOOL READINESS AND PSYCHOLOGICAL WELL-BEING IN ETHNIC MINORITY CHILDREN

Wenwen Cheng^a^, Lizhu Wang^b^, Yuanying Wang^b^, Ou Peng^b^

^a^ *Department of Literature, Chongqing Normal University, Chongqing, 401331, China,* ^b^ *Department of Preschool Education, Chengdu University, Chengdu, Sichuan, 610106, China.*

**Background:** School readiness, particularly language readiness, is a crucial predictor of children’s future academic success and overall well-being. Recognized by the U.S. National Education Goals Commission as one of the five key areas of school readiness, language development holds a prominent place in shaping a child’s educational trajectory. In China’s ethnic minority regions, such as Qiandongnan in Guizhou Province, language readiness carries additional significance, influencing not only academic outcomes but also mental health and emotional development. This study examines the language readiness of young children from ethnic minorities, specifically the Miao and Dong groups, with an emphasis on the intersection of psychological factors and well-being within the context of language development and school preparedness.

**Subjects and Methods:** The study measured the language readiness of 1,258 kindergarten children aged 5-7 years from 17 kindergartens in three counties (Tianzhu, Congjiang, and Liping) of the Qiandongnan ethnic area in Guizhou Province. The sample included children from various ethnic backgrounds, predominantly Dong (1,019) and Miao (161). Employing a mixed-methods approach, the research involved three primary components: 1) Language School Readiness Level Measurement using the *“Evaluation of Children’s School Readiness (Language) Scale”*; 2) Questionnaires distributed to 1,322 parents to assess the home language environment, with a 97.1% valid response rate; 3) Semi-structured interviews with 12 teachers to gain insights into children’s language development and its potential impact on their psychological adjustment. The study utilized purposive and stratified sampling methods to ensure representation from county towns, townships, and rural areas.

**Results:** The findings revealed that the development of various dimensions of young children’s language school readiness is not synchronized. The best performance was observed in “pre-reading skills” and “recognition of subtle differences” (scores above 80%), while “visual transfer” and “describing diagrams” showed poorer performance (scores around 50%). Significant differences were found in the level of young children’s language school readiness between urban and rural areas and regions. However, the differences in language school readiness among different ethnic groups were not significant, suggesting that factors other than ethnicity, such as geographic location, may play a more significant role in influencing language readiness.

**Conclusions:** The study emphasizes the need for targeted strategies to promote balanced development of language readiness across all dimensions, particularly in rural areas. To address these disparities and potentially improve children’s mental well-being. Recommendations call for targeted interventions include narrowing the gap in urban and rural resource allocation, establishing a reasonable mechanism for the equitable mobility of early childhood educators across regions, and promoting an understanding of language readiness among parents in towns and villages. These measures aim not only to improve language readiness but also to support the mental and emotional health of young children in ethnic minority regions. Future research could explore the direct relationships between language readiness and mental health outcomes, including anxiety levels, happiness, and social-emotional development within these communities.

**Acknowledgements**

This work was supported by a project grant from Ministry of Education Humanities and Social Science Fund project “Collation and Research of Characters in Vietnamese Han Nan Chinese Ancient Books” (Grant No.23XJC40001) and Key project of Humanities and Social Science Planning of Chongqing Education Commission “Research on Chinese Characters from the Perspective of Foreign and Local Chinese Ancient Books” (Grant No. 22KGH088).

## MHBM24098: PSYCHOLOGICAL HEALTH PERSPECTIVES ON CARBON REDUCTION PATHWAYS IN CONSTRUCTION: INSIGHTS FROM VALUE CHAIN MANAGEMENT

Weiyi Li^a^, Yogi Tri Prasetyo^a^

^a^ *School of Industrial Engineering & Management, Mapua University, Manila, Philippines*

**Objective:** This study explores the integration of psychological health principles with value chain management to improve carbon reduction pathways in the construction industry. Specifically, it examines how psychological health factors, such as stress, motivation, and well-being, influence decision-making and behavioral adoption of carbon-conscious practices across various stages of the construction value chain.

**Methods:** A mixed-methods approach was used, combining quantitative analysis of carbon emission data from construction firms with qualitative insights from behavioral surveys and expert consultations. Psychological health indicators, including stress levels, job satisfaction, and employee engagement, were measured to understand their impact on the adoption of carbon reduction strategies. Case studies of leading construction firms were used to assess how aligning psychological health factors with environmental goals can enhance both individual well-being and organizational carbon outcomes.

**Results:** Positive psychological health outcomes (e.g., lower stress and higher job satisfaction) were linked to greater employee engagement in carbon reduction strategies. Interventions that promoted psychological well-being, such as supportive work environments and stress management programs, enhanced compliance with sustainable practices. Behavioral nudges, such as recognition and reward systems, had a direct positive effect on motivation and adherence to carbon-conscious practices, particularly in high-stress environments where employees might otherwise experience burnout. Firms that prioritized employee psychological health created a culture of environmental responsibility, which in turn led to greater innovation and more effective implementation of carbon reduction practices. The integration of mental well-being programs alongside carbon reduction efforts significantly improved employees’ ability to think creatively about sustainable solutions and contributed to a sense of collective purpose. The stages of material procurement and waste management were identified as particularly sensitive to employee well-being. When employees felt psychologically supported, they were more likely to make decisions that prioritized sustainable materials and effective waste reduction, contributing to lower carbon footprints.

**Conclusion:** Incorporating psychological health principles into value chain management offers a powerful framework for improving both carbon reduction and employee well-being in the construction industry. By addressing mental health issues such as stress and burnout, and fostering an environment of support and motivation, firms can enhance compliance with carbon reduction strategies and drive sustainable innovation. This approach not only improves organizational sustainability but also promotes healthier, more engaged workforces. Future research should explore the long-term effects of integrating psychological health with environmental practices in the construction industry, with a focus on scaling these interventions for industry-wide impact.

## MHBM24099: RESEARCH ON THE MECHANISM OF THE INTEGRATION OF THE SPIRIT OF ‘TWO BOMBS AND ONE SATELLITE’ PROJECT INTO THE CIVICS OF SCIENCE AND ENGINEERING COURSES ON THE ENHANCEMENT OF STUDENTS’ MENTAL HEALTH

Yang Zou^a^, Bo Liu^b^

^a^ *MianYang Teachers’ College, MianYang, Sichuan 621000, China,* ^b^ *MianYang Teachers’ College, MianYang, Sichuan 621000, China.*

**Background:** With the intensification of global competition in science and technology and the increase in the social demand for innovative talents, science and technology education not only faces challenges at the academic and technical levels, but the mental health of students is also receiving more and more attention. Under the multiple pressures of academic pressure, scientific research failure and employment competition, science and engineering students are prone to psychological problems such as anxiety, depression and low self-esteem. Therefore, how to help students improve their psychological quality and enhance their stress resistance through ideological and political education has become an important topic in modern education. As an important spiritual wealth in the history of Chinese science and technology, the spirit of ‘Two Bombs and One Satellite’ Project, embodied by patriotism, dedication, self-reliance and teamwork, provides valuable spiritual guidance for the psychological growth of science and technology students.

**Subjects and Methods:** Combining the theoretical basis of ideological education and mental health, this paper discusses the promotion of students’ psychological growth by integrating the spirit of “Two Bullets, One Star” into the science and engineering curriculum. Through literature analysis and case studies, this paper analyses the positive impact of the ‘Two Bullets, One Star’ spirit on students’ mental health from the perspectives of value guidance, psychological needs satisfaction, personality shaping, and double promotion of emotion and cognition. The study focuses on how to enhance students’ stress resistance and psychological resilience through curriculum education, and how to promote students to maintain a healthy psychological state in the face of academic challenges through the cultivation of teamwork and sense of social responsibility.

**Results:** The core elements of the spirit of ‘Two Bombs and One Satellite’ Project can effectively help students to enhance their mental toughness in academia and life. In terms of value guidance, through learning the spirit of selfless dedication of scientific researchers, students have strengthened their sense of identification with the collective and the country, and gained a sense of belonging and value. In terms of psychological needs satisfaction and personality shaping, the study of the spirit of ‘Two Bullets and One Star’ helped students enhance their sense of self-efficacy and responsibility, and reduce anxiety and low self-esteem. The combination of cognitive and emotional education helped students maintain a positive and optimistic mindset in the face of scientific research failure and academic pressure.

**Conclusions:** The core elements of the ‘Two Bullets, One Star’ spirit have had a positive and significant impact on students’ mental health. Firstly, in terms of value guidance, through learning the spirit of selfless dedication of scientific researchers, students strengthened their sense of identification with the country and the collective, enhanced their sense of belonging and value, and then effectively alleviated the anxiety and loneliness caused by academic pressure and life challenges. Secondly, in terms of psychological needs satisfaction and personality shaping, the learning of the spirit of ‘Two Bullets, One Star’ helped students enhance their sense of self-efficacy and responsibility, strengthened their psychological resilience in the face of academic pressure and scientific research failures, and significantly reduced negative emotions such as low self-esteem and depression. In addition, students’ psychological adaptability was enhanced through the cultivation of teamwork and social responsibility in the course, and they showed stronger emotional regulation and maintained a positive and optimistic mindset in the face of scientific research setbacks. The combination of cognitive and emotional education further promotes the overall development of students’ mental health.

**Acknowledgments**

This work was supported by a project grant from Sichuan Provincial Education and Research Funding Project: Mining and Sorting out Ideological and Political Elements of Science and Engineering Courses for Meritorious Scientists of the ‘Two Bombs and One Satellite’ Project Program (Grant No. SCJG21A100).

## MHBM24100: THE FINANCING EFFICIENCY AND AFFECT FACTOR OF AI ENTERPRISES IN CHINA FROM PSYCHOLOGICAL HEALTH

Bingyun Zheng^a^, Shaocong Zhu^a^, Long Ding^a^

^a^ *Anhui University of Finance and Economics, Bengbu, China*

**Background:** The artificial intelligence (AI) industry plays a crucial role in transforming and upgrading the economic structure in China. Currently, AI enterprises face a relatively favorable financing environment, yet financing efficiency has become a key factor for the sustainable development of these companies. Financing efficiency is critical for AI enterprises, especially considering the highly competitive nature of the industry and the financial challenges they face. Understanding the factors influencing financing efficiency can provide essential insights for better capital allocation and business strategies. Additionally, the psychological health of enterprise founders and managers, such as stress, anxiety and emotional stability, can also play a crucial role in shaping the decision-making processes that impact financing outcomes.

**Subjects and Methods:** This study aims to measure and analyze the financing efficiency of AI enterprises in China, identify key influencing factors, and offer practical suggestions for improving financing strategies. To evaluate the efficiency, this research constructs a game cross-efficiency model, which incorporates both competitive and cooperative dynamics in financing activities. Moreover, the Tobit model is employed to explore the factors affecting financing efficiency, including organizational size, profitability, and growth potential. The psychological health of management could significantly affect managerial decisions regarding financing strategies and the overall ability to secure funds.

**Results:** The results of the game cross-efficiency model indicate that the financing efficiency of AI enterprises is generally low, with notable variations across different firms. Some companies demonstrate higher efficiency, while others struggle to achieve optimal financing results. The findings from the Tobit model suggest that company scale, profitability, and growth prospects have a significant and positive impact on financing efficiency. However, the level of debt financing negatively affects efficiency, highlighting the importance of balancing debt and equity financing in the decision-making process. The psychological health of management significantly and positively affect the financing efficiency

**Conclusions:** This study highlights that financing efficiency in AI enterprises is influenced by various organizational and financial factors, such as scale, profitability, and growth potential. A high level of debt financing is detrimental to financing efficiency, which underscores the importance of maintaining a balanced financial structure. To optimize financing strategies, AI enterprises should focus on improving their growth capabilities and financial management while avoiding over-reliance on debt. Furthermore, enhancing psychological health and cultivating positive mental health among business leaders and decision-makers can lead to more effective leadership, better decision-making, and ultimately improve financing efficiency.

## MHBM24101: STUDY ON VICTOR’S INFERIORITY COMPLEX IN *FRANKENSTEIN* FROM THE PERSPECTIVE OF ADLER’S INDIVIDUAL PSYCHOLOGY

Tong Li^a^, Ruotong Lu^a^, Jin Xu^a^

^a^ *School of Foreign Languages, Tianjin University, Tianjin, 300350, China*

**Objective:** The objective of this study is to analyze the character Victor Frankenstein from Mary Shelley’s *Frankenstein* through the lens of Adler’s individual psychology, focusing on his inferiority complex and its role in his tragic fate. The study aims to understand the manifestations, origins, and compensations of Victor’s inferiority complex and to explore the implications for individuals who may experience similar feelings.

**Subjects and Methods:** The study focuses on the character Victor Frankenstein and examines his psychological development. The investigation employs a qualitative research methodology and involves an in-depth textual analysis concerning inferiority complex. It scrutinizes Victor’s actions, motivations and psychological underpinnings based on Adler’s theories on inferiority and compensation. The study examines the overt and covert manifestations of Victor’s inferiority complex, and then delves into the roots of this complex within the realms of family, education, and society. Finally, it evaluates Victor’s strategies to compensate for and surpass his feelings of inferiority.

**Results:** The findings of the study demonstrate that Victor’s inferiority complex is the result of a complex interplay between environmental influences and his own conduct. His endeavors to rectify his perceived deficiencies not only prove ineffective in alleviating his sense of inferiority but also serve as a catalyst for his ultimate downfall. The research demonstrates that Victor’s inability to establish constructive goals and engage in collaborative efforts with others exacerbates his psychological distress, ultimately resulting in his tragic demise.

**Conclusions:** The study concludes that Victor’s inferiority complex, shaped by his environment and personal choices, is the primary factor that determines the tragic trajectory of his destiny. It is of the utmost importance that families, schools and society as a whole foster social interest and provide greater attention and love to assist victims with feelings of inferiority. It is imperative to comprehend the intricate behaviors and psychological processes of isolated individuals such as Victor in order to facilitate their healthy growth and development. Moreover, individuals must learn to regulate their emotional states, make effective compensations for feelings of inferiority, and strive for self-transcendence in order to avoid the destructive path that Victor followed.

## MHBM24102: COURSE DESIGN OF HANDWRITING ANALYSIS FROM THE PERSPECTIVE OF IDEOLOGICAL AND POLITICAL EDUCATION TO SUPPORT STUDENTS’ MENTAL HEALTH

Zhihui Ban^a^, Wenbo Fu^a^

^a^ *The National Police University for Criminal Justice, Baoding 071000, China.*

**Background:** Mental health issues among college students have become increasingly prominent with the intensification of social competition and increase of learning pressure, attracting great attention from all sectors of society. As a cradle for talent development, higher education institutions bear the critical responsibility of fostering comprehensive development of students, encompassing not only knowledge transmission, but also psychological quality cultivation. As a psychological analysis tool, handwriting analysis can reveal the psychological state of an individual through their writing characteristics, providing strong support for mental health education. From the perspective of ideological and political education, integration of handwriting analysis into course design aims to guide students to self-awareness, emotional management, and enhancing psychological resilience through scientific methods to promote their mental health development.

**Subjects and Methods:** This research explored the application pathways of handwriting analysis in mental health education course design for college students, based on the context of ideological and political education. Firstly, it clarified the theoretical basis of handwriting analysis and mental health, emphasizing on intrinsic connection between the two. Secondly, different teaching methods including case analysis, group discussions, and role-playing were applied during teaching process to enhance the engagement and experiential learning of students. At the same time, ideological and political education elements such as professional ethics and collectivism were introduced to help students establish correct values and a sense of social responsibility while learning. Through course effectiveness assessments, changes in the handwriting analysis and emotional management abilities of students were observed and recorded to evaluate their mental health status.

**Results:** After the implementation of the designed course, a significant improvement was observed in the mental health levels of students. Handwriting analysis course design not only helped students better understand themselves and their psychological states, but also strengthened their emotional management skills and psychological resilience. In addition, integration of ideological and political elements fostered a sense of social responsibility and collective honor among students during their learning process. Handwriting analysis helped students in self-awareness, improving their overall quality and cultivating a new generation equipped with social responsibility and innovative capabilities, effectively elevating their mental health levels.

**Conclusions:** Integrating handwriting analysis into mental health education course for college students was found to be a valuable attempt within the framework of ideological and political education. By applying scientific methods to guide students to self-awareness, emotional management, and psychological resilience enhancement, it can not only promote their mental health development, but also cultivate their sense of social responsibility and collective honor. In the future, further exploration of handwriting analysis application models in mental health education is required to subtly improve the qualities of students and help them establish correct mental health concepts.

**Acknowledgments**

This research was supported by the Central Judicial Police Academy’s university-level project titled “Research and Exploration of Teaching Reform in Document Examination Courses Based on OBE Educational Philosophy” (Project No. XYJ202002).

## MHBM24103: ANALYSIS OF TOURIST SATISFACTION IN HISTORICAL AND CULTURAL TOWNS BASED ON ONLINE REVIEWS

Xiwen Hao^a^, Juanjuan Luo^a^

^a^ *Faculty of New Commercial Science, Anhui Sanlian University, Heifei, 230601, China.*

**Background:** Tourist satisfaction is a comprehensive feedback on the degree to which tourists meet their needs for tourism resources, infrastructure, tourism services, and other aspects of a scenic spot. Compared with traditional methods such as questionnaire surveys and interviews, online comments have the advantages of being more spontaneous, authentic, and accurate, and have become important data for measuring tourist satisfaction. In view of this, this article selects Sanhe Ancient Town as a research case and evaluates its tourist satisfaction, providing reference and guidance for the management of historical and cultural famous towns.

**Subjects and Methods:** This paper first uses ROST CM6 to extract high-frequency keywords from tourists’ online reviews. Secondly, the extracted high-frequency words were summarized to determine the secondary indicators that affect tourist satisfaction. Thirdly, further classify the secondary indicators into primary indicators and construct a tourist satisfaction evaluation index system. Finally, this paper evaluates the perceived importance and satisfaction of tourists with different attributes of the scenic area based on the frequency of high-frequency word occurrence and the average sentiment score. Then, IPA analysis method is used to evaluate the satisfaction of tourists with various attributes in the indicator system.

**Result:** The paper finds that: (1) Although the overall satisfaction of tourists in Sanhe Ancient Town is relatively high, there is still significant room for improvement. (2) The dissatisfaction of tourist is mainly concentrated in degree of commercialization, supporting facilities, geographical location, convenient transportation, infrastructure, cultural sites, historical vicissitudes, architectural style, paid Services. (3) Sanhe Ancient Town needs to be optimized in terms of cultural sites, geographical location, convenient transportation and other attributes. historical vicissitudes, degree of commercialization, architectural style, infrastructure, scenic spot publicity, experience feeling, tour cost performance, travel time, peers, management order, atmosphere of life belong to secondary priority improvement attributes.

**Conclusion:** (1) Tourists will compare their perceived performance with their expectations, and when the perceived performance is lower than their previous expectations, it will lead to a decrease in satisfaction. (2) Tourists visiting scenic spots hope to gain a authentic experience, and local culture is the key to authenticity. (3) Tourists will frequently visit areas with convenient transportation, commercial gathering, and unique scenic spots. To improve tourist satisfaction, it is necessary to plan and construct these key areas well. (4) Historical and cultural towns should concentrate resources to do a good job in the protection and restoration of cultural relics; Make good use of the Internet and big data to help tourists reasonably plan their tour routes and avoid congestion. On the other hand, historical and cultural towns should conduct characteristic exploration, extract cultural connotations, and plan characteristic boutique tourism projects.

**Acknowledgements**

This study was supported by the Anhui Provincial Higher Education Science Research Project (Fund numbers: 2023AH051689, 2024AH052448).

## MHBM24104: A STUDY ON THE INFLUENCE OF ICONOLOGY ON MENTAL HEALTH FROM THE PERSPECTIVE OF ART HISTORY AND FILM AESTHETICS

Jing Ding ^a^

^a^ *Hechi University, Hechi, Guangxi, China, 546300.*

**Objective:** This study aims to examine the theories of imitation and formalism in the definition of traditional art from the perspective of iconology, explore the mechanisms of the impact of iconology on individual mental health, analyze how images act on human psychological structures and subsequently affect their mental health status, and promote a deeper understanding and scientific evaluation of the psychological health effects of image media.

**Subjects and Methods:** Classic and contemporary films from different historical periods and cultural backgrounds were selected as research objects, covering from the artistic exploration of the silent film era to the visual feast of contemporary digital cinema. By applying emerging research methods such as film aesthetics, we can fully understand the creators’ aesthetic perception, aesthetic emotions, and psychological factors.

**Results:** The study found similarities and correlations between iconographic methods and classical film theories. Film aesthetics not only shapes the audience’s aesthetic preferences but also subtly influences the audience’s emotional state and mental health through visual language such as color, composition, and light and shadow. In terms of iconology, the symbols, metaphors, and narrative structures in films can stimulate deep psychological resonance in the audience, promoting self-awareness, emotional release, or triggering the review and healing of psychological trauma.

**Conclusion:** By applying emerging research methods such as film aesthetics, we can gain a deeper understanding of the psychological expression and function of works of art. Film is not only a vehicle for entertainment but also a medium for exploring and growing the mind. It can be a positive promoter of mental health but may also exacerbate psychological distress in certain circumstances.

**Acknowledgments**

This paper is supported by Guangxi Educational Science “14th Five-Year Plan” 2023 Annual Special Project for Innovative and Entrepreneurial Education in Colleges and Universities, “Research on the Cultivation Model of Innovative and Entrepreneurial Talents in Minority Nationality Colleges and Universities with Ideological and Political Education as the Guide, Integration of Specialization and Innovation, and Industry-Academia Integration”, Project Number: 2023ZJY1489.

## MHBM24105: OPTIMIZATION OF THE PERFORMANCE EVALUATION INDEX SYSTEM FOR UNIVERSITY FACULTY: EMPHASIS ON PSYCHOLOGICAL NEEDS AND WORK STRESS

Wei Xia ^a,b^

^a^ *China University of Mining and Technology, Xuzhou, 221116, Jiangsu, China,* ^b^ *Wuxi Taihu University, Wuxi, 214000, Jiangsu, China.*

**Objective:** This study aims to optimize the performance evaluation index system for university faculty, emphasizing factors related to mental health, such as psychological needs, work stress, anxiety, and personality traits. The objective is to address gaps in traditional evaluation systems by recognizing the impact of these psychological factors on faculty productivity, motivation, and overall well-being. By developing a more comprehensive performance evaluation system, the study seeks to create an environment where faculty members’ psychological well-being is supported alongside their teaching and research achievements.

**Subjects and Methods:** This research employed a questionnaire-based survey to gather data from university faculty regarding their perceptions and experiences of the current performance evaluation system, particularly focusing on its impact on their mental health. A sample of faculty members from different departments was surveyed, capturing key indicators such as teaching workload, professional ethics, research development, and psychological factors. Data analysis included assessing the alignment of faculty psychological needs, work-life balance, and stress management within the context of their performance metrics. To further refine the evaluation criteria, the study applied the Key Performance Indicators (KPI) method, emphasizing multidimensional analysis to capture the nuanced impacts of performance evaluation on faculty mental health. Specifically, this involved weighting psychological factors such as job satisfaction, burnout risk, and motivation, to better understand how these elements influence both personal and professional growth.

**Results:** The survey results indicate that faculty members generally feel the current evaluation system lacks sufficient attention to mental health, contributing to increased work-related stress and reduced job satisfaction. Findings show that, due to rigid and narrowly focused criteria, faculty members often face heightened anxiety and difficulty balancing professional responsibilities with personal well-being. Furthermore, the absence of a robust feedback mechanism limits faculty members’ ability to address work challenges effectively and obtain necessary psychological support. To mitigate these issues, several recommendations are proposed: first, a revision of evaluation criteria to incorporate mental health considerations; second, the implementation of scientifically determined evaluation cycles that reflect realistic faculty workloads; and third, the establishment of feedback channels that enable continuous performance improvement and psychological support.

**Conclusions:** Optimizing the performance evaluation system requires a dual focus on faculty professional advancement and psychological health. By addressing mental health aspects such as stress management and emotional support, universities can improve faculty well-being, enhance motivation, and increase job satisfaction. Ultimately, a well-rounded evaluation system that prioritizes mental health can foster a supportive academic environment, leading to higher-quality teaching and research output and contributing to the long-term sustainability of university education systems.

**Acknowledgements**

This work was supported by a project granted from 2024 Wuxi Municipal Philosophy and Social Sciences bidding project (special project for social education development): collaborative education of “Home-School Community” to Improve Adolescents’ Mental Health Literacy (WXSK24-JY-B07).

## MHBM24106: RESEARCH ON THE PSYCHOLOGICAL DEMAND AND INFLUENCING FACTORS OF THE RENEWAL OF LAND TRANSFER IN FAMILY FARMS

Yong Lan^a^, Fu Liu^a^, Min Jiang^a^

^a^ *Business School, Hunan Agricultural University, Changsha, 410128,China.*

**Background:** In the context of the “separation of three rights” of land, it is an effective way to promote the moderate scale of operation by transferring land to family farms for non-agricultural employment, and further exploring the psychological needs of family farms for the renewal of mainstream land transfer is related to their acquisition of long-term stable land management rights and improving the efficiency of their scale operation, which is of great practical significance for the sustainable development of family farms.

**Subjects and Methods:** According to the decision-making process of the renewal psychological needs of family farms’ main land transfer, the psychological needs of family farms’ land transfer are divided into three levels. Based on the micro-survey data of 386 family farms in Hunan Province, the Triple-Hurdle model is used to systematically analyze the psychological needs of family farms’ land transfer and its influencing factors.

**Results:** Variables such as annual pure income, land connection, circulation period, and government subsidy have significantly positive psychological needs of family farms that circulate land; Variables such as land adjustment psychological needs, and circulation methods have significantly negatively negatively-adjusted psychological needs of family farms. Variables such as gender, age, pure income, government subsidies, and agricultural product market environment of family farmers significantly affect the expansion of the land transfer of family farms. Variables such as the age of farm owners and farmers’ default rates significantly affect family farms. The extension of the circulation of land; the land rent is significantly positively affecting the reduced area of the land transfer of the family farms. Variables such as gender, government subsidy, and loan difficulty of farm owners have significantly negatively affected the reduction of the area of land circulation of family farms.

**Conclusions:** The psychological needs of the mainstream of family farms are generally high, and there are many influencing factors. These factors affect the business willingness and actual area of the farmers in the future. Further propose to fully implement the training of family farmers, including skill training, psychological training, etc., comprehensively improve the level of family farm management and the psychological tolerance of risks; Gradually reduce the psychological needs of farmers ‘small operating area; we must improve the construction of agricultural land trading platforms, establish a reasonable land rent formation mechanism, reduce the impact of farmers’ liquidation and high rent on the psychological expectations of farmers; To improve agricultural production conditions through land leveling and other means, improve the efficiency of agricultural land utilization; to improve the government subsidy system, reduce the psychological cost burden of family farmers, improve the rural financial system, financial support gradually tilted towards family farms, reduce loan loans on family farms, and reduce loans on family farms. Suggestions such as risks.

**Acknowledgments**

This paper was funded by the National Social Science Fund Project “Research on the long-term mechanism of land management right stability of family farms from the perspective of life cycle” (Grant No. 16BJY093).

## MHBM24107: DISTRIBUTED LEADERSHIP, COLLABORATIVE MANAGEMENT STRATEGIES, AND INNOVATION IN CHINESE UNIVERSITIES: AN ANALYSIS FROM THE PERSPECTIVE OF EFFICACY

Yang Qian^a,b^, Arnie Christian D. Villena^c^

^a^ *Lyceum of the Philippines University Batangas, Batangas 4200, Philippines,* ^b^ *Faculty of Modern Health Care, Anhui Sanlian University, Hefei, Anhui 230601, China,* ^c^ *Dean of College of Education, Arts and Sciences, Lyceum of the Philippines University Batangas, Batangas 4200, Philippines.*

**Background:** This study investigates the relationship between distributed leadership, collaborative management strategies, and innovation in Chinese universities. Despite significant strides in Chinese higher education, many institutions still rely on traditional hierarchical leadership, which limits faculty and administrative contributions to innovation and creates psychological challenges, such as increased stress and unmet psychological needs. These dynamics can undermine both individual and organizational efficacy. Distributed leadership offers a promising approach to foster collaboration, address psychological well-being, and support innovation. However, its integration varies significantly across institutions, leading to uneven outcomes in research, teaching, and administrative effectiveness. This study explores strategies to better align these elements for a more innovative and psychologically supportive academic environment.

**Methods:** This study employed a descriptive research design, engaging 435 faculty members across various Chinese universities. Data were collected through a comprehensive survey examining distributed leadership, collaborative management strategies, innovation, and their impacts on psychological factors such as individual efficacy and mental well-being. The questionnaire utilized a four-point Likert scale to assess perceptions of decision-making, faculty empowerment, psychological needs, and stress management. Statistical analysis, including Spearman’s correlation, evaluated the relationships among distributed leadership, organizational efficacy, and innovation. Non-parametric tests were also used to explore demographic differences and their influence on psychological and organizational outcomes.

**Results:** Findings reveal that distributed leadership practices, characterized by shared decision-making and faculty empowerment, positively correlate with collaborative management strategies, innovation, and individual and organizational efficacy. Respondents highlighted that transparent decision-making and interdisciplinary teamwork reduced psychological stress and fulfilled psychological needs, fostering a supportive environment. Enhanced communication and conflict resolution contributed to innovation outcomes, such as increased research productivity, advanced teaching methodologies, and technological integration. However, inconsistent implementation and insufficient support for faculty development and mental well-being posed significant challenges.

**Conclusions:** The study concludes that distributed leadership, integrated with collaborative management strategies, not only supports innovation but also enhances individual and organizational efficacy in Chinese universities. By addressing psychological needs, reducing stress, and fostering shared decision-making, institutions can create a more inclusive and adaptive academic environment. These findings emphasize the need for leadership models that prioritize mental well-being and efficacy to sustain innovation. A continuous improvement program is proposed to guide universities in integrating distributed leadership, collaboration, and psychological support for academic excellence and competitiveness.

**Acknowledgements**

This work was supported by Anhui Provincial Department of Education Natural Science Key Project “Application Research of Machine Vision Technology in Abnormal Behavior Detection System for the Elderly” (No.2024AH050520), Anhui Provincial Department of Education Quality Engineering Project “Embedded Single Chip Microcomputer Application Technology” (No.2023xxkc387), Anhui Provincial Department of Education Quality Engineering Project “Electrical Engineering and Intelligent Control Excellent Engineer Education and Training Program” (No.2022zybj035), Anhui Sanlian University-level Collaborative Innovation Center Key Project “Application Research of Service Robot Face Detection Technology” (No.zjqr23002), Anhui Sanlian University-level Collaborative Innovation Center Key Project “Application Research of Exoskeleton Rehabilitation Training Robot” (No.zjqr23004), Anhui Sanlian University Quality Engineering Project “Service Robot Application Technology Course Teaching Team” (No.22zlgc108).

## MHBM24108: INVESTIGATION OF THE PROMOTIONAL VALUE AND STRATEGIC IMPLEMENTATION OF MINDFULNESS EDUCATION FOR THE MENTAL HEALTH OF MEDICAL STUDENTS

Jing Fang^a^, Jing Liu^b^, Xiang Chen^c^

^a^ *The Second Clinical Medical College of Zhejiang Chinese Medical University, Hangzhou, China,* ^b^ *College of Humanities and Management, Zhejiang Chinese Medical University, Hangzhou, China,* ^c^ *Zhejiang Chinese Medical University, Hangzhou, China.*

**Background:** Medical students inhabit an environment replete with hurdles and examinations. They must not only engage in rigorous academic endeavors, including the assimilation of extensive medical knowledge, intricate case analyses, and demanding scientific research experiments, but also confront the high-pressure clinical practice phase, actively participate in medical procedures, and grapple with issues of life and death, suffering, and humanity. Faced with such dual pressure, medical students will be able to engage in medical practice. Medical students frequently encounter mental health challenges, including anxiety and depression, which can adversely affect their academic and professional trajectories and potentially impair the quality of their future medical practice. In recent years, mindfulness education has emerged as a significant psychological intervention method, emphasizing its crucial role in enhancing the mental health of medical students. This study aims to thoroughly assess the impact of mindfulness education on enhancing the mental health of medical students and to suggest realistic implementation strategies to inform the future development of mental health curricula in higher medical education.

**Subjects and Methods:** This study employs a literature review methodology to thoroughly investigate the beneficial effects of mindfulness education on the mental health of medical students and its implementation methodologies, emphasising the positive influence of mindfulness education on their mental well-being.

**Results:** Mindfulness education has demonstrated considerable efficacy in addressing several psychological issues, including anxiety, depression, stress management, and emotional control. It is essential to enhance the education and promotion of mindfulness cognition, focus on the training and enhancement of mindfulness skills, broadly apply the application and expansion of mindfulness practice, and develop a robust support and assessment system for mindfulness education.

**Conclusions:** This study demonstrates that mindfulness education can substantially enhance the mental health of medical students and proposes an effective approach to offer useful insights and direction for the advancement of mental health in medical education. Nevertheless, in light of the absence of empirical evidence, forthcoming research should concentrate on the impacts of mindfulness education across various grade levels, individual variances, and specific contexts pertaining to medical students. Additionally, it should investigate the advancement and dissemination of digital and remote education tools, as well as implement systematic and multidimensional monitoring and assessment of the long-term effects of mindfulness education. It seeks to deliver more accurate and individualised mental health assistance for medical students.

## MHBM24109: EXPLORING ART PREFERENCES OF RETIRED SENIORS: UNDERSTANDING PSYCHOLOGICAL NEEDS AND WELL-BEING THROUGH CHINESE FREEHAND FLOWER AND BIRD PAINTINGS

Yuze Kang^a^, Jiamei Wu^b^, Shureen Faris Binti ABD Shukor^b^, Mohd Fabian Hasna^b^, Mohd Najmi Daud^b^

^a^ *China Academy of Art, Hangzhou, Zhejiang, 310002, China,* ^b^ *Universiti Putra Malaysia, Serdang Selangor, 43400, Malaysia.*

YK and JW contributed equally to this study.

**Background:** The increasing prevalence of mental health issues such as loneliness, anxiety, panic, and depression among older adults necessitates innovative approaches to enhance their psychological well-being. These issues are compounded by social isolation, losing loved ones, and the challenges of adapting to retirement. This study explores how engagement with art, mainly traditional Chinese freehand flower-and-bird painting, can serve as a therapeutic intervention. By understanding the artistic preferences of retired seniors in China, we gain valuable insights into their psychological needs and cultural connections, essential for fostering mental health in this demographic.

**Subjects and Methods:** A qualitative study was conducted involving retired seniors in China, utilizing the Nominal Group Technique (NGT) to gather their artistic preferences systematically. NGT sessions allowed participants to engage in meaningful discussions about their tastes, emotional responses, and the significance of various artworks. These sessions were complemented by a historical review of Chinese freehand paintings, analyzing 312 works from different dynasties. This comprehensive approach aimed to identify patterns in artistic preferences that resonate with the participant’s psychological and cultural contexts, thereby enriching our understanding of the role of art in their lives.

**Results:** The findings revealed a strong preference among retired seniors for floral motifs characterized by simple brushwork and subtle color palettes. Emotional expressiveness emerged as a critical factor in their appreciation of these artworks. Five favorite paintings stood out: Simple Flowers and Birds by Zhu Da, Yandang Mountain Flowers, Lotus Picture by Pan Tianshou, Water and Wood by Tsinghua University, and Two Birds in Lotus Pond by Zhu Da. Participants reported that these works evoked feelings of calm and tranquility while fostering a sense of cultural identity, significantly alleviating loneliness and anxiety.

**Conclusions:** Integrating traditional Chinese art into therapeutic and recreational activities presents a promising avenue for enhancing emotional and cultural well-being among older adults. This study underscores the importance of addressing psychological needs through artistic engagement, highlighting the potential of traditional art to improve mental health outcomes. Future research should further explore how art influences psychological states and emotional responses, potentially leading to innovative geriatric mental health care practices that leverage art’s therapeutic power to enrich older adults’ lives.

## MHBM24110: PBLI: A NEW PARADIGM FOR JAVA PROGRAMMING TEACHING TO ENHANCE LEARNING EFFICIENCY AND REDUCE LEARNING ANXIETY

Xiao Tan^a^, Zhenjin Li^a^, Muyangzi Lin^a^, Qing Wang^a^, Xilong Qu^b^

^a^ *Hunan University of Finance and Economics, Changsha, 410205, China,* ^b^ *Changsha Normal University, Changsha, 410000.China.*

**Background:** JAVA programming courses are known for their complex and practice-oriented content, which often leads to heightened learning anxiety among students, negatively impacting their learning outcomes. Traditional teaching methods tend to be lecture-focused, offering limited opportunities for active engagement and failing to adequately address students’ mental health needs. To address these challenges, this study proposes the PBLI (Project-Based Learning with Intelligent support) model, which integrates project-based learning, anxiety management, and an intelligent teaching platform to enhance learning efficiency and reduce anxiety. By incorporating personalized feedback and task-driven projects, PBLI enables students to progressively develop programming skills in a supportive environment that addresses both their cognitive and emotional needs.

**Subjects and Methods:** A controlled experiment was conducted over 16 weeks with 83 university students enrolled in a JAVA programming course, divided into an experimental group (PBLI) and a control group (traditional instruction). Students in the PBLI group engaged in team-based project learning, receiving real-time feedback and tailored resources through the intelligent platform. The control group followed traditional lecture-based methods. Data collected included online study time, task completion rates, resource access frequency, project scores, anxiety ratings, and learning motivation satisfaction.

**Results:** The results demonstrated that the PBLI model significantly improved learning engagement and task quality, with the experimental group showing higher average online study time and resource access frequency compared to the control group. PBLI students also reported lower anxiety levels and higher satisfaction with learning motivation, with scores of 88.2% for learning motivation satisfaction in the PBLI group versus 79.4% in the control group. Additionally, PBLI students showed greater proficiency in practical applications, suggesting that the model effectively enhances programming skills.

**Conclusions:** The PBLI model shows considerable promise in enhancing learning efficiency and reducing anxiety in JAVA programming courses. This study provides valuable insights into the benefits of incorporating mental health support and innovative teaching practices in programming education. PBLI not only serves as a reference for JAVA courses but also offers a potential framework for other technical subjects requiring a balance of cognitive and emotional support.

**Acknowledgements**

This research was supported by the Education Reform Project of Hunan Province (HNJG-2022-0352).

## MHBM24111: NEWS DISCOURSE ANALYSIS OF THE INFLUENCE ON ATHLETES’ MENTAL HEALTH: FROM THE PERSPECTIVE OF LOCAL GRAMMAR

Yi Chen^a^, Xiuwen Li^a^

^a^ *School of Foreign Languages, Changchun University of Technology, Changchun 130000, China.*

**Background:** Building China into a country with strong sports is one of the overall goals of China’s development by 2035. Enhancing China’s international influence and strengthening China’s international discourse and international communication capabilities are also important development directions for China. In the process, athletes take on the heavy lifting. And under the influence of the Sports Power Policy, the society’s attention to athletes is increasing, which leads to the possibility that athletes are under greater psychological pressure and their mental health is affected. In 2023, the International Olympic Committee developed a new “Mental Health Action Plan”, emphasizing that “there is no real health without mental health”. As one of the important tools to objectively describe information, news discourse aims to achieve a certain communication effect. So sports news discourse can affect the public opinion and should be analyzed. At the same time, with the continuous development of language research and technology, corpus-based research has gradually become a mainstream trend.

**Subjects and Methods:** To analyze the impact of sports news discourse on athletes’ mental health, this paper selects 25 sports news articles from People’s Daily Online to build a “People’s Daily Corpus”, retrieving evaluation discourse in it by using AntConc. In this paper, qualitative analysis is used to analyze the impact on athletes’ mental health, and quantitative analysis is used to analyze the characteristics of evaluation discourse in sports news from the perspective of local grammar.

**Results:** The results show that “Thing Evaluated + Hing + Evaluation + Action” and “Thing Evaluated + Hing + Evaluation + Evaluative Category” are the two most commonly used local grammatical patterns. Combined with the structure, meaning and function, it is found that the evaluation discourse of sports news has the characteristics of objectivity, rigor and conciseness. Only one of the example evaluates the performance of athletes with positive attitude, reflecting the awareness of protecting the mental health of athletes in terms of word selection and content.

**Conclusions:** In conclusion, evaluation discourse in sports news plays a role in guiding social public opinion, and to a certain extent, it has become one of the factors that affect the public’s attitude towards athletes and the mental health of athletes. The sports news discourse should pay attention to both the influence of negative discourse and positive discourse on athletes’ mental health.

**Acknowledgements**

This work was supported by a project grant from Humanities and Social Sciences Research Project of The Education Department of Jilin Province (JJKH20210772SK).

## MHBM24112: THE IMPACT OF NEIGHBORHOOD FEEDBACK ON ENERGY CONSUMPTION BEHAVIOR: THE MODERATING ROLE OF ENVIRONMENTAL ATTITUDE AND EMOTIONAL WELL-BEING, AND THE MEDIATING ROLE OF PERCEPTION OF SOCIAL NORMS

Yihang Lyu^a^, Jingjing Kong^a^

^a^ *Chengdu Neusoft University, Chengdu, 610041, China.*

**Background:** Energy consumption and conservation are critical global challenges, with rapid population growth and economic development contributing to environmental degradation. One promising approach to mitigating energy use is leveraging neighborhood feedback to influence individual behavior. By comparing residents’ energy consumption with that of their neighbors, neighborhood feedback can activate social comparison and norm conformity, encouraging energy-saving behaviors. Despite significant research, the underlying mechanisms of this influence, particularly the roles of social norms, environmental attitude, and emotional well-being, remain insufficiently explored. Emotional well-being, including factors such as happiness and anxiety, may influence how individuals respond to feedback, as those with higher emotional well-being are more likely to adopt sustainable behaviors.

**Subjects and Methods:** This study employs a quantitative research approach, collecting data via survey questionnaires to investigate the relationships between neighborhood feedback, perception of social norms, environmental attitude, emotional well-being, and energy consumption behavior. A stratified random sample of 324 urban households was used, ensuring gender, age, income, and education diversity. Standardized scales validated by previous empirical research were applied to measure all key variables. Statistical analyses, including mediation and moderation tests, were conducted using the SPSSAU platform to explore how neighborhood feedback influences energy consumption behavior through social norm perception, while also considering the moderating effects of environmental attitude and emotional well-being.

**Results:** The results confirmed that neighborhood feedback positively impacts energy consumption behavior, mediated by an enhanced perception of social norms. When individuals perceive a gap between their behavior and social expectations, they are more likely to adopt energy-saving actions. Environmental attitude significantly moderated the relationship between neighborhood feedback and energy consumption behavior; individuals with stronger environmental attitudes were more responsive to feedback and demonstrated greater energy conservation. Additionally, emotional well-being emerged as a critical moderating factor, with individuals experiencing higher levels of emotional well-being exhibiting a stronger response to neighborhood feedback and engaging in more proactive energy-saving behaviors.

**Conclusions:** This study deepens the understanding of how social norms and individual attitudes interact to influence energy-saving behaviors. Neighborhood feedback is validated as an effective tool for promoting energy conservation, particularly among individuals with strong environmental attitudes and higher emotional well-being. These findings provide valuable insights for designing community-level energy-saving interventions. Future policy measures should incorporate neighborhood feedback mechanisms, environmental education, and initiatives to enhance emotional well-being, fostering broader adoption of sustainable energy practices.

**Acknowledgments**

This project was supported by the Center for Agricultural Modernization and Rural Revitalization Research in 2024, Project No.: AMRR2024011.

## MHBM24113: PSYCHOLOGICAL ANALYSIS OF AUDIENCE COMMUNICATION IN THE NEW MEDIA ERA

Li Cui ^a^

^a^ *School of Culture and Tourism, Kaifeng University, Kaifeng, Henan, China.*

**Background:** The advent of the new media era has transformed the shortcomings of mass media’s information monopoly, while also heralding a generational shift in communication and information acquisition methods. In this information age, where new and old media converge, new media platforms offer personalized content to users. This experience can alleviate loneliness and enhance self-identity, thereby positively impacting mental health. The spread of uplifting videos can assist viewers in mitigating negative emotions such as anxiety and depression. By grasping the psychological communication traits of the audience, media professionals can steer the information dissemination trend, earn the audience’s affection and acknowledgment, and ultimately better serve society, contributing to the dissemination of social and cultural values.

**Subjects and Methods:** Research target: Audience of Webcast: as a new Internet product, webcast attracts a large number of users. Studying the audience psychology heath of webcast can help understand the reasons for its popularity and predict its future development trend. Audience of social media: social media such as microblog and Wechat occupy an important position in the new media era. Studying the audience psychology heath of these platforms can help understand their user behavior and platform development strategies. The research methods mainly include the following: questionnaire survey method, interview method, observation method and experimental method. These methods can be used alone or in combination to comprehensively and deeply study the psychological characteristics and behavior patterns of the audience in the new media era. In the information age of the integration of old and new media, mastering the communication psychological characteristics of the audience is essential for media professionals to guide the trend of information dissemination.

**Results:** Through the analysis of the audience’s mental health in the new media era, this paper aims to provide strong theoretical support and empirical basis for the practice of new media. Through in-depth understanding of the psychological needs and behavior patterns of the audience, media practitioners can more effectively formulate communication strategies and improve the communication effect.

**Conclusions:** We should recognize the negative impact of social media on the audience’s mental health, and actively take measures to cultivate the audience’s diversified interests and hobbies, and maximize its positive role. Through rational use of social media and comprehensive cultivation of teenagers’ mental health, they can better adapt to this information-based world. Through psychological analysis of the audience in the new media era, we hope that by improving reporting methods, refining reporting structures, and standardizing reporting content, new media can better cater to the needs of the broad audience. Furthermore, it lays a solid foundation for the further development of the omnimedia era.

## MHBM24114: HARNESSING THE MENTAL HEALTH POTENTIAL OF ROMANTIC ART SONGS: A STUDY OF SCHUBERT’S DER KÖNIG IN THULE AND LISZT’S ES WAR EIN KÖNIG IN THULE

Zhuo Jiang ^a^

^a^ *Lanzhou University of Arts and Science, Lanzhou, Gansu, China.*

**Objective:** This study investigates the mental health applications of Romantic art songs by analyzing Franz Schubert’s Der König in Thule and Franz Liszt’s Es war ein König in Thule. The research explores how compositional elements—melody, harmony, and piano accompaniment—affect emotional regulation, stress relief, and cognitive engagement, with an emphasis on their therapeutic potential for enhancing mental well-being.

**Methods:** A controlled listening experiment was conducted with 50 participants (25 musicians and 25 non-musicians) in a quiet, stress-free environment. Each participant listened to the two compositions and completed a structured questionnaire evaluating their emotional responses (e.g., sadness, relaxation, or tension) and mental health-related outcomes (e.g., emotional release, mindfulness, and focus). The data were analyzed to assess differences in the perceived benefits of each piece.

**Results:** Schubert’s Der König in Thule was associated with feelings of calmness and introspection, offering emotional solace and relief from stress. Participants described the composition as conducive to mindfulness and emotional reflection. Liszt’s Es war ein König in Thule elicited heightened emotional intensity, including awe and tension, which participants identified as mentally stimulating and resilience-building. Schubert’s work was noted for its capacity to promote relaxation and emotional clarity, making it suitable for interventions targeting stress and anxiety. Liszt’s dynamic and intricate composition encouraged cognitive focus and emotional empowerment, potentially aiding individuals in processing complex emotions or improving attention.

**Conclusion:** This study demonstrates the potential of Romantic art songs as tools for mental health support. Schubert’s soothing simplicity provides an avenue for emotional regulation and mindfulness, while Liszt’s dramatic expressiveness supports resilience and cognitive engagement. These findings suggest that integrating art songs into therapeutic practices could enrich mental health interventions, warranting further exploration into the intersection of music, psychology, and well-being.

**Acknowledgments**

This work was supported by the Doctoral Scientific Research Start-up Fund Project of Lanzhou University of Arts and Science, Project Number 2023QDJ09, titled “Innovative Practice Research on Music Teaching Models Based on Multicultural Backgrounds”.

## MHBM24115: PSO-BP NEURAL NETWORK INTEGRATED WITH MULTISOURCE DATA FUSION: A NEW APPROACH TO ASSESSING PSYCHOLOGICAL STRESS IN COLLEGE STUDENTS

Shaohong Chen^a^, Shaoyong Hong^b^, Jincheng Shi^b^, Lingling Dai^c^

^a^ *School of Accounting, Guangzhou Huashang College, Guangzhou 511300, China,* ^b^ *School of Artificial Intelligence, Guangzhou Huashang College, Guangzhou 511300, China,* ^c^ *School of Teacher Education, Guangzhou Huashang College, Guangzhou 511300, China*

**Objective:** With the acceleration of modern society’s pace, the psychological stress experienced by college students has increasingly become a focus of attention. Traditional methods of psychological stress assessment have certain limitations, while emerging methods based on information technology show great potential. The objective of this study is to construct and validate a multisource data fusion model based on the PSO-BP neural network for assessing the psychological stress of college students. It is anticipated that this model will offer a more precise and comprehensive assessment of psychological stress than existing methods and provide decision support for psychological intervention and health promotion.

**Subjects and Methods:** The subjects of this study’s data collection are undergraduate students from a comprehensive university, involving a total of 1068 students. Data preprocessing steps, including cleaning, standardization, and normalization, were undertaken to ensure data quality. The PSO algorithm was employed to optimize the weights and biases of the BP neural network, thereby enhancing the model’s predictive accuracy and generalization ability. Subsequently, a multisource data fusion model was constructed, and its effectiveness in understanding and predicting students’ mental health status was verified through empirical research.

**Results:** The evaluation results demonstrate that the model exhibits high predictive accuracy and stability on the training set, cross-validation set, and test set, with the coefficient of determination (R²) close to 0.9, indicating that the model can explain most of the variability in the data. Furthermore, this study conducted an in-depth analysis of the main factors affecting college students’ psychological stress and found that academic pressure, family factors, and social relationships are the primary influencing factors.

**Conclusions:** The model proposed in this study is not only theoretically innovative but also has great potential for practical application, providing a new scientific tool for the psychological health assessment of college students. Although the universality of the sample is limited, and future research needs to further explore the model’s generalization ability and applicability, overall, the model proposed in this study provides an effective scientific tool for the psychological health assessment of college students. It aids in providing necessary psychological support and intervention in a timely manner, promoting students’ psychological health and academic development.

**Acknowledgements**

This work was supported by a project grant from the Guangdong Province 2023 Educational Science Planning Project (Moral Education Special Project) titled “Undergraduate Psychological Stress Assessment and Analysis Based on Data Mining” (Grant No. 2023JKDY049). This work was also supported by a project grant from the 2022 Guangzhou Huashang College In-House Mentorship Research Project titled “Research on the Psychological Stress Assessment of College Students Based on Data Mining Technology” (Grant No. 2022HSDS24).

## MHBM24116: DIGITAL INTELLIGENCE IN BIOMEDICAL INCUBATION: ENABLING DATA-DRIVEN HEALTHCARE AND PRECISION MEDICINE ECOSYSTEMS

Shanting Wei ^a^

^a^ *Department of Business, Henan University, Kaifeng, 475004, China.*

**Background:** Professional incubation platforms are vital for advancing core technologies in personalized and data-driven healthcare, aligning with the national goal of fostering an innovative and technologically advanced society. Despite their significance, the academic exploration of value-creation mechanisms within these platforms, particularly in the context of digital transformation, remains under-explored. In the burgeoning digital economy, it is crucial to understand how digital intelligence can drive innovation and entrepreneurship across the entire healthcare and pharmaceutical value chain, from research and development to manufacturing and precision health management.

**Subjects and Methods:** This study uses an exploratory case study approach, focusing on the BioSciKin platform, a specialized biomedical enterprise incubation platform. The research examines the integration of digital intelligence technologies into the entire incubation management and ecosystem construction, covering the full spectrum of the healthcare and pharmaceutical processes. Data was collected through semi-structured interviews, document analysis, and direct observation, providing a rich, contextual understanding of the platform’s operations and the impact of digital technologies.

**Results:** BioSciKin has implemented a comprehensive, digitally-enabled service system, including advanced technology platforms, project think tanks, and industrial information platforms. These components enhance resource access, refine project selection, and optimize service delivery. The platform leverages big data and artificial intelligence to streamline drug discovery, improve clinical trial design, and enhance manufacturing efficiency. It also supports the development of personalized treatment plans and precision health management, ensuring tailored and effective patient care. This has led to the establishment of a specialized incubation ecosystem, significantly accelerating the translation and commercialization of biomedical and precision health innovations.

**Conclusions:** This research enhances the theoretical understanding of value creation within incubation platform ecosystems, particularly in the realm of data-driven and personalized healthcare. It provides practical recommendations for improving operational efficiency and fostering value co-creation among stakeholders. BioSciKin exemplifies the significant potential of digital intelligence in creating a dynamic and sustainable innovation ecosystem across the healthcare and pharmaceutical value chain, from R&D to precision health management. The platform’s strategies and outcomes offer a blueprint for leveraging digital transformation to foster innovation in healthcare incubation, underscoring the importance of mental health in maintaining a robust and adaptable workforce.

**Acknowledgements**

This work was supported by a project grant from China Social Science Foundation Youth Project (Grant No. 24CGL005) and Henan University Undergraduate Teaching Reform Research and Practice Project (Grant No. HDXJJG2023-068).

## MHBM24117: EXPLORING THE ROLE OF OPEN INNOVATION AND KNOWLEDGE MANAGEMENT CAPABILITIES IN IMPROVING THE INNOVATION PERFORMANCE OF SCIENCE AND TECHNOLOGY-BASED SMES: THE INFLUENCE OF WORKPLACE ANXIETY

Jie Wu^a^, Yaozhen Chen^a^

^a^ *Zhengzhou University of Industrial Technology, Zhengzhou, 451150, China*

**Background:** Open innovation and knowledge management capabilities are essential for driving innovation performance in small and medium-sized enterprises (SMEs). However, the impact of psychological factors like workplace anxiety on these capabilities is less understood. In resource-constrained environments, anxiety can influence employee productivity and collaboration, which are critical for innovation. This study explores how open innovation, knowledge management, and workplace anxiety interact to impact the innovation performance of SMEs.

**Subjects and Methods:** The study surveyed 100 SMEs in the Suzhou High-tech Development Zone in China, known for its focus on technological advancement. The survey assessed open innovation practices, knowledge management capabilities, workplace anxiety levels, and innovation performance. Additionally, environmental dynamics—such as market and technological changes—were considered to evaluate their moderating impact on knowledge management and innovation. Partial Least Squares Structural Equation Modeling (PLS-SEM) was used to analyze relationships among these variables.

**Results:** Results indicate that both open innovation and knowledge management significantly enhance innovation performance. SMEs that foster external collaboration and knowledge-sharing report higher performance. Workplace anxiety was found to have a dual effect: mild anxiety appeared to motivate employees, increasing engagement in innovation activities, while high anxiety impeded collaborative practices, thus limiting innovation. Environmental dynamics did not directly affect innovation performance but negatively moderated knowledge management capabilities. In highly dynamic environments, workplace anxiety further disrupted knowledge-sharing, thus reducing innovation effectiveness.

**Conclusions:** This study underscores the importance of open innovation and knowledge management in driving SME innovation and highlights the need for managing workplace anxiety to optimize these capabilities. SMEs should invest not only in knowledge-sharing and innovation practices but also in mental health support to manage anxiety levels. The findings provide actionable insights for SME leaders to incorporate mental health strategies into innovation processes. By balancing mental health considerations with innovation initiatives, SMEs can foster an environment that enhances engagement, strengthens adaptability to change, and sustains innovation performance.

## MHBM24118: DESIGN AND IMPLEMENTATION OF AN INTERNATIONAL EDUCATION BILINGUAL INTERACTION PLATFORM BASED ON SPRING BOOT FRAMEWORK AND PRINCIPLES OF MENTAL HEALTH: TAKE ZHENJIANG VOCATIONAL TECHNICAL COLLEGE IVECA COMMUNITY PLATFORM CONSTRUCTION AS AN EXAMPLE

Jun Chen^a^, Jing Chen^a^

^a^ *Department of Information Engineering, Zhenjiang Branch of Jiangsu Union Technical Institute Zhenjiang, China.*

**Background:** This paper focuses on the design and implementation of an international education bilingual interaction platform based on the Spring Boot framework. With the process of globalization, the demand for international education is increasing rapidly, and traditional educational methods are no longer able to meet this demand. we designed and implemented a bilingual interaction platform for international education based on the Spring Boot framework, aiming to provide a convenient and efficient learning environment to meet the learning needs of different students. During the design process, we took into full consideration the mental health issues of students.

**Subjects and Methods:** Firstly, we conducted an in-depth study of the psychological state of students, including their psychological needs, anxiety levels, personality traits, etc. We found that students may encounter various psychological problems during the learning process, such as high learning pressure, severe anxiety, complex interpersonal relationships, etc. These problems not only affect the learning effectiveness of students but also pose a threat to their mental health. To address these issues, we added some features related to mental health to the platform. For example, we provided a counseling service module through which students can seek help from professional counselors. In addition, we also provided a mental health education module through which students can learn some knowledge about mental health and improve their psychological quality. In the implementation process, we adopted the Spring Boot framework. Spring Boot is an open-source Java framework that can simplify the initial setup and development process of Spring applications, allowing developers to focus more on the development of business logic. We utilized the auto-configuration, standalone running, and production readiness features of Spring Boot to quickly build a stable and efficient bilingual interaction platform.

**Results:** Our platform has a good user experience and high usability. The user interface is simple and clear, and the operation process is straightforward, making it easy for students to use the platform for learning. At the same time, our platform also has strong data processing capabilities and can handle a large number of concurrent requests to ensure the stable operation of the platform. In summary, our bilingual interaction platform not only provides a convenient learning environment but also provides protection for students’ mental health.

**Conclusions:** The construction of the platform is a dynamic process that requires comprehensive consideration of current practical needs and students’ interests. We should strive to improve, deepen and enhance the platform, consider the problem from the user’s point of view, face the challenge with the perspective of development, apply the theory to practice, learn from the experienced platform, and achieve the goal of continuous improvement and development. Continue to optimize the functionality of the platform and improve its performance to meet the needs of more users. At the same time, we will further study students’ mental health issues and provide more help and support.

## MHBM24119: BALANCING AI ALGORITHM TRANSPARENCY AND AGILE GOVERNANCE IN MENTAL HEALTH SERVICES

Zhongwei Mei ^a^

^a^ *Southeast University, Nanjing, 210096, China.*

**Background:** With the widespread application of artificial intelligence (AI) technology in public governance, particularly in mental health services, the issue of AI transparency has increasingly become a focal point. Agile governance emphasizes rapid decision-making and flexible adaptation to address pressing mental health challenges such as anxiety, depression, and psychological well-being. However, the complexity of AI algorithms often reduces transparency, leading to public distrust of “black-box” decision-making, especially in sensitive areas like mental health where trust and psychological needs are paramount.

**Subjects and Methods:** Based on complex adaptive systems theory, this paper analyzes the inherent conflict between AI algorithm transparency and agile governance within the context of mental health governance. We examine the core elements of AI algorithm transparency—explainability, auditability, and traceability—and how they impact psychological trust in AI-driven mental health services. Through a review of existing literature and analysis of real-world cases, we propose a layered transparency mechanism and a dynamic feedback system designed to maintain governance efficiency while addressing psychological concerns related to AI transparency.

**Results:** Our findings indicate that while agile governance can significantly enhance the responsiveness and flexibility of mental health services, the opacity of complex AI algorithms can exacerbate feelings of anxiety and distrust among service users. By implementing a layered transparency mechanism, transparency requirements can be adjusted based on the risk levels and psychological sensitivity of different mental health applications. The dynamic feedback system allows for continuous adaptation and improvement of AI transparency, thereby enhancing psychological well-being and trust in AI-driven mental health interventions.

**Conclusions:** Balancing AI algorithm transparency and agile governance in mental health services is crucial for improving psychological trust and the effectiveness of AI applications in this sensitive field. The proposed strategies offer a viable path to reconcile the conflict between the need for rapid, flexible governance and the demand for transparency to alleviate psychological concerns. Future research should focus on developing standardized transparency frameworks and exploring the psychological impacts of AI transparency in mental health governance.

## MHBM24120: CORRELATION ANALYSIS OF CAREER ADAPTABILITY AND PROACTIVE PERSONALITY TRAITS AMONG NURSING INTERNS

Xinxin Liu^a^, Ning Xin^b^, Qian Wang^a^, Yiwei Zhang^a^, Yan Li^a^, Wen Liu^a^, Hong Xia^a^

^a^ *Medical School, Jingchu University of Technology, Jingmen, 448000, China,* ^b^ *Qingdao Hengxing University, Qingdao, 266100, China.*

XL and NX contributed equally to this study.

**Objective:** This study focuses on the group of nursing students interning in a tertiary-level hospital. It aims to deeply and comprehensively analyze the specific situation of the career adaptability and proactive personality characteristics of the intern nursing students, and to explore the differences between the two in detail. Internal correlation, and further explore the impact of career adaptability on career choice, thereby providing a strong reference for the optimization and decision-making of nursing education.

**Methods:** This study adopted a multi-stage stratified sampling method, and selected 181 nursing students who were interning in a tertiary-level A hospital from January to March 2024 as the research subjects. The occupational adaptability questionnaire and proactive personality questionnaire were used for comprehensive and in-depth investigation and research.

**Results:** The results showed that the mean career adaptability score of 181 intern nursing students was (82.64±14.70), and the mean proactive personality score was (34.03±7.74). Among them, factors such as being an only child or not, nursing professional identity status, and having a job offer have a significant impact on the career adaptability level of intern nursing students (P<0.05). At the same time, the total career adaptability score was significantly positively correlated with personality characteristics (P<0.05).

**Conclusions:** Research shows that interns’ career adaptability scores are unsatisfactory, are affected by a variety of factors, and are positively correlated with personality traits. Higher career adaptability helps nursing students better cope with the uncertainty in the career selection process, and can more calmly evaluate their suitability for different nursing positions, thereby making a career choice that better suits their own development needs. decision making. On the contrary, insufficient career adaptability may cause nursing students to face more difficulties in career choices, and they are prone to confusion and following trends, which will affect the stability and satisfaction of their career development. Nursing schools should fully realize this, provide nursing students with all-round social support from the physical and psychological levels, focus on improving their career adaptability skills, help nursing students take the initiative in career choices, and achieve healthy planning for career development.

**Acknowledgements**

This research was funded by the 2024 Scientific Research Project of the 14th Five-Year Plan for Educational Science of Jingmen City (No.: JMG2024008), the Science and Technology Project of Jingchu Institute of Technology (No.: QN202420), and the Humanities and Social Sciences Research Project of the Provincial Department of Education (No.: 23z623), and the Special Research Project on Integrated Thought Education for Primary, Secondary and Higher Education in Jingmen City (No.: DSZJS24028).

## MHBM24121: RESEARCH ON THE CONNOTATION AND PRACTICE PATH OF COMMON PROSPERITY BASED ON PEOPLE’S HAPPINESS

Jingang Song ^a^

^a^ *Northeastern University, Shenyang 110169, China.*

**Background:** In the current critical period of social and economic development, common prosperity, as an essential requirement of socialism, has become an important goal of our country development. With the improvement of people’s living standards, happiness has gradually become an important indicator of social development. This study aims to explore the connotation and practice path of common prosperity based on people’s happiness, which has theoretical and practical significance for promoting comprehensive social progress and realizing people’s longing for a better life.

**Subjects and Methods:** This article adopts the method of literature review, theoretical, and case study. By combing through relevant domestic and international literature, the theoretical foundation of common prosperity is clarified; through theoretical analysis, the intrinsic connection between’s happiness, economic development level, social fairness, and common prosperity is deeply analyzed; through case studies, the practice path of common prosperity in different regions and fields explored.

**Results:** Common prosperity refers not only to the common growth of material wealth but also to the enrichment of spiritual culture and the sustainable improvement of the environment. Among these, the happiness of the people is an important measure of common prosperity, reflecting the comprehensiveness and coordination of social development. Through empirical analysis this study proposes a practical path to common prosperity based on the happiness of the people. Specifically, this includes: optimizing the income distribution system to narrow the gap between rich and the poor; strengthening the construction of the social security system to enhance the welfare of the people; promoting the balanced development of public services such as education and to improve the quality of life; promoting cultural prosperity to meet the spiritual and cultural needs of the people; strengthening ecological and environmental protection to achieve harmony between man and. There is a significant positive correlation between the happiness of the people and the level of common prosperity. Enhancing the happiness of the people is an important path necessary condition for achieving common prosperity. Meanwhile, the practice of common prosperity requires the joint efforts of the government, the market, and society, forming a synergy to comprehensive social progress.

**Conclusions:** This study always takes the people’s well-being as the core concern, and through multi-dimensional and multi-level analysis, it reveals the intrinsic connection between-being and common prosperity. The research results show that the improvement of is an important manifestation of common prosperity and a key indicator to measure the effectiveness of social development. This study has significant theoretical guiding significance and practical value for promoting practice of common prosperity and improving people’s happiness, providing a scientific basis and reference for future policy-making.

## MHBM24122: A STUDY ON THE CURRENT WATER RESOURCE SITUATION AND STRATEGIES FOR ALLEVIATING ECOLOGICAL WATER ANXIETY AMONG RESIDENTS IN ARID AREAS

Youxi Yan^a^, Qingchun Fang^b^, Wenqi Gong^b^

^a^ *School of Economics and Business Administration, Yibin University, Yibin, Sichuan 644000, China,* ^b^ *Wuwei Productivity Promotion Center, Wuwei, Gansu 733000, China,* ^c^ *Department of Resources and Environment, Wuhan University of Technology, Wuhan, Hubei 430070, China.*

**Background:** Amidst the escalating global water crisis, the scarcity of water resources in arid regions significantly affects local living conditions, causing psychological anxiety among residents. To manage the limited water resources effectively and relieve residents’ ecological water concerns, continuous sustainable development of settlement systems in arid regions is essential. Ensuring the continuous sustainable development of settlement systems in arid regions is not only crucial for the environment but also for the mental and emotional health of the inhabitants.

**Subjects and Methods:** This study examines Minqin County in the arid northwest, characterized by its unique settlement system. Through field surveys and data analysis, the state of water resources is assessed, and the causes of residents’ ecological water anxiety are identified. The study proposes using the natural course of the Daxi River to deliver water to Qingtu Lake, forming a river-lake connectivity strategy. An ecological water demand prediction model is also established to assess the feasibility of this innovative approach.

**Results:** The total water consumption in Minqin County reaches 386.6 million m3 annually. Currently, 30.59 million m3 of ecological water are supplied to Qingtu Lake from the Hongyashan Reservoir through artificial channels. This results in an imbalanced “heavy at the ends, light in the middle” model, falling short of residents’ expectations for protection against sandstorms, leading to anxiety. It is estimated that the Daxi River could provide 94.26 million m3 annually, an increase of 63.67 million m3, adequate for the total annual water supply needs.

**Conclusion:** The prevailing psychological anxiety among residents of Minqin County, stemming from the ongoing water scarcity, underscores the urgency of addressing ecological water concerns. Based on the current total water usage within the Minqin settlement system and the existing ecological water input to Qingtu Lake, implementing a water transfer plan along the northwest line of the settlement “trunk” via the natural river channel of the Daxi River to achieve river-lake connectivity presents a practical and feasible ecological water strategy that not only meets the water needs but also alleviates the psychological distress of the residents. This approach can establish a natural ecological vegetation barrier along the northwest line of the settlement “trunk”, effectively addressing residents’ concerns over ecological water needs.

**Acknowledgements**

This work was supported by a project grant from General Project of Ministry of Education Foundation on Humanities and Social Sciences in 2021(Grant No. 21XJA630008), Key Laboratory of Urban-Rural Industrial Integration and Intelligent Decision-Making, Sichuan Province For Social Sciences, Water Transport Economic Research Center and Upper Yangtze River Shipping and Logistics Collaborative Innovation Center.

## MHBM24123: CONSTRUCTION OF ART ELEMENT LIBRARY OF “KU SHULAN PAPER-CUT” AND ITS PSYCHOLOGICAL EMPOWERING APPLICATION IN TEACHING

Lingwei Zhu ^a,b^

^a^ *Xi’an Eurasia University, Xi’an, Shaanxi, China,* ^b^ *Faculty of Education, Srinakharinwirot University, Thailand*.

**Background:** Over the past decade, research on the art of Ku Shulan paper-cutting has largely been confined to traditional domains. However, the exploration of its application in digital education and mental health has been scarce to an extreme degree. Under the background of modern education, the mental health of students, especially in the process of creation, has attracted more and more attention. Constructing an art elements library of Ku Shulan paper-cutting is expected to enhance students’ creative capabilities, fulfill their psychological demands, and offer provide support for their mental health.

**Objective and Methods:** The study adopts an unconventional approach by clearly defining relevant concepts and offering support with the PDCA (Plan-Do-Check-Act) cycle theory as the framework. The paper elaborates upon the methods for creating the “Ku Shlan” art element library and its application in animation courses, with the aim of facilitating flexible extraction and utilization of the elements. In this process, the study places a significant emphasis on fulfilling the students’ creative psychological needs, such as the need for self-expression, anxiety alleviation, and emotional release, etc.

**Results:** The creation of this library of artistic elements has been shown to enhance students’ creative abilities, helping them better express their personal emotions and enhance their self-confidence. By satisfying students’ psychological needs in the creative process, students can gain better emotional experience and improve mental health. The establishment of this art element library has been shown to enhance students’ creative capabilities, facilitating their superior expression of personal emotions and consolidation of self-confidence. Through fulfilling the psychological requirements of students during the creative process, students can acquire more favorable emotional experiences and improve their mental health.

**Conclusion:** The paper advocates the incorporation of the art elements of Ku Shulan into modern educational practices as a strategy to comprehensively fulfill the students’ creative psychological needs. This integration is not merely critical for facilitating the students’ creative growth but also holds substantial implications for enhancing their overall psychological well-being, stabilizing their emotions, and augmenting their sense of happiness. It is proposed that such measures are capable of making an outstanding contribution to the students’ overall development and healthy emotional growth.

**Acknowledgments**

Annual Project of Shaanxi Provincial Social Science Fund-Research on the Creation and Application of the “Ku Shulan Paper-cutting” Art Element Library (2023J040).

## MHBM24124: RESEARCH ON THE OPTIMIZATION OF TEACHER SUPPLEMENT MECHANISM IN GUANGXI PUBLIC KINDERGARTENS FROM THE PERSPECTIVE OF MENTAL HEALTH

Anyi Yang^a^, Jiachao Wei^b^

^a^ *Nanchong Cultural Tourism Vocational College, Langzhong, 637400, China,* ^b^ *Nanning Normal University, Nanning, 530299, China.*

**Background:** Preschool teachers are indispensable human resources for kindergartens and an important guarantee for high-quality preschool education. In the context of educational psychology, paying attention to the mental health of preschool teachers and optimizing the teacher supplement mechanism is not only an important part of strengthening the construction of public kindergarten teachers, but also crucial for maintaining a healthy and effective learning environment. The mental health of educators directly impacts their ability to provide quality education and foster a nurturing atmosphere conducive to the developmental needs of young children.

**Subjects and Methods:** This study takes public kindergartens in urban areas of Guangxi, China as an example, and randomly selects 20 public kindergartens as sample kindergartens for empirical research. Select full-time teachers and principals from 20 public kindergartens, as well as administrative department heads from Guangxi Education Bureau, as research subjects. Through questionnaire surveys and interviews, the operational effectiveness of the supplementary mechanism for public kindergarten teachers was understood from the perspective of mental health. Analyzed the problems and reasons for the teacher supplement mechanism in public kindergartens, with a focus on the psychological needs of kindergarten teachers, and provided a basis and suggestions for optimizing the teacher supplement mechanism in public kindergartens.

**Results:** Research has found that from the perspective of mental health, there are three gaps in the current situation of teacher supplementation mechanisms in public kindergartens in urban areas of Guangxi: firstly, the number is insufficient to meet the needs of teachers; Secondly, there is an imbalance in quality, making it difficult to ensure team stability; Thirdly, inadequate guarantees make it difficult to attract high-quality talents.

**Conclusions:** From the perspective of mental health, optimizing the supplementary mechanism for public kindergarten teachers can be carried out from three aspects: first, improving the organizational mechanism, refining the quantity requirements, and paying attention to the psychological needs of kindergarten teachers; The second is to strictly control the operation, optimize quality standards, and accelerate the psychological adaptation of kindergarten teachers; The third is to ensure the guarantee mechanism, strengthen incentives, and enhance the personal identity and sense of belonging of kindergarten teachers.

## MHBM24125: INNOVATIVE DESIGN OF CAMPUS TRANSPORT VEHICLE UNDER THE ORIENTATION OF PSYCHOLOGICAL EXPERIENCE NEEDS

Hao Huang^a,b^, Hua Zhang^a,b^

^a^ *Hunan University of Technology, Zhuzhou 412007, China,* ^b^ *Hunan Key Laboratory of Advanced Technology and Future Design, Zhuzhou 412007, China*

**Background:** This study aims to enhance campus transportation services at Hunan University of Technology by integrating a design approach based on behavioral experience, focusing on improving both the quality of interaction and the psychological well-being of users. By addressing factors such as anxiety, sensitivity to crowded environments, self-esteem, and emotional comfort, the study seeks to improve user satisfaction and create a supportive, less stressful transportation experience. Understanding the mental health aspects of user behavior is key to optimizing service quality, especially in an environment where students’ psychological needs play a critical role in their daily interactions with campus services.

**Subjects and Methods:** A ‘bottom-up’ feed-forward mechanism and a ‘top-down’ FBS (Function-Behavior-Structure) model were adopted to better capture and analyze the biases in user experience and their psychological needs. These methodologies were applied alongside the TOPSIS decision-making method to determine the most appropriate design direction for the campus transportation system. Key design aspects such as passenger capacity, vehicle size, and spatial layout were considered, with an emphasis on reducing anxiety and creating an emotionally supportive environment for users. The intervention aimed to not only fulfill functional requirements but also mitigate stressors that may affect users’ emotional states, ensuring a balance between utility and psychological comfort.

**Results:** The combination of the feed-forward mechanism and the FBS model successfully identified user demands and psychological biases, such as the need for personal space, emotional comfort, and stress reduction. This approach proved effective in refining design choices that meet both physical and psychological needs. The study revealed that by addressing users’ emotional well-being, including minimizing anxiety in crowded settings, overall satisfaction with the campus transportation system improved. Furthermore, the results provided empirical insights that can inform future design research on integrating psychological health with user experience in public service systems.

**Conclusions:** The integration of a feed-forward mechanism and the FBS model in the design of campus transportation vehicles enables the system to anticipate both functional and psychological demands. This approach not only enhances the practical use of campus transport but also addresses the mental health needs of users, such as reducing anxiety and improving emotional comfort. By incorporating psychological wellness into the design process, the study contributes to a more holistic understanding of user experience, promoting interdisciplinary research that bridges design, psychology, and user-centered service optimization. The findings offer valuable implications for future transportation design and mental health-focused service improvements.

**Acknowledgements**

This work was supported by a project grant from Hunan Province Graduate Research Innovation Project Funding (Grant No.CX20231112) and Project funding from Zhuzhou Academy of Social Sciences (Grant No.ZZSK2024162).

## MHBM24126: RESEARCH ON THE COUPLING AND COORDINATION OF RURAL REVITALIZATION, RURAL ELDERLY CARE, AND PSYCHOLOGICAL NEEDS IMPLEMENTATION

Jijun Zhang^a^, Leixi Cao^a^, Kangning Li^a^

^a^ *Yan’an University, Yan’an, Shaanxi 716000, China.*

**Background:** Physiological and psychological needs are the content of human development. The rural revitalization strategy emphasizes the comprehensive development of rural areas, which can meet the physiological and psychological needs of the people, promote the healthy development of personality, and promote the sustainable development of rural economy.

**Subjects and Methods:** This article takes five provinces and regions in northwest China as research objects, and establishes a measurement index system for the development level of rural revitalization and rural elderly care services based on people’s physiological needs, psychological needs, and personality development. The entropy method is used to measure the development level of both, and the coupling coordination model is used to calculate and analyze the coupling. Targeted countermeasures are proposed to meet people’s psychological and emotional needs, reduce public sensitivity, and promote good personality development.

**Results:** On the one hand, the comprehensive evaluation index of rural revitalization and rural elderly care service development is generally on the rise, meeting people’s psychological needs, narrowing the gap in psychological needs, enriching people’s emotional needs, meeting people’s physical and mental development, and promoting healthy personality development. On the other hand, the development level of rural revitalization and rural elderly care services in the five provinces and regions of Northwest China is characterized by “low in the middle and high on both sides”. People’s psychological and emotional realization also stimulate their self-worth. Although there are differences in psychological needs, the overall satisfaction of people’s inner needs is high, and their psychological recognition and happiness are also higher.

**Conclusions:** Only by continuously improving the development system of rural elderly care services, meeting the psychological needs of the people, reducing their anxiety, cultivating sound personality development, and continuously improving the quality of rural revitalization and rural elderly care service development in weak areas, can we efficiently promote the coupling and coordination of rural revitalization and rural elderly care services, meet the physical and mental care needs of rural elderly people, and improve the quality of people’s happy development.

**Acknowledgements**

This article has been funded by the Science Research Program of Shaanxi Provincial Department of Education (Project No. 22JY068).

## MHBM24127: STRATEGIES FOR ENHANCING INTELLIGENT LABORATORY MANAGEMENT AND BUILDING INNOVATIVE PSYCHOLOGICAL NEEDS WITH INTERNET-BASED THINKING

Zhaoyuan Zhang ^a^

^a^ *Mechanical Engineering College, Xi’an Shiyou University, Xi’an, 710065 China*

**Objective:** As China advances its strategic plan to become a leading innovative nation, it is crucial to elevate technological innovation across all sectors. Laboratories, serving as the cornerstone of scientific research and development, are integral to boosting research efficiency and driving innovation. The modernization of laboratory management is therefore vital for meeting the immediate psychological needs of research personnel. However, traditional laboratory management methods suffer from uneven resource distribution, information asymmetry, and a lack of sensitivity to evolving research patterns. These issues hinder the ability to keep pace with the rapid technological advancements and shifting directions in scientific research.

**Subjects and Methods:** To provide a strong theoretical foundation for the reform of experimental management, this paper employs the management philosophy of “internet thinking” to investigate the application of intelligent technologies in constructing a theory of intelligent laboratory management. It further examines the role of automation technologies in intelligent laboratory management systems to propose strategies for optimizing intelligent management while addressing the innovative psychological needs of researchers.

**Results:** Experimental analysis demonstrates that adopting intelligent and information-driven approaches can transform existing laboratory management paradigms. By optimizing resource allocation at each stage and increasing controllable risk thresholds, we can implement high-standard management strategies. These strategies enhance the safety and stability of the innovation process, support the construction of high-standard psychological environments for innovation, and make scientific research more precise and efficient. Consequently, research personnel can focus more on substantive scientific innovation, and laboratory safety management becomes more effective.

**Conclusions:** In conclusion, integrating internet-based thinking into laboratory management and enhancing the psychological support for innovation are pivotal strategies for dual improvements in research service quality and stability. This approach can significantly elevate laboratory management standards and the capacity for scientific innovation, thereby playing a crucial role in steering China’s scientific endeavors towards greater efficiency, intelligence, and advancement.

**Acknowledgments**

This work was financially supported by National Natural Science Foundation of China (51905426);

Shaanxi Provincial Department of Education Scientific Research Program (23JK0606); Open Fund of the National Oil and Gas Drilling Equipment Engineering Technology Research Center (BOMCOJ118-JKY016-2023)

## MHBM24128: MOTIVE TO MOVE, PSYCHOLOGY ANGUISH AND POST-RESETTLEMENT INCOME RESTORATION: THE EVIDENCE FROM CHINA

Muyao Hao ^a^

^a^ *Department of Business Aadministration, University of Northampton, Singapore 546080.*

**Background:** Prior studies have primarily examined how psychological impacts, such as stress and mental distress, arising from a lack of livelihood capital post-resettlement affect individuals’ well-being. These studies often assume that resettlement in China is typically forced, leading to mental anguish and reduced psychological well-being. However, the Chinese government’s recent poverty alleviation resettlement policies emphasize psychological voluntariness as the first principle, promoting resettlement only when people express a willingness to move. Nevertheless, there remains ambiguity around what constitutes “psychological voluntariness” and how to assess it effectively. This ambiguity in defining voluntariness can lead to policy gaps and challenges in implementation, impacting the mental and emotional well-being of resettled populations. This study aims to examine how psychological motives to relocate in China influence both income levels and mental health outcomes.

**Subjects and Methods:** This study collects data from semi-structured interviews and household surveys conducted in 34 poverty alleviation resettlement villages in western Shanxi and northern Shaanxi provinces, mountainous areas along the Loess Plateau. We focused on capturing the psychological motivations and mental readiness of individuals before and after their resettlement, as well as their involvement in policy-making processes throughout resettlement. Using a regression model, we analyzed three factors—psychological willingness to resettle, livelihood capital, and other control variables—to assess how these factors influence both income and mental health outcomes for resettlers.

**Results:** Findings indicate that resettlement must be psychologically voluntary to support mental well-being effectively. Resettlers exhibited varying degrees of willingness, with those showing higher psychological satisfaction and positive anticipation being more likely to adjust successfully to new environments, change livelihoods willingly, and find sustainable employment. In contrast, individuals forced to relocate often experience mental distress and reluctance, leading to poorer outcomes, as their mental resilience is weakened by the lack of choice. These “reluctant movers” are at greater risk for psychological distress, particularly as essential services are curtailed in their original communities.

**Conclusion:** For individuals with low willingness or experiencing mental distress prior to relocation, post-resettlement income growth remains limited, and natural and physical capital are primary influences on recovery. However, those who anticipate the move positively and express higher psychological willingness show faster income restoration and rely more on social, human, and financial capital. The findings underscore the importance of considering mental health and psychological readiness in resettlement policy to enhance both financial and emotional resilience.

## MHBM24129: OPTIMIZATION OF HIGH-LEVEL TALENT STRUCTURE FROM THE MENTAL HEALTH DIMENSION: DEMAND ANALYSIS UNDER NEW QUALITY PRODUCTIVITY

Yao Zhou ^a^

^a^ *Changzhou College of Information Technology, Changzhou, Jiangsu 213164, China.*

**Background:** In the current era, new quality productivity is witnessing rapid growth, which has brought about profound changes in various aspects of society. Against this backdrop, the structure of high-level talents is facing unprecedented challenges. The evolving work environment under new quality productivity not only requires talents to possess advanced professional skills and excellent qualities but also places higher demands on their mental health. For instance, the continuous emergence of new technologies and rapid market changes mean that talents need to have strong adaptability to cope with uncertainties, sufficient stress resistance to handle high-pressure tasks, and proficient emotion management abilities to maintain good working and interpersonal relationships. Traditional talent structures often fall short in these psychological aspects, making it essential to delve deeper into how to better support and develop the mental health of high-level talents.

**Subjects and Methods:** This research primarily focuses on comprehensively analyzing the specific requirements that new quality productivity imposes on the structure of high-level talents, with particular emphasis on both skills and mental health dimensions. To understand the roles played by education and talent policies in this regard, a multi-faceted approach is adopted. Extensive literature reviews are conducted to gather theoretical foundations and existing research findings related to talent development, mental health, and relevant policies. Meanwhile, numerous case studies of different educational institutions and enterprises are analyzed to observe how education practices and talent policy implementations influence the psychological state and structure of high-level talents in real-world scenarios.

**Results:** The results reveal that new quality productivity indeed demands high-level talents to possess a series of psychological competencies. Their adaptability determines whether they can smoothly keep up with the fast pace of technological and market changes without experiencing excessive anxiety. Strong stress resistance enables them to stay resilient when facing tough work challenges like project failures or tight deadlines. Effective emotion management skills contribute to a harmonious team atmosphere and efficient work progress. In terms of education, it is shown to have a positive impact on enhancing the psychological quality of talents, such as cultivating their self-confidence and emotional stability, and also influences their mental structures by shaping their cognitive patterns and coping mechanisms. Talent policies, on the other hand, can either support or impact the psychological structure of talents, for example, through providing reasonable incentives to boost motivation or causing stress due to improper evaluation systems. Additionally, specific directions for educational reform, practical suggestions for optimizing talent policies, and feasible solutions to psychological challenges emerging during policy implementation are all uncovered from a psychological perspective.

**Conclusions:** To sum up, education and talent policies play a pivotal role in optimizing the structure of high-level talents. Maintaining the mental health of talents is as important as improving their skills and qualities. Only by ensuring their psychological well-being can talents truly exert their potential and enhance their competitiveness. Therefore, it is imperative that all sectors, including the government, educational institutions, and enterprises, collaborate closely to address the existing challenges, thereby promoting high-quality economic and social development and making significant contributions to the great rejuvenation of the Chinese nation.

**Acknowledgements**

This research work was supported by: The 2022 Jiangsu Provincial University Philosophy and Social Science Research Project: Research on the Innovative Construction of Faculty Development Center in Higher Vocational Colleges under the Background of ‘Double High’ Construction (2022SJYB1364). The 2024 Research Project on the Teaching Reform of Information Technology Courses in National Higher Vocational Colleges of the Informatization Teaching Steering Committee of Vocational Colleges under the Ministry of Education: Research on the Cultivation and Training Model for Information Technology Teachers Adapting to the Digitalization of Education (KT2024018). The 2022 Jiangsu Province Education Planning Project: Study on the Construction of Big Data Professional Talent Cultivation System Based on the Integration of Science and Education (B/2022/02/86). The 2023 Research Project on the Reform of Higher Education Teaching in Jiangsu Province: Research on the Evaluation Index System for Specialty Construction in Higher Vocational Colleges under the Background of “Double High” Construction (2023JSJG619).

## MHBM24130: DIFFERENT DEMANDS IN TRADITIONAL CHINESE MEDICINE (TCM) HEALTH SERVICE IN PRIMARY HEALTHCARE INSTITUTIONS AMONG RESIDENTS WITH DIFFERENT GENDER, AGE AND INCOME: A CROSS-SECTIONAL SURVEY CONDUCTED IN HUBEI PROVINCE IN CHINA

Zihan Yang^a^, Changmin Tang^a,b,c^, Na Li^a,b,c^, Lingshan Li^a,b,c^, Ni Yan^a,b,c^, Xin Chen^a^

^a^ *School of Management, Hubei University of Chinese Medicine, Wuhan, Hubei, China,* ^b^ *Hubei Shizhen Laboratory (HSL), Wuhan, Hubei, China,* ^c^ *Key Research Institute of Humanities and Social Sciences of Hubei Province, Wuhan, Hubei, China.*

**Background:** Traditional Chinese Medicine (TCM) plays important role in the healthcare system in China. The development of TCM in primary healthcare institutions could improve the accessibility of TCM health services for residents and promote population health. This study investigated the differing demands for Traditional Chinese Medicine (TCM) health services in primary healthcare institutions among residents in Hubei Province, with a focus on gender, age, and income disparities.

**Subjects and Methods:** A cross-sectional survey was conducted among residents in two cities (Wuhan and Suizhou cities) in Hubei Province. A self-designed questionnaire was used to evaluate the residents’ different demands for various TCM health services in primary healthcare institutions, and a total of 1005 questionnaires were collected effectively. Analysis of variance methods were used to identify significant differences in TCM service demands among different demographic groups and statistical significance was set at p<0.05. Descriptive analysis was used to analyze sociodemographic characteristics and needs assessment of different TCM items among the residents surveyed.

**Results:** Female and older residents had more TCM health services needs in primary healthcare institutions, while middle-income residents (3001-6000 yuan) showed the greatest demand compared to other income groups. In 36 TCM service demand survey, there were significant differences among residents with different genders in 11 TCM health services and therapies, among residents with different ages in 35 TCM health services and therapies, and among residents with different incomes in 27 TCM health services and therapies (p<0.05).

**Conclusion:** The needs of TCM health services in primary healthcare institutions among residents with different social characteristics should be paid attention to accordingly. More consideration needs to be given to the TCM needs of the female, elderly and middle-income groups in order to provide TCM health services that better meet their demands. In order to provide better TCM health service, professional training of TCM practitioners in primary healthcare institutions should be arranged correspondingly. TCM culture should be promoted through lectures, bulletin boards and health activities in primary healthcare institutions to increase awareness and acceptance. Moreover, digital platforms can be used to provide personalized TCM health management according to residents’ specific needs.

**Acknowledgments**

This study was funded by Natural Science Foundation of Hubei Province (2023AFD161); Key Research Institute of Humanities and Social Sciences of Hubei Province, “Research Center for the Development of Traditional Chinese Medicine” Project (ZXYB004); Hubei University of Traditional Chinese Medicine’s “Traditional Chinese Medicine Inheritance and Innovation Plan” project; Excellent Young and Middle aged Science and Technology Innovation Team Program for colleges and universities in Hubei Province (T2023015); Major Special Project funded by Hubei Provincial Social Science Fund (23ZD180).

## MHBM24131: RESEARCH ON EMERGENCY MATERIAL SCHEDULING FOR DANGEROUS HAZARDOUS CHEMICALS STORAGE ACCIDENTS CONSIDERING PSYCHOLOGICAL PAIN OF VICTIMS

Jianfeng Lu ^a^

^a^ *College of Business Administration, Nanchang Institute of Technology, Nanchang, China.*

**Background:** The occurrence of disasters has disrupted the original living environment of disaster victims, broken their physical and psychological sense of security, and caused psychological pain due to the lack of resources. Post disaster emergency material dispatch is crucial for improving disaster response efficiency and reducing disaster losses. In order to reduce the psychological pain of disaster victims in the event of a shortage of Emergency Material, the problem of Emergency Material scheduling for hazardous chemicals storage accidents considering considering psychological pain of victims is studied in this paper.

**Subjects and Methods:** The measurement method of the perceived level of psychological pain among disaster victims in hazardous chemicals storage accidents is proposed. The emergency logistics network structure for hazardous chemical storage accidents has been designed, and the optimization of emergency resource scheduling for hazardous chemical storage has been explained. An optimization model for Emergency Material scheduling in hazardous chemicals storage accidents Considering psychological pain of victims is constructed, aiming to minimize total scheduling cost and psychological pain of victims. The solution method of the model includes two stages. The specific solving algorithm is combined with genetic algorithm to improve the ant colony algorithm. An example is given to demonstrate the effectiveness of the model and the solving method.

**Results:** The case study have shown that the emergency material dispatch model for hazardous chemicals proposed in this article can reduce the psychological pain of victims. In the process of the emergency materials scheduling for hazardous chemicals accidents, the problem that how to minimize total scheduling cost and psychological pain of victims is solved promptly.

**Conclusions:** Adding the objective function of psychological pain of victims to the emergency material dispatch model for hazardous chemicals accidents can reduce reduce the psychological pain of victims and improve their satisfaction with disaster relief. The research conclusion is helpful for emergency management departments to better develop emergency material dispatch plans applicable to dangerous hazardous chemicals storage accidents.

**Acknowledgments**

This research was funded by the Jiangxi Provincial University humanities and social science research and planning project (Grant No.GL22229, GL21126), and the project of high-level talent introduction of the scientific research of Nanchang Institute of Technology (Grant No. 2021kyqd022).

## MHBM24132: A STUDY ON THE RELATIONSHIP BETWEEN TEACHERS AND STUDENTS IN PRIMARY AND SECONDARY SCHOOLS FROM THE PERSPECTIVE OF “DUAL PERSPECTIVES OF TEACHERS AND STUDENTS PSYCHOLOGICAL HEALTH”: SURVEY BASED ON G PROVINCE

Bingquan Jia^a,b^, Ye Zhou^a^

^a^ *Northwest Normal University, Lanzhou, 730070, Gansu, China,* ^b^ *Officers College of PAP, Chengdu, 610213, China.*

**Background:** The teacher-student relationship in primary and secondary schools establishes an emotional connection between students and teachers, which is not only related to classroom experience and academic performance, but also affects students’ psychological health, and is a key element for the high-quality development of basic education. A ‘democratic’ teacher-student relationship can allow students to feel encouraged, supported, understood, and cared for by their teachers, leading to healthy psychological experiences such as confidence, self-improvement, and self-reliance. When transferred to learning, it can generate students’ motivation to explore actively. The “laissez faire”, “confrontational”, and “authoritarian” teacher-student relationships may lead to negative emotions such as frustration and despair among students, and over time, even negative behaviors such as truancy and rebellion may occur. This not only affects the improvement of teaching quality, but also may miss out on many educational opportunities.

**Subjects and Methods:** The relevant research on the types of teacher-student relationships in primary and secondary schools in China is mostly based on the perspective of their respective roles. This study is based on the perspective of teacher-student psychological identification, investigating the degree of “democratic”, “laissez faire”, “confrontational”, and “authoritarian” teacher-student relationships from the dual perspectives of primary and secondary school teachers and students. Based on this, the types of teacher-student relationships are constructed and the psychological reasons for the formation of these types are analyzed. Descriptive statistics and t-tests were conducted on 6271 “student questionnaires” and 901 “teacher questionnaires” regarding the teacher-student relationship in primary and secondary schools in G province.

**Results:** It was found that the ‘democratic type’ is the main type of teacher-student relationship, indicating that positive emotional feedback makes teachers and students feel recognized for each other psychologically, which helps to improve their self-esteem, confidence, and positive emotional experiences. In addition, the proportion of “authoritarian”, “laissez faire”, and “confrontational” types is small, but they also constitute the types of teacher-student relationships in primary and secondary schools, reflecting negative psychological identification problems between teachers and students, manifested as disharmony, disrespect, distrust, poor communication, and insufficient care, which will hinder the high-quality development of basic education.

**Conclusions:** Overall, the teacher-student relationship in primary and secondary schools is positive, which can have a positive impact on their psychological health. However, there is also a low quality of teacher-student interaction in terms of teaching relationships, which reflects poor psychological communication between teachers and students; Insufficient emotional attention in psychological relationships reflects a lack of mutual respect and trust between teachers and students; In terms of ethical relationships, the construction of teacher ethics and style is weak, reflecting a lack of concern and listening in the psychology of teachers and students. Finally, based on the perspective of psychological identity, countermeasures were proposed from three aspects: teaching relationship, psychological relationship, and ethical relationship.

## MHBM24133: THE PSYCHOLOGICAL NEEDS AND CULTURAL HERITAGE PRESERVATION: IMMERSIVE VIRTUAL REALITY EXPERIENCE DESIGN FOR NAXI REMEZHO DANCE

Chenxiao Li^a,b^, Ke Sun^a^, Kun Yu^b^

^a^ *Faculty of Art and Design, Lijiang Culture and Tourism College, 674199 Lijiang, Yunnan, China,* ^b^ *Department of Communication, Faculty of Modern Languages and Communication, Universiti Putra Malaysia, 43400 Serdang, Selangor, Malaysia*

**Background:** With the proliferation and advancement of digital technology, public demand for cultural heritage experiences has gradually shifted from static appreciation to dynamic interaction. In particular, immersive digital experiences that meet deep psychological needs have garnered increasing attention. However, research on how to construct emotional connections, satisfy users’ psychological needs, and enhance cultural transmission through virtual reality technology remains in its infancy, lacking systematic design frameworks and strategies.

**Subjects and Methods:** This study focuses on the dance culture of the Naxi ethnic group, *Re-Me-Zho,* analyzing users’ psychological needs (including emotional resonance, entertainment experience, and cultural identity) to design an immersive virtual reality (IVR) system experience framework. Utilizing highly realistic virtual scenes developed with the Unreal Engine, combined with gesture tracking and positional recognition technologies, the system features multi-layered interactive functionalities. Through narrative design and a segmented story mode, users are guided to explore cultural elements in a virtual ceremonial setting, experiencing the spiritual significance embedded in cultural transmission.

**Results:** Experimental results indicate that the system significantly stimulates users’ interest in cultural heritage and satisfies their psychological needs, particularly excelling in emotional connection and cultural understanding. Users’ sense of immersion and engagement was effectively enhanced, while also increasing their awareness of preserving the *Re-Me-Zho* dance culture of the Naxi ethnic group.

**Conclusions:** This study demonstrates that integrating virtual reality technology with psychological needs provides an innovative solution for the digital preservation of cultural heritage. Emotion-driven design strategies not only improve the dissemination and promotion of cultural heritage but also offer theoretical foundations and practical guidance for future immersive cultural experience designs.

## MHBM24134: THE IMPACT OF THE TRANSFORMATION OF TEACHING ART IN ONLINE EDUCATION ON THE PSYCHOLOGICAL NEEDS OF TEACHERS AND STUDENTS

Jiaxin Liu ^a^

^a^ *Shandong Huayu University of Technology, Dezhou Shandong 253000, China.*

**Background:** Since 2020, online education has emerged as a crucial component of contemporary teaching, catalyzing the integration of online and offline teaching practices. This hybrid approach is increasingly regarded as a normalized trend in the evolution of educational methodologies. However, the traditional concepts and applications of teaching art—an essential element of effective pedagogy—face significant challenges in adapting to the unique demands of the online education environment. These challenges disrupt not only the psychological interaction between teachers and students but also increase stress levels, hinder emotional connection, and fail to address the psychological needs essential for fostering engagement and motivation.

**Subjects and Methods:** In the online education context, teaching art is no longer limited to the creative use of pedagogical techniques but must evolve to address the psychological needs of both teachers and students. With the shift to remote learning, the absence of physical presence and immediate feedback has led to increased feelings of isolation, disengagement, and stress among students. Teachers, too, face challenges in maintaining their motivation and adapting their teaching approaches to a less interactive environment. Thus, the redefinition of teaching art must not only prioritize fostering meaningful teacher-student interaction but also support psychological well-being by creating an emotionally safe and engaging learning atmosphere.

**Results:** Practically, this requires leveraging information technology to enhance teacher-student communication and address mental health concerns. Tools such as interactive platforms, real-time feedback systems, and personalized learning experiences can help bridge the psychological distance in online education. For instance, creating opportunities for students to share their learning experiences and emotions during class or through anonymous surveys can provide teachers with insights into their students’ mental states. Similarly, integrating mental health support mechanisms, such as access to counselors and stress-relief activities, into the online learning experience ensures that students feel supported and motivated. Teachers, in turn, must develop strategies that foster empathy, adaptability, and responsiveness to students’ psychological needs, allowing them to create a more connected and nurturing virtual environment.

**Conclusions:** Ultimately, the transformation of teaching art in terms of both connotation and application is imperative to meet the evolving needs of online education. By prioritizing the psychological well-being of students and teachers, promoting interactive and empathetic teaching strategies, and leveraging technological advancements, educators can bridge the gaps created by online learning environments. This not only revitalizes the essence of teaching art but also ensures it continues to play a pivotal role in enhancing teacher-student interactions, supporting mental health, and advancing the overall quality of education in an increasingly digital era.

## MHBM24135: THE IMPACT OF WORK-FAMILY FACILITATION ON WORK ENGAGEMENT AMONG PHYSICAL EDUCATION TEACHERS: THE CHAIN MEDIATING ROLE OF PSYCHOLOGICAL RESILIENCE AND JOB SATISFACTION

Ziqi Wang^a^, Wenjun Wang^b^, Siyu Hong^a^, Zixuan Jia^a^, Xiangui Bu^a^

^a^ *Shandong sport university, Jinan 250102, China,* ^b^ *Shandong first medical university, Tai’an 271016, China.*

**Background:** This study aims to explore the impact of work-family facilitation on the work engagement of physical education teachers, with a particular focus on the chain mediation effects of psychological resilience and job satisfaction. While existing research often emphasizes the negative consequences of work-family conflict, there is limited investigation into the positive effects of work-family facilitation, especially within the field of physical education. This study integrates ecological systems theory, spillover theory, social exchange theory, and the two-factor theory to comprehensively understand the relationships between the key variables.

**Subjects and Methods:** A systematic literature review was conducted using authoritative databases such as CNKI and Web of Science. Inclusion criteria comprised peer-reviewed studies related to physical education teachers or educational groups that investigated the relevant variables. The review process followed the PRISMA guidelines, and validated measurement tools were identified, including the Work-Family Facilitation Scale, Resilience Scale, Teacher Job Satisfaction Questionnaire, and Utrecht Work Engagement Scale.

**Results:** 1.Direct Impact of Work-Family Facilitation on Work Engagement: Literature analysis revealed that work-family facilitation significantly enhances the work engagement of physical education teachers. According to spillover theory, positive transmission of emotions and resources between work and family increases teachers’ focus and vitality, validating the multiple role resource enrichment model.2. Mediating Role of Psychological Resilience: Psychological resilience serves as a key mediator in the relationship between work-family facilitation and work engagement. As a positive psychological resource, higher resilience helps teachers maintain psychological stability under pressure, enhancing work engagement and adaptability.3. Mediating Role of Job Satisfaction: Based on social exchange theory, job satisfaction was found to independently mediate the relationship between work-family facilitation and work engagement. When teachers perceive support for their family life from work, their job satisfaction increases, leading to higher work engagement.4. Chain Mediation of Psychological Resilience and Job Satisfaction: Psychological resilience and job satisfaction jointly form a chain mediation effect. High resilience enhances teachers’ sense of recognition and satisfaction, which in turn fosters greater work engagement, supporting the application of the two-factor theory in understanding work motivation and satisfaction.

**Conclusions:** This study, through a systematic review, elucidates how work-family facilitation impacts work engagement among physical education teachers via mediating mechanisms such as psychological resilience and job satisfaction, filling a gap in existing research. It recommends that schools and educational administrators provide psychological support and career development opportunities to help teachers balance work and family, enhancing their job satisfaction and engagement. Future studies should further investigate the moderating effects of individual characteristics, such as gender and teaching experience, and explore the mechanisms of work-family facilitation across different contexts to provide more comprehensive theoretical guidance and strategic recommendations for educational policies and practices.

## MHBM24136: RESEARCH ON THE DEVELOPMENT STRATEGY FOR SHARING SPORTS FACILITIES BETWEEN PUBLIC COMMUNITIES AND SCHOOLS FROM THE PERSPECTIVE OF SOCIAL PSYCHOLOGY

Ke Jiang^a^, Jiaguo Zhu^a^, Fang Jin^a^, Dingcai Liang^a^

^a^ *Yunnan University of Finance and Economics, Kunming 650221, Yunnan, China*

**Background:** Health is the eternal pursuit of human beings, but also the ultimate goal of social development and social governance. At present, with the in-depth implementation of the strategy of “Healthy China”, the sharing of sports facilities between public communities and schools has become a trend, and its reasonable sharing is of great significance for alleviating the shortage of public sports facilities. Social psychology provides a new perspective for studying the psychological mechanism of individuals and groups in the sharing process, and provides theoretical support for the formulation of effective sharing strategies.

**Subjects and Methods:** In this study, questionnaire survey, in-depth interview and case analysis were used to collect feedback from community residents and students on sports facility sharing, analyze the psychological motivation and promoting factors of sharing behavior, and make suggestions for improvement, so as to constantly improve the sharing strategy.

**Results:** According to the feedback information analysis of the survey data on the psychological needs of community residents and students for the sharing of sports facilities, community residents and students generally expect to be able to use these resources more conveniently, and students hope to have more opportunities for sports and exercise outside the campus. In addition, community residents and students’ expectation of shared sports facilities is not only limited to sports activities themselves, but also includes concerns about the environment and safety of sports facilities. They hope that shared sports facilities can provide a safe, clean and well-maintained environment, obtain physical and psychological satisfaction during sports, and enhance mutual communication and interaction. To form a closer and more harmonious community relationship, and provide a feasible reference for the sharing mode of sports facilities between public communities and schools. In addition, the research also found that the psychological motivations of community residents and students’ sharing behavior are complex and diverse, including individual needs, social identity, group pressure and other factors.

**Conclusions:** The research shows that the sharing mode of public community and school sports venues facilities can realize efficient utilization and optimal allocation of resources through reasonable planning and management, which can not only alleviate the problem of insufficient public sports facilities, but also promote the physical and mental health of community residents and students. In addition, community residents’ acceptance of shared facilities is closely related to their perception of community belonging. According to Maslow’s hierarchy of needs theory, when community residents feel that their basic social needs are met, they are more willing to participate in and support the use of shared facilities.

**Acknowledgments**

This work was supported by General Project of Pedagogy of National Social Science Foundation of China, titled “Research on Paid and Unpaid Development of Sports Facilities in Colleges and Universities under the Background of Big Health”, Project Approval No. BLA200215.

## MHBM24137: STUDY ON THE EFFECTS OF DIFFERENT EXPRESSION PROCESSING ON THE MEMORY OF MEANINGLESS WORDS BASED ON MENTAL HEALTH

Yanting Pan^a^, Zaifeng Yang^a^

^a^ *Institute of Teacher Education, Wuzhou University, Wuzhou, 543002, Guangxi, China.*

**Background:** Facial expression plays an indispensable role in interpersonal communication. In recent years, more and more researchers have begun to pay attention to the expression of emotions (such as joy, anger, sadness) and their effects on interpersonal relationship and mental health. So, how does it affect the effect of memory when the expressions of joy, anger and joy are presented at the same time as the memory task? Which emotional expressions enhance memory more effectively? These problems need to be explored in depth.

**Object and Methods:** This study adopted the learning-recognition paradigm to explore the effects of emotional expression processing on memory and the characteristics of neural activities behind it. The experiment mainly studied the influence of emotional expression on semantic processing, and analyzed the potential influence of emotional expression on individual mental state (such as anxiety, inferiority, sensitivity, etc.).

**Results:** The results showed that there was no significant difference in the effect of word memory under positive, neutral and negative facial expressions. In the EEG results, although there was no significant difference in the memory effect of the three emotional conditions, the N400 waveform appeared obviously in the time window of 350-450 ms, indicating that the subjects could recall the learned content while performing semantic processing. In the experiment, the effect of new and old words is significant, which shows that the correct recognition rate of new words is higher than that of old words. In the time window of 450-650 ms, the average amplitude of the new words is significantly higher than that of the old words, which indicates that the participants have performed deeper semantic processing than the old words when processing the new words. This finding also suggests that a good mental health state may contribute to memory and help individuals to process new information more effectively.

**Conclusion:** First, when expression is not related to semantic information, it has no significant effect on memory effect, which indicates that the connection between information nodes is loose, which makes it difficult for participants to integrate information. Second, when expression is related to semantic information, its consistency significantly promotes memory effect. Under the condition of negative expression, the consistency of expression and semantics can improve the accuracy of memory. Under the condition of positive expression, when the expression is consistent with the semantic meaning, the reaction time of the subjects is significantly faster. This study is not only significant for understanding memory mechanism, but also provides a new perspective for individual mental health.

**Acknowledgements**

This paper is one of the stage achievements of the teaching content construction and practice (2408030616) of the course “Education Research Methods” under the comprehensive literacy orientation National Social Science Fund Planning Project: Research on Practical Model of Health and Nursing Integration Services Based on Data Empowerment in Western Regions (24XGL046); 2024 Annual Guangxi Philosophy and Social Science Research Project: Study on the Ethical Risks and Prevention of Artificial Intelligence in Public Health.

## MHBM24138: ENHANCING MOTIVATION OF BUSINESS ENGLISH MAJORS WITH ECOLOGICAL PEDAGOGY: EFFECTS OF PSYCHOLOGICAL NEEDS SATISFACTION ON STUDENTS’ ENGAGEMENT IN ECOLOGICAL EDUCATION

Xiuqin Li ^a^

^a^ *Department of Foreign Language, Quzhou University, Quzhou, 324000, China.*

**Background:** This research investigates the potential of ecological pedagogy to enhance motivation among Business English majors while pursuing the satisfaction of psychological needs which exerts great impact on students academic engagement. Traditional teaching methods often fall short in fostering intrinsic motivation and deep learning, leading to disengagement and a lack of connection to real-world applications. Ecological Pedagogy (EP), rooted in principles of interconnectedness, sustainability, and experiential learning, offers a promising alternative. This study examines the theoretical underpinnings of motivation in light of cognitive psychology in Business English learning, drawing on Self-Determination Theory, Achievement Goal Theory, and Expectancy-Value Theory. It then explores the core tenets of ecological pedagogy, emphasizing its compatibility with Business English education.

**Subjects and Methods:** The research mainly employed a qualitative approach, integrating ecological principles into the instruction of a Business English program. Data collection involved surveys and student interviews to assess the impact of EP on student motivation, engagement, and learning outcomes in BE learning.

**Results:** Based on the analysis of research data, we can see the EP group who undertook disciplinary instruction with EP demonstrated significantly higher motivation across all statements compared to the control group who undertook traditional instruction no matter in terms of intrinsic motivation or extrinsic motivation. The students of EP group showed higher enjoyment of learning Business English, a stronger willingness to engage in autonomous study after class, and a greater belief in their ability to make significant progress. While confidence levels in business communication were moderate for both groups, the EP group still had a higher mean score. As for extrinsic motivation, both groups showed high motivation for exam-oriented learning and job market prospects, but the EP group outperformed the control group in terms of motivation for successful international business communication. These findings suggest that Ecological Pedagogy may be an effective approach to enhance motivation and self-directed learning in Business English education.

**Conclusions:** Findings indicate that ecological pedagogy can foster a more stimulating and meaningful learning experience for Business English majors, promoting intrinsic motivation, deeper understanding of subject matter, and a heightened sense of social responsibility. Challenges associated with implementation, such as the demand for faculty professional development and increased reliance on technology-aided teaching, are also discussed. The study concludes by highlighting the efficacy of ecological pedagogy in Business English education and suggesting directions for future research and practice.

**Acknowledgments**

This work was supported by the 2023 Ministry of Education Industry College Cooperation Collaborative Education Project of A Study on the Innovative Path of Business English Teaching Based on College Enterprise Cooperation in the Context of New Liberal Art (Grant No.230829564107193).

## MHBM24139: RESEARCH ON THE INFLUENCE MECHANISM OF JOB-REPLACEMENT ANXIETY IN HUMAN-INTELLIGENT MACHINE COLLABORATION

Guangling Zhong ^a^

^a^ *GUANGZHOU HUASHANG COLLEGE, Guangzhou, Guangdong 511300, China*

**Background:** With the popularization and application of artificial intelligence (AI) technology, the scenario of human-intelligent machine collaboration to complete work tasks within organizations is becoming an increasingly common phenomenon. With the normalization of human-intelligent machine collaboration, employees are more frequently encountering the risk of job replacement. This perceived risk in the context of collaborative work with intelligent machines intensifies employees’ anxiety and unease.

**Subjects and Methods:** This study took 636 enterprise employees who use intelligent machine or intelligent technology products to assist in their work as the research objects, with self-efficacy as the mediating variable and AI technology training as the moderating variable, to analyze the impact mechanism of employees’ job replacement anxiety in the context of human-intelligent machine collaboration.

**Results:** The research results are as follows: Firstly, there is a positive correlation between human-intelligent machine collaboration and employees’ job replacement anxiety. That is, the higher the degree of human-intelligent machine collaboration, the more likely employees are to have job replacement anxiety or the stronger the job replacement anxiety. Secondly, AI technology training does not moderate the negative relationship between human-intelligent machine collaboration and job replacement anxiety. Thirdly, self-efficacy plays a mediating role in the process of human-intelligent machine collaboration influencing job replacement anxiety. Under the situation of human-intelligent machine collaboration, employees’ sense of job replacement anxiety is getting stronger and stronger. Organizations should attach great importance to this and do their best to help employees reduce such anxiety, enabling them to focus more on improving work efficiency and work quality.

**Conclusions:** When enterprises aim to help employees reduce their sense of job replacement anxiety, they cannot simply consider the impact of implementing AI technology training on employees. Moreover, self-efficacy will affect employees’ perception of job replacement anxiety. This study provides valuable insights for enterprises to better understand and address the issue of employees’ job replacement anxiety in the era of human-intelligent machine collaboration, highlighting the importance of considering multiple factors rather than relying solely on AI technology training. It also contributes to the further exploration of the complex relationship between human-intelligent machine interaction and employees’ psychological states in the workplace.

## MHBM24140: AN ANALYSIS OF THE MALE AND FEMALE STEREOTYPES AND THE IMPACTS ON FEMALE’S PSYCHOLOGICAL NEEDS: THE CASE OF A DATING PROGRAM “YOU ARE THE ONE”

Yating Zhang ^a^

^a^ *School of Foreign Languages, Anhui Vocational and Technical College, Hefei, 230011, China.*

**Background:** Mass media has created a large number of gender stereotypes, which is an important way for people to form gender stereotypes indirectly. With the increasing popularity of “You Are the One” launched by Jiangsu Satellite TV channel, it quickly became the most popular TV dating program in China. The relationship between stereotypes caused by this program and female’s psychological needs is attracting public attention.

**Subjects and Methods:** The dialogues happened among the host and all male and female paticipants in program “You Are the One” on November 17, 2018 are typed into a text, then a group of three doctors who major in English translated the dialogues into English. In this way, a data with 11222 English words and 19506 Chinese characters were created. With a case study of “You Are the One”, this paper analyzed the statements expressed by all male and female participants so as to explored the male and female stereotypes and the impacts this program cast on female’s psychological needs with data analysis and discourse analysis.

**Results:** The program’s stereotyped expression of gender constructs and strengthens the gender stereotype of the audience, depriving social members of their diversity and uniqueness as “people” to a great extent, the program can easily form and strengthen the unfairness of gender distribution of social resources, which will restrict and hinder the satisfaction of female’s psychological needs for self-esteem and self-actualization. The traditional characteristics of women are still men’s tendency to choose female guests, and the image of Chinese women still caters to the constraints of Chinese traditional ideas on women, which caused frustration and depression to women.

**Conclusions:** Mainstream values with fairness and respect and self-actualization should be established. Changing the stereotyped gender expression of the media and create a fair and harmonious gender environment is of importance. We should reflect, eliminate and criticize the existing tradition centered on male hegemony so as to meet the female’s psychological needs of self-esteem and self-actualization. We should also try to avoid simply dividing people’s roles, values, personality, abilities and grades according to their physical characteristics. TV programs should shoulder the responsibility of eliminating discrimination, prejudice, oppression and objectification against women. We can build advanced gender culture which is “people-oriented”, respecting different levels of needs individual has and giving each individual equal development opportunities, rights, responsibilities and rational allocation of social resources, so that females can fully develop their potential and satisfy their psychological needs of respect, be loved, self-esteem and self-actualization according to their personal characteristics rather than gender framework in a fair and harmonious gender environment.

**Acknowledgments**

This work was supported by a project grant from Anhui Province Higher Education Science Research Project (Humanities and Social Sciences) “Research on Pragmatic Presupposition Discourse Analysis from the Perspective of High Context Culture and Low Context Culture Theory” (Grant No.: 2024AH052689).

## MHBM24141: A MULTIDIMENSIONAL AND IN-DEPTH ANALYSIS OF NETIZEN PSYCHOLOGY FROM THE PERSPECTIVE OF INTERNET LANGUAGE

Ou Peng^a^, Lizhu Wang^a^, Kaijuan Zhou^a^, Wenwen Cheng^b^

^a^ *Chengdu University, Chengdu, Sichuan, 610106, China,* ^b^ *Chongqing Normal University, Chongqing, 401331, China.*

**Background:** As of June 2024, the number of Chinese netizens has reached 1099.67 million, and online language is experiencing explosive growth. The academic community lacks in-depth analysis of psychological differences in different cultural contexts. This article deeply analyzes the psychology of netizens from multiple dimensions of internet language, aiming to accurately reveal the psychological mechanisms behind internet language and provide theoretical and practical support for the healthy development of internet culture to improve netizens’ mental health.

**Subjects and Methods:** This study focuses on the close relationship between internet slang and netizen psychology. Analyzed the characteristics of 987 internet slang, surveyed 540 netizens, summarized the psychological motivations for netizens to use internet slang, and revealed the complex relationship between internet slang and netizens’ psychology. This article uses literature research method to sort out relevant theoretical achievements; Using a questionnaire survey method, 540 netizens were surveyed to understand their habits and psychological needs in using internet language; Exploring the psychological stories behind typical internet slang through in-depth interviews; Using content analysis to analyze the psychological information contained in online language texts and find the methods to promote mental health of netizens.

**Results:** Through analyzing 987 internet slang, it was found that internet slang mainly includes film, television, animation, games, dialects, homophones, abbreviations, and symbols. It has characteristics such as innovation and uniqueness, simplicity and efficiency, popularity and timeliness, imagery and interest, diversity and inclusiveness. Through a survey of 540 netizens, it was found that their psychological motivations for using online language are mainly to stand out, crave group identity, and release social pressure. Data shows that during the outbreak of popular online events, the dissemination speed was very fast, reflecting netizens’ attention to hot topics and their strong desire to participate in discussions and share opinions. They quickly integrate into the trend of topic discussions through online language, becoming an important sdriving force for the dissemination of online culture, and deeply reflecting the complex relationship between online language and netizens’ psychology.

**Conclusions:** Film, television, animation, and games are the main sources of online language, and the generation of online language is closely related to the psychological needs of netizens. The theoretical basis mainly comes from four aspects: social psychology, cognitive psychology, personality psychology, and behaviorism psychology. The dissemination of online language is driven by various psychological mechanisms and has a negative impact on the psychology of netizens.

**Acknowledgements**

This work was supported by a project grant from Ministry of Education Humanities and Social Science Fund project “Collation and Research of Characters in Vietnamese Han Nan Chinese Ancient Books” (Grant No.23XJC40001) and Key project of Humanities and Social Science Planning of Chongqing Education Commission “Research on Chinese Characters from the Perspective of Foreign and Local Chinese Ancient Books” (Grant No. 22KGH088).

## MHBM24142: RESEARCH ON THE DRIVING FACTORS OF THE INTEGRATED DEVELOPMENT OF DIGITAL ECONOMY AND REAL ECONOMY AND THE INFLUENCE OF MANAGERS’ PSYCHOLOGICAL STRESS AND DECISION-MAKING

Fan Yang ^a^

^a^ *Zhengzhou Normal University, Zhengzhou 450044, China.*

**Background:** This paper mainly discusses the driving factors of the integration and development of digital economy and real economy. Based on the systematic review of relevant literature, this paper analyzes the internal mechanism of the integration of digital technology and the real economy and the driving factors of the integration and development of the real economy, discusses the driving role of managers’ behaviors on the integration and development, and verifies the effectiveness of the theoretical model. It provides theoretical basis and practical guidance to promote the deep integration of digital economy and real economy. The driving force of the integration of the real economy has an all-round impact on the psychological decision-making of managers. Managers’ emotional stability, depression and anxiety, mental health and emotional stability affect the quality of their decisions to some extent. They must constantly learn and adapt to make correct management decisions in the new economic environment and lead the enterprise to success.

**Subjects and Methods:** This paper uses Matlab software, on the basis of the selected analysis index dimensions, to clarify the statistical boundary of digital economy and real economy for statistics, and uses R&D model for simulation. Through the selection of digital economy related enterprises to visit and investigate, to understand the degree of penetration and integration of digital technology in enterprises. Analyze the common characteristics of digital economy enterprises and the differences between different enterprises and different industries, and visit authoritative statistical institutions such as the Bureau of Statistics to understand the difficulties and challenges faced by China in classifying digital economy industries. In addition, the managers’ mental health, emotional stability, anxiety and depression caused by work pressure are studied. While studying the impact mechanism of digital economy on economic growth, it also pays attention to the impact of managers’ physical and mental health.

**Results:** First of all, the research on the impact of managers’ mental decision-making shows that managers’ emotional stability, mental health, anxiety and mental attitude are affected by cognitive bias, risk preference, information asymmetry and decision-making situation. Secondly, the driving force of the integrated development of digital economy and real economy is biased in different industries. Managers may be resistant, because change is often accompanied by a sense of threat to the status quo, and managers may be resistant to new tools or processes.

**Conclusions:** This paper analyzes the industrial correlation heterogeneity of digital economy, because according to the theory of regional economics, anxiety and depression caused by resources, traffic, regional location, managers’ mental health, and work pressure in different regions. In addition, there are differences in population and other factors, which lead to unbalanced economic development, so it will also have a certain impact on the direction and degree of industrial integration.

**Acknowledgements**

1. This paper is a general research project of humanities and social sciences in universities of Henan Province. Project name: Accelerating the construction of Zhengzhou National Central City, project leader: Yang Fan, project approval number: 2024-ZDJH-315, periodic research results. 2. This paper is a Soft Science Research project of Henan Province in 2025, Department of Science and Technology of Henan Province. Project name: “Study on the impact of digital intelligent Drive on the Green Development Efficiency of Henan Manufacturing Industry” phased research results project.

## MHBM24143: PHYSICAL ATTRIBUTES AND PSYCHOLOGICAL CHARACTERISTICS OF BASKETBALL PLAYERS TOWARDS CREATION OF A HEALTH MANAGEMENT

Shuai Zhu ^a^

^a^ *Sport institute, Yulin University, Yulin, Shaanxi 719000, China.*

**Background:** The objective of this research is to examine the evaluations provided by 44 basketball coaches according to the physical features and psychological traits and talents of their players. Relying on these evaluations for health management and serving as the basis for health management.The primary aim is to enhance coaching effectiveness and promote overall well-being. The profiles of coaches exhibited a broad range of coaching backgrounds, including various age groups, genders, and years of experience.

**Subjects and Methods:** The evaluation of athletes’ physical characteristics, including speed, agility, core strength, and leaping ability, was carried out, uncovering notable variations in coaches’ evaluations of athletes’ speed across different age categories. Nevertheless, there were no notable disparities observed with regards to any physical characteristics. Furthermore, an evaluation was conducted to assess the psychological attributes and abilities of players, such as visualization, relaxation, attentional focus, anxiety management, self-confidence, and motivation towards athletic success.

**Results:** The findings revealed that there were no notable variations based on the age, gender, or years of experience of the coaches. Furthermore, the research has successfully proven a noteworthy correlation between the physical features and psychological characteristics and talents of players, thereby emphasizing the interdependence of these dimensions within the realm of coaching.Coaches have poor health management abilities for team members, and emphasis should be placed on cultivating this ability. Therefore, it is suggested that a comprehensive wellness program be implemented for basketball coaches, with a primary focus on strengthening coaching efficacy.

**Conclusions:** This can be achieved by developing coaches’ understanding and proficiency in analyzing and enhancing players’ physical features, as well as their psychological traits and Self-health management methods. The program integrates a range of tactics, including scheduled training sessions, workshops, and mentorship opportunities, with the aim of addressing multiple dimensions of coaching well-being. Through the implementation of this program, basketball coaches have the opportunity to enhance their coaching capabilities and make a positive impact on player development and team success. This program underscores the significance of continuous support and development for coaches in fostering the physical and psychological growth of athletes in the realm of basketball.

## MHBM24144: DATA ASSET VALUE ASSESSMENT OF PHARMACEUTICAL ENTERPRISES BASED ON MULTI-PERIOD EXCESS RETURN MODEL: A CASE STUDY OF WUXI APPTEC

Liu Yang^a^, Xiaolin Wang^b,c^

^a^ *Guangzhou Panyu Polytechnic, Guangzhou 510300, China,* ^b^ *Guangdong Polytechnic Normal University, Guangzhou 510300, China,* ^c^ *Guangzhou Hongxing Decoration Engineering Co., Guangzhou 510300, China*

**Background:** With the rapid development of Internet technology, data assets have become the core driving force for enterprise transformation and upgrading. However, the field of data asset value assessment still faces many challenges and lacks a unified and effective valuation system. This study aims to solve the challenges of data asset value assessment by constructing a value assessment model applicable to the characteristics of data assets, and provide scientific guidance for enterprises in the management and mining of data assets.

**Subjects and Methods:** This study firstly reviews the relevant theories and existing methods of data asset value assessment, and deeply analyzes the key factors affecting the value of data assets. Building on this foundation, we optimize the multi-period excess return method to construct a value assessment model for data assets. This model measures the overall enterprise earnings, deducts the contribution value of other assets, and attributes the residual earnings to data assets. These earnings are then discounted to their present value using an appropriate discount rate to assess the value of the data assets. To validate our model, we select WuXi AppTec, a leading global pharmaceutical R&D enabling platform with significant contributions to psychological disease drug R&D, as a case study. We apply the constructed valuation model to WuXi AppTec’s financial data and market performance, taking into account its unique data-driven approach to drug discovery.

**Results:** Through the case study, we find that WuXi AppTec’s data assets, which have significantly contributed to its psychological disease drug R&D efforts, are valued at $10.361 billion on the valuation base date (December 31, 2023). Furthermore, our analysis indicates that these data assets are expected to generate substantial year-on-year incremental excess returns in the coming years, highlighting their strong economic benefits and growth potential in the field of psychological disease drug R&D.

**Conclusions:** This study successfully constructs a data asset value assessment model based on the multi-period excess earnings method. The case study of WuXi AppTec demonstrates the model’s effectiveness and feasibility, underscoring the important role of data assets in enterprise value creation, particularly in the pharmaceutical industry. Our findings suggest that digital transformation, driven by the effective utilization of data assets, is crucial for enhancing enterprise competitiveness in the field of psychological disease drug R&D. In the future, this model is expected to provide a scientific basis for data asset value assessment across the pharmaceutical industry and potentially other sectors, facilitating the digital high-quality transformation of enterprises.

**Acknowledgments**

This work was supported by a project grant from the 2021 Key Projects for General Universities of Guangdong Province (Grant No. 2021ZDZX3028).

## MHBM24145: RESEARCH ON THE PATH OF INTEGRATING MENTAL HEALTH EDUCATION INTO BLENDED TEACHING OF IDEOLOGICAL AND POLITICAL COURSES IN COLLEGES AND UNIVERSITIES

Saiyu Zhang ^a^

^a^ *Minjiang University, Fuzhou 350108, China.*

**Background:** The traditional teaching mode of ideological and political courses in colleges and universities is gradually unable to meet the diversified needs of college students in the new era, especially in the aspects of value shaping and ideological guidance, it is urgent to carry out teaching innovation. Ideological and political courses in colleges and universities should not only impart knowledge, but also pay attention to the psychological growth and emotional needs of college students. With the fierce competition in modern society, college students are faced with multiple troubles such as academic pressure and employment pressure, and mental health problems have gradually become an important factor affecting students’ physical and mental development. How to integrate mental health education in ideological and political lessons to help students improve their psychological quality has become an important topic in the field of education. As a new model combining traditional classroom and online education, blended teaching mode provides an opportunity for the deep integration of ideological and political lessons and mental health education. This mode breaks the limitations of traditional classroom, can more flexibly meet students’ personalized learning needs, and promotes the organic combination of mental health education and ideological and political lessons.

**Subjects and Methods:** The research object of this paper is college students, focusing on the implementation of the integration of ideological and political courses and mental health education in colleges and universities, aiming to explore how to effectively promote the two-way improvement of students’ ideological and political quality and mental health through the mixed teaching mode. The research methods include literature analysis, questionnaire survey, case analysis and interview.

**Results:** The results show that the mixed teaching model can effectively promote the deep integration of mental health education and ideological and political lessons. Through online and offline interaction, students’ sense of participation and emotional identity are enhanced, and students’ psychological quality and ideological and political level are improved. Students have a high acceptance of this fusion mode, believing that it is helpful to relieve psychological pressure and enhance psychological adjustment ability. Teachers’ psychological literacy and interdisciplinary collaboration ability are crucial to the integration effect. Finally, the study suggests that universities continue to optimize curriculum design and teacher training.

**Conclusions:** The integration of mental health education and ideological and political courses in colleges and universities can effectively improve students’ comprehensive quality. Through the organic coordination between online and offline, the blended teaching mode promotes the development of students’ mental health and the simultaneous improvement of ideological and political literacy. Research shows that teachers’ psychological knowledge and interdisciplinary collaboration ability are crucial to the integration effect. The article suggests that universities should strengthen the integration of curriculum content, optimize teaching methods, and enhance the professional level of teachers, so as to promote the deep integration of ideological and political courses and mental health education.

**Acknowledgments**

This work was supported by Education and Teaching Research Project of Fujian Province: “Comprehensive Reform of ‘Four-in-one’ Practical Teaching Mode of Ideological and Political Courses in Colleges and Universities Based on Resource Optimization” (FBJY20230094); Minjiang University first-class undergraduate course construction project: “The Basic Principles of Marxism Online and Offline mixed course” (MJU2021KC302); Minjiang University Public basic Course construction project “Basic Principles of Marxism” (MJU2022KC108); Minjiang University Fashu Charity Foundation donated funds research special Humanities and Social science key project: “Yan Fu Cultural Subjectivity Thought historical investigation and practical significance research” (MFS24001).

## MHBM24146: INTEGRATING MENTAL HEALTH IN PRACTICE TEACHING MODELS OF ENGINEERING EDUCATION AT PRIVATE UNIVERSITIES: A CASE STUDY OF HANKOU UNIVERSITY’S SCHOOL OF AERONAUTICS AND INTELLIGENT MANUFACTURING

Yan Deng^a^, Xiaowei Guo^a^, Xianyong Xu^a^, Can Yuan^a^, Xia Zhao^a^, Yiwen Hu^a^

^a^ *Hankou University, Hubei 430212, China*

**Background:** The primary objective of this study is to delve into the incorporation of mental health considerations within the pedagogical frameworks of engineering education at private universities. This research endeavors to scrutinize the case of Hankou University’s School of Aeronautics and Intelligent Manufacturing, with the aim of elucidating how the integration of mental health support and awareness can potentially amplify student success in technical fields. By examining the interplay between mental health initiatives and academic performance, this study seeks to provide a comprehensive understanding of the benefits that mental health integration can bring to the engineering discipline. The importance of this research is underscored by the growing recognition of mental health’s role in academic success and the subsequent need for educational institutions to adapt their strategies accordingly.

**Subjects and Methods:** Adopting a mixed-methods approach, this study amalgamates quantitative analysis of student performance metrics and employment statistics with in-depth qualitative interviews conducted with both faculty and students. The quantitative data provides a macro perspective of the overall trends and patterns, while the qualitative insights offer a nuanced understanding of individual experiences and perceptions. The analysis zeroes in on the nexus between mental health programs and scholastic achievements, scrutinizing key initiatives such as the incorporation of cognitive internship practices and avant-garde teaching methodologies, all of which are fortified by a suite of mental health resources. These resources include, but are not limited to, interactive workshops and personalized counseling sessions. Furthermore, the study delves into the institutional commitment to fostering an environment conducive to psychological well-being, assessing the efficacy of such investments in bolstering academic outcomes. The mixed-methods approach allows for a more holistic understanding of the impact of mental health integration, capturing both the broad trends and the individual student experiences.

**Results:** The study’s findings underscore a robust positive correlation between mental health support and enhanced student performance. A case in point is the School of Aeronautics and Intelligent Manufacturing, which has witnessed a marked escalation in employment rates, hitting a pinnacle of 97.06% in 2024. The data suggests that the enhancement of practical teaching facilities, coupled with the infusion of mental health resources, has been instrumental in achieving these results. These interventions have been shown to cultivate resilience and mitigate anxiety among students, thereby optimizing their academic trajectories. Qualitative interviews reveal a pervasive sentiment among students that they feel more prepared and emboldened due to the holistic approach that intertwines mental health considerations within the academic curriculum. The synergistic effect of a nurturing campus milieu, complemented by regular mental health workshops and tailored counseling, has been pivotal in cultivating a more salubrious academic climate. The results indicate that mental health support not only improves academic performance but also enhances students’ overall well-being and preparedness for future challenges.

**Conclusions:** Integrating mental health into practice teaching models is imperative for private universities that aspire to enhance the efficacy of their engineering programs. The case study of Hankou University serves as a compelling exemplar of the profound impact that can be achieved by synergizing educational strategies with mental health support. In addressing industry demand while simultaneously ensuring the comprehensive development of students, it is imperative for engineering programs to enshrine psychological well-being as a core component of their strategic planning. This paradigm not only bolsters academic achievements but also equips students with the emotional fortitude necessary to navigate the challenges of future professional endeavors. By prioritizing mental health, institutions can foster a new generation of engineers who are not only technically proficient but also emotionally resilient, ready to meet the demands of the industry with both intellectual and emotional readiness.

**Acknowledgments**

This study was supported by the Ministry of Education of the People’s Republic of China through the School-Enterprise Cooperation Collaborative Education Project (No. 221006517131032), the Supply-Demand Matching Employment Education Project (No. 2023122984622), and the Major Project of Teaching Reform Research at Hankou University in 2023 (No. 2023JY03). Special thanks to these initiatives for their support.

## MHBM24147: THE PERCEPTION AND DESIGN EVALUATION OF COMMUNITY MEDICAL SYSTEMS ON MEDICAL HEALTH DIAGNOSIS FROM THE PERSPECTIVE OF COGNITIVE LOAD

Peng Han^a^, Zehua Pan^b^

^a^ *Xiamen Huaxia College, Xiamen, 361024, China,* ^b^ *Jiangxi Industry Polytechnic College, Nanchang 330095, China.*

**Background:** As internet technology advances, community clinics have begun to implement Medical Information Systems (MIS) to assist clinicians in the diagnosis and treatment of diseases, aiming to enhance the efficiency of healthcare professionals in the fields of disease diagnosis, treatment, and public health education. However, community physicians often face an excessive cognitive load due to the unreasonable layout of the system interfaces. This not only affects the sensory experience of doctors during their work but also reduces the accuracy and timeliness of disease diagnosis, potentially yielding negative impacts on patient treatment outcomes.

**Methods:** To address this challenge, the current investigation, rooted in the tenets of cognitive load theory and integrating the sensory perceptions of healthcare practitioners with the Analytic Hierarchy Process (AHP), seeks to investigate the correlative mapping between the processing of medical information and the perceptual experience within community-based healthcare information systems. The goal is elevate the standard of diagnostic care delivered by community medical facilities. Initially, the research meticulously delineates the discomfort points in the sensory engagement with the medical information system during the diagnostic procedure by synthesizing user experience mapping with the Subjective Workload Assessment Technique (SWAT). Drawing from these identified discomfort points, the study examines 15 cognitive load parameters that influence the clinical user experience and converts them into actionable user requirement metrics. Thereafter, the AHP is utilized to construct a hierarchical framework of diagnostic evaluation criteria for community healthcare information systems, with the definitive weight and prioritization of each criterion ascertained through the analysis of a comparative judgment matrix. The research elucidates the sensory perceptions of physicians during the utilization of the diagnostic system.

**Results:** Community physicians often encounter difficulties such as the identification of interface elements and slow comprehension of information during the processes of acquiring, processing, and receiving feedback from medical information systems when disease diagnosis. To alleviate the issues caused by excessive cognitive load, researchers have integrated the identified pain points of the perceptual experience with cognitive load theory.Reveals the correlations between user perceptual needs and experience evaluations, and distills strategies for the interface design and optimization of community diagnostic and treatment systems.

**Conclusions:** Optimizing the text within medical information system interfaces can effectively mitigate the psychological load imposed on physicians during disease diagnosis due to insufficient information perception. Prolonging the interaction wait time can assist physicians in achieving a better understanding during the diagnostic process. Enhancing the interface’s guidance, adaptability, and comfort can help address the experiential challenges faced by doctors in terms of patient information feedback and system operation during disease diagnosis.

**Acknowledgments**

This work was supported by a project grant from education and research of young and middle-aged teachers in Fujian Province in 2022, grant number(JAS22213).

## MHBM24148: RESEARCH ON PERFORMANCE EVALUATION BASED ON PSYCHOLOGICAL NEEDS

Jianjian Zhao ^a^

^a^ *Department of History, Beijing Institute of Education, Beijing, China*

**Background:** Performance evaluation is an assessment method that gauges students’ abilities by requiring them to complete tasks or activities in specific contexts. Evaluators determine if students have achieved the expected learning goals by observing, recording, and analyzing their performances, including behaviors, creations, and outcomes. Unlike traditional pen-and-paper tests, which focus on the retention and reproduction of knowledge, performance evaluations aim to provide a more comprehensive assessment of students’ abilities and psychological states.

**Methods:** This study investigates the role of performance evaluation in education from the perspective of psychological needs. It specifically examines how performance evaluation satisfies students’ needs for autonomy, competence, and belonging, and its positive effects on learning motivation, psychological well-being, and social interactions. Performance evaluation is an evaluation method that focuses on students’ performance in specific tasks, requiring them to complete tasks in real or simulated contexts. Unlike traditional paper-and-pencil tests, performance evaluation emphasizes both the process and outcome of tasks, providing a more comprehensive evaluation of students’ abilities, especially in areas such as knowledge application, problem-solving, and creative thinking. This approach also promotes psychological development.

**Results:** The study reveals that performance evaluation effectively fulfills students’ primary psychological needs: autonomy, competence, and belonging. Firstly, providing students with choices and autonomy in task completion and problem-solving stimulates intrinsic motivation and enhances their self-efficacy. Secondly, engaging in challenging tasks such as mathematical modeling or scientific experiments allows students to achieve a sense of accomplishment, improving their self-perception and competence. Finally, group tasks within performance evaluation meet students’ need for belonging, fostering teamwork, social connection, and positively impacting their social skills and psychological health.

**Conclusions:** By addressing students’ psychological needs, performance evaluation fosters both academic achievement and psychological well-being. Educators should design assessment tasks that cater to these psychological needs, thereby improving teaching effectiveness and supporting the holistic development of students.

## MHBM24149: EXPLORING THE MEDIATING ROLE OF CULTURAL IDENTITY IN THE RELATIONSHIP AMONG UNDERGRADUATES’ GLOBAL COMPETENCE, MOTIVATION, AND ANXIETY IN LEARNING ENGLISH

Yan Zhao^a^, Chetanath Gautam^b^

^a^ *Sanming University, Sanming 365004, China,* ^b^ *Delaware State University, 19901, USA.*

**Background:** Global competence and English language skills are increasingly essential for undergraduates in this globalized society. This study examines the mediating role of cultural identity in the relationship between undergraduates’ global competence, motivation, and anxiety to learn English in Southern China, focusing on alleviating language learning anxiety from a psychological perspective.

**Subjects and Methods:** This study employed an explanatory sequential mixed-method design, which combined quantitative and qualitative approaches to analyze the mediation of cultural identity between global competence and English learning motivation and explore ways to reduce English learning anxiety. Quantitative data were collected from 1,215 undergraduate respondents via online surveys, followed by statistical analyses such as regression and mediation tests using SPSS. For the qualitative phase, 12 in-depth interviews were conducted with a subset of participants based on survey responses. NVivo software was used to code and organize the data.

**Results:** Regression analyses confirmed the mediating role of cultural identity in the relationship between global competence, English learning motivation, and anxiety. The global competence regression analysis indicated that learning motivation impacted global competence (β = 0.468, P < 0.001). Cultural identity positively affected global competence (β = 0.399, P < 0.001), confirming its role as a partial mediator. In the domain of knowledge, skills, attitudes, and values regression analysis, qualitative data findings suggest that cultural identity enhances global competence and facilitates a reinforcing cycle where engagement with global cultures through English deepens students’ appreciation for their culture. Positive cultural identity can improve college students’ global competence and regulate English learning anxiety. The impact of different language systems on cultural identity and the expectations of parents and teachers may cause students to have emotional states such as anxiety, and English learning anxiety will also have a counter-effect on cultural identity.

**Conclusions:** This study explores the mediating role of cultural identity between global competence, motivation, and anxiety about learning English, which shows that a strong sense of cultural identity may alleviate English learning anxiety and enhance motivation among undergraduates. Culture identity plays an importance role on improving undergraduates’ language learning and global competence. Strategies based on cultural identity and creating a good learning atmosphere can be adopted to reduce anxiety. Practice recommendations are provided for institutions, teachers, policymakers, students/parents/community leaders, and educational technology developers, along with suggestions for further research.

**Acknowledgments**

This work was supported by a project grant from *horizontal project in collaboration between Sanming university and Foreign Language Teaching and Research Press Co., Ltd* (Grant No.KH190104).

## MHBM24150: PSYCHOLOGICAL PERSPECTIVES ON TAX PLANNING STRATEGIES: ENHANCING FINANCIAL DECISION-MAKING AND CORPORATE MANAGEMENT IN SEED INDUSTRY ENTERPRISES

Chunwei Shen ^a^

^a^ *Anhui Institute of International Business, Hefei, Anhui 231131, China*

**Objective:** This study examines the psychological dimensions of tax planning strategies in seed industry enterprises, focusing on how psychological factors influence financial decision-making and corporate management practices. The research aims to integrate behavioral finance concepts to improve the effectiveness of tax planning and corporate financial health while addressing cognitive biases and risk perceptions inherent in financial decision-making.

**Methods:** Using a case study approach, this research analyzes M Seed Industry Company’s corporate income tax planning strategies. Financial data from 2022 and 2023, alongside qualitative assessments of managerial decision-making processes, were used to explore the relationship between psychological factors and tax planning efficiency. Key variables include risk perception, decision confidence, and cognitive biases. The study incorporates structured interviews with financial managers to evaluate the psychological impact of tax planning strategies on corporate decision-making.

**Results:** The findings reveal that effective tax planning reduces financial stress among managers and enhances decision-making confidence. Strategic adjustments in revenue recognition timing, cost accounting, and tax incentive utilization significantly improve financial outcomes and cash flow. However, cognitive biases, such as overconfidence and loss aversion, were found to hinder optimal tax strategy implementation. Addressing these biases through targeted training and behavioral interventions enhances the psychological readiness of managers for complex tax planning tasks.

**Conclusion:** Incorporating psychological insights into tax planning fosters more effective financial decision-making and corporate management. By addressing cognitive biases and enhancing decision-making confidence, seed industry enterprises can optimize tax strategies, improve cash flow, and strengthen financial stability. Future research should explore the long-term psychological impacts of tax strategies across diverse industries to refine behavioral finance applications in corporate management.

**Acknowledgments**

This work was supported by Humanities and social science research projects of colleges and universities in Anhui Province: Research on the Innovation and Upgrading of Anhui High-tech Industry by Tax Incentives under the Background of Yangtze River Delta Integration(No: 2021XJSK03).

## MHBM24151: THE CULTURAL HERITAGE ATTRIBUTES AND PSYCHOLOGICAL HEALING VALUE OF THE TANG-TUBO (XIZANG) ANCIENT ROAD

Lirong Zeng ^a^

^a^ *School of Ethnology and History, Academy of Xixia Studies, Ningxia University, Yinchuan 750021, China.*

**Objective:** In contemporary times, cultural heritage research places a growing emphasis on understanding the intrinsic cultural qualities of heritage, with the comprehension of its value becoming a core focus. Particularly, the psychological healing effects and value of cultural heritage for the public have been recognized internationally across various fields. However, in China, research and practices concerning cultural heritage often remain at the preservation level. This paper uses the Tang-Tubo (Xizang) Ancient Road as a case study to explore the inherent attributes of this cultural heritage and analyze its psychological healing value for individuals.

**Subjects and Methods:** The Tang-Tubo (Xizang) Ancient Road, a vital segment of the southern Silk Road, has historically served as a bridge for interethnic interaction, economic exchange, and cross-cultural integration. This study applies a literature-based research approach, focusing on the evolution of the “cultural route” concept and its criteria for attribute identification. It examines the nature of the Tang-Tubo (Xizang) Ancient Road as a cultural route from three perspectives: temporal-spatial attributes, cross-cultural attributes, and functional attributes. Furthermore, it assesses the psychological healing value of the route.

**Results:** (1) The Tang-Tubo (Xizang) Ancient Road, as a linear spatial entity, conforms to the criteria for defining a cultural route, representing a large-scale cross-regional cultural pathway. (2) This extensive natural and cultural landscape offers significant psychological healing benefits to residents and visitors of the heritage sites along the route in three key dimensions: ecological environment, artistic aesthetics, and emotional cohesion.

**Conclusions:** (1) The Tang-Tubo (Xizang) Ancient Road is a networked structure consisting of a main route and two auxiliary routes, forming a cultural route with clear boundaries. It has historically facilitated political exchanges, economic trade, and cultural dissemination, particularly the spread of Buddhist culture between the Tang Dynasty and the Tubo (Xizang) regime in ancient China. (2) The psychological healing value of the Tang-Tubo (Xizang) Ancient Road is evident in its unique and beautiful ecological environment, which provides psychological healing value; its historical architecture and cultural landscapes, which convey artistic aesthetics; and the emotional elements, such as national spirit and family patriotism, reflected along the route, which resonate emotionally with the public.

**Acknowledgements**

This work was supported by the National Social Science Fund Project “Study on the Cultural Route Heritage Connectivity of the Tang-Tubo (Xizang) Ancient Road” (Project No. 23XMZ037).

## MHBM24152: AN INVESTIGATION OF THE IMPACT OF FINANCIAL LITERACY ON FLEXIBLE EMPLOYEES BASED ON INSURANCE DECISION DRIVEN BY PSYCHOLOGICAL STRESS OF EMPLOYEES

Bo He^a^, Jumei Deng^b^, Yang Xu^a^

^a^ *Nanchang Institute of Science &Technology, Nanchang, 330108, China,* ^b^ *Nanchang Institute of Technology, Nanchang, 330044, China.*

**Background:** In recent years, China has formulated a series of unemployment insurance and flexible employment guarantee strategies aimed at improving residents’ job satisfaction. However, residents are reluctant to participate in insurance, which may be due to psychological barriers caused by their lack of financial literacy, such as bias against employment insurance, risk aversion and dissatisfaction with benefits. Moreover, it is unclear whether employment insurance is effective in alleviating flexible workers’ aversion to insecure work. While previous research has explored the factors that influence employment decisions, few studies have examined the role of financial literacy. A growing body of behavioral scientific evidence suggests that residents are significantly influenced by cognitive biases, heuristic thinking, and psychological factors when making employment insurance decisions that may exacerbate their aversion to flexible employment. As an effective way to relieve residents’ anxiety and dissatisfaction with unemployment, flexible employment has been paid more and more attention. This paper aims to explore how financial literacy affects residents’ employment insurance decisions and its impact on satisfaction and aversion to flexible employment.

**Subject and Method:** This study is based on behavioral science and psychology, exploring the relationship between residents’ financial literacy and employment insurance decisions, and further analyzing how financial literacy affects the psychological pressure and aversion experienced by residents in the process of flexible employment. The data comes from the 2019 China Household Finance Survey (CHFS), with a sample of 2423 flexible employees. In addition to personal and family characteristics, risk preferences are also included in the analysis. In order to fully consider cognitive biases and behavioral tendencies that may affect individual decision-making, a generalized ordered logit regression model is used to analyze the determining factors of insurance participation, revealing the potential role of financial literacy in reducing psychological pressure and dispelling aversion in flexible employment.

**Result:** Financial literacy can significantly increase residents’ willingness to participate in insurance and reduce their psychological pressure on unemployment, thereby lowering their aversion to flexible employment. In addition, financial literacy can help improve residents’ awareness of the benefits of employment insurance, reduce negative emotions caused by insufficient or misunderstood information, and thus enhance their willingness to participate. Intermediary analysis shows that financial knowledge can alleviate the financial exclusion that flexible employment workers may experience, thereby increasing their participation in various insurance plans. This impact is particularly evident among urban residents, risk averse individuals, and risk neutral groups, indicating that financial literacy plays an important role in helping these groups reduce psychological stress and dissatisfaction with flexible employment.

**Conclusion:** Improving financial knowledge is the key to promoting the participation of flexible workers in employment insurance and increasing job satisfaction. Enhancing financial knowledge enables flexible workers to make more rational and informed employment decisions, overcome cognitive biases that hinder insurance participation, thereby alleviating psychological stress and increasing their satisfaction with flexible employment. In addition, the improvement of financial knowledge helps to reduce negative emotions caused by financial difficulties, promote wealth accumulation and asset protection, thereby enhancing financial stability and life satisfaction. By obtaining insurance, residents can reduce potential risks in their lives, alleviate economic burdens, and cultivate a stronger sense of security, further reducing aversion caused by uncertainty.

**Acknowledgements**

This study was supported by the Social Sciences of Jiangxi Province 14th Five-Year Plan (2023) Fund Project (23YJ15), the research on the Application of Digital Technology to Support the Needs of Diverse Learners in University Classrooms, 2024 University-level Higher Education Teaching Reform Research Project of Guangzhou Xinhua University, Project No. 2024J063 and the research on the cultivation path of College Students’ Entrepreneurial Practical Ability Enabled by Digital Technology, Education and Teaching Reform Planning project of China Association of Business Economics in 2024, Project number: 20251068.

## MHBM24153: AN EMPIRICAL ANALYSIS OF URBAN HOUSE PRICE AND STOCK MARKET VOLATILITY IN CHINA AND THE UNITED STATES UNDER THE PUBLIC HEALTH CRISIS EVENT

Yucheng Zhuang^a,†^, Tao Li^b,†^, Huiwen Guo^c,†^, Tao Wang^d,e,†^, Wei Ding^e,†^

^a^ *The Chinese University of Hong Kong, Shatin, N.T., Hong Kong 999077, China;* ^b^ *Management College, Ocean University of China, Qingdao 266100, Shandong, China;* ^c^ *School of Public Policy and Administration, Chongqing University, Chongqing 400044, People’s Republic of China;* ^d^ *School of Management Science and Engineering, Shandong University of Finance and Economics, 250014, China;* ^e^ *Graduate School of International Studies, Hanyang University, Seoul 04763, Republic of Korea.*

^†^ *These authors contributed equally and are considered co-first authors*

**Background:** The global public health crisis event in recent years has profoundly impacted economies and mental well-being worldwide, with significant effects on the real estate and stock markets of major economies like China and the United States. Lockdowns, economic disruptions, and supply chain issues not only led to market volatility but also exacerbated stress and anxiety levels among the population.

**Subjects and Methods:** This study empirically analyzes the interplay between urban housing prices and stock market volatility in China and the US during the public health crisis event, incorporating considerations for the potential impact on mental health. Utilizing the Vector Autoregressive (VAR) model, the research investigates monthly data from the Shanghai Stock Exchange (SSE) and New York Stock Exchange (NYSE) Composite Indices, alongside corresponding housing price indices, from 2020 to 2022. The study employs ADF and KPSS tests for data stationarity, Granger causality tests to establish directional relationships, JJ cointegration tests to verify long-term correlations, and impulse response functions and variance decomposition for dynamic analysis. Additionally, mental health indicators such as stress levels and anxiety prevalence are discussed in the context of market volatility.

**Results:** The findings reveal a notable connection between real estate price shifts and stock market dynamics in both countries, with implications for mental health. In China, fluctuations in the stock market were found to Granger-cause variations in urban housing prices, potentially exacerbating financial stress and anxiety. Conversely, in the US, variations in urban housing prices preceded and influenced stock market volatility, again with potential mental health ramifications. Both markets showed cointegration, suggesting long-term correlations. Impulse response analysis highlighted a strong responsiveness between the two indices, with real estate prices showing particular sensitivity. Variance decomposition analysis revealed that while the stock market largely fuels its own volatility, real estate prices have a growing impact on stock market fluctuations, potentially compounding mental health concerns related to financial uncertainty.

**Conclusions:** The study concludes that during the public health crisis event, real estate prices played a potentially dominant role in influencing stock market fluctuations in both China and the US, with significant implications for mental health. Policy recommendations include tailored fiscal and monetary policies to stabilize the real estate market, enhanced financial market transparency and regulation, and increased support for SMEs to mitigate financial vulnerabilities. Additionally, addressing mental health through counseling services, financial education, and community support programs is crucial to help individuals cope with market-related stress and anxiety. By implementing these comprehensive strategies, policymakers can aim to maintain market equilibrium, foster long-term economic growth, and safeguard public mental well-being.

**Acknowledgements**

This work was supported by a project the Taishan Scholars in Shandong Province (Grant tsqnz20240830).

## MHBM24154: OPTIMIZATION ANALYSIS OF THE BUSINESS MODEL OF NEW ENERGY VEHICLES DURING THE PUBLIC HEALTH EVENT: INTEGRATING MENTAL HEALTH IMPACT - TAKING NIO’S BAAS MODEL IN NORWAY AS AN EXAMPLE

Zichao Xue^a,†^, Hailin Zhu^b,†^, Tao Wang^b,c,†^, Xu Jin^d^, Huiwen Guo^e,†^

^a^ *School of Law, Shandong University of Finance and Economics, Jinan 250014, Shandong, China;* ^b^ *School of Management Science and Engineering, Shandong University of Finance and Economics, Jinan 250014, Shandong, China;* ^c^ *Chinese Studies of Hanyang University, Graduate School of International Studies, Hanyang University, Seoul 04763, Republic of Korea;* ^d^ *Department of Global Business, Konkuk University, Seoul 04763, Republic of Korea;* ^e^ *School of Public Policy and Administration, Chongqing University, Chongqing 400044, Sichuang, China.*

^†^ *These authors contributed equally and are considered co-first author.*

**Background:** The ongoing global public health crisis has profoundly reshaped the world economy and consumer behavior patterns, presenting significant challenges to various industries, including the new energy vehicle (NEV) sector. NIO, a leading Chinese NEV manufacturer, has achieved remarkable success with its innovative Battery-as-a-Service (Baas) business model in the domestic market. To further expand its global presence, NIO chose Norway as its strategic entry point into the European market in 2019. However, the public health event pandemic has introduced numerous uncertainties and challenges to NIO’s expansion plans in Norway, such as supply chain disruptions, economic volatility, and shifting consumer preferences. Additionally, the crisis has heightened mental health concerns among both employees and consumers, underscoring the importance of integrating mental well-being into business strategies.

**Subjects and Methods:** This study delves into the optimization and analysis of NIO’s Baas business model implementation strategy in the Norwegian market, with a particular focus on the impact of mental health on business operations. Utilizing Porter’s Diamond Theory, PEST analysis, SWOT analysis, and other strategic frameworks, the research examines the macroeconomic, political, social, and technological factors influencing NIO’s market entry and expansion in Norway. It also assesses NIO’s internal strengths, weaknesses, opportunities, and threats, incorporating the influence of mental health on employee performance, consumer behavior, and brand loyalty. By doing so, the study aims to provide a comprehensive understanding of NIO’s current market position and the multifaceted challenges it faces.

**Results:** The findings reveal that NIO encounters numerous challenges in Norway, including the need for localized adaptations to its Baas model, intense competition from established players, and limited brand recognition. The pandemic has exacerbated mental health issues among employees and consumers, affecting productivity, purchasing decisions, and brand perception. In response, the study proposes a multifaceted strategy that emphasizes localization efforts, strategic partnerships, technological innovation, and the establishment of a comprehensive mental health support system. These strategies aim to address both operational and mental health aspects, thereby enhancing NIO’s market competitiveness, improving employee well-being, and fostering consumer trust and loyalty.

**Conclusions:** This study underscores the criticality of integrating mental health considerations into business strategies, especially in the context of a global public health crisis. By addressing NIO’s challenges in Norway through a holistic approach that balances operational efficiency and employee/consumer well-being, this research provides valuable insights for NIO and other Chinese NEV brands aiming to expand into international markets. The proposed strategies not only aim to optimize NIO’s Baas business model but also contribute to the broader discourse on corporate social responsibility and mental health in the workplace. As the global NEV industry evolves, a focus on mental health and sustainable business practices will be paramount for long-term success in an increasingly competitive and interconnected world.

**Acknowledgements**

This work was supported by a project the Taishan Scholars in Shandong Province (Grant tsqnz20240830).

## MHBM24155: RESEARCH ON THE COUPLING AND COORDINATION DEGREE OF PHARMACEUTICAL MANUFACTURING INDUSTRY AND REGIONAL ECONOMY IN YANGTZE RIVER ECONOMIC BELT FROM THE PERSPECTIVE OF HEALTHCARE SYSTEM

Jiangxue Di^a,b^, Siying Zhang^a,b^, Ningjie Wu^a,b^, Hanmei Jiang^a^, Xin Wen^a^

^a^ *Hubei University of Chinese Medicine, Wuhan, Hubei, China,* ^b^ *Research center for the development of traditional Chinese medicine, key research institute, Wuhan, Hubei, China.*

**Background:** By investigating the coordinated development level between the pharmaceutical manufacturing industry and the regional economy in the Yangtze River Economic Belt of our country, this provides a reference basis for enhancing the efficacy of the healthcare system, improving health management levels, and optimizing the industrial structure to promote coordinated development among regions.

**Subjects and Methods:** With reference to the “China High-tech Industry Statistical Yearbook in 2016-2022” and the “China Statistical Yearbook in 2016-2022, relevant data of 11 provinces (municipalities directly under the Central Government) along the Yangtze River Economic Belt from 2016 to 2022 were collected.In light of the development status of healthcare systems and economic in the Yangtze River Economic Belt, an integrated evaluation index system and a coupling coordination model for the pharmaceutical manufacturing industry and the regional economy are constructed to analyze the coupling and coordinated relationship between the pharmaceutical manufacturing industry and the regional economic development in the Yangtze River Economic Belt

**Results:** During the period from 2016 to 2022, the comprehensive development level of the pharmaceutical manufacturing industry and the regional economy in the Yangtze River Economic Belt of our country has been continuously improving, but the growth rate is relatively slow. In terms of coordinated development, there indeed exists a dynamic coupling relationship between the pharmaceutical manufacturing industry and the regional economy in the Yangtze River Economic Belt. Due to geography and healthy resource limitations in different provinces, the development of healthcare system and regional economy is imbalance.The coupling coordination level of downstream provinces and cities along the Yangtze River is higher than that of the middle-stream cities, and the coordinated development level of the middle-stream cities is higher than that of the upstream cities within the region.

**Conclusion:** In view of the situation where there exists a degree of disparity in the coordinated development of pharmaceutical manufacturing industry and the regional economy in the Yangtze River Economic Belt, it is proposed that the relevant provinces should expedite the adjustment of their industrial structures and advance industrial transfer. The government should through formulating relevant policies and plans, increasing fixed investment and enhancing infrastructure construction, provide a forceful policy guarantee for the healthcare system and health management, thereby facilitating the coordinated development of the pharmaceutical manufacturing industry and the regional economy.

**Acknowledgments**

This work was supported by youth foundation of Hubei University of Chinese medicine (2022ZZXQ06).

## MHBM24156: STRESS MANAGEMENT FOR COLLEGE STUDENTS IN THE DIGITAL ERA A NEW PATH FOR INTEGRATING MANAGEMENT PSYCHOLOGY AND TECHNOLOGY

Minjie Li^a^, Xianzhong Lin^b^

^a^ *Guangxi University of Finance and Economics, Nanning, China,* ^b^ *School of Marxism, Shenzhen Polytechnic University, Guangdong, Shenzhen, China.*

**Background:** In the digital era, college students grapple with escalating psychological stress, including heightened academic competition, social media-induced anxiety, job insecurity, and information overload. The Blue Book reveals a surge in depression and anxiety among Chinese students, underscoring the urgency of mental health concerns. To bolster psychological resilience and self-efficacy amidst these stressors, our study integrates management psychology with technology. It aims to develop tailored stress management programs that leverage AI, big data, and VR to enhance coping mechanisms and foster mental well-being in the digital age.

**Subjects and Methods:** In the digital age, college students confront a myriad of stressors, from academic rivalry to social media anxiety, job uncertainty, and digital overload, contributing to rising levels of depression and anxiety. This paper delves into the multifaceted stress experienced by students, highlighting how personal factors, social settings, and tech habits shape stress perception. We propose a fusion of management psychology with cutting-edge tech to craft stress management solutions. These include a digital mental health platform, an app for stress reduction, and VR for relaxation, all designed to fortify students’ psychological resilience and self-efficacy, equipping them with personalized tools to navigate the digital landscape and safeguard their mental well-being.

**Results:** This study uncovers the complex stressors of college students in the digital age, noting how personal traits, social contexts, and tech habits influence stress and anxiety. It advocates for a blend of psychological strategies and tech to boost students’ resilience and self-efficacy. Digital tools, by personalizing stress relief, bolster mental fortitude and psychological well-being, crucial for ethical tech advancement that safeguards students’ privacy and mental health.

**Conclusions:** This study dissects the stressors of the digital era for college students, unveiling how personal factors, social dynamics, and tech habits shape stress perception. It champions a fusion of managerial psychology and tech to arm students with resilience and stress-coping mechanisms. Proposed solutions, like a digital mental health platform and VR relaxation, aim to fortify students’ psychological well-being and self-efficacy amidst anxiety and depression.

**Acknowledgments**

This article is a research on the path to improving the conversational skills of college counselors based on psychological counseling techniques (Project No. 2023XJ04), and it is a research outcome of the Guangxi University of Finance and Economics’ annual youth scientific research project for the year 2023.

## MHBM24157: EXPLORING THE IMPROVEMENT OF COLLEGE STUDENTS’ MENTAL HEALTH LITERACY FROM THE PERSPECTIVE OF POSITIVE PSYCHOLOGY

Chaobo Pang ^a^

^a^ *Guangxi Vocational Normal University, Nanning, Guangxi, 530000, China.*

**Background:** Positive psychology advocates the use of scientific research methods to explore human positive qualities, fully stimulate individual potential, and enable them to achieve a sense of happiness. In terms of research methods, positive psychology has absorbed the vast majority of research methods and tools from traditional mainstream psychology, and organically combined these research methods and tools with humanistic phenomenological methods, empirical analysis methods, etc. Applying positive psychology to college students’ mental health education can effectively solve the problems and challenges faced by college students’ mental health, improve the effectiveness of college students’ mental health teaching, promote the development process of college students’ mental health, and thus ensure the mental health of college students.

**Subjects and Methods:** Combining positive psychology theory with mental health education, exploring how to improve the mental health level of college students from the perspective of positive psychology is of great significance. This study summarizes relevant literature from previous research, first elaborating on the importance of improving the mental health literacy of college students, analyzing the difficulties and challenges in improving their mental health literacy, and proposing corresponding solutions from the perspective of positive psychology, providing reference and guidance for universities to strengthen the psychological construction of college students and guide them to cultivate positive psychological qualities.

**Results:** According to the research findings, it can be found that most college students currently have poor psychological resilience and lack a positive attitude due to long-term stay in comfortable campus environments, protection from schools and teachers, and lack of contact with society. In addition, there are significant problems with mental health education in universities, and some universities do not have corresponding mental health counseling courses, which makes students more blind when they or others have psychological problems. Therefore, it is particularly important to analyze and improve the mental health literacy of college students from the perspective of health psychology.

**Conclusions:** According to the research results, students’ mental health literacy should be improved from the following aspects. Firstly, multiple forces should be gathered, and different disciplines should adopt different methods to work together to improve students’ mental health literacy. Secondly, the comprehensive improvement of mental health literacy needs to be combined with the ideological and political work platform in universities, and innovative exploration carriers and projects are needed. Thirdly, we should seek more targeted, unique, and effective plans to improve mental health literacy from different perspectives.

## MHBM24158: DIGITAL TRANSFORMATION OF IDEOLOGICAL AND ETHICAL EDUCATION FOR COLLEGE TEACHERS: INTEGRATING MENTAL HEALTH PERSPECTIVES THROUGH BIG DATA ANALYTICS

Xiaodan Zhou^a^, Xianzhong Lin^b^

^a^ *Guangxi University of Finance and Economics, Nanning, China,* ^b^ *Shenzhen Polytechnic University, Guangdong, Shenzhen, China.*

**Background:** The digital revolution presents opportunities and challenges for the ideological and moral education of university teachers, with a growing emphasis on mental health and well-being. Technologies such as AI, big data, and cloud computing are reshaping education, necessitating universities to prepare their faculty for digital demands, particularly in moral education, which is integral to fostering a mentally healthy academic environment. This transformation is critical for enhancing teacher quality, maintaining academic integrity, and supporting mental health, all of which are interconnected.

**Subjects and Methods:** This study investigates the impact of digital transformation on university teachers’ moral education through the lens of big data, aiming to boost outcomes and teacher development. It assesses the current state of moral education, the challenges of digital transformation, and the potential of big data in education. Employing a mixed-methods approach, it integrates big data analysis with case studies to pinpoint issues in digital transformation and suggests strategies like personalized plans and outcome evaluations that consider mental health indicators. Case studies showcase practical digital transformation in moral education, offering insights for other institutions and aiming to optimize education through data-driven methods that also promote psychological well-being.

**Results:** The study reveals that big data can tailor education plans to individual teacher needs, enhancing engagement, achievement, and mental health. Data-driven feedback mechanisms improve the effectiveness of teaching strategies and contribute to a supportive learning environment. Case studies, like the “Moral Compass” platform, exemplify successful digital integration, fostering interactive learning, collaboration, and psychological resilience. These findings underscore big data’s role in enhancing the precision and quality of moral education, as well as mental health support in higher education.

**Conclusions:** Digital transformation in moral education for university teachers is essential for educational relevance, effectiveness, and the promotion of mental health. Big data analytics is pivotal for personalizing education, assessing outcomes, and refining teaching strategies that also consider the mental well-being of educators. The study highlights the imperative for universities to embrace big data and emerging technologies to cultivate a dynamic, responsive, and ethical teacher education approach that nurtures mental health. The future of higher education hinges on the innovative application of these technologies to develop knowledgeable, morally adept, and psychologically resilient teachers in the digital age.

**Acknowledgments**

This article is the result of the following research projects. The 2021 Guangxi College Students’ Ideological and Political Education Theory and Practice Research Project “A Study on the Pathways to Enhancing the Political Leadership of University Counselors in the New Era” (2021MSZ049); and the Guangxi University of Finance and Economics 2022 Party Building and Ideological and Political Work Research Project “A Study on the Precision of University Counselors’ Ideological and Political Education from the Perspective of Big Data” (2022SZB08).

## MHBM24159: THE IMPACT OF WORK-FAMILY CONFLICT ON SUBJECTIVE WELL-BEING OF PHYSICAL EDUCATION TEACHERS

Wenmin Yu^a^, Shuaiwei Zheng^b^

^a^ *Qingdao University of Technology, Qingdao 66061, China,* ^b^ *Shandong Sport University, Jinan 250102, China.*

**Background:** The purpose of this study was to explore the influence of work-family conflict on physical education teachers’ subjective well-being, and to analyze the mediating role of mental resilience and job engagement in their relationship. Through the investigation and analysis of PE teachers, this paper explores the key factors that affect PE teachers’ subjective well-being, and provides theoretical basis and practical suggestions for the career development of educators, especially PE teachers. The results of this study are expected to provide valuable reference for improving the working environment, mental health and life quality of PE teachers.

**Subjects and Methods:** Using the Work-Family Conflict Scale, the Psychological Resilience Scale, the Work Involvement Scale and the Subjective Well-Being Scale, 425 physical education teachers in Province A were surveyed from 10 May 2024 to 20 August 2024 to explore the mechanisms of action through correlations and mediating effects.

**Results:** Key findings include: (1) Work-family conflict among PE teachers was below the moderate level, while psychological resilience, work engagement, and subjective well-being were above the moderate level. (2) Work-family conflict negatively correlated with psychological resilience, work engagement, and subjective well-being, while these three variables showed significant positive interrelations. (3) Work-family conflict influenced subjective well-being both directly and indirectly via psychological resilience and work engagement. The pathways identified were: (i) direct effects (work-family conflict → subjective well-being); (ii) independent mediating effects (work-family conflict → psychological resilience → subjective well-being and work-family conflict → work engagement → subjective well-being); and (iii) chain mediating effects (work-family conflict → psychological resilience → work engagement → subjective well-being).

**Conclusions:** This study shows that work-family conflict is an important factor affecting PE teachers’ subjective well-being. Specifically, work-family conflict indirectly affects physical education teachers’ subjective well-being by reducing their mental resilience and job engagement. Therefore, improving physical education teachers’ mental resilience and work engagement can effectively improve their subjective well-being and reduce the negative impact of conflict on their well-being.

## MHBM24160: THE IMPACT OF GLOBAL VALUE CHAIN EMBEDMENT ON CARBON EMISSIONS AND PSYCHOLOGICAL ANXIETY OF CHINA’S EQUIPMENT MANUFACTURING INDUSTRY

Runtian Pei^a^, Famgmiao Hou^b^, Xiaojian Li^a^, Feijiao Liu^a^

^a^ *Department of Economics and Trade, Shanxi Conservancy Technical Institute, Taiyuan, Shanxi 030032, China,* ^b^ *School of Economics and Management, Beijing Forestry University, Beijing 100083, China.*

**Background:** With the deepening of economic globalization, China’s importance in international trade is also increasing. However, there is no denying that China’s integration into global value chains, while reaping profits, has also brought badly needed solutions to environmental pollution, high carbon emissions and psychological anxiety.

**Subjects and Methods:** Although the equipment manufacturing industry is facing great psychological anxiety and pressure, as an important part of China’s manufacturing industry, it actively shoulders the responsibility of realizing China’s low-carbon and green development. Therefore, this paper takes the equipment manufacturing industry as the research object to explore the impact of its global value chain embedment level on carbon emissions. By calculating the global value chain (GVC) positive and backward participation and carbon emission levels of China’s equipment manufacturing industry and its sub-industries, taking technological progress, industrial scale and industrial structure as intermediary variables, the mechanism is further discussed.

**Results:** First, both backward and forward participation in the global value chain of the equipment manufacturing industry have a positive impact on carbon emissions at a significant level of 1%. Second, the backward and forward participation in the global value chain of the equipment manufacturing industry will have a positive impact on its carbon emissions through the intermediary variables of industry structure, technological progress and industry size. Third, China’s participation in the global value chain has indeed brought some psychological anxiety to the equipment manufacturing industry. This anxiety mainly stems from the concern about the competition in the international market, the concern about the level of their own technology, and the uncertainty of future development.

**Conclusions:** First of all, the equipment manufacturing industry needs to participate more actively in the global value chain, enhance the status of the international division of labor, accelerate the pace of technological innovation, break through key core technologies, and change the mode of economic growth. Second, promote structural upgrading and adjustment of the equipment manufacturing industry to improve the core competitiveness of enterprises. Third, gradually reduce the scale of backward industries with large carbon emissions and high pollution levels in the equipment manufacturing industry, increase policy support for industries with high innovation level and low pollution level, and enhance industrial competitiveness. Finally, China’s equipment manufacturing industry needs to actively cope with anxiety and pressure, strive to seek cooperation opportunities with international leading enterprises, learn international advanced management experience and market operation mode, and enhance the international competitiveness of enterprises.

**Acknowledgments**

This work was supported by “Research on the Quality Evaluation System of Vocational Higher Education in Water Conservancy Industry under the Perspective of Industry-Education Integration Community”grant from Yellow River Valley Education and Industry Alliance(Grant No.HHLYQN82),“Research on Green and Low-carbon Development Path and Policy of China’s Forest Product Industry under the ‘Dual-carbon’” grant from National Social Science Fund (Grant No.22BJY222); “Tracking And Evaluating the Implementation Effect of Forest Chief System in Beijing” grant from Beijing Landscaping Planning And Resource Monitoring Center (Grant No.GJH-2023-013). The authors are grateful for constructive comments and suggestions, as well as criticisms, provided by the peer reviewers and editors. Any remaining errors are solely our own.

## MHBM24161: STUDY ON THE EFFECT OF EMOTIONAL FREEDOM TECHNIQUES ON THE COGNITION OF PAIN CATASTROPHIZING IN PATIENTS AFTER MIXED HEMORRHOID SURGERY

Xinxin Liu^a^, Yining Wang^b^, Wen Liu^a^, Qian Wang^a^, Yan Li^a^, Yiwei Zhang^a^, Lu Liu^a^, Hong Xia^a^, Haoyu Niu^c^

^a^ *Medical School, Jingchu University of Technology, Jingmen 448000, China,* ^b^ *Guangzhou Nanyang Polytechnic College, Guangzhou 510900, China.,* ^c^ *Xuanhua Vocational College of Science & Technology, Zhangjiakou 075100, China.*

XL and YW contributed equally to this research

**Objective:** to investigate the efficacy of Emotional Freedom Techniques (EFT) in mitigating the catastrophic cognition of pain among patients who have undergone mixed hemorrhoid surgery. Hemorrhoid surgery can lead to significant postoperative pain, which is often accompanied by psychological distress and anxiety. These psychological factors can exacerbate the perception of pain and hinder the recovery process. EFT, a form of psychological acupressure, is hypothesized to alleviate these negative emotions and improve the patient’s cognitive response to pain, thereby enhancing the overall recovery experience.

**Methods:** A total of 90 patients with mixed hemorrhoids were randomly divided into a control group and an experimental group using the random number table method, with 45 cases in each group. The control group was given routine nursing intervention, and the experimental group was given emotional freedom techniques on the basis of the control group. The study observed the improvement of catastrophic pain cognition, postoperative pain relief, and nursing satisfaction in both groups. EFT, which includes psychological support and stress reduction techniques, was implemented to alleviate the psychological burden and anxiety associated with postoperative pain, thereby enhancing the overall mental well-being of patients.

**Results:** After the intervention, the scores of pain catastrophizing cognition and pain in experimental group were significantly lower than those in control group (P<0.01), The experimental group also reported higher levels of nursing satisfaction compared to the control group (P<0.01). These results indicate that EFT can effectively reduce pain levels, anxiety, and improve patient satisfaction with nursing care. The reduction in pain catastrophizing cognition suggests an improvement in patients’ mental health and a positive impact on their psychological state.

**Conclusion:** Emotional freedom techniques can effectively reduce the pain level, reduce anxiety and improve nursing satisfaction of patients after mixed hemorrhoid surgery. By incorporating psychological interventions such as EFT, hospitals can improve patients’ cognition of pain catastrophizing after surgery, alleviate their pain catastrophizing, and improve their nursing satisfaction. This approach not only addresses the physical symptoms but also the mental health aspects of patient recovery, leading to better postoperative outcomes and an enhanced quality of life for patients.

**Acknowledgements**

This research was supported by the Jingchu Institute of Technology Science and Technology Project (China, No.: QN202420). Jingmen City Education Science “14th Five-Year Plan” 2024 Annual Research Project (JMG2024008). Humanities and Social Sciences Research Project of the Provincial Department of Education (23z623).

## MHBM24162: INVESTIGATION ON PSYCHOLOGICAL ADJUSTMENT STRATEGIES FOR COLLEGE STUDENTS BASED ON PHYSICAL FITNESS TESTS

Zhiqiang Long ^a^

^a^ *School of Physical Education and Health of Wuzhou University, Wuzhou, 543002 China.*

**Objective:** Physical fitness tests are crucial for evaluating college students’ overall health. These assessments provide a comprehensive understanding of students’ physical conditions through scientific testing methods. Traditional physical fitness tests often focus solely on physiological metrics, overlooking the strong correlation between physical and psychological health. Investigating this relationship is essential for thorough evaluations of students’ well-being and for developing effective psychological health education strategies.

**Methods:** This research, based on physical fitness tests, focuses on the body mass and psychological health of college students at a specific university, primarily using quantitative research methods. The study employs testing software as its main tool, combining non-scale methods (such as student interviews, observations, and questionnaire surveys) with scaled methods (including psychological health assessments and analyses under sampled operationalization). Through a holistic examination of college students’ psychological health, the study gathers extensive data, which are analyzed inductively to develop theories and understand students’ psychological health and meaning construction through interactions with the research subjects.

**Results:** In evaluating 10,564 students using the 90-item Symptom Checklist (SCL-90), the Self-Rating Anxiety Scale (SAS), and the Self-Rating Depression Scale (SDS), the study found that 25.83% exhibited some degree of psychological health issues. Specifically, 2.50% were identified with moderate anxiety, 0.56% with severe anxiety, 9.73% with moderate depression, and 0.66% with severe depression. The overall results indicate a generally positive trend in the psychological health of college students, with most maintaining good psychological health. However, a segment of the student population experiences psychological challenges, such as anxiety and depressive disorders.

**Conclusions:** The research systematically explores psychological adjustment strategies for college students based on physical fitness tests. It delineates the close relationship between physical fitness metrics and psychological health, revealing how different physical fitness indicators potentially impact psychological well-being. Considering the unique attributes of the college student demographic, the study proposes comprehensive, actionable strategies: enhancing psychological health education and promotion, establishing a personalized psychological counseling service system, encouraging physical exercise and outdoor activities, promoting social events and teamwork, and developing psychological crisis intervention mechanisms. These strategies aim to help college students manage psychological pressures more effectively and improve their overall psychological health.

## MHBM24163: ART THERAPY AND MENTAL HEALTH: THE PSYCHOLOGICAL EFFECTS OF CULTURAL IMAGERY IN IMMERSIVE PERFORMANCES

Yuan Li^a^, Yuelan Xu^a^

^a^ *Xianda College of Economics and Humanities Shanghai International Studies University, Shanghai, China.*

**Objective:** This study aims to explore the role of immersive virtual exhibitions combined with art therapy in promoting mental health, investigating how these two work synergistically to meet psychological needs and enhance individual mental health levels. It analyzes the practical effects of immersive exhibitions as a new type of mental health intervention, revealing its advantages and challenges in the field of mental health.

**Methods:** To achieve the research objectives, this paper employs grounded theory as the research method. We collect user reviews from review websites and conduct interviews with 20 viewers, which serve as the data source for analysis. The data undergoes a three-level coding process to assess the changes in participants’ mental health status before and after the immersive exhibition. Based on the participants’ psychological needs, we construct a model regarding the psychological effects of cultural imagery in immersive exhibitions.

**Results:** Research findings indicate that the combination of immersive exhibitions and art therapy can effectively meet individuals’ psychological needs, such as self-expression, emotional release, and social interaction. After participating in immersive exhibitions, participants generally feel more relaxed, with reduced levels of anxiety and depression. Narrative imagery, geographical imagery, scenic imagery, interactive imagery, and technological imagery are the main factors that constitute the psychological effects of cultural imagery on audiences; they collectively influence the audience’s psychological experience, enhancing the sense of immersion and therapeutic effects of the exhibition. The combination of immersive exhibitions and art therapy can alleviate anxiety, reduce sensitivity, relieve depression, dispel loneliness, and chase away despondency, providing a new form of support for mental health. Through this innovative therapeutic method, participants can not only experience the beauty of art but also find solace in their artistic participation and experience, thus gaining positive effects on mental health and personal growth.

**Conclusions:** Comprehensive analysis reveals that combining immersive exhibitions with art therapy as a method to promote mental health has multiple advantages, including transcending the limitations of time and space, fostering interaction, stimulating creativity, and providing effective therapeutic outcomes, demonstrating significant potential and value. This integration offers individuals a safe and controlled space to delve into and express their inner world, thereby positively impacting mental health. It meets the demand for engaging and personalized therapeutic experiences through a variety of interactive and creative activities, contributing to the innovative development and widespread dissemination of art therapy.

**Acknowledgments**

This work was supported by a project grant from 2023 Shanghai Undergraduate First-Class Course “Exhibition Space Design” (Grant No. A3111.24.0701.015).

## MHBM24164: THE IMPACT OF FOOTBALL ON COLLEGE STUDENTS’ INTERPERSONAL SENSITIVITY: A MODERATED MEDIATION MODEL

Miao Shen^a,b^, Sihang Wang^b^, Junyu Tang^a^, Pei Li^b^, Jianlin Gong^a^

^a^ *School of Physical Education, Guangdong University of Technology, Guangzhou, China,* ^b^ *School of Physical Education & Sports Science, South China Normal University, Guangzhou, China.*

**Objective:** The purpose of this study is to thoroughly explore the impact of football on the interpersonal sensitivity of college students, dissect the mediating role of psychological resilience and the moderating role of self-efficacy within it, so as to provide a scientific basis for promoting the mental health of college students.

**Subjects and Methods:** A total of 586 college students who opted for football courses were chosen as the research participants from 20 universities across China by means of a questionnaire survey. The testing process involved the utilisation of the Physical Activity Scale, Symptom Checklist-90, General Self-Efficacy Scale, and Mental Resilience Scale. For the confirmatory factor analysis, AMOS 26.0 was put into use, while SPSS 24.0 was employed for the hypothesis testing.

**Results:** The research findings indicate that football participation is significantly negatively correlated with interpersonal sensitivity and significantly positively correlated with psychological resilience. The mediating effect of psychological resilience between football and interpersonal sensitivity accounts for 84.75% of the total effect. Self-efficacy has a significant moderating effect on the relationship between football participation and interpersonal sensitivity and has a significant positive predictive effect on the psychological resilience of college students. Specifically, as the amount of football exercise increases, the interpersonal sensitivity of college students decreases; psychological resilience mediates the relationship between football and interpersonal sensitivity, that is, football improves psychological resilience, which in turn indirectly reduces interpersonal sensitivity; self-efficacy moderates this mediating process, and the higher the self-efficacy, the stronger the positive impact of psychological resilience on interpersonal sensitivity.

**Conclusions:** Participation in football can have a positive impact on improving the interpersonal sensitivity of college students through the mediating role of psychological resilience and the moderating role of self-efficacy, which is worthy of promotion. However, this study has certain limitations. For example, the cross-sectional research method used cannot clarify the temporal sequence of changes among variables, and the influence of external social factors on self-efficacy, psychological resilience, and interpersonal relationships has not been fully considered. Future research should combine longitudinal research methods to deeply explore the longitudinal relationships among various factors in football, and use experimental methods to strictly control irrelevant variables to further investigate the impact of football on individuals’ cognition, emotion, and behaviour. At the same time, the scope of the survey and the sample size should be expanded to more comprehensively reveal the promoting effect of football on the physical and mental health of people in different regions and age groups.

**Acknowledgments**

This project was supported by the Project of Guangdong Philosophy and Social Sciences Planning. Project (GD24YTY05), and South China Normal University 2024 Graduate Student Research Innovation Program (43204027).

## MHBM24165: EXPLORING THE INTERVENTION MECHANISM OF MUSIC EDUCATION ON COLLEGE STUDENTS’ ANXIETY: FROM PSYCHOLOGICAL RESILIENCE TO EMOTION REGULATION

Shirang Wang ^a^

^a^ *Chongqing Normal University, Chongqing 401331, China*

**Background:** In recent years, anxiety has become more prevalent among college students as academic, social, and employment pressures increase. Excessive anxiety not only affects college students’ academic and social life, but also poses a serious threat to their overall psychological health. In response to this problem, music education has gradually attracted the attention of researchers as a means of emotion regulation and psychological intervention. Music education can effectively alleviate students’ anxiety and enhance their psychological resilience by providing a pathway for emotional release and satisfying individual psychological needs. Combined with Maslow’s hierarchy of needs theory, this study aims to explore the specific mechanism of music education in the intervention of college students’ anxiety, and to analyse how it can effectively regulate emotions and enhance psychological resilience by satisfying students’ needs for safety, social interaction and respect.

**Subjects and Methods:** The subjects of this study were college students with different levels of anxiety. Qualitative research methods were used to explore the role mechanisms of music education in meeting students’ psychological needs and alleviating anxiety based on in-depth interviews and case studies. Data collection included students’ emotional experiences and feedback during music relaxation training, receptive music therapy and group music activities. Meanwhile, the interaction between emotion regulation and psychological resilience was analysed based on Maslow’s hierarchy of needs theory, focusing on the performance and interrelationship of different levels of needs in anxiety intervention.

**Results:** The study found that music education has a positive effect in anxiety intervention through different forms. Music relaxation training mainly met students‘ safety needs and provided them with a pathway for emotional relief; receptive music therapy was significant in alleviating academic and social anxiety, helping students to meet their social and respect needs and enhance their sense of self-identity; and group music activities enhanced students’ sense of belonging and social support through emotional support and social needs fulfilment. Psychological resilience and emotion regulation interact in music education, providing an effective dual mechanism for anxiety management.

**Conclusions:** Music education has demonstrated good intervention effects in college students’ mental health education. Through diversified educational forms and the process of need satisfaction, music education not only effectively relieves college students’ anxiety, but also plays a significant role in enhancing psychological resilience and promoting long-term psychological growth. This study provides new intervention perspectives and practical strategies for mental health education in colleges and universities, and suggests that music education programmes should be further promoted in colleges and universities to build a more comprehensive emotional management and psychological support system.

## MHBM24166: DEVELOPMENT PROCESS, CURRENT CHALLENGES, AND IMPLEMENTATION PATHWAYS OF MENTAL HEALTH WELFARE FOR CHILDBIRTH OF CHINA’S RURAL LEFT-BEHIND WOMEN——A STUDY OF THE IMPACT OF FERTILITY WELFARE POLICIES ON THE MENTAL NEED FOR CHILDBIRTH OF LEFT-BEHIND WOMEN IN L PROVINCE

Lei Zhang ^a^

^a^ *Department of Marxist, Liaoning University, Shenyang, Liaoning, 110036, China.*

**Background:** This paper examines the development of mental health welfare for childbirth of left-behind women in rural China, analyzing existing policies, identifying current challenges, and proposing pathways for improvement. It aims to understand how welfare policies impact the mental health and emotional well-being of left-behind women and to offer insights for enhancing mental health support in rural areas.

**Subjects and Methods:** Rural left-behind women in H village and D town in L province were taken as the research objects from 2022-2023. The research conducted a survey by visiting households and interviewing village officials. The survey questions mainly focused on three aspects: (1) How much subsidy do the left-behind women in this village currently receive? Is it covered by the urban and rural residents’ medical insurance? Is the reimbursement process complicated? How much does it usually cost to give birth to a child out-of-pocket? (2) Do the left-behind women in this village have the intention to have second or third child? (3) Will the small amount of urban and rural residents’ medical insurance subsidy affect the mental need for childbirth of the left-behind women and families in this village and mental health.

**Results:** The findings reveal that mental health challenges for childbirth among rural left-behind women in L province are closely tied to economic pressures and insufficient welfare support. Although some women receive subsidies, the amount is inadequate, and the reimbursement process is overly complex, leading to frustration and anxiety. Younger women expressed lower childbirth intentions, citing high costs of childbirth and child-rearing as significant stressors that negatively affect their mental well-being. The limited scope of welfare policies fails to address these mental health stressors effectively. Furthermore, the administrative difficulties in accessing subsidies exacerbate mental health challenges, showing that existing policies fall short in supporting emotional resilience and mental health stability among rural left-behind women.

**Conclusions:** In the context of rural revitalization, the mental health welfare system for childbirth of left-behind women has seen initial development but remains underdeveloped compared to urban regions. This research highlights systemic barriers, including insufficient subsidies and administrative inefficiencies, that hinder mental health support. Addressing these issues requires a combination of economic growth, simplified policy implementation, and increased awareness of mental health needs in rural communities. Strengthening mental health welfare for childbirth is essential for improving the well-being of rural left-behind women and fostering social and economic sustainability in China’s countryside.

**Acknowledgments**

This research was funded by the Liaoning Provincial Department of Education, with a funding amount of 20,000 RMB. The project name and number are “Research on the Fundamental Experience of Rural Women’s Welfare Development in Liaoning Province Since the New Era” (LJKR0054).

## MHBM24167: THE INFLUENCE OF NEGATIVE EMOTIONS ON ORAL ENGLISH DISORDER

Yadong Wang ^a^

^a^ *Nanchang Institute of Science and Technology, Nanchang, Jiangxi, China.*

**Background:** In order to study the influence of negative emotions such as anxiety, embarrassment, etc. on oral English disorders of college students, the author conducted a network survey on both English majors and non-English majors from the author’s institute and found out that about 90% of the respondents have certain level of oral English disorders. Based on the factors analysis (FA), the author extracted ten key factors that play a crucial role in the formation of oral English disorders of college students. And based on the confirmatory factors analysis(CFA),the author constructed ten CFA models and found out that seven CFA are fitted and three others are not fitted. Based on the structural equation modeles (SEM), the author constructed three intermediate effects SEM in an attempt to reveal the correlations and intermediate effects between the key factors.

**Subjects and Methods:** A total of 747 English majors and non-English majors from Nanchang Institute of Technology took part in the online survey. The main study methods employed in this paper are online questionnaire survey, maximum likelihood estimation algorithms, EFA, CFA and SEM evaluation. In online survey stage, online questionnaire survey method is employed to collect influencing factors of negative emotions such as anxiety, embarrassment, shyness, inferiority, etc. on oral English disorders. In factor analysis stage, EFA method is employed to reduce the number of factors affecting oral English disorder and extract key factors. In factor verification analysis stage, measured models are built to test model fit. In SEM constructing stage, SEM method is used to explore the intermediate effects between different paths.

**Results:** The statistic analysis of the questionnaire showed that 90% of the students have oral English disorder of various degree. The factors analysis results showed ten key influencing factors play a dominant role. Ten measured models are conducted to test model fit. The CFA results showed that out of the 10 measured models, 7 are fitted while 3 others are not fitted. Three intermediate effect SEM are constructed to find out the intermediate effects between the key factors. The test results showed that there exists an intermediate effect between engage factor and class factor and that emotion factor has an intermediate effect on disorder factor through emotion to disorder path.

**Conclusions:** In conclusion, psychological factors are the chief reasons contribute to oral English disorder of college students.Among them, negative emotions, study engagement, lying flat attitude, poor motivation play a leading role. The study findings showed that psychological factors such as negative emotions has a major influence on students’ performance of learning habits and that poor engagement, poor base. Poor study habit have secondary influence. The factors’ intermediate effect analysis show that there is an intermediate effect between emotion and lying flat. For negative emotions can lower the level of engagement, and poor engagement will eventually result in lying flat. There also exists an intermediate effect between emotions and disorder. Because emotions affect class, class affects engage, engage affects disorder, forming a chain of indirect effects.

## MHBM24168: RESEARCH OF MUSIC CONTEXT-BASED EMOTIONAL REGULATION BASED ON CROSS-ATTENTION

Jinxiong Liao^a^, Shimin Wang^b^, Ming Zhao^c^

^a^ *Nanchang University of Aeronautics and Astronautics, Nanchang, Jiangxi 330000, China,* ^b^ *Jiangxi Normal University, Nanchang, Jiangxi 330000, China,* ^c^ *Jiangxi Institute of Applied Science and Technology, Nanchang, Jiangxi 330000, China.*

**Background:** Emotions are an indispensable part of human life, not only affecting mental health, but also directly influencing behavior and interpersonal relationships. Whether it’s work pressure, trivial matters in life, or interpersonal conflicts, it’s inevitable to experience various emotional fluctuations. Effectively controlling emotions can not only help people better cope with challenges in life, but also improve their quality of life.

**Subjects and Methods:** Research has shown that in order to effectively regulate emotions, music can be used to consciously shift attention away from the current emotional stimulus point, or temporarily leave a situation and go to a new place that is conducive to emotional adjustment. Therefore, this paper proposes a music context based emotional regulation based on cross attention. This includes the following steps: 1) Building a self-built sample dataset, decomposing music into 8 emotional elements based on the audience’s aesthetic characteristics and research on music. The self-built music sample dataset includes a self-built music collection and situational descriptions. 2) The effectiveness of data preprocessing and music emotion recognition is closely related to the representation of music features. Before data training, a series of data processing is required to meet the input format requirements of the model. 3) The method of feature extraction using long short-term memory networks extracts preprocessed datasets to obtain music data features and contextual description text features. 4) Cross-attention transformer feature fusion, which uses cross-attention transformer to fuse music data features and contextual description text features. 5) The prediction results can be captured separately by the cross attention Transformer, which can capture the paired relationship and channel dependency relationship between music data and contextual description features. Then, a linear layer is used to linearly change the data to obtain the output of the specified dimension, completing the model prediction.

**Results:** The experiment used a self-built sample dataset to test the proposed model, mainly analyzing the effects of music data and situational data on emotion regulation. The results showed that: 1) Music selection effect is significant, and choosing appropriate music can induce corresponding emotional reactions. 2) The interaction between music selection and context is significant, as music induces stronger emotional intensity in the context of interference.

**Conclusions:** Music choice has a significant impact on music emotions, and context has a significant impact on music emotions. Listening to music in a distracting context induces stronger emotions than in a focused context. This paper investigates the influence of music and context on emotional regulation, and also helps to understand the differences that individuals may have when listening to music through different choices in different situations, which has practical significance.

## MHBM24169: ASSOCIATION OF GLYCEMIC VARIABILITY WITH HEART FAILURE IN PATIENTS WITH TYPE 2 DIABETES MELLITUS AND THE GENERAL POPULATION: A SYSTEMATIC REVIEW AND META-ANALYSIS

Xiaohan Zhang^a,†^, Chengde Li^a,†^, Shumei Mao^a,†^, Haoming Li^b^, Jing Li^c^, Fang Wang^a^, Wenling Zheng^d^, Yan Huang^e^, Xuezheng Zhang^f^

^a^ *School of Pharmacy, Shandong Second Medical University, Weifang, Shandong 261053, China,* ^b^ *School of Management, Shandong Second Medical University, Weifang, Shandong 261053, China,* ^c^ *Department of Rehabilitation, Weifang Hospital of Traditional Chinese Medicine, Weifang, Shandong 261041, China,* ^d^ *Department of Invasive Technology, Weifang Hospital of Traditional Chinese Medicine, Weifang, Shandong 261041, China,* ^e^ *Department of Pediatrics, Weifang Hospital of Traditional Chinese Medicine, Weifang, Shandong 261041, China,* ^f^ *Department of Health Care Medicine, Weifang Hospital of Traditional Chinese Medicine, Weifang, Shandong 261041, China.*

^†^ *These authors contributed equally to this work.*

**Background:** This systematic review and meta-analysis aimed to investigate the association between glycemic variability (GV) and heart failure (HF) in patients with type 2 diabetes (T2DM) and the general population, and to examine both long-term and short-term glycemic variability as potential risk factors for heart failure.

**Subjects and Methods:** A rigorous systematic review was conducted using multiple electronic databases, including Embase, Web of Science, and PubMed, with an extensive search spanning from database inception to January 2019, and subsequently updated in April 2024. The research methodology focused on identifying and analyzing observational studies that reported on the association between glycemic variability and heart failure risk. Pooled data were analyzed using random-effects and fixed-effects models, with hazard ratios (HRs) and 95% confidence intervals (CIs) presented. Subgroup meta-analyses were performed based on glycemic variability indicators, including HbA1c-SD, FBG-HVS, and FBG-SD.

**Results:** The meta-analysis encompassed seven comprehensive studies involving a substantial cohort of 313,396 individuals, of whom 18,246 were diagnosed with heart failure. The findings revealed that both long-term and short-term glycemic variability were independently associated with an increased risk of heart failure in T2DM patients. The overall analysis demonstrated that glycemic variability was associated with a 30% increased risk of heart failure (HR 1.30, 95% CI 1.27 to 1.34, P < 0.001, I² = 29%). Subgroup analyses revealed that long-term glycemic variability (HbA1c-SD) was associated with a 32% increased risk (HR 1.32, 95% CI 1.28 to 1.37), while short-term glycemic variability (FBG-SD) was linked to a 33% increased risk (HR 1.33, 95% CI 1.27 to 1.40).

**Conclusions:** The study conclusively demonstrates that glycemic variability is independently associated with an elevated risk of heart failure in T2DM patients. Both short-term and long-term glycemic variability contribute significantly to this increased risk, highlighting the importance of managing glycemic fluctuations in reducing heart failure risk among T2DM patients.

